# Supplementary material for: Optimal minimal residual disease threshold in pediatric acute myeloid leukemia: A retrospective cohort study based on the TARGET database
Source: PLoS Med. 2026 May 8;23(5):e1005088. doi: 10.1371/journal.pmed.1005088 (PMC13155632; doi:10.1371/journal.pmed.1005088)
Supplement: S1 Checklist — This checklist is available under the Creative Commons Attribution 4.0 License (https://creativecommons.org/licenses/by/4.0/deed.en) and is adapted from the STROBE Statement (https://www.strobe-statement.org/). (DOCX) [file pmed.1005088.s001.docx]

STROBE Statement—checklist of items that should be included in reports of observational studies

|  | Item No | Recommendation | Page  No |
| --- | --- | --- | --- |
| **Title and abstract** | 1 | (*a*) Indicate the study’s design with a commonly used term in the title or the abstract | **Abstract section**: The title includes the term "retrospective cohort study" (title line), and the abstract explicitly states the design in the methods paragraph: "We conducted a retrospective cohort study" (Abstract section, methods paragraph，Line30-31). |
|  |  | (*b*) Provide in the abstract an informative and balanced summary of what was done and what was found | **Abstract section**: The abstract is structured into paragraphs: background (first paragraph), methods (second paragraph), results (third paragraph), and conclusions (fourth paragraph). It summarizes the study aim, methods, key findings (e.g., optimal threshold of 0.05%), and implications(Line 25-50, Line30-40, Line41-48）. |
| Introduction | | | |
| Background/rationale | 2 | Explain the scientific background and rationale for the investigation being reported | **Introduction section (Section 1)​**: The first paragraph discusses the prognosis of pediatric AML and the need for better biomarkers. Subsequent paragraphs review MRD monitoring, the conventional 0.1% threshold, and the rationale for reevaluating a lower threshold (Line 93-135, Line103-118, Line119-125). |
| Objectives | 3 | State specific objectives, including any prespecified hypotheses | **Introduction section, final paragraph**: States objectives. **Methods section, subsection "Hypotheses and Analysis Plan" (2.4)​**: Explicitly lists prespecified hypotheses (Line126-134）. |
| Methods | | | |
| Study design | 4 | Present key elements of study design early in the paper | **Abstract and Introduction sections**: The abstract mentions the retrospective cohort design. The introduction concludes with an overview of the study design, including data source (TARGET cohort) and objectives（Line126-134）. |
| Setting | 5 | Describe the setting, locations, and relevant dates, including periods of recruitment, exposure, follow-up, and data collection | **Methods section, subsection "Study Population and Data Source" (2.1)​**: Describes the setting (TARGET-AML initiative, COG trials), recruitment period (September 1996 to December 2016), and follow-up (median 6.2 years). Locations are implied through COG centers (Line137-155). |
| Participants | 6 | (*a*) *Cohort study*—Give the eligibility criteria, and the sources and methods of selection of participants. Describe methods of follow-up  *Case-control study*—Give the eligibility criteria, and the sources and methods of case ascertainment and control selection. Give the rationale for the choice of cases and controls  *Cross-sectional study*—Give the eligibility criteria, and the sources and methods of selection of participants | **Methods section, subsection "Study Population and Data Source" (2.1)​**: Eligibility criteria include pediatric de novo AML patients with available MRD data. Sources are the TARGET database (Line137-146). |
|  |  | (*b*) *Cohort study*—For matched studies, give matching criteria and number of exposed and unexposed  *Case-control study*—For matched studies, give matching criteria and the number of controls per case | Not applicable, as the study is not matched. |
| Variables | 7 | Clearly define all outcomes, exposures, predictors, potential confounders, and effect modifiers. Give diagnostic criteria, if applicable | **Methods section**: Outcomes like 5-year EFS are defined in the "Statistical Analysis" subsection (paragraph on EFS definition). Exposures (MRD thresholds) are defined in "MRD Assessment" subsection. Confounders are listed in "Study Population" and Table 1. Diagnostic criteria for CR are in "Treatment Protocols" subsection（Line141-145）. |
| Data sources/ measurement | 8* | For each variable of interest, give sources of data and details of methods of assessment (measurement). Describe comparability of assessment methods if there is more than one group | **Methods section, subsection "MRD Assessment"(2.3)**: Details MRD measurement by flow cytometry, including sensitivity and standardization. Data sources are described in "Study Population" subsection. No multiple groups with different methods, so comparability not discussed (Line177-188). |
| Bias | 9 | Describe any efforts to address potential sources of bias | Limited explicit description. Bias is indirectly addressed through standardized MRD protocols and statistical adjustments (e.g., multivariable models in "Statistical Analysis" subsection), but no dedicated section on bias mitigation（Line177-183）. |
| Study size | 10 | Explain how the study size was arrived at | The sample size is based on available data from the TARGET cohort (Methods section, "Study Population" paragraph), but no power calculation or justification is provided（Line137-140）. |
| Quantitative variables | 11 | Explain how quantitative variables were handled in the analyses. If applicable, describe which groupings were chosen and why | **Methods section, subsection "Statistical Analysis" (2.5)​**: Describes handling of MRD as continuous (for ROC) and categorical (groupings like <0.05%, 0.05-<0.1%, ≥0.1%). The "Hypotheses" subsection explains the rationale for threshold comparisons（Line240-278）. |
| Statistical methods | 12 | (*a*) Describe all statistical methods, including those used to control for confounding | **Methods section, subsection "Statistical Analysis" (2.5)​**: Lists methods (Kaplan-Meier, Cox regression, ROC, NRI). Multivariable models control for confounders （Line248-252）. |
|  |  | (*b*) Describe any methods used to examine subgroups and interactions | **Methods section, subsection "Subgroup Analysis" (2.5.5)​**: Describes stratified Cox models for genetic risk subgroups（Line277-281）. |
|  |  | (*c*) Explain how missing data were addressed | Not explicitly discussed, though Table 1 shows missing data for some variables (e.g., WT1 status). |
|  |  | (*d*) *Cohort study*—If applicable, explain how loss to follow-up was addressed  *Case-control study*—If applicable, explain how matching of cases and controls was addressed  *Cross-sectional study*—If applicable, describe analytical methods taking account of sampling strategy | Follow-up time is reported, but no details on loss to follow-up（Line34-35）. |
|  |  | (*e*) Describe any sensitivity analyses | **Methods section, subsection "ROC Curve Analysis" (2.5.3)​**: Describes sensitivity analysis weighting sensitivity over specificity for threshold optimization（Line264-267）. |

Continued on next page

| Results | | | |
| --- | --- | --- | --- |
| Participants | 13* | (a) Report numbers of individuals at each stage of study—eg numbers potentially eligible, examined for eligibility, confirmed eligible, included in the study, completing follow-up, and analysed | **Results section, subsection "Study Participants" (3.1)​**: Reports total n=1,205 and groups (event vs. non-event), but no flowchart or stage-wise numbers（Line 292-297）. |
|  |  | (b) Give reasons for non-participation at each stage | Not provided. |
|  |  | (c) Consider use of a flow diagram | No flow diagram is included or mentioned. |
| Descriptive data | 14* | (a) Give characteristics of study participants (eg demographic, clinical, social) and information on exposures and potential confounders | **Results section, subsection "Study Participants" (3.1) and Table 1**: Provides detailed baseline characteristics, including demographics, clinical factors, and MRD levels（Line654-696）. |
|  |  | (b) Indicate number of participants with missing data for each variable of interest | Table 1 shows some variables with missing data (e.g., WT1 status), but no systematic reporting of missing numbers. |
|  |  | (c) *Cohort study*—Summarise follow-up time (eg, average and total amount) | **Methods section, "Study Population" paragraph**: Reports median follow-up of 6.2 years (range 0.5-20.1 years)（Line Line34-35）. |
| Outcome data | 15* | *Cohort study*—Report numbers of outcome events or summary measures over time | **Results section**: Reports event numbers (n=611 events vs. n=594 non-events) and EFS rates over time (e.g., 5-year EFS in survival analyses)（Line322-328）. |
|  |  | *Case-control study—*Report numbers in each exposure category, or summary measures of exposure | **Results section**: Unadjusted estimates in Table 2; adjusted estimates in Table 3 (multivariable Cox model). Confounders adjusted include WT1 mutation, SCT, etc., with model selection based on AIC (Methods section). |
|  |  | *Cross-sectional study—*Report numbers of outcome events or summary measures | **Results section**: Reports event numbers (n=611 events vs. n=594 non-events) and EFS rates over time (e.g., 5-year EFS in survival analyses). |
| Main results | 16 | (*a*) Give unadjusted estimates and, if applicable, confounder-adjusted estimates and their precision (eg, 95% confidence interval). Make clear which confounders were adjusted for and why they were included | **Results section**: Unadjusted estimates in Table 2; adjusted estimates in Table 3 (multivariable Cox model). Confounders adjusted include WT1 mutation, SCT, etc., with model selection based on AIC (Methods section)（Line662-668）. |
|  |  | (*b*) Report category boundaries when continuous variables were categorized | **Methods section, subsection "MRD Assessment" (2.3)​**: Categories defined as <0.05%, 0.05-<0.1%, and ≥0.1%(Line187-188). |
|  |  | (*c*) If relevant, consider translating estimates of relative risk into absolute risk for a meaningful time period | **Results section, survival analyses**: Reports absolute risks (e.g., 5-year EFS percentages) in addition to hazard ratios（Line322-328）. |
| Other analyses | 17 | Report other analyses done—eg analyses of subgroups and interactions, and sensitivity analyses | **Results section**: Includes subgroup analyses (by genetic risk), NRI analysis, and sensitivity analysis (ROC weighting). Described in subsections like "Subgroup Analysis" and "NRI Analysis"(Line277-28、Line400-411）. |
| Discussion | | | |
| Key results | 18 | Summarise key results with reference to study objectives | **Discussion section (Section 4)​**: First paragraph summarizes key results (e.g., superiority of 0.05% threshold), linking back to objectives stated in the Introduction（Line413-431）. |
| Limitations | 19 | Discuss limitations of the study, taking into account sources of potential bias or imprecision. Discuss both direction and magnitude of any potential bias | **Discussion section, later paragraphs**: Discusses limitations like retrospective design, heterogeneity across trials, and technological evolution, addressing potential bias directions（Line485-507）. |
| Interpretation | 20 | Give a cautious overall interpretation of results considering objectives, limitations, multiplicity of analyses, results from similar studies, and other relevant evidence | **Discussion section**: Interprets results cautiously, compares with similar studies, and considers limitations. Conclusion section reinforces interpretation（Line413-517）. |
| Generalisability | 21 | Discuss the generalisability (external validity) of the study results | **Discussion section**: Notes that findings may apply to modern regimens but require prospective validation, addressing generalizability（Line493-495）. |
| Other information | | | |
| Funding | 22 | Give the source of funding and the role of the funders for the present study and, if applicable, for the original study on which the present article is based | **Declarations section**: Lists funding sources (e.g., Guangdong grants), but does not specify the funders' roles（Line548-561）. |

*Give information separately for cases and controls in case-control studies and, if applicable, for exposed and unexposed groups in cohort and cross-sectional studies.

**Note:** An Explanation and Elaboration article discusses each checklist item and gives methodological background and published examples of transparent reporting. The STROBE checklist is best used in conjunction with this article (freely available on the Web sites of PLoS Medicine at http://www.plosmedicine.org/, Annals of Internal Medicine at http://www.annals.org/, and Epidemiology at http://www.epidem.com/). Information on the STROBE Initiative is available at www.strobe-statement.org.
