# Supplementary material for: Optimal minimal residual disease threshold in pediatric acute myeloid leukemia: A retrospective cohort study based on the TARGET database
Source: PLoS Med. 2026 May 8;23(5):e1005088. doi: 10.1371/journal.pmed.1005088 (PMC13155632; doi:10.1371/journal.pmed.1005088)
Supplement: S1 Code — (ZIP) [file pmed.1005088.s002.zip › S2 code/PROJ8_2_tbl/PROJ8_2_tbl.htm]

## Ô¤²âÄ£ÐÍÓëROC·ÖÎö

Please use EmpowerStats ver. 2.2 or 3.0 since R packages updated, results from this version may be inaccurate.
Outcome:
First Event
Time:
X28

Model 0: Multiple Fractional Polynomial model from observed data
0.27028\*log(((X25 + 0.1)/10))

|  |  |  |  |  |  |  |
| --- | --- | --- | --- | --- | --- | --- |
|  | coeff. | se. | HR | Low 95%CI | High 95%CI | P value |
| log(((X25 + 0.1)/10)) | 0.2703 | 0.0512 | 1.3103 | 1.1852 | 1.4486 | <0.0001 |

rsq = 0.2780, maxrsq = 0.9992
, Log likelihood -318.1816

|  |  |  |  |  |  |  |
| --- | --- | --- | --- | --- | --- | --- |
| concordant | discordant | tied.risk | tied.time | std(c-d) | C | se(C) |
| 2621 | 1242 | 263 | 230 | 315.7571 | 0.6671 | 0.0383 |

Create new variables for Nomogram: 
V.new1 = ((X25+0.1)/10)
Model 1: Full model from observed data
0.02242\*X25

|  |  |  |  |  |  |  |
| --- | --- | --- | --- | --- | --- | --- |
|  | coeff. | se. | HR | Low 95%CI | High 95%CI | P value |
| X25 | 0.0224 | 0.0046 | 1.0227 | 1.0135 | 1.0320 | <0.0001 |

rsq = 0.2033, maxrsq = 0.9992
, Log likelihood -322.8083

|  |  |  |  |  |  |  |
| --- | --- | --- | --- | --- | --- | --- |
| concordant | discordant | tied.risk | tied.time | std(c-d) | C | se(C) |
| 2621 | 1242 | 263 | 230 | 315.7571 | 0.6671 | 0.0383 |

|  |  |  |  |  |  |  |  |  |  |
| --- | --- | --- | --- | --- | --- | --- | --- | --- | --- |
| Obs | Events | Model L.R. | d.f. | P.value | Score | Score P | R2 | g | gr |
| 94 | 93 | 21.36 | 1 | <0.0001 | 25.656 | 0 | 0.2034 | 0.5781 | 1.7826 |

Ô¤²âÄ£ÐÍROCÇúÏß·ÖÎö¼°×î¼ÑãÐÖµ·ÖÎö

|  |  |  |  |  |  |  |  |  |  |  |
| --- | --- | --- | --- | --- | --- | --- | --- | --- | --- | --- |
| Model | Time | Best.cut.X | Best.cut.sens | Best.cut.spec | Cases | N.survivor | N.censored | Cum.incidence | Surv.prob | AUC |
| mfp | 0.19 | 0.365597765654369 | 0.563 | 0.746 | 16 | 67 | 11 | 0.17 | 0.7128 | 0.628 |
| mfp | 0.2 | 0.433096453262956 | 0.556 | 0.73 | 27 | 63 | 4 | 0.287 | 0.6702 | 0.626 |
| mfp | 0.21 | 0.63426569551517 | 0.677 | 0.635 | 31 | 52 | 11 | 0.33 | 0.5532 | 0.657 |
| mfp | 0.22 | 0.679853092714179 | 0.738 | 0.69 | 42 | 42 | 10 | 0.447 | 0.4468 | 0.727 |
| mfp | 0.23 | 0.679853092714179 | 0.769 | 0.765 | 52 | 34 | 8 | 0.553 | 0.3617 | 0.777 |
| mfp | 0.25 | 0.679853092714179 | 0.746 | 0.857 | 63 | 28 | 3 | 0.67 | 0.2979 | 0.813 |
| mfp | 0.3 | 0.723988204823196 | 0.792 | 0.947 | 72 | 19 | 3 | 0.766 | 0.2021 | 0.864 |
| Full | 0.19 | -0.148105138741546 | 0.563 | 0.746 | 16 | 67 | 11 | 0.17 | 0.7128 | 0.628 |
| Full | 0.2 | -0.0584123976414425 | 0.556 | 0.73 | 27 | 63 | 4 | 0.287 | 0.6702 | 0.626 |
| Full | 0.21 | 0.390051307859076 | 0.677 | 0.635 | 31 | 52 | 11 | 0.33 | 0.5532 | 0.657 |
| Full | 0.22 | 0.547013604784257 | 0.738 | 0.69 | 42 | 42 | 10 | 0.447 | 0.4468 | 0.727 |
| Full | 0.23 | 0.547013604784257 | 0.769 | 0.765 | 52 | 34 | 8 | 0.553 | 0.3617 | 0.777 |
| Full | 0.25 | 0.547013604784257 | 0.746 | 0.857 | 63 | 28 | 3 | 0.67 | 0.2979 | 0.813 |
| Full | 0.3 | 0.726399086984465 | 0.792 | 0.947 | 72 | 19 | 3 | 0.766 | 0.2021 | 0.864 |

×î¼ÑãÐÖµÈ¡Ãô¸Ð¶È+ÌØÒì¶È×î´óµÄ·Ö½çÖµ¡£¸÷·Ö½çµã¶ÔÓ¦µÄÃô¸Ð¶ÈÌØÒì±£´æÔÚROCÊä³öÎÄ¼þ£¨.xls£©Àï
Inverse Probability of Censoring Weighting (IPCW) estimates of Cumulative/Dynamic time-dependent ROC curve.

Use subset of data: (is.na(X22) | (X22==1)) & (is.na(X28) | (X28<0.5))
