# Supplementary material for: Optimal minimal residual disease threshold in pediatric acute myeloid leukemia: A retrospective cohort study based on the TARGET database
Source: PLoS Med. 2026 May 8;23(5):e1005088. doi: 10.1371/journal.pmed.1005088 (PMC13155632; doi:10.1371/journal.pmed.1005088)
Supplement: S1 Code — (ZIP) [file pmed.1005088.s002.zip › S2 code/PROJ8_7_tbl1/PROJ8_7_tbl1.htm]

## ÑÐ¾¿ÈËÈºÃèÊö

|  |  |
| --- | --- |
|  |  |
|  | Mean(SD) Median (Min-Max) |
| WBC | 65.4 (93.7) 25.9 (0.6-918.5) |
| BM blast(%) | 64.5 (24.2) 70.0 (0.0-100.0) |
| Peripheral blasts (%) | 43.3 (31.1) 42.0 (0.0-99.0) |
| MRD % at end of course 1 | 3.1 (11.6) 0.0 (0.0-92.0) |
| MRD % at end of course 2 | 1.9 (9.0) 0.0 (0.0-75.0) |
|  | N (%) |
| Gender |  |
| Male | 624 (51.8%) |
| Female | 581 (48.2%) |
| Age group |  |
| £¼10 | 656 (54.4%) |
| ¡Ý10 | 549 (45.6%) |
| Chemotherapy Protocol |  |
| AAML1031 | 669 (55.5%) |
| AAML0531 | 473 (39.3%) |
| AAML03P1 | 63 (5.2%) |
| Risk group |  |
| Low risk | 483 (40.8%) |
| Standard risk | 574 (48.4%) |
| High risk | 128 (10.8%) |
| Karyotype |  |
| MLL | 256 (21.7%) |
| t(8;21) | 183 (15.5%) |
| inv(16) | 146 (12.4%) |
| Normal | 279 (23.6%) |
| Other | 317 (26.8%) |
| FLT3-ITD Status |  |
| FLT3-ITD-wt | 1021 (84.7%) |
| FLT3-ITD-mt | 184 (15.3%) |
| NPM1 Status |  |
| NPM1-wt | 1086 (90.4%) |
| NPM1-mt | 115 (9.6%) |
| CEBPA Status |  |
| CEBPA-wt | 1124 (93.7%) |
| CEBPA-mt | 75 (6.3%) |
| WT1 Status |  |
| WT1-wt | 492 (92.5%) |
| WT1-mt | 40 (7.5%) |
| NUP214 |  |
| Negative | 1180 (97.9%) |
| Positive | 25 (2.1%) |
| NUP98 |  |
| Negative | 1168 (96.9%) |
| Positive | 37 (3.1%) |
| FAB Category |  |
| M0 | 37 (4.1%) |
| M1 | 119 (13.3%) |
| M2 | 265 (29.7%) |
| M4 | 206 (23.1%) |
| M5 | 199 (22.3%) |
| M6 | 10 (1.1%) |
| M7 | 56 (6.3%) |
| CNS disease |  |
| CNS1 | 451 (69.7%) |
| CNS2 | 145 (22.4%) |
| CNS3 | 51 (7.9%) |
| Chloroma |  |
| No | 754 (89.2%) |
| Yes | 91 (10.8%) |
| CR status at end of course 1 |  |
| CR | 959 (80.2%) |
| Not in CR | 237 (19.8%) |
| CR status at end of course 2 |  |
| CR | 1107 (93.0%) |
| Not in CR | 81 (6.8%) |
| Death | 2 (0.2%) |
| MRD at end of course 1 |  |
| Negative | 924 (76.7%) |
| Positive | 281 (23.3%) |
| MRD at end of course 2 |  |
| Negative | 1073 (89.0%) |
| Positive | 132 (11.0%) |
| SCT in 1st CR |  |
| No | 943 (84.1%) |
| Yes | 178 (15.9%) |

±íÖÐ½á¹û:
Mean(SD) Median (Min-Max) / N(%)
´Ë±íÓÃÒ×õÍ³¼ÆÈí¼þ (www.empowerstats.com) ºÍRÈí¼þÉú³É£¬Éú³ÉÈÕÆÚ£º 2025-10-07
