# Supplementary material for: Optimal minimal residual disease threshold in pediatric acute myeloid leukemia: A retrospective cohort study based on the TARGET database
Source: PLoS Med. 2026 May 8;23(5):e1005088. doi: 10.1371/journal.pmed.1005088 (PMC13155632; doi:10.1371/journal.pmed.1005088)
Supplement: S1 Code — (ZIP) [file pmed.1005088.s002.zip › S2 code/PROJ8_13_tbl1/PROJ8_13_tbl1.htm]

## Kaplan Meier Survival Curve

Outcome: First Event
Time: EFS(years)
Landmark analysis for time segment: EFS(years) <= 5
Log rank test: implements the G-rho family of Harrington and Fleming (1982), with weights on each death of S(t)^rho, where S is the Kaplan-Meier estimate of survival. With rho = 0 this is the log-rank or Mantel-Haenszel test.

|  |  |  |  |
| --- | --- | --- | --- |
|  | N | Observed | Expected |
| factor(X23.CS)=0 | 399 | 228 | 287.3848 |
| factor(X23.CS)=1 | 175 | 143 | 83.6152 |

Chisq=
55.2303
on
1
degree of freedom, p=
<0.0001
Survival table

|  |  |  |  |  |  |  |  |
| --- | --- | --- | --- | --- | --- | --- | --- |
|  | EFS(years) | N.Risk | N.Event | N.Censor | Survival | 95%CI Low | 95%CI Upp |
| factor(X23.CS)=0 | 0.16 | 399 | 1 | 0 | 0.9975 | 0.9926 | 1.0000 |
| factor(X23.CS)=0 | 0.17 | 398 | 3 | 0 | 0.9900 | 0.9802 | 0.9998 |
| factor(X23.CS)=0 | 0.18 | 395 | 2 | 0 | 0.9850 | 0.9731 | 0.9970 |
| factor(X23.CS)=0 | 0.19 | 393 | 0 | 1 | 0.9850 | 0.9731 | 0.9970 |
| factor(X23.CS)=0 | 0.2 | 392 | 4 | 0 | 0.9749 | 0.9597 | 0.9904 |
| factor(X23.CS)=0 | 0.21 | 388 | 2 | 0 | 0.9699 | 0.9533 | 0.9868 |
| factor(X23.CS)=0 | 0.23 | 386 | 0 | 1 | 0.9699 | 0.9533 | 0.9868 |
| factor(X23.CS)=0 | 0.26 | 385 | 2 | 0 | 0.9648 | 0.9469 | 0.9831 |
| factor(X23.CS)=0 | 0.27 | 383 | 2 | 1 | 0.9598 | 0.9407 | 0.9793 |
| factor(X23.CS)=0 | 0.28 | 380 | 4 | 0 | 0.9497 | 0.9285 | 0.9714 |
| factor(X23.CS)=0 | 0.29 | 376 | 1 | 0 | 0.9472 | 0.9254 | 0.9694 |
| factor(X23.CS)=0 | 0.31 | 375 | 1 | 0 | 0.9447 | 0.9224 | 0.9674 |
| factor(X23.CS)=0 | 0.32 | 374 | 1 | 0 | 0.9421 | 0.9194 | 0.9654 |
| factor(X23.CS)=0 | 0.33 | 373 | 2 | 0 | 0.9371 | 0.9135 | 0.9613 |
| factor(X23.CS)=0 | 0.37 | 371 | 1 | 0 | 0.9346 | 0.9105 | 0.9592 |
| factor(X23.CS)=0 | 0.38 | 370 | 2 | 0 | 0.9295 | 0.9047 | 0.9550 |
| factor(X23.CS)=0 | 0.4 | 368 | 4 | 0 | 0.9194 | 0.8930 | 0.9466 |
| factor(X23.CS)=0 | 0.41 | 364 | 1 | 0 | 0.9169 | 0.8901 | 0.9444 |
| factor(X23.CS)=0 | 0.42 | 363 | 1 | 0 | 0.9143 | 0.8872 | 0.9423 |
| factor(X23.CS)=0 | 0.44 | 362 | 3 | 0 | 0.9068 | 0.8786 | 0.9358 |
| factor(X23.CS)=0 | 0.45 | 359 | 1 | 0 | 0.9042 | 0.8757 | 0.9337 |
| factor(X23.CS)=0 | 0.46 | 358 | 2 | 0 | 0.8992 | 0.8700 | 0.9293 |
| factor(X23.CS)=0 | 0.47 | 356 | 1 | 0 | 0.8967 | 0.8672 | 0.9271 |
| factor(X23.CS)=0 | 0.5 | 355 | 2 | 0 | 0.8916 | 0.8615 | 0.9227 |
| factor(X23.CS)=0 | 0.51 | 353 | 1 | 0 | 0.8891 | 0.8587 | 0.9205 |
| factor(X23.CS)=0 | 0.52 | 352 | 3 | 0 | 0.8815 | 0.8503 | 0.9139 |
| factor(X23.CS)=0 | 0.53 | 349 | 2 | 0 | 0.8765 | 0.8447 | 0.9095 |
| factor(X23.CS)=0 | 0.54 | 347 | 2 | 0 | 0.8714 | 0.8391 | 0.9050 |
| factor(X23.CS)=0 | 0.55 | 345 | 1 | 0 | 0.8689 | 0.8363 | 0.9027 |
| factor(X23.CS)=0 | 0.56 | 344 | 6 | 0 | 0.8537 | 0.8196 | 0.8892 |
| factor(X23.CS)=0 | 0.57 | 338 | 1 | 0 | 0.8512 | 0.8169 | 0.8870 |
| factor(X23.CS)=0 | 0.58 | 337 | 3 | 0 | 0.8436 | 0.8086 | 0.8801 |
| factor(X23.CS)=0 | 0.59 | 334 | 4 | 0 | 0.8335 | 0.7976 | 0.8710 |
| factor(X23.CS)=0 | 0.6 | 330 | 2 | 0 | 0.8285 | 0.7922 | 0.8664 |
| factor(X23.CS)=0 | 0.62 | 328 | 2 | 0 | 0.8234 | 0.7867 | 0.8618 |
| factor(X23.CS)=0 | 0.63 | 326 | 1 | 0 | 0.8209 | 0.7840 | 0.8595 |
| factor(X23.CS)=0 | 0.64 | 325 | 1 | 0 | 0.8184 | 0.7813 | 0.8572 |
| factor(X23.CS)=0 | 0.65 | 324 | 4 | 0 | 0.8083 | 0.7704 | 0.8480 |
| factor(X23.CS)=0 | 0.67 | 320 | 1 | 0 | 0.8057 | 0.7677 | 0.8456 |
| factor(X23.CS)=0 | 0.68 | 319 | 2 | 0 | 0.8007 | 0.7623 | 0.8410 |
| factor(X23.CS)=0 | 0.69 | 317 | 4 | 0 | 0.7906 | 0.7515 | 0.8317 |
| factor(X23.CS)=0 | 0.7 | 313 | 2 | 0 | 0.7855 | 0.7461 | 0.8270 |
| factor(X23.CS)=0 | 0.71 | 311 | 2 | 0 | 0.7805 | 0.7408 | 0.8223 |
| factor(X23.CS)=0 | 0.72 | 309 | 4 | 0 | 0.7704 | 0.7301 | 0.8129 |
| factor(X23.CS)=0 | 0.73 | 305 | 4 | 0 | 0.7603 | 0.7194 | 0.8035 |
| factor(X23.CS)=0 | 0.74 | 301 | 1 | 0 | 0.7577 | 0.7167 | 0.8011 |
| factor(X23.CS)=0 | 0.75 | 300 | 1 | 0 | 0.7552 | 0.7140 | 0.7988 |
| factor(X23.CS)=0 | 0.76 | 299 | 5 | 0 | 0.7426 | 0.7008 | 0.7869 |
| factor(X23.CS)=0 | 0.77 | 294 | 1 | 0 | 0.7401 | 0.6981 | 0.7845 |
| factor(X23.CS)=0 | 0.78 | 293 | 2 | 0 | 0.7350 | 0.6928 | 0.7798 |
| factor(X23.CS)=0 | 0.79 | 291 | 2 | 0 | 0.7300 | 0.6875 | 0.7750 |
| factor(X23.CS)=0 | 0.8 | 289 | 3 | 0 | 0.7224 | 0.6796 | 0.7679 |
| factor(X23.CS)=0 | 0.81 | 286 | 2 | 0 | 0.7173 | 0.6743 | 0.7631 |
| factor(X23.CS)=0 | 0.82 | 284 | 2 | 0 | 0.7123 | 0.6691 | 0.7583 |
| factor(X23.CS)=0 | 0.83 | 282 | 4 | 0 | 0.7022 | 0.6586 | 0.7487 |
| factor(X23.CS)=0 | 0.84 | 278 | 1 | 0 | 0.6997 | 0.6559 | 0.7463 |
| factor(X23.CS)=0 | 0.86 | 277 | 1 | 0 | 0.6971 | 0.6533 | 0.7439 |
| factor(X23.CS)=0 | 0.88 | 276 | 3 | 0 | 0.6895 | 0.6455 | 0.7367 |
| factor(X23.CS)=0 | 0.89 | 273 | 1 | 0 | 0.6870 | 0.6428 | 0.7342 |
| factor(X23.CS)=0 | 0.9 | 272 | 2 | 0 | 0.6820 | 0.6376 | 0.7294 |
| factor(X23.CS)=0 | 0.92 | 270 | 4 | 0 | 0.6719 | 0.6272 | 0.7197 |
| factor(X23.CS)=0 | 0.93 | 266 | 6 | 0 | 0.6567 | 0.6116 | 0.7052 |
| factor(X23.CS)=0 | 0.95 | 260 | 1 | 0 | 0.6542 | 0.6090 | 0.7027 |
| factor(X23.CS)=0 | 0.96 | 259 | 0 | 1 | 0.6542 | 0.6090 | 0.7027 |
| factor(X23.CS)=0 | 0.99 | 258 | 1 | 0 | 0.6517 | 0.6064 | 0.7003 |
| factor(X23.CS)=0 | 1 | 257 | 3 | 0 | 0.6440 | 0.5986 | 0.6930 |
| factor(X23.CS)=0 | 1.01 | 254 | 2 | 0 | 0.6390 | 0.5934 | 0.6881 |
| factor(X23.CS)=0 | 1.02 | 252 | 2 | 0 | 0.6339 | 0.5882 | 0.6832 |
| factor(X23.CS)=0 | 1.03 | 250 | 2 | 0 | 0.6288 | 0.5830 | 0.6783 |
| factor(X23.CS)=0 | 1.05 | 248 | 2 | 0 | 0.6238 | 0.5778 | 0.6734 |
| factor(X23.CS)=0 | 1.07 | 246 | 3 | 0 | 0.6162 | 0.5701 | 0.6660 |
| factor(X23.CS)=0 | 1.08 | 243 | 2 | 0 | 0.6111 | 0.5649 | 0.6610 |
| factor(X23.CS)=0 | 1.1 | 241 | 1 | 0 | 0.6085 | 0.5623 | 0.6586 |
| factor(X23.CS)=0 | 1.12 | 240 | 2 | 0 | 0.6035 | 0.5572 | 0.6536 |
| factor(X23.CS)=0 | 1.13 | 238 | 1 | 0 | 0.6009 | 0.5546 | 0.6512 |
| factor(X23.CS)=0 | 1.14 | 237 | 2 | 0 | 0.5959 | 0.5494 | 0.6462 |
| factor(X23.CS)=0 | 1.18 | 235 | 1 | 0 | 0.5933 | 0.5469 | 0.6438 |
| factor(X23.CS)=0 | 1.19 | 234 | 2 | 0 | 0.5883 | 0.5417 | 0.6388 |
| factor(X23.CS)=0 | 1.2 | 232 | 1 | 0 | 0.5857 | 0.5391 | 0.6363 |
| factor(X23.CS)=0 | 1.21 | 231 | 0 | 1 | 0.5857 | 0.5391 | 0.6363 |
| factor(X23.CS)=0 | 1.26 | 230 | 1 | 0 | 0.5832 | 0.5366 | 0.6338 |
| factor(X23.CS)=0 | 1.27 | 229 | 2 | 0 | 0.5781 | 0.5314 | 0.6289 |
| factor(X23.CS)=0 | 1.28 | 227 | 1 | 0 | 0.5755 | 0.5288 | 0.6264 |
| factor(X23.CS)=0 | 1.3 | 226 | 2 | 0 | 0.5704 | 0.5237 | 0.6214 |
| factor(X23.CS)=0 | 1.34 | 224 | 3 | 0 | 0.5628 | 0.5160 | 0.6139 |
| factor(X23.CS)=0 | 1.35 | 221 | 1 | 0 | 0.5603 | 0.5134 | 0.6114 |
| factor(X23.CS)=0 | 1.36 | 220 | 2 | 0 | 0.5552 | 0.5083 | 0.6064 |
| factor(X23.CS)=0 | 1.37 | 218 | 1 | 0 | 0.5526 | 0.5057 | 0.6039 |
| factor(X23.CS)=0 | 1.38 | 217 | 2 | 0 | 0.5475 | 0.5006 | 0.5989 |
| factor(X23.CS)=0 | 1.39 | 215 | 2 | 0 | 0.5424 | 0.4955 | 0.5938 |
| factor(X23.CS)=0 | 1.4 | 213 | 1 | 0 | 0.5399 | 0.4929 | 0.5913 |
| factor(X23.CS)=0 | 1.41 | 212 | 1 | 0 | 0.5373 | 0.4904 | 0.5888 |
| factor(X23.CS)=0 | 1.42 | 211 | 3 | 0 | 0.5297 | 0.4827 | 0.5813 |
| factor(X23.CS)=0 | 1.43 | 208 | 3 | 0 | 0.5221 | 0.4750 | 0.5737 |
| factor(X23.CS)=0 | 1.45 | 205 | 1 | 0 | 0.5195 | 0.4725 | 0.5712 |
| factor(X23.CS)=0 | 1.49 | 204 | 1 | 0 | 0.5170 | 0.4700 | 0.5687 |
| factor(X23.CS)=0 | 1.51 | 203 | 1 | 0 | 0.5144 | 0.4674 | 0.5662 |
| factor(X23.CS)=0 | 1.53 | 202 | 1 | 0 | 0.5119 | 0.4649 | 0.5636 |
| factor(X23.CS)=0 | 1.56 | 201 | 1 | 0 | 0.5093 | 0.4623 | 0.5611 |
| factor(X23.CS)=0 | 1.58 | 200 | 3 | 0 | 0.5017 | 0.4547 | 0.5535 |
| factor(X23.CS)=0 | 1.6 | 197 | 2 | 0 | 0.4966 | 0.4496 | 0.5484 |
| factor(X23.CS)=0 | 1.62 | 195 | 2 | 0 | 0.4915 | 0.4446 | 0.5434 |
| factor(X23.CS)=0 | 1.64 | 193 | 1 | 0 | 0.4890 | 0.4420 | 0.5408 |
| factor(X23.CS)=0 | 1.72 | 192 | 1 | 0 | 0.4864 | 0.4395 | 0.5383 |
| factor(X23.CS)=0 | 1.75 | 191 | 0 | 1 | 0.4864 | 0.4395 | 0.5383 |
| factor(X23.CS)=0 | 1.77 | 190 | 1 | 0 | 0.4838 | 0.4370 | 0.5358 |
| factor(X23.CS)=0 | 1.89 | 189 | 2 | 0 | 0.4787 | 0.4319 | 0.5306 |
| factor(X23.CS)=0 | 1.9 | 187 | 2 | 0 | 0.4736 | 0.4268 | 0.5255 |
| factor(X23.CS)=0 | 1.92 | 185 | 1 | 0 | 0.4710 | 0.4243 | 0.5230 |
| factor(X23.CS)=0 | 2.05 | 184 | 1 | 0 | 0.4685 | 0.4217 | 0.5204 |
| factor(X23.CS)=0 | 2.07 | 183 | 1 | 0 | 0.4659 | 0.4192 | 0.5179 |
| factor(X23.CS)=0 | 2.08 | 182 | 1 | 0 | 0.4634 | 0.4167 | 0.5153 |
| factor(X23.CS)=0 | 2.11 | 181 | 1 | 0 | 0.4608 | 0.4141 | 0.5127 |
| factor(X23.CS)=0 | 2.13 | 180 | 1 | 0 | 0.4582 | 0.4116 | 0.5102 |
| factor(X23.CS)=0 | 2.15 | 179 | 1 | 0 | 0.4557 | 0.4091 | 0.5076 |
| factor(X23.CS)=0 | 2.18 | 178 | 0 | 1 | 0.4557 | 0.4091 | 0.5076 |
| factor(X23.CS)=0 | 2.2 | 177 | 1 | 0 | 0.4531 | 0.4065 | 0.5050 |
| factor(X23.CS)=0 | 2.22 | 176 | 2 | 0 | 0.4480 | 0.4015 | 0.4999 |
| factor(X23.CS)=0 | 2.23 | 174 | 0 | 1 | 0.4480 | 0.4015 | 0.4999 |
| factor(X23.CS)=0 | 2.34 | 173 | 1 | 0 | 0.4454 | 0.3989 | 0.4973 |
| factor(X23.CS)=0 | 2.36 | 172 | 1 | 0 | 0.4428 | 0.3963 | 0.4947 |
| factor(X23.CS)=0 | 2.43 | 171 | 0 | 1 | 0.4428 | 0.3963 | 0.4947 |
| factor(X23.CS)=0 | 2.49 | 170 | 0 | 1 | 0.4428 | 0.3963 | 0.4947 |
| factor(X23.CS)=0 | 2.5 | 169 | 0 | 1 | 0.4428 | 0.3963 | 0.4947 |
| factor(X23.CS)=0 | 2.51 | 168 | 0 | 1 | 0.4428 | 0.3963 | 0.4947 |
| factor(X23.CS)=0 | 2.56 | 167 | 0 | 1 | 0.4428 | 0.3963 | 0.4947 |
| factor(X23.CS)=0 | 2.61 | 166 | 1 | 1 | 0.4401 | 0.3937 | 0.4920 |
| factor(X23.CS)=0 | 2.62 | 164 | 2 | 0 | 0.4347 | 0.3884 | 0.4866 |
| factor(X23.CS)=0 | 2.63 | 162 | 0 | 1 | 0.4347 | 0.3884 | 0.4866 |
| factor(X23.CS)=0 | 2.89 | 161 | 0 | 1 | 0.4347 | 0.3884 | 0.4866 |
| factor(X23.CS)=0 | 2.98 | 160 | 0 | 1 | 0.4347 | 0.3884 | 0.4866 |
| factor(X23.CS)=0 | 3.05 | 159 | 0 | 1 | 0.4347 | 0.3884 | 0.4866 |
| factor(X23.CS)=0 | 3.07 | 158 | 0 | 1 | 0.4347 | 0.3884 | 0.4866 |
| factor(X23.CS)=0 | 3.16 | 157 | 0 | 1 | 0.4347 | 0.3884 | 0.4866 |
| factor(X23.CS)=0 | 3.18 | 156 | 1 | 2 | 0.4320 | 0.3856 | 0.4838 |
| factor(X23.CS)=0 | 3.25 | 153 | 0 | 1 | 0.4320 | 0.3856 | 0.4838 |
| factor(X23.CS)=0 | 3.28 | 152 | 0 | 1 | 0.4320 | 0.3856 | 0.4838 |
| factor(X23.CS)=0 | 3.29 | 151 | 0 | 1 | 0.4320 | 0.3856 | 0.4838 |
| factor(X23.CS)=0 | 3.3 | 150 | 0 | 1 | 0.4320 | 0.3856 | 0.4838 |
| factor(X23.CS)=0 | 3.33 | 149 | 0 | 1 | 0.4320 | 0.3856 | 0.4838 |
| factor(X23.CS)=0 | 3.35 | 148 | 0 | 2 | 0.4320 | 0.3856 | 0.4838 |
| factor(X23.CS)=0 | 3.36 | 146 | 0 | 1 | 0.4320 | 0.3856 | 0.4838 |
| factor(X23.CS)=0 | 3.38 | 145 | 0 | 2 | 0.4320 | 0.3856 | 0.4838 |
| factor(X23.CS)=0 | 3.39 | 143 | 1 | 1 | 0.4289 | 0.3826 | 0.4809 |
| factor(X23.CS)=0 | 3.4 | 141 | 0 | 1 | 0.4289 | 0.3826 | 0.4809 |
| factor(X23.CS)=0 | 3.41 | 140 | 0 | 3 | 0.4289 | 0.3826 | 0.4809 |
| factor(X23.CS)=0 | 3.44 | 137 | 0 | 2 | 0.4289 | 0.3826 | 0.4809 |
| factor(X23.CS)=0 | 3.45 | 135 | 0 | 1 | 0.4289 | 0.3826 | 0.4809 |
| factor(X23.CS)=0 | 3.46 | 134 | 0 | 1 | 0.4289 | 0.3826 | 0.4809 |
| factor(X23.CS)=0 | 3.48 | 133 | 0 | 1 | 0.4289 | 0.3826 | 0.4809 |
| factor(X23.CS)=0 | 3.5 | 132 | 0 | 1 | 0.4289 | 0.3826 | 0.4809 |
| factor(X23.CS)=0 | 3.56 | 131 | 0 | 1 | 0.4289 | 0.3826 | 0.4809 |
| factor(X23.CS)=0 | 3.62 | 130 | 0 | 1 | 0.4289 | 0.3826 | 0.4809 |
| factor(X23.CS)=0 | 3.63 | 129 | 0 | 1 | 0.4289 | 0.3826 | 0.4809 |
| factor(X23.CS)=0 | 3.81 | 128 | 0 | 1 | 0.4289 | 0.3826 | 0.4809 |
| factor(X23.CS)=0 | 3.84 | 127 | 0 | 1 | 0.4289 | 0.3826 | 0.4809 |
| factor(X23.CS)=0 | 3.96 | 126 | 0 | 1 | 0.4289 | 0.3826 | 0.4809 |
| factor(X23.CS)=0 | 3.99 | 125 | 1 | 0 | 0.4255 | 0.3792 | 0.4775 |
| factor(X23.CS)=0 | 4.04 | 124 | 0 | 1 | 0.4255 | 0.3792 | 0.4775 |
| factor(X23.CS)=0 | 4.07 | 123 | 1 | 0 | 0.4221 | 0.3757 | 0.4742 |
| factor(X23.CS)=0 | 4.08 | 122 | 0 | 1 | 0.4221 | 0.3757 | 0.4742 |
| factor(X23.CS)=0 | 4.19 | 121 | 0 | 2 | 0.4221 | 0.3757 | 0.4742 |
| factor(X23.CS)=0 | 4.22 | 119 | 0 | 1 | 0.4221 | 0.3757 | 0.4742 |
| factor(X23.CS)=0 | 4.23 | 118 | 1 | 0 | 0.4185 | 0.3720 | 0.4707 |
| factor(X23.CS)=0 | 4.27 | 117 | 0 | 1 | 0.4185 | 0.3720 | 0.4707 |
| factor(X23.CS)=0 | 4.31 | 116 | 0 | 1 | 0.4185 | 0.3720 | 0.4707 |
| factor(X23.CS)=0 | 4.34 | 115 | 0 | 1 | 0.4185 | 0.3720 | 0.4707 |
| factor(X23.CS)=0 | 4.35 | 114 | 0 | 1 | 0.4185 | 0.3720 | 0.4707 |
| factor(X23.CS)=0 | 4.38 | 113 | 0 | 2 | 0.4185 | 0.3720 | 0.4707 |
| factor(X23.CS)=0 | 4.41 | 111 | 0 | 1 | 0.4185 | 0.3720 | 0.4707 |
| factor(X23.CS)=0 | 4.42 | 110 | 0 | 1 | 0.4185 | 0.3720 | 0.4707 |
| factor(X23.CS)=0 | 4.44 | 109 | 0 | 1 | 0.4185 | 0.3720 | 0.4707 |
| factor(X23.CS)=0 | 4.45 | 108 | 0 | 1 | 0.4185 | 0.3720 | 0.4707 |
| factor(X23.CS)=0 | 4.47 | 107 | 0 | 1 | 0.4185 | 0.3720 | 0.4707 |
| factor(X23.CS)=0 | 4.48 | 106 | 0 | 1 | 0.4185 | 0.3720 | 0.4707 |
| factor(X23.CS)=0 | 4.49 | 105 | 0 | 2 | 0.4185 | 0.3720 | 0.4707 |
| factor(X23.CS)=0 | 4.5 | 103 | 0 | 1 | 0.4185 | 0.3720 | 0.4707 |
| factor(X23.CS)=0 | 4.53 | 102 | 0 | 1 | 0.4185 | 0.3720 | 0.4707 |
| factor(X23.CS)=0 | 4.58 | 101 | 0 | 2 | 0.4185 | 0.3720 | 0.4707 |
| factor(X23.CS)=0 | 4.6 | 99 | 0 | 1 | 0.4185 | 0.3720 | 0.4707 |
| factor(X23.CS)=0 | 4.62 | 98 | 0 | 1 | 0.4185 | 0.3720 | 0.4707 |
| factor(X23.CS)=0 | 4.79 | 97 | 0 | 1 | 0.4185 | 0.3720 | 0.4707 |
| factor(X23.CS)=0 | 4.85 | 96 | 0 | 3 | 0.4185 | 0.3720 | 0.4707 |
| factor(X23.CS)=0 | 4.87 | 93 | 0 | 2 | 0.4185 | 0.3720 | 0.4707 |
| factor(X23.CS)=0 | 4.9 | 91 | 0 | 1 | 0.4185 | 0.3720 | 0.4707 |
| factor(X23.CS)=0 | 4.99 | 90 | 0 | 1 | 0.4185 | 0.3720 | 0.4707 |
| factor(X23.CS)=0 | 5 | 89 | 0 | 89 | 0.4185 | 0.3720 | 0.4707 |
| factor(X23.CS)=1 | 0.15 | 175 | 1 | 0 | 0.9943 | 0.9832 | 1.0000 |
| factor(X23.CS)=1 | 0.16 | 174 | 3 | 0 | 0.9771 | 0.9552 | 0.9995 |
| factor(X23.CS)=1 | 0.17 | 171 | 1 | 0 | 0.9714 | 0.9471 | 0.9964 |
| factor(X23.CS)=1 | 0.18 | 170 | 1 | 0 | 0.9657 | 0.9391 | 0.9931 |
| factor(X23.CS)=1 | 0.19 | 169 | 7 | 0 | 0.9257 | 0.8877 | 0.9654 |
| factor(X23.CS)=1 | 0.2 | 162 | 3 | 0 | 0.9086 | 0.8669 | 0.9523 |
| factor(X23.CS)=1 | 0.21 | 159 | 10 | 0 | 0.8514 | 0.8003 | 0.9058 |
| factor(X23.CS)=1 | 0.22 | 149 | 7 | 0 | 0.8114 | 0.7555 | 0.8715 |
| factor(X23.CS)=1 | 0.23 | 142 | 7 | 0 | 0.7714 | 0.7117 | 0.8362 |
| factor(X23.CS)=1 | 0.24 | 135 | 2 | 0 | 0.7600 | 0.6993 | 0.8260 |
| factor(X23.CS)=1 | 0.25 | 133 | 2 | 0 | 0.7486 | 0.6870 | 0.8157 |
| factor(X23.CS)=1 | 0.27 | 131 | 2 | 0 | 0.7371 | 0.6747 | 0.8053 |
| factor(X23.CS)=1 | 0.28 | 129 | 1 | 0 | 0.7314 | 0.6686 | 0.8001 |
| factor(X23.CS)=1 | 0.3 | 128 | 3 | 0 | 0.7143 | 0.6504 | 0.7845 |
| factor(X23.CS)=1 | 0.32 | 125 | 1 | 0 | 0.7086 | 0.6443 | 0.7792 |
| factor(X23.CS)=1 | 0.33 | 124 | 0 | 1 | 0.7086 | 0.6443 | 0.7792 |
| factor(X23.CS)=1 | 0.35 | 123 | 2 | 0 | 0.6970 | 0.6322 | 0.7686 |
| factor(X23.CS)=1 | 0.36 | 121 | 1 | 0 | 0.6913 | 0.6261 | 0.7633 |
| factor(X23.CS)=1 | 0.37 | 120 | 1 | 0 | 0.6855 | 0.6201 | 0.7579 |
| factor(X23.CS)=1 | 0.43 | 119 | 1 | 0 | 0.6798 | 0.6140 | 0.7526 |
| factor(X23.CS)=1 | 0.45 | 118 | 1 | 0 | 0.6740 | 0.6080 | 0.7472 |
| factor(X23.CS)=1 | 0.47 | 117 | 1 | 0 | 0.6682 | 0.6020 | 0.7418 |
| factor(X23.CS)=1 | 0.48 | 116 | 2 | 0 | 0.6567 | 0.5900 | 0.7310 |
| factor(X23.CS)=1 | 0.49 | 114 | 2 | 0 | 0.6452 | 0.5780 | 0.7202 |
| factor(X23.CS)=1 | 0.52 | 112 | 1 | 0 | 0.6394 | 0.5720 | 0.7148 |
| factor(X23.CS)=1 | 0.53 | 111 | 1 | 0 | 0.6337 | 0.5661 | 0.7093 |
| factor(X23.CS)=1 | 0.54 | 110 | 1 | 0 | 0.6279 | 0.5602 | 0.7039 |
| factor(X23.CS)=1 | 0.55 | 109 | 2 | 0 | 0.6164 | 0.5483 | 0.6929 |
| factor(X23.CS)=1 | 0.56 | 107 | 1 | 0 | 0.6106 | 0.5424 | 0.6874 |
| factor(X23.CS)=1 | 0.57 | 106 | 1 | 0 | 0.6049 | 0.5365 | 0.6819 |
| factor(X23.CS)=1 | 0.58 | 105 | 1 | 0 | 0.5991 | 0.5306 | 0.6764 |
| factor(X23.CS)=1 | 0.59 | 104 | 1 | 0 | 0.5934 | 0.5248 | 0.6709 |
| factor(X23.CS)=1 | 0.6 | 103 | 1 | 0 | 0.5876 | 0.5189 | 0.6654 |
| factor(X23.CS)=1 | 0.64 | 102 | 1 | 0 | 0.5818 | 0.5130 | 0.6598 |
| factor(X23.CS)=1 | 0.68 | 101 | 3 | 0 | 0.5646 | 0.4955 | 0.6432 |
| factor(X23.CS)=1 | 0.69 | 98 | 2 | 0 | 0.5530 | 0.4839 | 0.6320 |
| factor(X23.CS)=1 | 0.7 | 96 | 4 | 0 | 0.5300 | 0.4608 | 0.6095 |
| factor(X23.CS)=1 | 0.72 | 92 | 1 | 0 | 0.5242 | 0.4551 | 0.6039 |
| factor(X23.CS)=1 | 0.74 | 91 | 2 | 0 | 0.5127 | 0.4436 | 0.5926 |
| factor(X23.CS)=1 | 0.75 | 89 | 1 | 0 | 0.5069 | 0.4379 | 0.5869 |
| factor(X23.CS)=1 | 0.76 | 88 | 2 | 0 | 0.4954 | 0.4265 | 0.5755 |
| factor(X23.CS)=1 | 0.77 | 86 | 2 | 0 | 0.4839 | 0.4151 | 0.5641 |
| factor(X23.CS)=1 | 0.78 | 84 | 2 | 1 | 0.4724 | 0.4038 | 0.5527 |
| factor(X23.CS)=1 | 0.79 | 81 | 2 | 0 | 0.4607 | 0.3923 | 0.5411 |
| factor(X23.CS)=1 | 0.81 | 79 | 3 | 0 | 0.4432 | 0.3752 | 0.5236 |
| factor(X23.CS)=1 | 0.82 | 76 | 2 | 0 | 0.4316 | 0.3638 | 0.5119 |
| factor(X23.CS)=1 | 0.84 | 74 | 1 | 0 | 0.4257 | 0.3582 | 0.5060 |
| factor(X23.CS)=1 | 0.87 | 73 | 1 | 0 | 0.4199 | 0.3525 | 0.5001 |
| factor(X23.CS)=1 | 0.88 | 72 | 1 | 0 | 0.4141 | 0.3469 | 0.4942 |
| factor(X23.CS)=1 | 0.9 | 71 | 1 | 0 | 0.4082 | 0.3413 | 0.4883 |
| factor(X23.CS)=1 | 0.91 | 70 | 1 | 0 | 0.4024 | 0.3357 | 0.4824 |
| factor(X23.CS)=1 | 0.92 | 69 | 1 | 0 | 0.3966 | 0.3300 | 0.4765 |
| factor(X23.CS)=1 | 0.95 | 68 | 1 | 0 | 0.3907 | 0.3245 | 0.4706 |
| factor(X23.CS)=1 | 0.97 | 67 | 1 | 0 | 0.3849 | 0.3189 | 0.4646 |
| factor(X23.CS)=1 | 0.98 | 66 | 1 | 0 | 0.3791 | 0.3133 | 0.4587 |
| factor(X23.CS)=1 | 0.99 | 65 | 2 | 0 | 0.3674 | 0.3022 | 0.4467 |
| factor(X23.CS)=1 | 1.02 | 63 | 1 | 0 | 0.3616 | 0.2966 | 0.4407 |
| factor(X23.CS)=1 | 1.06 | 62 | 1 | 0 | 0.3557 | 0.2911 | 0.4347 |
| factor(X23.CS)=1 | 1.08 | 61 | 2 | 0 | 0.3441 | 0.2801 | 0.4227 |
| factor(X23.CS)=1 | 1.17 | 59 | 1 | 0 | 0.3382 | 0.2746 | 0.4166 |
| factor(X23.CS)=1 | 1.2 | 58 | 2 | 0 | 0.3266 | 0.2637 | 0.4045 |
| factor(X23.CS)=1 | 1.25 | 56 | 2 | 0 | 0.3149 | 0.2528 | 0.3924 |
| factor(X23.CS)=1 | 1.3 | 54 | 1 | 0 | 0.3091 | 0.2473 | 0.3863 |
| factor(X23.CS)=1 | 1.31 | 53 | 2 | 0 | 0.2974 | 0.2365 | 0.3740 |
| factor(X23.CS)=1 | 1.35 | 51 | 1 | 0 | 0.2916 | 0.2311 | 0.3679 |
| factor(X23.CS)=1 | 1.38 | 50 | 2 | 0 | 0.2799 | 0.2204 | 0.3556 |
| factor(X23.CS)=1 | 1.42 | 48 | 1 | 0 | 0.2741 | 0.2150 | 0.3494 |
| factor(X23.CS)=1 | 1.46 | 47 | 1 | 0 | 0.2683 | 0.2097 | 0.3432 |
| factor(X23.CS)=1 | 1.5 | 46 | 1 | 0 | 0.2624 | 0.2043 | 0.3370 |
| factor(X23.CS)=1 | 1.58 | 45 | 1 | 0 | 0.2566 | 0.1990 | 0.3308 |
| factor(X23.CS)=1 | 1.72 | 44 | 1 | 0 | 0.2508 | 0.1937 | 0.3246 |
| factor(X23.CS)=1 | 1.98 | 43 | 4 | 0 | 0.2274 | 0.1727 | 0.2995 |
| factor(X23.CS)=1 | 2.26 | 39 | 1 | 0 | 0.2216 | 0.1675 | 0.2932 |
| factor(X23.CS)=1 | 2.53 | 38 | 1 | 0 | 0.2158 | 0.1623 | 0.2868 |
| factor(X23.CS)=1 | 2.71 | 37 | 0 | 1 | 0.2158 | 0.1623 | 0.2868 |
| factor(X23.CS)=1 | 2.72 | 36 | 3 | 0 | 0.1978 | 0.1464 | 0.2673 |
| factor(X23.CS)=1 | 2.83 | 33 | 1 | 0 | 0.1918 | 0.1411 | 0.2608 |
| factor(X23.CS)=1 | 3.05 | 32 | 0 | 1 | 0.1918 | 0.1411 | 0.2608 |
| factor(X23.CS)=1 | 3.18 | 31 | 0 | 1 | 0.1918 | 0.1411 | 0.2608 |
| factor(X23.CS)=1 | 3.39 | 30 | 2 | 0 | 0.1790 | 0.1298 | 0.2470 |
| factor(X23.CS)=1 | 3.53 | 28 | 0 | 1 | 0.1790 | 0.1298 | 0.2470 |
| factor(X23.CS)=1 | 3.68 | 27 | 0 | 1 | 0.1790 | 0.1298 | 0.2470 |
| factor(X23.CS)=1 | 3.88 | 26 | 0 | 1 | 0.1790 | 0.1298 | 0.2470 |
| factor(X23.CS)=1 | 4.04 | 25 | 0 | 1 | 0.1790 | 0.1298 | 0.2470 |
| factor(X23.CS)=1 | 4.22 | 24 | 0 | 1 | 0.1790 | 0.1298 | 0.2470 |
| factor(X23.CS)=1 | 4.44 | 23 | 0 | 1 | 0.1790 | 0.1298 | 0.2470 |
| factor(X23.CS)=1 | 4.53 | 22 | 0 | 2 | 0.1790 | 0.1298 | 0.2470 |
| factor(X23.CS)=1 | 4.55 | 20 | 0 | 1 | 0.1790 | 0.1298 | 0.2470 |
| factor(X23.CS)=1 | 4.6 | 19 | 0 | 1 | 0.1790 | 0.1298 | 0.2470 |
| factor(X23.CS)=1 | 4.61 | 18 | 0 | 1 | 0.1790 | 0.1298 | 0.2470 |
| factor(X23.CS)=1 | 4.65 | 17 | 0 | 1 | 0.1790 | 0.1298 | 0.2470 |
| factor(X23.CS)=1 | 4.7 | 16 | 0 | 1 | 0.1790 | 0.1298 | 0.2470 |
| factor(X23.CS)=1 | 4.72 | 15 | 1 | 0 | 0.1671 | 0.1179 | 0.2369 |
| factor(X23.CS)=1 | 4.81 | 14 | 0 | 1 | 0.1671 | 0.1179 | 0.2369 |
| factor(X23.CS)=1 | 4.88 | 13 | 0 | 1 | 0.1671 | 0.1179 | 0.2369 |
| factor(X23.CS)=1 | 5 | 12 | 0 | 12 | 0.1671 | 0.1179 | 0.2369 |

|  |  |  |  |  |  |  |  |  |  |
| --- | --- | --- | --- | --- | --- | --- | --- | --- | --- |
| X23.CS | records | n.max | n.start | events | \*rmean | \*se(rmean) | median | 0.95LCL | 0.95UCL |
| factor(X23.CS)=0 | 399 | 399 | 399 | 228 | 2.689 | 0.103 | 1.6 | 1.39 | 2.22 |
| factor(X23.CS)=1 | 175 | 175 | 175 | 143 | 1.526 | 0.132 | 0.76 | 0.68 | 0.88 |

Landmark analysis for time segment: EFS(years) > 5
Log rank test: implements the G-rho family of Harrington and Fleming (1982), with weights on each death of S(t)^rho, where S is the Kaplan-Meier estimate of survival. With rho = 0 this is the log-rank or Mantel-Haenszel test.

|  |  |  |  |
| --- | --- | --- | --- |
|  | N | Observed | Expected |
| factor(X23.CS)=0 | 89 | 1 | 0.8947 |
| factor(X23.CS)=1 | 12 | 0 | 0.1053 |

Chisq=
0.1176
on
1
degree of freedom, p=
0.7316
Survival table

|  |  |  |  |  |  |  |  |
| --- | --- | --- | --- | --- | --- | --- | --- |
|  | EFS(years) | N.Risk | N.Event | N.Censor | Survival | 95%CI Low | 95%CI Upp |
| factor(X23.CS)=0 | 5.02 | 89 | 0 | 1 | 1.0000 | 1.0000 | 1.0000 |
| factor(X23.CS)=0 | 5.04 | 88 | 0 | 1 | 1.0000 | 1.0000 | 1.0000 |
| factor(X23.CS)=0 | 5.17 | 87 | 0 | 1 | 1.0000 | 1.0000 | 1.0000 |
| factor(X23.CS)=0 | 5.18 | 86 | 0 | 1 | 1.0000 | 1.0000 | 1.0000 |
| factor(X23.CS)=0 | 5.2 | 85 | 0 | 1 | 1.0000 | 1.0000 | 1.0000 |
| factor(X23.CS)=0 | 5.27 | 84 | 0 | 2 | 1.0000 | 1.0000 | 1.0000 |
| factor(X23.CS)=0 | 5.3 | 82 | 0 | 1 | 1.0000 | 1.0000 | 1.0000 |
| factor(X23.CS)=0 | 5.33 | 81 | 0 | 1 | 1.0000 | 1.0000 | 1.0000 |
| factor(X23.CS)=0 | 5.35 | 80 | 0 | 1 | 1.0000 | 1.0000 | 1.0000 |
| factor(X23.CS)=0 | 5.37 | 79 | 0 | 2 | 1.0000 | 1.0000 | 1.0000 |
| factor(X23.CS)=0 | 5.38 | 77 | 0 | 1 | 1.0000 | 1.0000 | 1.0000 |
| factor(X23.CS)=0 | 5.41 | 76 | 0 | 1 | 1.0000 | 1.0000 | 1.0000 |
| factor(X23.CS)=0 | 5.44 | 75 | 0 | 2 | 1.0000 | 1.0000 | 1.0000 |
| factor(X23.CS)=0 | 5.46 | 73 | 0 | 1 | 1.0000 | 1.0000 | 1.0000 |
| factor(X23.CS)=0 | 5.52 | 72 | 0 | 1 | 1.0000 | 1.0000 | 1.0000 |
| factor(X23.CS)=0 | 5.54 | 71 | 0 | 2 | 1.0000 | 1.0000 | 1.0000 |
| factor(X23.CS)=0 | 5.57 | 69 | 0 | 1 | 1.0000 | 1.0000 | 1.0000 |
| factor(X23.CS)=0 | 5.58 | 68 | 1 | 0 | 0.9853 | 0.9571 | 1.0000 |
| factor(X23.CS)=0 | 5.6 | 67 | 0 | 1 | 0.9853 | 0.9571 | 1.0000 |
| factor(X23.CS)=0 | 5.61 | 66 | 0 | 1 | 0.9853 | 0.9571 | 1.0000 |
| factor(X23.CS)=0 | 5.63 | 65 | 0 | 1 | 0.9853 | 0.9571 | 1.0000 |
| factor(X23.CS)=0 | 5.67 | 64 | 0 | 1 | 0.9853 | 0.9571 | 1.0000 |
| factor(X23.CS)=0 | 5.7 | 63 | 0 | 1 | 0.9853 | 0.9571 | 1.0000 |
| factor(X23.CS)=0 | 5.75 | 62 | 0 | 1 | 0.9853 | 0.9571 | 1.0000 |
| factor(X23.CS)=0 | 5.79 | 61 | 0 | 1 | 0.9853 | 0.9571 | 1.0000 |
| factor(X23.CS)=0 | 5.81 | 60 | 0 | 2 | 0.9853 | 0.9571 | 1.0000 |
| factor(X23.CS)=0 | 5.84 | 58 | 0 | 1 | 0.9853 | 0.9571 | 1.0000 |
| factor(X23.CS)=0 | 5.92 | 57 | 0 | 1 | 0.9853 | 0.9571 | 1.0000 |
| factor(X23.CS)=0 | 5.95 | 56 | 0 | 1 | 0.9853 | 0.9571 | 1.0000 |
| factor(X23.CS)=0 | 6 | 55 | 0 | 1 | 0.9853 | 0.9571 | 1.0000 |
| factor(X23.CS)=0 | 6.04 | 54 | 0 | 1 | 0.9853 | 0.9571 | 1.0000 |
| factor(X23.CS)=0 | 6.12 | 53 | 0 | 1 | 0.9853 | 0.9571 | 1.0000 |
| factor(X23.CS)=0 | 6.16 | 52 | 0 | 1 | 0.9853 | 0.9571 | 1.0000 |
| factor(X23.CS)=0 | 6.17 | 51 | 0 | 1 | 0.9853 | 0.9571 | 1.0000 |
| factor(X23.CS)=0 | 6.35 | 50 | 0 | 1 | 0.9853 | 0.9571 | 1.0000 |
| factor(X23.CS)=0 | 6.37 | 49 | 0 | 1 | 0.9853 | 0.9571 | 1.0000 |
| factor(X23.CS)=0 | 6.4 | 48 | 0 | 1 | 0.9853 | 0.9571 | 1.0000 |
| factor(X23.CS)=0 | 6.45 | 47 | 0 | 1 | 0.9853 | 0.9571 | 1.0000 |
| factor(X23.CS)=0 | 6.46 | 46 | 0 | 1 | 0.9853 | 0.9571 | 1.0000 |
| factor(X23.CS)=0 | 6.48 | 45 | 0 | 1 | 0.9853 | 0.9571 | 1.0000 |
| factor(X23.CS)=0 | 6.49 | 44 | 0 | 1 | 0.9853 | 0.9571 | 1.0000 |
| factor(X23.CS)=0 | 6.5 | 43 | 0 | 2 | 0.9853 | 0.9571 | 1.0000 |
| factor(X23.CS)=0 | 6.51 | 41 | 0 | 2 | 0.9853 | 0.9571 | 1.0000 |
| factor(X23.CS)=0 | 6.52 | 39 | 0 | 1 | 0.9853 | 0.9571 | 1.0000 |
| factor(X23.CS)=0 | 6.53 | 38 | 0 | 1 | 0.9853 | 0.9571 | 1.0000 |
| factor(X23.CS)=0 | 6.57 | 37 | 0 | 1 | 0.9853 | 0.9571 | 1.0000 |
| factor(X23.CS)=0 | 6.58 | 36 | 0 | 2 | 0.9853 | 0.9571 | 1.0000 |
| factor(X23.CS)=0 | 6.63 | 34 | 0 | 1 | 0.9853 | 0.9571 | 1.0000 |
| factor(X23.CS)=0 | 6.64 | 33 | 0 | 1 | 0.9853 | 0.9571 | 1.0000 |
| factor(X23.CS)=0 | 6.81 | 32 | 0 | 1 | 0.9853 | 0.9571 | 1.0000 |
| factor(X23.CS)=0 | 6.96 | 31 | 0 | 1 | 0.9853 | 0.9571 | 1.0000 |
| factor(X23.CS)=0 | 7 | 30 | 0 | 1 | 0.9853 | 0.9571 | 1.0000 |
| factor(X23.CS)=0 | 7.08 | 29 | 0 | 1 | 0.9853 | 0.9571 | 1.0000 |
| factor(X23.CS)=0 | 7.21 | 28 | 0 | 1 | 0.9853 | 0.9571 | 1.0000 |
| factor(X23.CS)=0 | 7.26 | 27 | 0 | 1 | 0.9853 | 0.9571 | 1.0000 |
| factor(X23.CS)=0 | 7.38 | 26 | 0 | 1 | 0.9853 | 0.9571 | 1.0000 |
| factor(X23.CS)=0 | 7.41 | 25 | 0 | 1 | 0.9853 | 0.9571 | 1.0000 |
| factor(X23.CS)=0 | 7.45 | 24 | 0 | 1 | 0.9853 | 0.9571 | 1.0000 |
| factor(X23.CS)=0 | 7.48 | 23 | 0 | 1 | 0.9853 | 0.9571 | 1.0000 |
| factor(X23.CS)=0 | 7.54 | 22 | 0 | 1 | 0.9853 | 0.9571 | 1.0000 |
| factor(X23.CS)=0 | 7.55 | 21 | 0 | 1 | 0.9853 | 0.9571 | 1.0000 |
| factor(X23.CS)=0 | 7.56 | 20 | 0 | 2 | 0.9853 | 0.9571 | 1.0000 |
| factor(X23.CS)=0 | 7.62 | 18 | 0 | 1 | 0.9853 | 0.9571 | 1.0000 |
| factor(X23.CS)=0 | 7.68 | 17 | 0 | 1 | 0.9853 | 0.9571 | 1.0000 |
| factor(X23.CS)=0 | 7.74 | 16 | 0 | 3 | 0.9853 | 0.9571 | 1.0000 |
| factor(X23.CS)=0 | 7.94 | 13 | 0 | 2 | 0.9853 | 0.9571 | 1.0000 |
| factor(X23.CS)=0 | 7.98 | 11 | 0 | 1 | 0.9853 | 0.9571 | 1.0000 |
| factor(X23.CS)=0 | 8.01 | 10 | 0 | 1 | 0.9853 | 0.9571 | 1.0000 |
| factor(X23.CS)=0 | 8.17 | 9 | 0 | 1 | 0.9853 | 0.9571 | 1.0000 |
| factor(X23.CS)=0 | 8.21 | 8 | 0 | 1 | 0.9853 | 0.9571 | 1.0000 |
| factor(X23.CS)=0 | 8.27 | 7 | 0 | 1 | 0.9853 | 0.9571 | 1.0000 |
| factor(X23.CS)=0 | 8.49 | 6 | 0 | 2 | 0.9853 | 0.9571 | 1.0000 |
| factor(X23.CS)=0 | 8.72 | 4 | 0 | 1 | 0.9853 | 0.9571 | 1.0000 |
| factor(X23.CS)=0 | 8.84 | 3 | 0 | 1 | 0.9853 | 0.9571 | 1.0000 |
| factor(X23.CS)=0 | 9.26 | 2 | 0 | 1 | 0.9853 | 0.9571 | 1.0000 |
| factor(X23.CS)=0 | 9.95 | 1 | 0 | 1 | 0.9853 | 0.9571 | 1.0000 |
| factor(X23.CS)=1 | 5.2 | 12 | 0 | 1 | 1.0000 | 1.0000 | 1.0000 |
| factor(X23.CS)=1 | 5.33 | 11 | 0 | 1 | 1.0000 | 1.0000 | 1.0000 |
| factor(X23.CS)=1 | 5.36 | 10 | 0 | 1 | 1.0000 | 1.0000 | 1.0000 |
| factor(X23.CS)=1 | 5.38 | 9 | 0 | 1 | 1.0000 | 1.0000 | 1.0000 |
| factor(X23.CS)=1 | 6.4 | 8 | 0 | 1 | 1.0000 | 1.0000 | 1.0000 |
| factor(X23.CS)=1 | 6.79 | 7 | 0 | 1 | 1.0000 | 1.0000 | 1.0000 |
| factor(X23.CS)=1 | 7.12 | 6 | 0 | 1 | 1.0000 | 1.0000 | 1.0000 |
| factor(X23.CS)=1 | 7.36 | 5 | 0 | 2 | 1.0000 | 1.0000 | 1.0000 |
| factor(X23.CS)=1 | 7.46 | 3 | 0 | 1 | 1.0000 | 1.0000 | 1.0000 |
| factor(X23.CS)=1 | 8.1 | 2 | 0 | 1 | 1.0000 | 1.0000 | 1.0000 |
| factor(X23.CS)=1 | 8.36 | 1 | 0 | 1 | 1.0000 | 1.0000 | 1.0000 |

|  |  |  |  |  |  |  |  |  |  |
| --- | --- | --- | --- | --- | --- | --- | --- | --- | --- |
| X23.CS | records | n.max | n.start | events | \*rmean | \*se(rmean) | median | 0.95LCL | 0.95UCL |
| factor(X23.CS)=0 | 89 | 89 | 89 | 1 | 9.102 | 0.052 | NA | NA | NA |
| factor(X23.CS)=1 | 12 | 12 | 12 | 0 | 9.155 | 0 | NA | NA | NA |

Use subset of data: (!is.na(X5) & (X5==2))
Created by EmpowerStats (www.empowerstats.com) and R on 2025-10-07
