# Supplementary material for: Optimal minimal residual disease threshold in pediatric acute myeloid leukemia: A retrospective cohort study based on the TARGET database
Source: PLoS Med. 2026 May 8;23(5):e1005088. doi: 10.1371/journal.pmed.1005088 (PMC13155632; doi:10.1371/journal.pmed.1005088)
Supplement: S1 Code — (ZIP) [file pmed.1005088.s002.zip › S2 code/PROJ8_5_tbl/PROJ8_5_tbl.htm]

## Kaplan Meier Survival Curve

Outcome: First Event
Time: EFS(years)
Landmark analysis for time segment: EFS(years) <= 5
Log rank test: implements the G-rho family of Harrington and Fleming (1982), with weights on each death of S(t)^rho, where S is the Kaplan-Meier estimate of survival. With rho = 0 this is the log-rank or Mantel-Haenszel test.

|  |  |  |  |
| --- | --- | --- | --- |
|  | N | Observed | Expected |
| factor(X25.CS)=0 | 1068 | 490 | 567.7896 |
| factor(X25.CS)=1 | 137 | 113 | 35.2104 |

Chisq=
184.2423
on
1
degree of freedom, p=
<0.0001
Survival table

|  |  |  |  |  |  |  |  |
| --- | --- | --- | --- | --- | --- | --- | --- |
|  | EFS(years) | N.Risk | N.Event | N.Censor | Survival | 95%CI Low | 95%CI Upp |
| factor(X25.CS)=0 | 0.16 | 1068 | 1 | 0 | 0.9991 | 0.9972 | 1.0000 |
| factor(X25.CS)=0 | 0.17 | 1067 | 2 | 0 | 0.9972 | 0.9940 | 1.0000 |
| factor(X25.CS)=0 | 0.18 | 1065 | 4 | 0 | 0.9934 | 0.9886 | 0.9983 |
| factor(X25.CS)=0 | 0.19 | 1061 | 2 | 1 | 0.9916 | 0.9861 | 0.9971 |
| factor(X25.CS)=0 | 0.2 | 1058 | 5 | 0 | 0.9869 | 0.9801 | 0.9937 |
| factor(X25.CS)=0 | 0.21 | 1053 | 5 | 0 | 0.9822 | 0.9743 | 0.9902 |
| factor(X25.CS)=0 | 0.22 | 1048 | 2 | 0 | 0.9803 | 0.9720 | 0.9887 |
| factor(X25.CS)=0 | 0.23 | 1046 | 2 | 1 | 0.9785 | 0.9698 | 0.9872 |
| factor(X25.CS)=0 | 0.24 | 1043 | 1 | 0 | 0.9775 | 0.9687 | 0.9864 |
| factor(X25.CS)=0 | 0.25 | 1042 | 1 | 0 | 0.9766 | 0.9675 | 0.9857 |
| factor(X25.CS)=0 | 0.26 | 1041 | 4 | 0 | 0.9728 | 0.9631 | 0.9826 |
| factor(X25.CS)=0 | 0.27 | 1037 | 3 | 1 | 0.9700 | 0.9598 | 0.9803 |
| factor(X25.CS)=0 | 0.28 | 1033 | 4 | 0 | 0.9663 | 0.9555 | 0.9772 |
| factor(X25.CS)=0 | 0.29 | 1029 | 3 | 0 | 0.9634 | 0.9522 | 0.9748 |
| factor(X25.CS)=0 | 0.31 | 1026 | 1 | 0 | 0.9625 | 0.9512 | 0.9740 |
| factor(X25.CS)=0 | 0.32 | 1025 | 3 | 0 | 0.9597 | 0.9479 | 0.9716 |
| factor(X25.CS)=0 | 0.33 | 1022 | 3 | 1 | 0.9569 | 0.9447 | 0.9691 |
| factor(X25.CS)=0 | 0.35 | 1018 | 1 | 0 | 0.9559 | 0.9437 | 0.9683 |
| factor(X25.CS)=0 | 0.36 | 1017 | 1 | 0 | 0.9550 | 0.9426 | 0.9675 |
| factor(X25.CS)=0 | 0.37 | 1016 | 3 | 0 | 0.9522 | 0.9394 | 0.9651 |
| factor(X25.CS)=0 | 0.38 | 1013 | 2 | 0 | 0.9503 | 0.9373 | 0.9634 |
| factor(X25.CS)=0 | 0.4 | 1011 | 4 | 0 | 0.9465 | 0.9331 | 0.9601 |
| factor(X25.CS)=0 | 0.41 | 1007 | 4 | 0 | 0.9428 | 0.9289 | 0.9568 |
| factor(X25.CS)=0 | 0.42 | 1003 | 3 | 0 | 0.9399 | 0.9258 | 0.9543 |
| factor(X25.CS)=0 | 0.43 | 1000 | 1 | 0 | 0.9390 | 0.9247 | 0.9535 |
| factor(X25.CS)=0 | 0.44 | 999 | 5 | 0 | 0.9343 | 0.9195 | 0.9493 |
| factor(X25.CS)=0 | 0.45 | 994 | 3 | 0 | 0.9315 | 0.9164 | 0.9468 |
| factor(X25.CS)=0 | 0.46 | 991 | 2 | 0 | 0.9296 | 0.9144 | 0.9451 |
| factor(X25.CS)=0 | 0.47 | 989 | 3 | 0 | 0.9268 | 0.9113 | 0.9426 |
| factor(X25.CS)=0 | 0.48 | 986 | 2 | 0 | 0.9249 | 0.9092 | 0.9409 |
| factor(X25.CS)=0 | 0.5 | 984 | 3 | 0 | 0.9221 | 0.9061 | 0.9383 |
| factor(X25.CS)=0 | 0.51 | 981 | 1 | 0 | 0.9211 | 0.9051 | 0.9375 |
| factor(X25.CS)=0 | 0.52 | 980 | 5 | 0 | 0.9164 | 0.9000 | 0.9332 |
| factor(X25.CS)=0 | 0.53 | 975 | 7 | 0 | 0.9099 | 0.8928 | 0.9272 |
| factor(X25.CS)=0 | 0.54 | 968 | 3 | 0 | 0.9070 | 0.8898 | 0.9247 |
| factor(X25.CS)=0 | 0.55 | 965 | 5 | 0 | 0.9023 | 0.8847 | 0.9204 |
| factor(X25.CS)=0 | 0.56 | 960 | 7 | 0 | 0.8958 | 0.8776 | 0.9143 |
| factor(X25.CS)=0 | 0.57 | 953 | 5 | 0 | 0.8911 | 0.8725 | 0.9100 |
| factor(X25.CS)=0 | 0.58 | 948 | 5 | 0 | 0.8864 | 0.8675 | 0.9056 |
| factor(X25.CS)=0 | 0.59 | 943 | 4 | 0 | 0.8826 | 0.8635 | 0.9022 |
| factor(X25.CS)=0 | 0.6 | 939 | 5 | 0 | 0.8779 | 0.8585 | 0.8978 |
| factor(X25.CS)=0 | 0.61 | 934 | 1 | 0 | 0.8770 | 0.8575 | 0.8969 |
| factor(X25.CS)=0 | 0.62 | 933 | 2 | 0 | 0.8751 | 0.8555 | 0.8952 |
| factor(X25.CS)=0 | 0.63 | 931 | 1 | 0 | 0.8741 | 0.8544 | 0.8943 |
| factor(X25.CS)=0 | 0.64 | 930 | 2 | 0 | 0.8723 | 0.8524 | 0.8926 |
| factor(X25.CS)=0 | 0.65 | 928 | 4 | 0 | 0.8685 | 0.8484 | 0.8890 |
| factor(X25.CS)=0 | 0.66 | 924 | 2 | 0 | 0.8666 | 0.8464 | 0.8873 |
| factor(X25.CS)=0 | 0.67 | 922 | 3 | 0 | 0.8638 | 0.8434 | 0.8847 |
| factor(X25.CS)=0 | 0.68 | 919 | 8 | 0 | 0.8563 | 0.8355 | 0.8776 |
| factor(X25.CS)=0 | 0.69 | 911 | 5 | 0 | 0.8516 | 0.8305 | 0.8732 |
| factor(X25.CS)=0 | 0.7 | 906 | 5 | 0 | 0.8469 | 0.8255 | 0.8688 |
| factor(X25.CS)=0 | 0.71 | 901 | 4 | 0 | 0.8431 | 0.8216 | 0.8653 |
| factor(X25.CS)=0 | 0.72 | 897 | 7 | 0 | 0.8365 | 0.8146 | 0.8591 |
| factor(X25.CS)=0 | 0.73 | 890 | 4 | 0 | 0.8328 | 0.8107 | 0.8555 |
| factor(X25.CS)=0 | 0.74 | 886 | 4 | 0 | 0.8290 | 0.8067 | 0.8520 |
| factor(X25.CS)=0 | 0.75 | 882 | 4 | 0 | 0.8253 | 0.8028 | 0.8484 |
| factor(X25.CS)=0 | 0.76 | 878 | 10 | 0 | 0.8159 | 0.7929 | 0.8395 |
| factor(X25.CS)=0 | 0.77 | 868 | 1 | 0 | 0.8149 | 0.7919 | 0.8386 |
| factor(X25.CS)=0 | 0.78 | 867 | 4 | 1 | 0.8112 | 0.7880 | 0.8350 |
| factor(X25.CS)=0 | 0.79 | 862 | 8 | 0 | 0.8036 | 0.7801 | 0.8279 |
| factor(X25.CS)=0 | 0.8 | 854 | 4 | 0 | 0.7999 | 0.7762 | 0.8243 |
| factor(X25.CS)=0 | 0.81 | 850 | 8 | 0 | 0.7924 | 0.7684 | 0.8171 |
| factor(X25.CS)=0 | 0.82 | 842 | 9 | 0 | 0.7839 | 0.7595 | 0.8090 |
| factor(X25.CS)=0 | 0.83 | 833 | 4 | 0 | 0.7801 | 0.7556 | 0.8054 |
| factor(X25.CS)=0 | 0.84 | 829 | 9 | 0 | 0.7716 | 0.7468 | 0.7973 |
| factor(X25.CS)=0 | 0.85 | 820 | 2 | 0 | 0.7698 | 0.7449 | 0.7955 |
| factor(X25.CS)=0 | 0.86 | 818 | 3 | 0 | 0.7669 | 0.7420 | 0.7928 |
| factor(X25.CS)=0 | 0.87 | 815 | 2 | 0 | 0.7651 | 0.7400 | 0.7910 |
| factor(X25.CS)=0 | 0.88 | 813 | 8 | 0 | 0.7575 | 0.7322 | 0.7837 |
| factor(X25.CS)=0 | 0.89 | 805 | 2 | 0 | 0.7557 | 0.7303 | 0.7819 |
| factor(X25.CS)=0 | 0.9 | 803 | 3 | 0 | 0.7528 | 0.7273 | 0.7792 |
| factor(X25.CS)=0 | 0.91 | 800 | 5 | 0 | 0.7481 | 0.7225 | 0.7747 |
| factor(X25.CS)=0 | 0.92 | 795 | 5 | 0 | 0.7434 | 0.7176 | 0.7701 |
| factor(X25.CS)=0 | 0.93 | 790 | 9 | 0 | 0.7349 | 0.7089 | 0.7620 |
| factor(X25.CS)=0 | 0.94 | 781 | 2 | 0 | 0.7331 | 0.7070 | 0.7601 |
| factor(X25.CS)=0 | 0.95 | 779 | 1 | 0 | 0.7321 | 0.7060 | 0.7592 |
| factor(X25.CS)=0 | 0.96 | 778 | 2 | 1 | 0.7302 | 0.7041 | 0.7574 |
| factor(X25.CS)=0 | 0.97 | 775 | 3 | 0 | 0.7274 | 0.7011 | 0.7547 |
| factor(X25.CS)=0 | 0.98 | 772 | 1 | 0 | 0.7265 | 0.7002 | 0.7538 |
| factor(X25.CS)=0 | 0.99 | 771 | 4 | 1 | 0.7227 | 0.6963 | 0.7501 |
| factor(X25.CS)=0 | 1 | 766 | 4 | 0 | 0.7189 | 0.6924 | 0.7465 |
| factor(X25.CS)=0 | 1.01 | 762 | 3 | 0 | 0.7161 | 0.6895 | 0.7437 |
| factor(X25.CS)=0 | 1.02 | 759 | 3 | 1 | 0.7133 | 0.6866 | 0.7410 |
| factor(X25.CS)=0 | 1.03 | 755 | 3 | 0 | 0.7104 | 0.6837 | 0.7382 |
| factor(X25.CS)=0 | 1.05 | 752 | 6 | 0 | 0.7048 | 0.6779 | 0.7327 |
| factor(X25.CS)=0 | 1.06 | 746 | 3 | 1 | 0.7019 | 0.6750 | 0.7300 |
| factor(X25.CS)=0 | 1.07 | 742 | 4 | 0 | 0.6981 | 0.6711 | 0.7263 |
| factor(X25.CS)=0 | 1.08 | 738 | 6 | 0 | 0.6925 | 0.6653 | 0.7208 |
| factor(X25.CS)=0 | 1.09 | 732 | 2 | 0 | 0.6906 | 0.6633 | 0.7189 |
| factor(X25.CS)=0 | 1.1 | 730 | 2 | 0 | 0.6887 | 0.6614 | 0.7171 |
| factor(X25.CS)=0 | 1.11 | 728 | 1 | 2 | 0.6877 | 0.6604 | 0.7162 |
| factor(X25.CS)=0 | 1.12 | 725 | 4 | 1 | 0.6839 | 0.6566 | 0.7125 |
| factor(X25.CS)=0 | 1.13 | 720 | 4 | 0 | 0.6801 | 0.6527 | 0.7088 |
| factor(X25.CS)=0 | 1.14 | 716 | 2 | 1 | 0.6782 | 0.6507 | 0.7069 |
| factor(X25.CS)=0 | 1.15 | 713 | 1 | 1 | 0.6773 | 0.6498 | 0.7060 |
| factor(X25.CS)=0 | 1.16 | 711 | 2 | 0 | 0.6754 | 0.6478 | 0.7041 |
| factor(X25.CS)=0 | 1.17 | 709 | 2 | 0 | 0.6735 | 0.6459 | 0.7023 |
| factor(X25.CS)=0 | 1.18 | 707 | 2 | 0 | 0.6716 | 0.6439 | 0.7004 |
| factor(X25.CS)=0 | 1.19 | 705 | 2 | 0 | 0.6697 | 0.6420 | 0.6986 |
| factor(X25.CS)=0 | 1.2 | 703 | 5 | 0 | 0.6649 | 0.6371 | 0.6939 |
| factor(X25.CS)=0 | 1.21 | 698 | 1 | 1 | 0.6640 | 0.6361 | 0.6930 |
| factor(X25.CS)=0 | 1.24 | 696 | 1 | 0 | 0.6630 | 0.6352 | 0.6921 |
| factor(X25.CS)=0 | 1.25 | 695 | 2 | 1 | 0.6611 | 0.6332 | 0.6902 |
| factor(X25.CS)=0 | 1.26 | 692 | 1 | 0 | 0.6601 | 0.6323 | 0.6893 |
| factor(X25.CS)=0 | 1.27 | 691 | 4 | 0 | 0.6563 | 0.6284 | 0.6855 |
| factor(X25.CS)=0 | 1.28 | 687 | 2 | 1 | 0.6544 | 0.6264 | 0.6837 |
| factor(X25.CS)=0 | 1.3 | 684 | 4 | 0 | 0.6506 | 0.6225 | 0.6799 |
| factor(X25.CS)=0 | 1.31 | 680 | 2 | 0 | 0.6487 | 0.6206 | 0.6780 |
| factor(X25.CS)=0 | 1.32 | 678 | 1 | 0 | 0.6477 | 0.6196 | 0.6771 |
| factor(X25.CS)=0 | 1.34 | 677 | 3 | 1 | 0.6448 | 0.6167 | 0.6743 |
| factor(X25.CS)=0 | 1.35 | 673 | 3 | 0 | 0.6420 | 0.6137 | 0.6715 |
| factor(X25.CS)=0 | 1.36 | 670 | 3 | 0 | 0.6391 | 0.6108 | 0.6687 |
| factor(X25.CS)=0 | 1.37 | 667 | 2 | 1 | 0.6372 | 0.6089 | 0.6668 |
| factor(X25.CS)=0 | 1.38 | 664 | 3 | 0 | 0.6343 | 0.6060 | 0.6640 |
| factor(X25.CS)=0 | 1.39 | 661 | 4 | 1 | 0.6305 | 0.6021 | 0.6602 |
| factor(X25.CS)=0 | 1.4 | 656 | 1 | 0 | 0.6295 | 0.6011 | 0.6593 |
| factor(X25.CS)=0 | 1.41 | 655 | 1 | 0 | 0.6285 | 0.6001 | 0.6583 |
| factor(X25.CS)=0 | 1.42 | 654 | 2 | 0 | 0.6266 | 0.5982 | 0.6564 |
| factor(X25.CS)=0 | 1.43 | 652 | 4 | 0 | 0.6228 | 0.5943 | 0.6527 |
| factor(X25.CS)=0 | 1.45 | 648 | 2 | 0 | 0.6209 | 0.5923 | 0.6508 |
| factor(X25.CS)=0 | 1.46 | 646 | 1 | 0 | 0.6199 | 0.5913 | 0.6498 |
| factor(X25.CS)=0 | 1.48 | 645 | 2 | 0 | 0.6180 | 0.5894 | 0.6479 |
| factor(X25.CS)=0 | 1.49 | 643 | 3 | 0 | 0.6151 | 0.5865 | 0.6451 |
| factor(X25.CS)=0 | 1.5 | 640 | 2 | 0 | 0.6132 | 0.5845 | 0.6432 |
| factor(X25.CS)=0 | 1.51 | 638 | 1 | 0 | 0.6122 | 0.5835 | 0.6423 |
| factor(X25.CS)=0 | 1.53 | 637 | 1 | 0 | 0.6112 | 0.5826 | 0.6413 |
| factor(X25.CS)=0 | 1.54 | 636 | 1 | 0 | 0.6103 | 0.5816 | 0.6404 |
| factor(X25.CS)=0 | 1.55 | 635 | 3 | 0 | 0.6074 | 0.5787 | 0.6376 |
| factor(X25.CS)=0 | 1.56 | 632 | 1 | 0 | 0.6064 | 0.5777 | 0.6366 |
| factor(X25.CS)=0 | 1.58 | 631 | 5 | 0 | 0.6016 | 0.5728 | 0.6319 |
| factor(X25.CS)=0 | 1.6 | 626 | 2 | 0 | 0.5997 | 0.5709 | 0.6300 |
| factor(X25.CS)=0 | 1.61 | 624 | 0 | 1 | 0.5997 | 0.5709 | 0.6300 |
| factor(X25.CS)=0 | 1.62 | 623 | 2 | 0 | 0.5978 | 0.5689 | 0.6281 |
| factor(X25.CS)=0 | 1.64 | 621 | 1 | 0 | 0.5968 | 0.5680 | 0.6271 |
| factor(X25.CS)=0 | 1.65 | 620 | 2 | 0 | 0.5949 | 0.5660 | 0.6252 |
| factor(X25.CS)=0 | 1.66 | 618 | 1 | 0 | 0.5939 | 0.5650 | 0.6243 |
| factor(X25.CS)=0 | 1.67 | 617 | 1 | 0 | 0.5930 | 0.5641 | 0.6233 |
| factor(X25.CS)=0 | 1.71 | 616 | 0 | 1 | 0.5930 | 0.5641 | 0.6233 |
| factor(X25.CS)=0 | 1.72 | 615 | 3 | 0 | 0.5901 | 0.5611 | 0.6205 |
| factor(X25.CS)=0 | 1.75 | 612 | 0 | 1 | 0.5901 | 0.5611 | 0.6205 |
| factor(X25.CS)=0 | 1.77 | 611 | 1 | 0 | 0.5891 | 0.5602 | 0.6195 |
| factor(X25.CS)=0 | 1.78 | 610 | 1 | 0 | 0.5881 | 0.5592 | 0.6186 |
| factor(X25.CS)=0 | 1.79 | 609 | 1 | 0 | 0.5872 | 0.5582 | 0.6176 |
| factor(X25.CS)=0 | 1.81 | 608 | 1 | 0 | 0.5862 | 0.5572 | 0.6167 |
| factor(X25.CS)=0 | 1.83 | 607 | 0 | 1 | 0.5862 | 0.5572 | 0.6167 |
| factor(X25.CS)=0 | 1.87 | 606 | 1 | 1 | 0.5852 | 0.5563 | 0.6157 |
| factor(X25.CS)=0 | 1.89 | 604 | 2 | 0 | 0.5833 | 0.5543 | 0.6138 |
| factor(X25.CS)=0 | 1.9 | 602 | 2 | 0 | 0.5814 | 0.5524 | 0.6119 |
| factor(X25.CS)=0 | 1.92 | 600 | 2 | 1 | 0.5794 | 0.5504 | 0.6100 |
| factor(X25.CS)=0 | 1.94 | 597 | 1 | 2 | 0.5785 | 0.5494 | 0.6090 |
| factor(X25.CS)=0 | 1.96 | 594 | 0 | 1 | 0.5785 | 0.5494 | 0.6090 |
| factor(X25.CS)=0 | 1.98 | 593 | 4 | 1 | 0.5746 | 0.5455 | 0.6052 |
| factor(X25.CS)=0 | 1.99 | 588 | 1 | 0 | 0.5736 | 0.5445 | 0.6042 |
| factor(X25.CS)=0 | 2.03 | 587 | 1 | 0 | 0.5726 | 0.5435 | 0.6033 |
| factor(X25.CS)=0 | 2.05 | 586 | 1 | 0 | 0.5716 | 0.5425 | 0.6023 |
| factor(X25.CS)=0 | 2.07 | 585 | 1 | 0 | 0.5707 | 0.5415 | 0.6013 |
| factor(X25.CS)=0 | 2.08 | 584 | 1 | 0 | 0.5697 | 0.5406 | 0.6004 |
| factor(X25.CS)=0 | 2.09 | 583 | 2 | 0 | 0.5677 | 0.5386 | 0.5984 |
| factor(X25.CS)=0 | 2.1 | 581 | 0 | 1 | 0.5677 | 0.5386 | 0.5984 |
| factor(X25.CS)=0 | 2.11 | 580 | 1 | 0 | 0.5667 | 0.5376 | 0.5975 |
| factor(X25.CS)=0 | 2.13 | 579 | 1 | 0 | 0.5658 | 0.5366 | 0.5965 |
| factor(X25.CS)=0 | 2.14 | 578 | 1 | 0 | 0.5648 | 0.5356 | 0.5955 |
| factor(X25.CS)=0 | 2.15 | 577 | 1 | 0 | 0.5638 | 0.5346 | 0.5946 |
| factor(X25.CS)=0 | 2.18 | 576 | 0 | 1 | 0.5638 | 0.5346 | 0.5946 |
| factor(X25.CS)=0 | 2.2 | 575 | 1 | 0 | 0.5628 | 0.5336 | 0.5936 |
| factor(X25.CS)=0 | 2.22 | 574 | 2 | 1 | 0.5609 | 0.5317 | 0.5917 |
| factor(X25.CS)=0 | 2.23 | 571 | 1 | 1 | 0.5599 | 0.5307 | 0.5907 |
| factor(X25.CS)=0 | 2.27 | 569 | 0 | 2 | 0.5599 | 0.5307 | 0.5907 |
| factor(X25.CS)=0 | 2.3 | 567 | 1 | 0 | 0.5589 | 0.5297 | 0.5897 |
| factor(X25.CS)=0 | 2.31 | 566 | 1 | 0 | 0.5579 | 0.5287 | 0.5887 |
| factor(X25.CS)=0 | 2.32 | 565 | 0 | 2 | 0.5579 | 0.5287 | 0.5887 |
| factor(X25.CS)=0 | 2.34 | 563 | 1 | 0 | 0.5569 | 0.5277 | 0.5878 |
| factor(X25.CS)=0 | 2.36 | 562 | 1 | 0 | 0.5559 | 0.5267 | 0.5868 |
| factor(X25.CS)=0 | 2.37 | 561 | 1 | 1 | 0.5549 | 0.5257 | 0.5858 |
| factor(X25.CS)=0 | 2.39 | 559 | 0 | 1 | 0.5549 | 0.5257 | 0.5858 |
| factor(X25.CS)=0 | 2.4 | 558 | 0 | 1 | 0.5549 | 0.5257 | 0.5858 |
| factor(X25.CS)=0 | 2.41 | 557 | 0 | 1 | 0.5549 | 0.5257 | 0.5858 |
| factor(X25.CS)=0 | 2.42 | 556 | 1 | 0 | 0.5539 | 0.5247 | 0.5848 |
| factor(X25.CS)=0 | 2.43 | 555 | 0 | 1 | 0.5539 | 0.5247 | 0.5848 |
| factor(X25.CS)=0 | 2.44 | 554 | 0 | 2 | 0.5539 | 0.5247 | 0.5848 |
| factor(X25.CS)=0 | 2.46 | 552 | 0 | 1 | 0.5539 | 0.5247 | 0.5848 |
| factor(X25.CS)=0 | 2.47 | 551 | 0 | 1 | 0.5539 | 0.5247 | 0.5848 |
| factor(X25.CS)=0 | 2.48 | 550 | 1 | 0 | 0.5529 | 0.5237 | 0.5838 |
| factor(X25.CS)=0 | 2.49 | 549 | 0 | 1 | 0.5529 | 0.5237 | 0.5838 |
| factor(X25.CS)=0 | 2.5 | 548 | 0 | 4 | 0.5529 | 0.5237 | 0.5838 |
| factor(X25.CS)=0 | 2.51 | 544 | 1 | 1 | 0.5519 | 0.5227 | 0.5828 |
| factor(X25.CS)=0 | 2.52 | 542 | 1 | 0 | 0.5509 | 0.5216 | 0.5818 |
| factor(X25.CS)=0 | 2.53 | 541 | 1 | 1 | 0.5499 | 0.5206 | 0.5808 |
| factor(X25.CS)=0 | 2.55 | 539 | 0 | 1 | 0.5499 | 0.5206 | 0.5808 |
| factor(X25.CS)=0 | 2.56 | 538 | 0 | 1 | 0.5499 | 0.5206 | 0.5808 |
| factor(X25.CS)=0 | 2.6 | 537 | 0 | 1 | 0.5499 | 0.5206 | 0.5808 |
| factor(X25.CS)=0 | 2.61 | 536 | 1 | 1 | 0.5488 | 0.5196 | 0.5798 |
| factor(X25.CS)=0 | 2.62 | 534 | 2 | 0 | 0.5468 | 0.5175 | 0.5778 |
| factor(X25.CS)=0 | 2.63 | 532 | 0 | 1 | 0.5468 | 0.5175 | 0.5778 |
| factor(X25.CS)=0 | 2.64 | 531 | 0 | 1 | 0.5468 | 0.5175 | 0.5778 |
| factor(X25.CS)=0 | 2.68 | 530 | 0 | 1 | 0.5468 | 0.5175 | 0.5778 |
| factor(X25.CS)=0 | 2.7 | 529 | 1 | 1 | 0.5458 | 0.5164 | 0.5767 |
| factor(X25.CS)=0 | 2.71 | 527 | 0 | 1 | 0.5458 | 0.5164 | 0.5767 |
| factor(X25.CS)=0 | 2.73 | 526 | 0 | 1 | 0.5458 | 0.5164 | 0.5767 |
| factor(X25.CS)=0 | 2.75 | 525 | 0 | 1 | 0.5458 | 0.5164 | 0.5767 |
| factor(X25.CS)=0 | 2.78 | 524 | 0 | 2 | 0.5458 | 0.5164 | 0.5767 |
| factor(X25.CS)=0 | 2.79 | 522 | 0 | 2 | 0.5458 | 0.5164 | 0.5767 |
| factor(X25.CS)=0 | 2.83 | 520 | 1 | 1 | 0.5447 | 0.5154 | 0.5757 |
| factor(X25.CS)=0 | 2.89 | 518 | 0 | 1 | 0.5447 | 0.5154 | 0.5757 |
| factor(X25.CS)=0 | 2.93 | 517 | 0 | 1 | 0.5447 | 0.5154 | 0.5757 |
| factor(X25.CS)=0 | 2.95 | 516 | 0 | 1 | 0.5447 | 0.5154 | 0.5757 |
| factor(X25.CS)=0 | 2.98 | 515 | 0 | 1 | 0.5447 | 0.5154 | 0.5757 |
| factor(X25.CS)=0 | 2.99 | 514 | 1 | 0 | 0.5437 | 0.5143 | 0.5747 |
| factor(X25.CS)=0 | 3 | 513 | 0 | 1 | 0.5437 | 0.5143 | 0.5747 |
| factor(X25.CS)=0 | 3.01 | 512 | 0 | 1 | 0.5437 | 0.5143 | 0.5747 |
| factor(X25.CS)=0 | 3.02 | 511 | 0 | 1 | 0.5437 | 0.5143 | 0.5747 |
| factor(X25.CS)=0 | 3.03 | 510 | 0 | 1 | 0.5437 | 0.5143 | 0.5747 |
| factor(X25.CS)=0 | 3.04 | 509 | 0 | 2 | 0.5437 | 0.5143 | 0.5747 |
| factor(X25.CS)=0 | 3.05 | 507 | 0 | 2 | 0.5437 | 0.5143 | 0.5747 |
| factor(X25.CS)=0 | 3.07 | 505 | 0 | 1 | 0.5437 | 0.5143 | 0.5747 |
| factor(X25.CS)=0 | 3.11 | 504 | 0 | 1 | 0.5437 | 0.5143 | 0.5747 |
| factor(X25.CS)=0 | 3.14 | 503 | 0 | 1 | 0.5437 | 0.5143 | 0.5747 |
| factor(X25.CS)=0 | 3.15 | 502 | 0 | 1 | 0.5437 | 0.5143 | 0.5747 |
| factor(X25.CS)=0 | 3.16 | 501 | 0 | 2 | 0.5437 | 0.5143 | 0.5747 |
| factor(X25.CS)=0 | 3.17 | 499 | 0 | 1 | 0.5437 | 0.5143 | 0.5747 |
| factor(X25.CS)=0 | 3.18 | 498 | 1 | 3 | 0.5426 | 0.5132 | 0.5736 |
| factor(X25.CS)=0 | 3.19 | 494 | 0 | 1 | 0.5426 | 0.5132 | 0.5736 |
| factor(X25.CS)=0 | 3.2 | 493 | 0 | 1 | 0.5426 | 0.5132 | 0.5736 |
| factor(X25.CS)=0 | 3.22 | 492 | 0 | 1 | 0.5426 | 0.5132 | 0.5736 |
| factor(X25.CS)=0 | 3.23 | 491 | 0 | 1 | 0.5426 | 0.5132 | 0.5736 |
| factor(X25.CS)=0 | 3.24 | 490 | 0 | 1 | 0.5426 | 0.5132 | 0.5736 |
| factor(X25.CS)=0 | 3.25 | 489 | 0 | 2 | 0.5426 | 0.5132 | 0.5736 |
| factor(X25.CS)=0 | 3.28 | 487 | 0 | 3 | 0.5426 | 0.5132 | 0.5736 |
| factor(X25.CS)=0 | 3.29 | 484 | 0 | 1 | 0.5426 | 0.5132 | 0.5736 |
| factor(X25.CS)=0 | 3.3 | 483 | 0 | 2 | 0.5426 | 0.5132 | 0.5736 |
| factor(X25.CS)=0 | 3.32 | 481 | 0 | 1 | 0.5426 | 0.5132 | 0.5736 |
| factor(X25.CS)=0 | 3.33 | 480 | 0 | 3 | 0.5426 | 0.5132 | 0.5736 |
| factor(X25.CS)=0 | 3.34 | 477 | 0 | 2 | 0.5426 | 0.5132 | 0.5736 |
| factor(X25.CS)=0 | 3.35 | 475 | 0 | 3 | 0.5426 | 0.5132 | 0.5736 |
| factor(X25.CS)=0 | 3.36 | 472 | 0 | 2 | 0.5426 | 0.5132 | 0.5736 |
| factor(X25.CS)=0 | 3.38 | 470 | 0 | 3 | 0.5426 | 0.5132 | 0.5736 |
| factor(X25.CS)=0 | 3.39 | 467 | 1 | 2 | 0.5414 | 0.5120 | 0.5724 |
| factor(X25.CS)=0 | 3.4 | 464 | 0 | 1 | 0.5414 | 0.5120 | 0.5724 |
| factor(X25.CS)=0 | 3.41 | 463 | 0 | 4 | 0.5414 | 0.5120 | 0.5724 |
| factor(X25.CS)=0 | 3.43 | 459 | 0 | 1 | 0.5414 | 0.5120 | 0.5724 |
| factor(X25.CS)=0 | 3.44 | 458 | 0 | 4 | 0.5414 | 0.5120 | 0.5724 |
| factor(X25.CS)=0 | 3.45 | 454 | 0 | 2 | 0.5414 | 0.5120 | 0.5724 |
| factor(X25.CS)=0 | 3.46 | 452 | 0 | 1 | 0.5414 | 0.5120 | 0.5724 |
| factor(X25.CS)=0 | 3.48 | 451 | 0 | 2 | 0.5414 | 0.5120 | 0.5724 |
| factor(X25.CS)=0 | 3.5 | 449 | 0 | 3 | 0.5414 | 0.5120 | 0.5724 |
| factor(X25.CS)=0 | 3.52 | 446 | 0 | 1 | 0.5414 | 0.5120 | 0.5724 |
| factor(X25.CS)=0 | 3.53 | 445 | 0 | 1 | 0.5414 | 0.5120 | 0.5724 |
| factor(X25.CS)=0 | 3.54 | 444 | 1 | 1 | 0.5402 | 0.5108 | 0.5713 |
| factor(X25.CS)=0 | 3.56 | 442 | 0 | 4 | 0.5402 | 0.5108 | 0.5713 |
| factor(X25.CS)=0 | 3.58 | 438 | 0 | 1 | 0.5402 | 0.5108 | 0.5713 |
| factor(X25.CS)=0 | 3.62 | 437 | 0 | 1 | 0.5402 | 0.5108 | 0.5713 |
| factor(X25.CS)=0 | 3.63 | 436 | 0 | 1 | 0.5402 | 0.5108 | 0.5713 |
| factor(X25.CS)=0 | 3.65 | 435 | 0 | 2 | 0.5402 | 0.5108 | 0.5713 |
| factor(X25.CS)=0 | 3.66 | 433 | 0 | 1 | 0.5402 | 0.5108 | 0.5713 |
| factor(X25.CS)=0 | 3.74 | 432 | 0 | 1 | 0.5402 | 0.5108 | 0.5713 |
| factor(X25.CS)=0 | 3.79 | 431 | 0 | 1 | 0.5402 | 0.5108 | 0.5713 |
| factor(X25.CS)=0 | 3.81 | 430 | 0 | 2 | 0.5402 | 0.5108 | 0.5713 |
| factor(X25.CS)=0 | 3.83 | 428 | 0 | 1 | 0.5402 | 0.5108 | 0.5713 |
| factor(X25.CS)=0 | 3.84 | 427 | 0 | 2 | 0.5402 | 0.5108 | 0.5713 |
| factor(X25.CS)=0 | 3.87 | 425 | 0 | 1 | 0.5402 | 0.5108 | 0.5713 |
| factor(X25.CS)=0 | 3.88 | 424 | 0 | 1 | 0.5402 | 0.5108 | 0.5713 |
| factor(X25.CS)=0 | 3.89 | 423 | 0 | 1 | 0.5402 | 0.5108 | 0.5713 |
| factor(X25.CS)=0 | 3.9 | 422 | 0 | 2 | 0.5402 | 0.5108 | 0.5713 |
| factor(X25.CS)=0 | 3.95 | 420 | 0 | 1 | 0.5402 | 0.5108 | 0.5713 |
| factor(X25.CS)=0 | 3.96 | 419 | 0 | 1 | 0.5402 | 0.5108 | 0.5713 |
| factor(X25.CS)=0 | 3.97 | 418 | 0 | 1 | 0.5402 | 0.5108 | 0.5713 |
| factor(X25.CS)=0 | 3.99 | 417 | 1 | 0 | 0.5389 | 0.5095 | 0.5700 |
| factor(X25.CS)=0 | 4.04 | 416 | 0 | 2 | 0.5389 | 0.5095 | 0.5700 |
| factor(X25.CS)=0 | 4.05 | 414 | 0 | 2 | 0.5389 | 0.5095 | 0.5700 |
| factor(X25.CS)=0 | 4.06 | 412 | 0 | 2 | 0.5389 | 0.5095 | 0.5700 |
| factor(X25.CS)=0 | 4.07 | 410 | 1 | 1 | 0.5376 | 0.5081 | 0.5687 |
| factor(X25.CS)=0 | 4.08 | 408 | 0 | 2 | 0.5376 | 0.5081 | 0.5687 |
| factor(X25.CS)=0 | 4.13 | 406 | 0 | 2 | 0.5376 | 0.5081 | 0.5687 |
| factor(X25.CS)=0 | 4.15 | 404 | 0 | 2 | 0.5376 | 0.5081 | 0.5687 |
| factor(X25.CS)=0 | 4.16 | 402 | 0 | 1 | 0.5376 | 0.5081 | 0.5687 |
| factor(X25.CS)=0 | 4.19 | 401 | 0 | 2 | 0.5376 | 0.5081 | 0.5687 |
| factor(X25.CS)=0 | 4.22 | 399 | 0 | 2 | 0.5376 | 0.5081 | 0.5687 |
| factor(X25.CS)=0 | 4.23 | 397 | 1 | 0 | 0.5362 | 0.5067 | 0.5674 |
| factor(X25.CS)=0 | 4.26 | 396 | 0 | 1 | 0.5362 | 0.5067 | 0.5674 |
| factor(X25.CS)=0 | 4.27 | 395 | 0 | 2 | 0.5362 | 0.5067 | 0.5674 |
| factor(X25.CS)=0 | 4.31 | 393 | 0 | 2 | 0.5362 | 0.5067 | 0.5674 |
| factor(X25.CS)=0 | 4.32 | 391 | 0 | 1 | 0.5362 | 0.5067 | 0.5674 |
| factor(X25.CS)=0 | 4.33 | 390 | 0 | 1 | 0.5362 | 0.5067 | 0.5674 |
| factor(X25.CS)=0 | 4.34 | 389 | 0 | 1 | 0.5362 | 0.5067 | 0.5674 |
| factor(X25.CS)=0 | 4.35 | 388 | 0 | 4 | 0.5362 | 0.5067 | 0.5674 |
| factor(X25.CS)=0 | 4.36 | 384 | 0 | 4 | 0.5362 | 0.5067 | 0.5674 |
| factor(X25.CS)=0 | 4.38 | 380 | 0 | 3 | 0.5362 | 0.5067 | 0.5674 |
| factor(X25.CS)=0 | 4.4 | 377 | 0 | 1 | 0.5362 | 0.5067 | 0.5674 |
| factor(X25.CS)=0 | 4.41 | 376 | 0 | 2 | 0.5362 | 0.5067 | 0.5674 |
| factor(X25.CS)=0 | 4.42 | 374 | 0 | 2 | 0.5362 | 0.5067 | 0.5674 |
| factor(X25.CS)=0 | 4.44 | 372 | 0 | 3 | 0.5362 | 0.5067 | 0.5674 |
| factor(X25.CS)=0 | 4.45 | 369 | 0 | 1 | 0.5362 | 0.5067 | 0.5674 |
| factor(X25.CS)=0 | 4.47 | 368 | 0 | 2 | 0.5362 | 0.5067 | 0.5674 |
| factor(X25.CS)=0 | 4.48 | 366 | 0 | 2 | 0.5362 | 0.5067 | 0.5674 |
| factor(X25.CS)=0 | 4.49 | 364 | 0 | 2 | 0.5362 | 0.5067 | 0.5674 |
| factor(X25.CS)=0 | 4.5 | 362 | 0 | 1 | 0.5362 | 0.5067 | 0.5674 |
| factor(X25.CS)=0 | 4.53 | 361 | 0 | 3 | 0.5362 | 0.5067 | 0.5674 |
| factor(X25.CS)=0 | 4.54 | 358 | 0 | 1 | 0.5362 | 0.5067 | 0.5674 |
| factor(X25.CS)=0 | 4.55 | 357 | 1 | 1 | 0.5347 | 0.5052 | 0.5660 |
| factor(X25.CS)=0 | 4.58 | 355 | 0 | 2 | 0.5347 | 0.5052 | 0.5660 |
| factor(X25.CS)=0 | 4.59 | 353 | 0 | 1 | 0.5347 | 0.5052 | 0.5660 |
| factor(X25.CS)=0 | 4.6 | 352 | 0 | 4 | 0.5347 | 0.5052 | 0.5660 |
| factor(X25.CS)=0 | 4.61 | 348 | 0 | 3 | 0.5347 | 0.5052 | 0.5660 |
| factor(X25.CS)=0 | 4.62 | 345 | 0 | 1 | 0.5347 | 0.5052 | 0.5660 |
| factor(X25.CS)=0 | 4.64 | 344 | 0 | 1 | 0.5347 | 0.5052 | 0.5660 |
| factor(X25.CS)=0 | 4.65 | 343 | 0 | 1 | 0.5347 | 0.5052 | 0.5660 |
| factor(X25.CS)=0 | 4.68 | 342 | 1 | 0 | 0.5331 | 0.5036 | 0.5645 |
| factor(X25.CS)=0 | 4.7 | 341 | 0 | 1 | 0.5331 | 0.5036 | 0.5645 |
| factor(X25.CS)=0 | 4.72 | 340 | 1 | 1 | 0.5316 | 0.5019 | 0.5630 |
| factor(X25.CS)=0 | 4.73 | 338 | 0 | 2 | 0.5316 | 0.5019 | 0.5630 |
| factor(X25.CS)=0 | 4.75 | 336 | 0 | 1 | 0.5316 | 0.5019 | 0.5630 |
| factor(X25.CS)=0 | 4.79 | 335 | 0 | 1 | 0.5316 | 0.5019 | 0.5630 |
| factor(X25.CS)=0 | 4.81 | 334 | 0 | 2 | 0.5316 | 0.5019 | 0.5630 |
| factor(X25.CS)=0 | 4.82 | 332 | 0 | 1 | 0.5316 | 0.5019 | 0.5630 |
| factor(X25.CS)=0 | 4.85 | 331 | 0 | 4 | 0.5316 | 0.5019 | 0.5630 |
| factor(X25.CS)=0 | 4.88 | 327 | 0 | 1 | 0.5316 | 0.5019 | 0.5630 |
| factor(X25.CS)=0 | 4.89 | 326 | 0 | 1 | 0.5316 | 0.5019 | 0.5630 |
| factor(X25.CS)=0 | 4.9 | 325 | 0 | 1 | 0.5316 | 0.5019 | 0.5630 |
| factor(X25.CS)=0 | 4.91 | 324 | 0 | 1 | 0.5316 | 0.5019 | 0.5630 |
| factor(X25.CS)=0 | 4.94 | 323 | 0 | 2 | 0.5316 | 0.5019 | 0.5630 |
| factor(X25.CS)=0 | 4.97 | 321 | 0 | 1 | 0.5316 | 0.5019 | 0.5630 |
| factor(X25.CS)=0 | 4.99 | 320 | 0 | 1 | 0.5316 | 0.5019 | 0.5630 |
| factor(X25.CS)=0 | 5 | 319 | 0 | 319 | 0.5316 | 0.5019 | 0.5630 |
| factor(X25.CS)=1 | 0.13 | 137 | 2 | 0 | 0.9854 | 0.9655 | 1.0000 |
| factor(X25.CS)=1 | 0.15 | 135 | 2 | 0 | 0.9708 | 0.9430 | 0.9994 |
| factor(X25.CS)=1 | 0.16 | 133 | 5 | 0 | 0.9343 | 0.8937 | 0.9767 |
| factor(X25.CS)=1 | 0.17 | 128 | 4 | 0 | 0.9051 | 0.8573 | 0.9555 |
| factor(X25.CS)=1 | 0.18 | 124 | 2 | 0 | 0.8905 | 0.8397 | 0.9444 |
| factor(X25.CS)=1 | 0.19 | 122 | 10 | 0 | 0.8175 | 0.7553 | 0.8848 |
| factor(X25.CS)=1 | 0.2 | 112 | 3 | 0 | 0.7956 | 0.7309 | 0.8661 |
| factor(X25.CS)=1 | 0.21 | 109 | 10 | 0 | 0.7226 | 0.6514 | 0.8016 |
| factor(X25.CS)=1 | 0.22 | 99 | 9 | 0 | 0.6569 | 0.5821 | 0.7414 |
| factor(X25.CS)=1 | 0.23 | 90 | 8 | 0 | 0.5985 | 0.5218 | 0.6865 |
| factor(X25.CS)=1 | 0.24 | 82 | 3 | 0 | 0.5766 | 0.4996 | 0.6656 |
| factor(X25.CS)=1 | 0.25 | 79 | 2 | 0 | 0.5620 | 0.4848 | 0.6516 |
| factor(X25.CS)=1 | 0.27 | 77 | 1 | 0 | 0.5547 | 0.4775 | 0.6445 |
| factor(X25.CS)=1 | 0.28 | 76 | 3 | 0 | 0.5328 | 0.4555 | 0.6233 |
| factor(X25.CS)=1 | 0.29 | 73 | 1 | 0 | 0.5255 | 0.4482 | 0.6162 |
| factor(X25.CS)=1 | 0.3 | 72 | 3 | 0 | 0.5036 | 0.4265 | 0.5947 |
| factor(X25.CS)=1 | 0.32 | 69 | 1 | 0 | 0.4964 | 0.4193 | 0.5875 |
| factor(X25.CS)=1 | 0.34 | 68 | 1 | 0 | 0.4891 | 0.4121 | 0.5803 |
| factor(X25.CS)=1 | 0.35 | 67 | 1 | 0 | 0.4818 | 0.4049 | 0.5731 |
| factor(X25.CS)=1 | 0.47 | 66 | 2 | 0 | 0.4672 | 0.3907 | 0.5586 |
| factor(X25.CS)=1 | 0.49 | 64 | 3 | 0 | 0.4453 | 0.3693 | 0.5368 |
| factor(X25.CS)=1 | 0.5 | 61 | 1 | 0 | 0.4380 | 0.3623 | 0.5294 |
| factor(X25.CS)=1 | 0.53 | 60 | 1 | 0 | 0.4307 | 0.3552 | 0.5221 |
| factor(X25.CS)=1 | 0.59 | 59 | 1 | 0 | 0.4234 | 0.3482 | 0.5147 |
| factor(X25.CS)=1 | 0.62 | 58 | 1 | 0 | 0.4161 | 0.3412 | 0.5074 |
| factor(X25.CS)=1 | 0.64 | 57 | 3 | 0 | 0.3942 | 0.3203 | 0.4851 |
| factor(X25.CS)=1 | 0.69 | 54 | 1 | 0 | 0.3869 | 0.3133 | 0.4776 |
| factor(X25.CS)=1 | 0.7 | 53 | 2 | 0 | 0.3723 | 0.2995 | 0.4627 |
| factor(X25.CS)=1 | 0.72 | 51 | 1 | 0 | 0.3650 | 0.2926 | 0.4552 |
| factor(X25.CS)=1 | 0.74 | 50 | 2 | 0 | 0.3504 | 0.2789 | 0.4401 |
| factor(X25.CS)=1 | 0.77 | 48 | 2 | 0 | 0.3358 | 0.2653 | 0.4249 |
| factor(X25.CS)=1 | 0.78 | 46 | 1 | 0 | 0.3285 | 0.2585 | 0.4173 |
| factor(X25.CS)=1 | 0.86 | 45 | 0 | 1 | 0.3285 | 0.2585 | 0.4173 |
| factor(X25.CS)=1 | 0.87 | 44 | 1 | 0 | 0.3210 | 0.2516 | 0.4096 |
| factor(X25.CS)=1 | 0.9 | 43 | 1 | 0 | 0.3135 | 0.2447 | 0.4018 |
| factor(X25.CS)=1 | 0.91 | 42 | 1 | 0 | 0.3061 | 0.2378 | 0.3940 |
| factor(X25.CS)=1 | 0.95 | 41 | 1 | 0 | 0.2986 | 0.2309 | 0.3861 |
| factor(X25.CS)=1 | 1 | 40 | 1 | 0 | 0.2911 | 0.2241 | 0.3783 |
| factor(X25.CS)=1 | 1.02 | 39 | 1 | 0 | 0.2837 | 0.2173 | 0.3704 |
| factor(X25.CS)=1 | 1.07 | 38 | 1 | 0 | 0.2762 | 0.2105 | 0.3625 |
| factor(X25.CS)=1 | 1.15 | 37 | 2 | 0 | 0.2613 | 0.1970 | 0.3466 |
| factor(X25.CS)=1 | 1.17 | 35 | 1 | 0 | 0.2538 | 0.1903 | 0.3386 |
| factor(X25.CS)=1 | 1.38 | 34 | 1 | 0 | 0.2464 | 0.1836 | 0.3305 |
| factor(X25.CS)=1 | 1.42 | 33 | 3 | 0 | 0.2240 | 0.1637 | 0.3063 |
| factor(X25.CS)=1 | 1.74 | 30 | 1 | 0 | 0.2165 | 0.1572 | 0.2982 |
| factor(X25.CS)=1 | 2.26 | 29 | 1 | 0 | 0.2090 | 0.1507 | 0.2900 |
| factor(X25.CS)=1 | 2.72 | 28 | 3 | 0 | 0.1866 | 0.1313 | 0.2653 |
| factor(X25.CS)=1 | 3.39 | 25 | 2 | 0 | 0.1717 | 0.1186 | 0.2486 |
| factor(X25.CS)=1 | 3.68 | 23 | 0 | 1 | 0.1717 | 0.1186 | 0.2486 |
| factor(X25.CS)=1 | 4.61 | 22 | 0 | 1 | 0.1717 | 0.1186 | 0.2486 |
| factor(X25.CS)=1 | 4.87 | 21 | 0 | 2 | 0.1717 | 0.1186 | 0.2486 |
| factor(X25.CS)=1 | 5 | 19 | 0 | 19 | 0.1717 | 0.1186 | 0.2486 |

|  |  |  |  |  |  |  |  |  |  |
| --- | --- | --- | --- | --- | --- | --- | --- | --- | --- |
| X25.CS | records | n.max | n.start | events | \*rmean | \*se(rmean) | median | 0.95LCL | 0.95UCL |
| factor(X25.CS)=0 | 1068 | 1068 | 1068 | 490 | 3.155 | 0.063 | NA | NA | NA |
| factor(X25.CS)=1 | 137 | 137 | 137 | 113 | 1.326 | 0.152 | 0.32 | 0.24 | 0.64 |

Landmark analysis for time segment: EFS(years) > 5
Log rank test: implements the G-rho family of Harrington and Fleming (1982), with weights on each death of S(t)^rho, where S is the Kaplan-Meier estimate of survival. With rho = 0 this is the log-rank or Mantel-Haenszel test.

|  |  |  |  |
| --- | --- | --- | --- |
|  | N | Observed | Expected |
| factor(X25.CS)=0 | 318 | 7 | 7.4079 |
| factor(X25.CS)=1 | 19 | 1 | 0.5921 |

Chisq=
0.3055
on
1
degree of freedom, p=
0.5805
Survival table

|  |  |  |  |  |  |  |  |
| --- | --- | --- | --- | --- | --- | --- | --- |
|  | EFS(years) | N.Risk | N.Event | N.Censor | Survival | 95%CI Low | 95%CI Upp |
| factor(X25.CS)=0 | 5.02 | 318 | 0 | 1 | 1.0000 | 1.0000 | 1.0000 |
| factor(X25.CS)=0 | 5.03 | 317 | 0 | 1 | 1.0000 | 1.0000 | 1.0000 |
| factor(X25.CS)=0 | 5.04 | 316 | 0 | 1 | 1.0000 | 1.0000 | 1.0000 |
| factor(X25.CS)=0 | 5.05 | 315 | 0 | 2 | 1.0000 | 1.0000 | 1.0000 |
| factor(X25.CS)=0 | 5.08 | 313 | 0 | 1 | 1.0000 | 1.0000 | 1.0000 |
| factor(X25.CS)=0 | 5.1 | 312 | 0 | 1 | 1.0000 | 1.0000 | 1.0000 |
| factor(X25.CS)=0 | 5.12 | 311 | 1 | 1 | 0.9968 | 0.9905 | 1.0000 |
| factor(X25.CS)=0 | 5.14 | 309 | 0 | 1 | 0.9968 | 0.9905 | 1.0000 |
| factor(X25.CS)=0 | 5.15 | 308 | 0 | 2 | 0.9968 | 0.9905 | 1.0000 |
| factor(X25.CS)=0 | 5.16 | 306 | 0 | 1 | 0.9968 | 0.9905 | 1.0000 |
| factor(X25.CS)=0 | 5.17 | 305 | 0 | 1 | 0.9968 | 0.9905 | 1.0000 |
| factor(X25.CS)=0 | 5.18 | 304 | 0 | 3 | 0.9968 | 0.9905 | 1.0000 |
| factor(X25.CS)=0 | 5.2 | 301 | 0 | 2 | 0.9968 | 0.9905 | 1.0000 |
| factor(X25.CS)=0 | 5.22 | 299 | 0 | 1 | 0.9968 | 0.9905 | 1.0000 |
| factor(X25.CS)=0 | 5.25 | 298 | 0 | 2 | 0.9968 | 0.9905 | 1.0000 |
| factor(X25.CS)=0 | 5.26 | 296 | 0 | 2 | 0.9968 | 0.9905 | 1.0000 |
| factor(X25.CS)=0 | 5.27 | 294 | 0 | 2 | 0.9968 | 0.9905 | 1.0000 |
| factor(X25.CS)=0 | 5.29 | 292 | 0 | 2 | 0.9968 | 0.9905 | 1.0000 |
| factor(X25.CS)=0 | 5.3 | 290 | 0 | 1 | 0.9968 | 0.9905 | 1.0000 |
| factor(X25.CS)=0 | 5.31 | 289 | 0 | 1 | 0.9968 | 0.9905 | 1.0000 |
| factor(X25.CS)=0 | 5.32 | 288 | 0 | 2 | 0.9968 | 0.9905 | 1.0000 |
| factor(X25.CS)=0 | 5.33 | 286 | 0 | 2 | 0.9968 | 0.9905 | 1.0000 |
| factor(X25.CS)=0 | 5.34 | 284 | 0 | 2 | 0.9968 | 0.9905 | 1.0000 |
| factor(X25.CS)=0 | 5.35 | 282 | 0 | 6 | 0.9968 | 0.9905 | 1.0000 |
| factor(X25.CS)=0 | 5.36 | 276 | 0 | 2 | 0.9968 | 0.9905 | 1.0000 |
| factor(X25.CS)=0 | 5.37 | 274 | 0 | 2 | 0.9968 | 0.9905 | 1.0000 |
| factor(X25.CS)=0 | 5.38 | 272 | 0 | 2 | 0.9968 | 0.9905 | 1.0000 |
| factor(X25.CS)=0 | 5.39 | 270 | 0 | 1 | 0.9968 | 0.9905 | 1.0000 |
| factor(X25.CS)=0 | 5.4 | 269 | 0 | 1 | 0.9968 | 0.9905 | 1.0000 |
| factor(X25.CS)=0 | 5.41 | 268 | 0 | 2 | 0.9968 | 0.9905 | 1.0000 |
| factor(X25.CS)=0 | 5.42 | 266 | 0 | 4 | 0.9968 | 0.9905 | 1.0000 |
| factor(X25.CS)=0 | 5.43 | 262 | 0 | 1 | 0.9968 | 0.9905 | 1.0000 |
| factor(X25.CS)=0 | 5.44 | 261 | 0 | 4 | 0.9968 | 0.9905 | 1.0000 |
| factor(X25.CS)=0 | 5.45 | 257 | 0 | 1 | 0.9968 | 0.9905 | 1.0000 |
| factor(X25.CS)=0 | 5.46 | 256 | 0 | 2 | 0.9968 | 0.9905 | 1.0000 |
| factor(X25.CS)=0 | 5.48 | 254 | 0 | 3 | 0.9968 | 0.9905 | 1.0000 |
| factor(X25.CS)=0 | 5.49 | 251 | 0 | 1 | 0.9968 | 0.9905 | 1.0000 |
| factor(X25.CS)=0 | 5.5 | 250 | 0 | 1 | 0.9968 | 0.9905 | 1.0000 |
| factor(X25.CS)=0 | 5.51 | 249 | 0 | 2 | 0.9968 | 0.9905 | 1.0000 |
| factor(X25.CS)=0 | 5.52 | 247 | 0 | 2 | 0.9968 | 0.9905 | 1.0000 |
| factor(X25.CS)=0 | 5.54 | 245 | 0 | 4 | 0.9968 | 0.9905 | 1.0000 |
| factor(X25.CS)=0 | 5.55 | 241 | 0 | 2 | 0.9968 | 0.9905 | 1.0000 |
| factor(X25.CS)=0 | 5.56 | 239 | 0 | 2 | 0.9968 | 0.9905 | 1.0000 |
| factor(X25.CS)=0 | 5.57 | 237 | 0 | 1 | 0.9968 | 0.9905 | 1.0000 |
| factor(X25.CS)=0 | 5.58 | 236 | 1 | 1 | 0.9926 | 0.9822 | 1.0000 |
| factor(X25.CS)=0 | 5.6 | 234 | 0 | 1 | 0.9926 | 0.9822 | 1.0000 |
| factor(X25.CS)=0 | 5.61 | 233 | 0 | 1 | 0.9926 | 0.9822 | 1.0000 |
| factor(X25.CS)=0 | 5.63 | 232 | 0 | 2 | 0.9926 | 0.9822 | 1.0000 |
| factor(X25.CS)=0 | 5.65 | 230 | 0 | 1 | 0.9926 | 0.9822 | 1.0000 |
| factor(X25.CS)=0 | 5.66 | 229 | 0 | 1 | 0.9926 | 0.9822 | 1.0000 |
| factor(X25.CS)=0 | 5.67 | 228 | 0 | 3 | 0.9926 | 0.9822 | 1.0000 |
| factor(X25.CS)=0 | 5.7 | 225 | 0 | 3 | 0.9926 | 0.9822 | 1.0000 |
| factor(X25.CS)=0 | 5.71 | 222 | 0 | 2 | 0.9926 | 0.9822 | 1.0000 |
| factor(X25.CS)=0 | 5.72 | 220 | 0 | 1 | 0.9926 | 0.9822 | 1.0000 |
| factor(X25.CS)=0 | 5.73 | 219 | 0 | 2 | 0.9926 | 0.9822 | 1.0000 |
| factor(X25.CS)=0 | 5.74 | 217 | 0 | 1 | 0.9926 | 0.9822 | 1.0000 |
| factor(X25.CS)=0 | 5.75 | 216 | 0 | 2 | 0.9926 | 0.9822 | 1.0000 |
| factor(X25.CS)=0 | 5.77 | 214 | 0 | 1 | 0.9926 | 0.9822 | 1.0000 |
| factor(X25.CS)=0 | 5.78 | 213 | 0 | 2 | 0.9926 | 0.9822 | 1.0000 |
| factor(X25.CS)=0 | 5.79 | 211 | 0 | 1 | 0.9926 | 0.9822 | 1.0000 |
| factor(X25.CS)=0 | 5.8 | 210 | 0 | 1 | 0.9926 | 0.9822 | 1.0000 |
| factor(X25.CS)=0 | 5.81 | 209 | 0 | 2 | 0.9926 | 0.9822 | 1.0000 |
| factor(X25.CS)=0 | 5.84 | 207 | 0 | 2 | 0.9926 | 0.9822 | 1.0000 |
| factor(X25.CS)=0 | 5.85 | 205 | 0 | 1 | 0.9926 | 0.9822 | 1.0000 |
| factor(X25.CS)=0 | 5.88 | 204 | 0 | 1 | 0.9926 | 0.9822 | 1.0000 |
| factor(X25.CS)=0 | 5.89 | 203 | 0 | 2 | 0.9926 | 0.9822 | 1.0000 |
| factor(X25.CS)=0 | 5.92 | 201 | 0 | 1 | 0.9926 | 0.9822 | 1.0000 |
| factor(X25.CS)=0 | 5.93 | 200 | 0 | 1 | 0.9926 | 0.9822 | 1.0000 |
| factor(X25.CS)=0 | 5.94 | 199 | 0 | 2 | 0.9926 | 0.9822 | 1.0000 |
| factor(X25.CS)=0 | 5.95 | 197 | 0 | 3 | 0.9926 | 0.9822 | 1.0000 |
| factor(X25.CS)=0 | 5.96 | 194 | 0 | 1 | 0.9926 | 0.9822 | 1.0000 |
| factor(X25.CS)=0 | 5.98 | 193 | 0 | 1 | 0.9926 | 0.9822 | 1.0000 |
| factor(X25.CS)=0 | 6 | 192 | 0 | 6 | 0.9926 | 0.9822 | 1.0000 |
| factor(X25.CS)=0 | 6.01 | 186 | 0 | 1 | 0.9926 | 0.9822 | 1.0000 |
| factor(X25.CS)=0 | 6.02 | 185 | 0 | 1 | 0.9926 | 0.9822 | 1.0000 |
| factor(X25.CS)=0 | 6.04 | 184 | 0 | 2 | 0.9926 | 0.9822 | 1.0000 |
| factor(X25.CS)=0 | 6.07 | 182 | 0 | 1 | 0.9926 | 0.9822 | 1.0000 |
| factor(X25.CS)=0 | 6.08 | 181 | 0 | 1 | 0.9926 | 0.9822 | 1.0000 |
| factor(X25.CS)=0 | 6.12 | 180 | 0 | 2 | 0.9926 | 0.9822 | 1.0000 |
| factor(X25.CS)=0 | 6.15 | 178 | 0 | 3 | 0.9926 | 0.9822 | 1.0000 |
| factor(X25.CS)=0 | 6.16 | 175 | 0 | 2 | 0.9926 | 0.9822 | 1.0000 |
| factor(X25.CS)=0 | 6.17 | 173 | 0 | 1 | 0.9926 | 0.9822 | 1.0000 |
| factor(X25.CS)=0 | 6.18 | 172 | 0 | 2 | 0.9926 | 0.9822 | 1.0000 |
| factor(X25.CS)=0 | 6.22 | 170 | 0 | 1 | 0.9926 | 0.9822 | 1.0000 |
| factor(X25.CS)=0 | 6.25 | 169 | 0 | 2 | 0.9926 | 0.9822 | 1.0000 |
| factor(X25.CS)=0 | 6.27 | 167 | 0 | 1 | 0.9926 | 0.9822 | 1.0000 |
| factor(X25.CS)=0 | 6.28 | 166 | 0 | 1 | 0.9926 | 0.9822 | 1.0000 |
| factor(X25.CS)=0 | 6.29 | 165 | 0 | 1 | 0.9926 | 0.9822 | 1.0000 |
| factor(X25.CS)=0 | 6.32 | 164 | 0 | 1 | 0.9926 | 0.9822 | 1.0000 |
| factor(X25.CS)=0 | 6.35 | 163 | 0 | 2 | 0.9926 | 0.9822 | 1.0000 |
| factor(X25.CS)=0 | 6.36 | 161 | 0 | 1 | 0.9926 | 0.9822 | 1.0000 |
| factor(X25.CS)=0 | 6.37 | 160 | 0 | 2 | 0.9926 | 0.9822 | 1.0000 |
| factor(X25.CS)=0 | 6.38 | 158 | 0 | 2 | 0.9926 | 0.9822 | 1.0000 |
| factor(X25.CS)=0 | 6.39 | 156 | 0 | 4 | 0.9926 | 0.9822 | 1.0000 |
| factor(X25.CS)=0 | 6.4 | 152 | 0 | 2 | 0.9926 | 0.9822 | 1.0000 |
| factor(X25.CS)=0 | 6.42 | 150 | 0 | 3 | 0.9926 | 0.9822 | 1.0000 |
| factor(X25.CS)=0 | 6.45 | 147 | 3 | 1 | 0.9723 | 0.9478 | 0.9975 |
| factor(X25.CS)=0 | 6.46 | 143 | 0 | 2 | 0.9723 | 0.9478 | 0.9975 |
| factor(X25.CS)=0 | 6.47 | 141 | 0 | 3 | 0.9723 | 0.9478 | 0.9975 |
| factor(X25.CS)=0 | 6.48 | 138 | 0 | 2 | 0.9723 | 0.9478 | 0.9975 |
| factor(X25.CS)=0 | 6.49 | 136 | 0 | 1 | 0.9723 | 0.9478 | 0.9975 |
| factor(X25.CS)=0 | 6.5 | 135 | 0 | 2 | 0.9723 | 0.9478 | 0.9975 |
| factor(X25.CS)=0 | 6.51 | 133 | 0 | 2 | 0.9723 | 0.9478 | 0.9975 |
| factor(X25.CS)=0 | 6.52 | 131 | 0 | 1 | 0.9723 | 0.9478 | 0.9975 |
| factor(X25.CS)=0 | 6.53 | 130 | 0 | 1 | 0.9723 | 0.9478 | 0.9975 |
| factor(X25.CS)=0 | 6.54 | 129 | 0 | 1 | 0.9723 | 0.9478 | 0.9975 |
| factor(X25.CS)=0 | 6.55 | 128 | 0 | 1 | 0.9723 | 0.9478 | 0.9975 |
| factor(X25.CS)=0 | 6.56 | 127 | 1 | 1 | 0.9646 | 0.9362 | 0.9939 |
| factor(X25.CS)=0 | 6.57 | 125 | 0 | 2 | 0.9646 | 0.9362 | 0.9939 |
| factor(X25.CS)=0 | 6.58 | 123 | 0 | 2 | 0.9646 | 0.9362 | 0.9939 |
| factor(X25.CS)=0 | 6.59 | 121 | 0 | 1 | 0.9646 | 0.9362 | 0.9939 |
| factor(X25.CS)=0 | 6.6 | 120 | 0 | 1 | 0.9646 | 0.9362 | 0.9939 |
| factor(X25.CS)=0 | 6.63 | 119 | 0 | 1 | 0.9646 | 0.9362 | 0.9939 |
| factor(X25.CS)=0 | 6.64 | 118 | 0 | 2 | 0.9646 | 0.9362 | 0.9939 |
| factor(X25.CS)=0 | 6.65 | 116 | 0 | 3 | 0.9646 | 0.9362 | 0.9939 |
| factor(X25.CS)=0 | 6.68 | 113 | 0 | 1 | 0.9646 | 0.9362 | 0.9939 |
| factor(X25.CS)=0 | 6.69 | 112 | 0 | 1 | 0.9646 | 0.9362 | 0.9939 |
| factor(X25.CS)=0 | 6.73 | 111 | 0 | 1 | 0.9646 | 0.9362 | 0.9939 |
| factor(X25.CS)=0 | 6.75 | 110 | 0 | 3 | 0.9646 | 0.9362 | 0.9939 |
| factor(X25.CS)=0 | 6.77 | 107 | 0 | 1 | 0.9646 | 0.9362 | 0.9939 |
| factor(X25.CS)=0 | 6.78 | 106 | 0 | 1 | 0.9646 | 0.9362 | 0.9939 |
| factor(X25.CS)=0 | 6.79 | 105 | 0 | 2 | 0.9646 | 0.9362 | 0.9939 |
| factor(X25.CS)=0 | 6.81 | 103 | 0 | 2 | 0.9646 | 0.9362 | 0.9939 |
| factor(X25.CS)=0 | 6.85 | 101 | 0 | 1 | 0.9646 | 0.9362 | 0.9939 |
| factor(X25.CS)=0 | 6.87 | 100 | 0 | 1 | 0.9646 | 0.9362 | 0.9939 |
| factor(X25.CS)=0 | 6.89 | 99 | 0 | 2 | 0.9646 | 0.9362 | 0.9939 |
| factor(X25.CS)=0 | 6.91 | 97 | 0 | 1 | 0.9646 | 0.9362 | 0.9939 |
| factor(X25.CS)=0 | 6.92 | 96 | 0 | 1 | 0.9646 | 0.9362 | 0.9939 |
| factor(X25.CS)=0 | 6.94 | 95 | 0 | 1 | 0.9646 | 0.9362 | 0.9939 |
| factor(X25.CS)=0 | 6.96 | 94 | 0 | 4 | 0.9646 | 0.9362 | 0.9939 |
| factor(X25.CS)=0 | 6.98 | 90 | 0 | 1 | 0.9646 | 0.9362 | 0.9939 |
| factor(X25.CS)=0 | 7 | 89 | 0 | 1 | 0.9646 | 0.9362 | 0.9939 |
| factor(X25.CS)=0 | 7.08 | 88 | 0 | 1 | 0.9646 | 0.9362 | 0.9939 |
| factor(X25.CS)=0 | 7.14 | 87 | 0 | 1 | 0.9646 | 0.9362 | 0.9939 |
| factor(X25.CS)=0 | 7.16 | 86 | 0 | 1 | 0.9646 | 0.9362 | 0.9939 |
| factor(X25.CS)=0 | 7.2 | 85 | 0 | 1 | 0.9646 | 0.9362 | 0.9939 |
| factor(X25.CS)=0 | 7.21 | 84 | 0 | 1 | 0.9646 | 0.9362 | 0.9939 |
| factor(X25.CS)=0 | 7.26 | 83 | 0 | 3 | 0.9646 | 0.9362 | 0.9939 |
| factor(X25.CS)=0 | 7.29 | 80 | 0 | 1 | 0.9646 | 0.9362 | 0.9939 |
| factor(X25.CS)=0 | 7.35 | 79 | 0 | 2 | 0.9646 | 0.9362 | 0.9939 |
| factor(X25.CS)=0 | 7.36 | 77 | 0 | 6 | 0.9646 | 0.9362 | 0.9939 |
| factor(X25.CS)=0 | 7.37 | 71 | 0 | 1 | 0.9646 | 0.9362 | 0.9939 |
| factor(X25.CS)=0 | 7.38 | 70 | 0 | 1 | 0.9646 | 0.9362 | 0.9939 |
| factor(X25.CS)=0 | 7.39 | 69 | 0 | 2 | 0.9646 | 0.9362 | 0.9939 |
| factor(X25.CS)=0 | 7.4 | 67 | 0 | 1 | 0.9646 | 0.9362 | 0.9939 |
| factor(X25.CS)=0 | 7.41 | 66 | 0 | 1 | 0.9646 | 0.9362 | 0.9939 |
| factor(X25.CS)=0 | 7.42 | 65 | 0 | 1 | 0.9646 | 0.9362 | 0.9939 |
| factor(X25.CS)=0 | 7.45 | 64 | 0 | 1 | 0.9646 | 0.9362 | 0.9939 |
| factor(X25.CS)=0 | 7.47 | 63 | 0 | 1 | 0.9646 | 0.9362 | 0.9939 |
| factor(X25.CS)=0 | 7.48 | 62 | 0 | 3 | 0.9646 | 0.9362 | 0.9939 |
| factor(X25.CS)=0 | 7.5 | 59 | 0 | 1 | 0.9646 | 0.9362 | 0.9939 |
| factor(X25.CS)=0 | 7.53 | 58 | 0 | 1 | 0.9646 | 0.9362 | 0.9939 |
| factor(X25.CS)=0 | 7.54 | 57 | 0 | 1 | 0.9646 | 0.9362 | 0.9939 |
| factor(X25.CS)=0 | 7.55 | 56 | 0 | 1 | 0.9646 | 0.9362 | 0.9939 |
| factor(X25.CS)=0 | 7.56 | 55 | 0 | 2 | 0.9646 | 0.9362 | 0.9939 |
| factor(X25.CS)=0 | 7.58 | 53 | 0 | 4 | 0.9646 | 0.9362 | 0.9939 |
| factor(X25.CS)=0 | 7.59 | 49 | 0 | 1 | 0.9646 | 0.9362 | 0.9939 |
| factor(X25.CS)=0 | 7.6 | 48 | 0 | 2 | 0.9646 | 0.9362 | 0.9939 |
| factor(X25.CS)=0 | 7.61 | 46 | 0 | 1 | 0.9646 | 0.9362 | 0.9939 |
| factor(X25.CS)=0 | 7.62 | 45 | 0 | 1 | 0.9646 | 0.9362 | 0.9939 |
| factor(X25.CS)=0 | 7.67 | 44 | 0 | 1 | 0.9646 | 0.9362 | 0.9939 |
| factor(X25.CS)=0 | 7.68 | 43 | 0 | 1 | 0.9646 | 0.9362 | 0.9939 |
| factor(X25.CS)=0 | 7.69 | 42 | 0 | 2 | 0.9646 | 0.9362 | 0.9939 |
| factor(X25.CS)=0 | 7.73 | 40 | 0 | 2 | 0.9646 | 0.9362 | 0.9939 |
| factor(X25.CS)=0 | 7.74 | 38 | 0 | 3 | 0.9646 | 0.9362 | 0.9939 |
| factor(X25.CS)=0 | 7.81 | 35 | 0 | 1 | 0.9646 | 0.9362 | 0.9939 |
| factor(X25.CS)=0 | 7.82 | 34 | 0 | 1 | 0.9646 | 0.9362 | 0.9939 |
| factor(X25.CS)=0 | 7.9 | 33 | 0 | 1 | 0.9646 | 0.9362 | 0.9939 |
| factor(X25.CS)=0 | 7.94 | 32 | 0 | 2 | 0.9646 | 0.9362 | 0.9939 |
| factor(X25.CS)=0 | 7.98 | 30 | 0 | 1 | 0.9646 | 0.9362 | 0.9939 |
| factor(X25.CS)=0 | 7.99 | 29 | 1 | 1 | 0.9314 | 0.8641 | 1.0000 |
| factor(X25.CS)=0 | 8.01 | 27 | 0 | 1 | 0.9314 | 0.8641 | 1.0000 |
| factor(X25.CS)=0 | 8.06 | 26 | 0 | 1 | 0.9314 | 0.8641 | 1.0000 |
| factor(X25.CS)=0 | 8.07 | 25 | 0 | 1 | 0.9314 | 0.8641 | 1.0000 |
| factor(X25.CS)=0 | 8.17 | 24 | 0 | 1 | 0.9314 | 0.8641 | 1.0000 |
| factor(X25.CS)=0 | 8.21 | 23 | 0 | 1 | 0.9314 | 0.8641 | 1.0000 |
| factor(X25.CS)=0 | 8.26 | 22 | 0 | 1 | 0.9314 | 0.8641 | 1.0000 |
| factor(X25.CS)=0 | 8.27 | 21 | 0 | 1 | 0.9314 | 0.8641 | 1.0000 |
| factor(X25.CS)=0 | 8.28 | 20 | 0 | 1 | 0.9314 | 0.8641 | 1.0000 |
| factor(X25.CS)=0 | 8.29 | 19 | 0 | 1 | 0.9314 | 0.8641 | 1.0000 |
| factor(X25.CS)=0 | 8.36 | 18 | 0 | 1 | 0.9314 | 0.8641 | 1.0000 |
| factor(X25.CS)=0 | 8.48 | 17 | 0 | 1 | 0.9314 | 0.8641 | 1.0000 |
| factor(X25.CS)=0 | 8.49 | 16 | 0 | 2 | 0.9314 | 0.8641 | 1.0000 |
| factor(X25.CS)=0 | 8.5 | 14 | 0 | 1 | 0.9314 | 0.8641 | 1.0000 |
| factor(X25.CS)=0 | 8.53 | 13 | 0 | 1 | 0.9314 | 0.8641 | 1.0000 |
| factor(X25.CS)=0 | 8.72 | 12 | 0 | 1 | 0.9314 | 0.8641 | 1.0000 |
| factor(X25.CS)=0 | 8.84 | 11 | 0 | 1 | 0.9314 | 0.8641 | 1.0000 |
| factor(X25.CS)=0 | 8.88 | 10 | 0 | 3 | 0.9314 | 0.8641 | 1.0000 |
| factor(X25.CS)=0 | 9.04 | 7 | 0 | 1 | 0.9314 | 0.8641 | 1.0000 |
| factor(X25.CS)=0 | 9.06 | 6 | 0 | 2 | 0.9314 | 0.8641 | 1.0000 |
| factor(X25.CS)=0 | 9.08 | 4 | 0 | 1 | 0.9314 | 0.8641 | 1.0000 |
| factor(X25.CS)=0 | 9.26 | 3 | 0 | 1 | 0.9314 | 0.8641 | 1.0000 |
| factor(X25.CS)=0 | 9.61 | 2 | 0 | 1 | 0.9314 | 0.8641 | 1.0000 |
| factor(X25.CS)=0 | 9.95 | 1 | 0 | 1 | 0.9314 | 0.8641 | 1.0000 |
| factor(X25.CS)=1 | 5.33 | 19 | 0 | 1 | 1.0000 | 1.0000 | 1.0000 |
| factor(X25.CS)=1 | 5.38 | 18 | 0 | 1 | 1.0000 | 1.0000 | 1.0000 |
| factor(X25.CS)=1 | 5.4 | 17 | 0 | 1 | 1.0000 | 1.0000 | 1.0000 |
| factor(X25.CS)=1 | 5.81 | 16 | 1 | 0 | 0.9375 | 0.8261 | 1.0000 |
| factor(X25.CS)=1 | 6.19 | 15 | 0 | 1 | 0.9375 | 0.8261 | 1.0000 |
| factor(X25.CS)=1 | 6.2 | 14 | 0 | 1 | 0.9375 | 0.8261 | 1.0000 |
| factor(X25.CS)=1 | 6.4 | 13 | 0 | 1 | 0.9375 | 0.8261 | 1.0000 |
| factor(X25.CS)=1 | 6.51 | 12 | 0 | 1 | 0.9375 | 0.8261 | 1.0000 |
| factor(X25.CS)=1 | 7 | 11 | 0 | 2 | 0.9375 | 0.8261 | 1.0000 |
| factor(X25.CS)=1 | 7.12 | 9 | 0 | 1 | 0.9375 | 0.8261 | 1.0000 |
| factor(X25.CS)=1 | 7.27 | 8 | 0 | 1 | 0.9375 | 0.8261 | 1.0000 |
| factor(X25.CS)=1 | 7.46 | 7 | 0 | 1 | 0.9375 | 0.8261 | 1.0000 |
| factor(X25.CS)=1 | 7.72 | 6 | 0 | 1 | 0.9375 | 0.8261 | 1.0000 |
| factor(X25.CS)=1 | 7.74 | 5 | 0 | 2 | 0.9375 | 0.8261 | 1.0000 |
| factor(X25.CS)=1 | 8.1 | 3 | 0 | 1 | 0.9375 | 0.8261 | 1.0000 |
| factor(X25.CS)=1 | 8.15 | 2 | 0 | 1 | 0.9375 | 0.8261 | 1.0000 |
| factor(X25.CS)=1 | 8.69 | 1 | 0 | 1 | 0.9375 | 0.8261 | 1.0000 |

|  |  |  |  |  |  |  |  |  |  |
| --- | --- | --- | --- | --- | --- | --- | --- | --- | --- |
| X25.CS | records | n.max | n.start | events | \*rmean | \*se(rmean) | median | 0.95LCL | 0.95UCL |
| factor(X25.CS)=0 | 318 | 318 | 318 | 7 | 9.167 | 0.061 | NA | NA | NA |
| factor(X25.CS)=1 | 19 | 19 | 19 | 1 | 9.101 | 0.212 | NA | NA | NA |

Created by EmpowerStats (www.empowerstats.com) and R on 2025-10-07
