# Supplementary material for: Optimal minimal residual disease threshold in pediatric acute myeloid leukemia: A retrospective cohort study based on the TARGET database
Source: PLoS Med. 2026 May 8;23(5):e1005088. doi: 10.1371/journal.pmed.1005088 (PMC13155632; doi:10.1371/journal.pmed.1005088)
Supplement: S1 Code — (ZIP) [file pmed.1005088.s002.zip › S2 code/PROJ8_12_tbl1/PROJ8_12_tbl1.htm]

## Kaplan Meier Survival Curve

Outcome: First Event
Time: EFS(years)
Landmark analysis for time segment: EFS(years) <= 5
Log rank test: implements the G-rho family of Harrington and Fleming (1982), with weights on each death of S(t)^rho, where S is the Kaplan-Meier estimate of survival. With rho = 0 this is the log-rank or Mantel-Haenszel test.

|  |  |  |  |
| --- | --- | --- | --- |
|  | N | Observed | Expected |
| factor(X23.CS)=0 | 418 | 121 | 134.9231 |
| factor(X23.CS)=1 | 65 | 32 | 18.0769 |

Chisq=
12.1929
on
1
degree of freedom, p=
0.0005
Survival table

|  |  |  |  |  |  |  |  |
| --- | --- | --- | --- | --- | --- | --- | --- |
|  | EFS(years) | N.Risk | N.Event | N.Censor | Survival | 95%CI Low | 95%CI Upp |
| factor(X23.CS)=0 | 0.19 | 418 | 1 | 0 | 0.9976 | 0.9929 | 1.0000 |
| factor(X23.CS)=0 | 0.23 | 417 | 2 | 0 | 0.9928 | 0.9848 | 1.0000 |
| factor(X23.CS)=0 | 0.26 | 415 | 2 | 0 | 0.9880 | 0.9777 | 0.9985 |
| factor(X23.CS)=0 | 0.29 | 413 | 2 | 0 | 0.9833 | 0.9710 | 0.9956 |
| factor(X23.CS)=0 | 0.32 | 411 | 1 | 0 | 0.9809 | 0.9678 | 0.9941 |
| factor(X23.CS)=0 | 0.42 | 410 | 1 | 0 | 0.9785 | 0.9647 | 0.9925 |
| factor(X23.CS)=0 | 0.47 | 409 | 2 | 0 | 0.9737 | 0.9585 | 0.9892 |
| factor(X23.CS)=0 | 0.5 | 407 | 1 | 0 | 0.9713 | 0.9554 | 0.9874 |
| factor(X23.CS)=0 | 0.53 | 406 | 3 | 0 | 0.9641 | 0.9464 | 0.9821 |
| factor(X23.CS)=0 | 0.57 | 403 | 3 | 0 | 0.9569 | 0.9377 | 0.9766 |
| factor(X23.CS)=0 | 0.58 | 400 | 1 | 0 | 0.9545 | 0.9348 | 0.9747 |
| factor(X23.CS)=0 | 0.6 | 399 | 2 | 0 | 0.9498 | 0.9290 | 0.9709 |
| factor(X23.CS)=0 | 0.61 | 397 | 1 | 0 | 0.9474 | 0.9262 | 0.9690 |
| factor(X23.CS)=0 | 0.64 | 396 | 1 | 0 | 0.9450 | 0.9234 | 0.9671 |
| factor(X23.CS)=0 | 0.67 | 395 | 1 | 0 | 0.9426 | 0.9205 | 0.9652 |
| factor(X23.CS)=0 | 0.68 | 394 | 3 | 0 | 0.9354 | 0.9121 | 0.9593 |
| factor(X23.CS)=0 | 0.7 | 391 | 1 | 0 | 0.9330 | 0.9094 | 0.9573 |
| factor(X23.CS)=0 | 0.71 | 390 | 1 | 0 | 0.9306 | 0.9066 | 0.9553 |
| factor(X23.CS)=0 | 0.72 | 389 | 1 | 0 | 0.9282 | 0.9038 | 0.9533 |
| factor(X23.CS)=0 | 0.74 | 388 | 2 | 0 | 0.9234 | 0.8983 | 0.9493 |
| factor(X23.CS)=0 | 0.75 | 386 | 1 | 0 | 0.9211 | 0.8956 | 0.9473 |
| factor(X23.CS)=0 | 0.76 | 385 | 2 | 0 | 0.9163 | 0.8901 | 0.9432 |
| factor(X23.CS)=0 | 0.79 | 383 | 4 | 0 | 0.9067 | 0.8792 | 0.9350 |
| factor(X23.CS)=0 | 0.8 | 379 | 1 | 0 | 0.9043 | 0.8765 | 0.9330 |
| factor(X23.CS)=0 | 0.81 | 378 | 2 | 0 | 0.8995 | 0.8712 | 0.9288 |
| factor(X23.CS)=0 | 0.82 | 376 | 4 | 0 | 0.8900 | 0.8605 | 0.9205 |
| factor(X23.CS)=0 | 0.84 | 372 | 2 | 0 | 0.8852 | 0.8551 | 0.9163 |
| factor(X23.CS)=0 | 0.86 | 370 | 1 | 0 | 0.8828 | 0.8525 | 0.9142 |
| factor(X23.CS)=0 | 0.87 | 369 | 1 | 0 | 0.8804 | 0.8498 | 0.9120 |
| factor(X23.CS)=0 | 0.88 | 368 | 3 | 0 | 0.8732 | 0.8419 | 0.9057 |
| factor(X23.CS)=0 | 0.9 | 365 | 1 | 0 | 0.8708 | 0.8392 | 0.9036 |
| factor(X23.CS)=0 | 0.91 | 364 | 1 | 0 | 0.8684 | 0.8366 | 0.9014 |
| factor(X23.CS)=0 | 0.93 | 363 | 2 | 0 | 0.8636 | 0.8314 | 0.8972 |
| factor(X23.CS)=0 | 0.94 | 361 | 2 | 0 | 0.8589 | 0.8261 | 0.8929 |
| factor(X23.CS)=0 | 0.96 | 359 | 2 | 0 | 0.8541 | 0.8209 | 0.8886 |
| factor(X23.CS)=0 | 0.97 | 357 | 1 | 0 | 0.8517 | 0.8183 | 0.8864 |
| factor(X23.CS)=0 | 0.99 | 356 | 1 | 1 | 0.8493 | 0.8157 | 0.8843 |
| factor(X23.CS)=0 | 1 | 354 | 1 | 0 | 0.8469 | 0.8131 | 0.8821 |
| factor(X23.CS)=0 | 1.01 | 353 | 1 | 0 | 0.8445 | 0.8104 | 0.8800 |
| factor(X23.CS)=0 | 1.02 | 352 | 1 | 1 | 0.8421 | 0.8078 | 0.8778 |
| factor(X23.CS)=0 | 1.03 | 350 | 1 | 0 | 0.8397 | 0.8052 | 0.8756 |
| factor(X23.CS)=0 | 1.05 | 349 | 3 | 0 | 0.8325 | 0.7974 | 0.8691 |
| factor(X23.CS)=0 | 1.06 | 346 | 2 | 1 | 0.8276 | 0.7922 | 0.8647 |
| factor(X23.CS)=0 | 1.07 | 343 | 1 | 0 | 0.8252 | 0.7896 | 0.8625 |
| factor(X23.CS)=0 | 1.08 | 342 | 1 | 0 | 0.8228 | 0.7870 | 0.8603 |
| factor(X23.CS)=0 | 1.09 | 341 | 1 | 0 | 0.8204 | 0.7844 | 0.8581 |
| factor(X23.CS)=0 | 1.1 | 340 | 1 | 0 | 0.8180 | 0.7818 | 0.8559 |
| factor(X23.CS)=0 | 1.12 | 339 | 1 | 1 | 0.8156 | 0.7792 | 0.8536 |
| factor(X23.CS)=0 | 1.13 | 337 | 2 | 0 | 0.8107 | 0.7740 | 0.8492 |
| factor(X23.CS)=0 | 1.14 | 335 | 0 | 1 | 0.8107 | 0.7740 | 0.8492 |
| factor(X23.CS)=0 | 1.15 | 334 | 1 | 0 | 0.8083 | 0.7714 | 0.8470 |
| factor(X23.CS)=0 | 1.17 | 333 | 1 | 0 | 0.8059 | 0.7688 | 0.8448 |
| factor(X23.CS)=0 | 1.2 | 332 | 2 | 0 | 0.8010 | 0.7636 | 0.8403 |
| factor(X23.CS)=0 | 1.21 | 330 | 1 | 0 | 0.7986 | 0.7610 | 0.8380 |
| factor(X23.CS)=0 | 1.24 | 329 | 1 | 0 | 0.7962 | 0.7584 | 0.8358 |
| factor(X23.CS)=0 | 1.27 | 328 | 2 | 0 | 0.7913 | 0.7533 | 0.8313 |
| factor(X23.CS)=0 | 1.28 | 326 | 1 | 1 | 0.7889 | 0.7507 | 0.8291 |
| factor(X23.CS)=0 | 1.3 | 324 | 1 | 0 | 0.7865 | 0.7481 | 0.8268 |
| factor(X23.CS)=0 | 1.32 | 323 | 1 | 0 | 0.7840 | 0.7455 | 0.8246 |
| factor(X23.CS)=0 | 1.34 | 322 | 0 | 1 | 0.7840 | 0.7455 | 0.8246 |
| factor(X23.CS)=0 | 1.37 | 321 | 1 | 0 | 0.7816 | 0.7429 | 0.8223 |
| factor(X23.CS)=0 | 1.42 | 320 | 1 | 0 | 0.7791 | 0.7403 | 0.8200 |
| factor(X23.CS)=0 | 1.43 | 319 | 1 | 0 | 0.7767 | 0.7377 | 0.8178 |
| factor(X23.CS)=0 | 1.48 | 318 | 2 | 0 | 0.7718 | 0.7325 | 0.8132 |
| factor(X23.CS)=0 | 1.49 | 316 | 2 | 0 | 0.7669 | 0.7274 | 0.8087 |
| factor(X23.CS)=0 | 1.54 | 314 | 1 | 0 | 0.7645 | 0.7248 | 0.8064 |
| factor(X23.CS)=0 | 1.55 | 313 | 1 | 0 | 0.7620 | 0.7222 | 0.8041 |
| factor(X23.CS)=0 | 1.58 | 312 | 1 | 0 | 0.7596 | 0.7196 | 0.8018 |
| factor(X23.CS)=0 | 1.65 | 311 | 2 | 0 | 0.7547 | 0.7145 | 0.7973 |
| factor(X23.CS)=0 | 1.66 | 309 | 1 | 0 | 0.7523 | 0.7119 | 0.7950 |
| factor(X23.CS)=0 | 1.67 | 308 | 1 | 0 | 0.7498 | 0.7093 | 0.7927 |
| factor(X23.CS)=0 | 1.71 | 307 | 0 | 1 | 0.7498 | 0.7093 | 0.7927 |
| factor(X23.CS)=0 | 1.72 | 306 | 1 | 0 | 0.7474 | 0.7067 | 0.7904 |
| factor(X23.CS)=0 | 1.78 | 305 | 1 | 0 | 0.7449 | 0.7042 | 0.7881 |
| factor(X23.CS)=0 | 1.81 | 304 | 1 | 0 | 0.7425 | 0.7016 | 0.7858 |
| factor(X23.CS)=0 | 1.87 | 303 | 1 | 1 | 0.7400 | 0.6990 | 0.7835 |
| factor(X23.CS)=0 | 1.92 | 301 | 0 | 1 | 0.7400 | 0.6990 | 0.7835 |
| factor(X23.CS)=0 | 1.94 | 300 | 1 | 2 | 0.7376 | 0.6964 | 0.7811 |
| factor(X23.CS)=0 | 1.96 | 297 | 0 | 1 | 0.7376 | 0.6964 | 0.7811 |
| factor(X23.CS)=0 | 1.98 | 296 | 0 | 1 | 0.7376 | 0.6964 | 0.7811 |
| factor(X23.CS)=0 | 1.99 | 295 | 1 | 0 | 0.7351 | 0.6938 | 0.7788 |
| factor(X23.CS)=0 | 2.03 | 294 | 1 | 0 | 0.7326 | 0.6912 | 0.7764 |
| factor(X23.CS)=0 | 2.09 | 293 | 1 | 0 | 0.7301 | 0.6885 | 0.7741 |
| factor(X23.CS)=0 | 2.1 | 292 | 0 | 1 | 0.7301 | 0.6885 | 0.7741 |
| factor(X23.CS)=0 | 2.22 | 291 | 0 | 1 | 0.7301 | 0.6885 | 0.7741 |
| factor(X23.CS)=0 | 2.23 | 290 | 1 | 0 | 0.7275 | 0.6859 | 0.7717 |
| factor(X23.CS)=0 | 2.27 | 289 | 0 | 2 | 0.7275 | 0.6859 | 0.7717 |
| factor(X23.CS)=0 | 2.3 | 287 | 1 | 0 | 0.7250 | 0.6832 | 0.7693 |
| factor(X23.CS)=0 | 2.31 | 286 | 1 | 0 | 0.7225 | 0.6806 | 0.7670 |
| factor(X23.CS)=0 | 2.32 | 285 | 0 | 1 | 0.7225 | 0.6806 | 0.7670 |
| factor(X23.CS)=0 | 2.37 | 284 | 0 | 1 | 0.7225 | 0.6806 | 0.7670 |
| factor(X23.CS)=0 | 2.39 | 283 | 0 | 1 | 0.7225 | 0.6806 | 0.7670 |
| factor(X23.CS)=0 | 2.4 | 282 | 0 | 1 | 0.7225 | 0.6806 | 0.7670 |
| factor(X23.CS)=0 | 2.41 | 281 | 0 | 1 | 0.7225 | 0.6806 | 0.7670 |
| factor(X23.CS)=0 | 2.42 | 280 | 1 | 0 | 0.7199 | 0.6779 | 0.7645 |
| factor(X23.CS)=0 | 2.44 | 279 | 0 | 2 | 0.7199 | 0.6779 | 0.7645 |
| factor(X23.CS)=0 | 2.47 | 277 | 0 | 1 | 0.7199 | 0.6779 | 0.7645 |
| factor(X23.CS)=0 | 2.48 | 276 | 1 | 0 | 0.7173 | 0.6751 | 0.7621 |
| factor(X23.CS)=0 | 2.5 | 275 | 0 | 2 | 0.7173 | 0.6751 | 0.7621 |
| factor(X23.CS)=0 | 2.51 | 273 | 1 | 0 | 0.7147 | 0.6724 | 0.7596 |
| factor(X23.CS)=0 | 2.52 | 272 | 1 | 0 | 0.7120 | 0.6696 | 0.7571 |
| factor(X23.CS)=0 | 2.53 | 271 | 0 | 1 | 0.7120 | 0.6696 | 0.7571 |
| factor(X23.CS)=0 | 2.55 | 270 | 0 | 1 | 0.7120 | 0.6696 | 0.7571 |
| factor(X23.CS)=0 | 2.64 | 269 | 0 | 1 | 0.7120 | 0.6696 | 0.7571 |
| factor(X23.CS)=0 | 2.68 | 268 | 0 | 1 | 0.7120 | 0.6696 | 0.7571 |
| factor(X23.CS)=0 | 2.7 | 267 | 1 | 1 | 0.7094 | 0.6668 | 0.7546 |
| factor(X23.CS)=0 | 2.75 | 265 | 0 | 1 | 0.7094 | 0.6668 | 0.7546 |
| factor(X23.CS)=0 | 2.79 | 264 | 0 | 1 | 0.7094 | 0.6668 | 0.7546 |
| factor(X23.CS)=0 | 2.83 | 263 | 0 | 1 | 0.7094 | 0.6668 | 0.7546 |
| factor(X23.CS)=0 | 2.93 | 262 | 0 | 1 | 0.7094 | 0.6668 | 0.7546 |
| factor(X23.CS)=0 | 3.02 | 261 | 0 | 1 | 0.7094 | 0.6668 | 0.7546 |
| factor(X23.CS)=0 | 3.03 | 260 | 0 | 1 | 0.7094 | 0.6668 | 0.7546 |
| factor(X23.CS)=0 | 3.11 | 259 | 0 | 1 | 0.7094 | 0.6668 | 0.7546 |
| factor(X23.CS)=0 | 3.14 | 258 | 0 | 1 | 0.7094 | 0.6668 | 0.7546 |
| factor(X23.CS)=0 | 3.15 | 257 | 0 | 1 | 0.7094 | 0.6668 | 0.7546 |
| factor(X23.CS)=0 | 3.16 | 256 | 0 | 1 | 0.7094 | 0.6668 | 0.7546 |
| factor(X23.CS)=0 | 3.17 | 255 | 0 | 1 | 0.7094 | 0.6668 | 0.7546 |
| factor(X23.CS)=0 | 3.19 | 254 | 0 | 1 | 0.7094 | 0.6668 | 0.7546 |
| factor(X23.CS)=0 | 3.2 | 253 | 0 | 1 | 0.7094 | 0.6668 | 0.7546 |
| factor(X23.CS)=0 | 3.22 | 252 | 0 | 1 | 0.7094 | 0.6668 | 0.7546 |
| factor(X23.CS)=0 | 3.23 | 251 | 0 | 1 | 0.7094 | 0.6668 | 0.7546 |
| factor(X23.CS)=0 | 3.24 | 250 | 0 | 1 | 0.7094 | 0.6668 | 0.7546 |
| factor(X23.CS)=0 | 3.28 | 249 | 0 | 1 | 0.7094 | 0.6668 | 0.7546 |
| factor(X23.CS)=0 | 3.3 | 248 | 0 | 1 | 0.7094 | 0.6668 | 0.7546 |
| factor(X23.CS)=0 | 3.32 | 247 | 0 | 1 | 0.7094 | 0.6668 | 0.7546 |
| factor(X23.CS)=0 | 3.33 | 246 | 0 | 2 | 0.7094 | 0.6668 | 0.7546 |
| factor(X23.CS)=0 | 3.34 | 244 | 0 | 2 | 0.7094 | 0.6668 | 0.7546 |
| factor(X23.CS)=0 | 3.35 | 242 | 0 | 1 | 0.7094 | 0.6668 | 0.7546 |
| factor(X23.CS)=0 | 3.36 | 241 | 0 | 1 | 0.7094 | 0.6668 | 0.7546 |
| factor(X23.CS)=0 | 3.38 | 240 | 0 | 1 | 0.7094 | 0.6668 | 0.7546 |
| factor(X23.CS)=0 | 3.41 | 239 | 0 | 1 | 0.7094 | 0.6668 | 0.7546 |
| factor(X23.CS)=0 | 3.43 | 238 | 0 | 1 | 0.7094 | 0.6668 | 0.7546 |
| factor(X23.CS)=0 | 3.44 | 237 | 0 | 2 | 0.7094 | 0.6668 | 0.7546 |
| factor(X23.CS)=0 | 3.45 | 235 | 0 | 1 | 0.7094 | 0.6668 | 0.7546 |
| factor(X23.CS)=0 | 3.5 | 234 | 0 | 2 | 0.7094 | 0.6668 | 0.7546 |
| factor(X23.CS)=0 | 3.52 | 232 | 0 | 1 | 0.7094 | 0.6668 | 0.7546 |
| factor(X23.CS)=0 | 3.54 | 231 | 1 | 1 | 0.7063 | 0.6635 | 0.7518 |
| factor(X23.CS)=0 | 3.56 | 229 | 0 | 3 | 0.7063 | 0.6635 | 0.7518 |
| factor(X23.CS)=0 | 3.58 | 226 | 0 | 1 | 0.7063 | 0.6635 | 0.7518 |
| factor(X23.CS)=0 | 3.65 | 225 | 0 | 2 | 0.7063 | 0.6635 | 0.7518 |
| factor(X23.CS)=0 | 3.66 | 223 | 0 | 1 | 0.7063 | 0.6635 | 0.7518 |
| factor(X23.CS)=0 | 3.79 | 222 | 0 | 1 | 0.7063 | 0.6635 | 0.7518 |
| factor(X23.CS)=0 | 3.81 | 221 | 0 | 1 | 0.7063 | 0.6635 | 0.7518 |
| factor(X23.CS)=0 | 3.83 | 220 | 0 | 1 | 0.7063 | 0.6635 | 0.7518 |
| factor(X23.CS)=0 | 3.9 | 219 | 0 | 1 | 0.7063 | 0.6635 | 0.7518 |
| factor(X23.CS)=0 | 3.95 | 218 | 0 | 1 | 0.7063 | 0.6635 | 0.7518 |
| factor(X23.CS)=0 | 4.05 | 217 | 0 | 1 | 0.7063 | 0.6635 | 0.7518 |
| factor(X23.CS)=0 | 4.06 | 216 | 0 | 2 | 0.7063 | 0.6635 | 0.7518 |
| factor(X23.CS)=0 | 4.07 | 214 | 0 | 1 | 0.7063 | 0.6635 | 0.7518 |
| factor(X23.CS)=0 | 4.08 | 213 | 0 | 1 | 0.7063 | 0.6635 | 0.7518 |
| factor(X23.CS)=0 | 4.13 | 212 | 0 | 2 | 0.7063 | 0.6635 | 0.7518 |
| factor(X23.CS)=0 | 4.15 | 210 | 0 | 2 | 0.7063 | 0.6635 | 0.7518 |
| factor(X23.CS)=0 | 4.26 | 208 | 0 | 1 | 0.7063 | 0.6635 | 0.7518 |
| factor(X23.CS)=0 | 4.27 | 207 | 0 | 1 | 0.7063 | 0.6635 | 0.7518 |
| factor(X23.CS)=0 | 4.31 | 206 | 0 | 1 | 0.7063 | 0.6635 | 0.7518 |
| factor(X23.CS)=0 | 4.32 | 205 | 0 | 1 | 0.7063 | 0.6635 | 0.7518 |
| factor(X23.CS)=0 | 4.35 | 204 | 0 | 3 | 0.7063 | 0.6635 | 0.7518 |
| factor(X23.CS)=0 | 4.36 | 201 | 0 | 3 | 0.7063 | 0.6635 | 0.7518 |
| factor(X23.CS)=0 | 4.38 | 198 | 0 | 1 | 0.7063 | 0.6635 | 0.7518 |
| factor(X23.CS)=0 | 4.4 | 197 | 0 | 1 | 0.7063 | 0.6635 | 0.7518 |
| factor(X23.CS)=0 | 4.42 | 196 | 0 | 1 | 0.7063 | 0.6635 | 0.7518 |
| factor(X23.CS)=0 | 4.44 | 195 | 0 | 1 | 0.7063 | 0.6635 | 0.7518 |
| factor(X23.CS)=0 | 4.54 | 194 | 0 | 1 | 0.7063 | 0.6635 | 0.7518 |
| factor(X23.CS)=0 | 4.59 | 193 | 0 | 1 | 0.7063 | 0.6635 | 0.7518 |
| factor(X23.CS)=0 | 4.6 | 192 | 0 | 1 | 0.7063 | 0.6635 | 0.7518 |
| factor(X23.CS)=0 | 4.61 | 191 | 0 | 1 | 0.7063 | 0.6635 | 0.7518 |
| factor(X23.CS)=0 | 4.64 | 190 | 0 | 1 | 0.7063 | 0.6635 | 0.7518 |
| factor(X23.CS)=0 | 4.72 | 189 | 0 | 1 | 0.7063 | 0.6635 | 0.7518 |
| factor(X23.CS)=0 | 4.73 | 188 | 0 | 1 | 0.7063 | 0.6635 | 0.7518 |
| factor(X23.CS)=0 | 4.75 | 187 | 0 | 1 | 0.7063 | 0.6635 | 0.7518 |
| factor(X23.CS)=0 | 4.81 | 186 | 0 | 1 | 0.7063 | 0.6635 | 0.7518 |
| factor(X23.CS)=0 | 4.82 | 185 | 0 | 1 | 0.7063 | 0.6635 | 0.7518 |
| factor(X23.CS)=0 | 4.85 | 184 | 0 | 1 | 0.7063 | 0.6635 | 0.7518 |
| factor(X23.CS)=0 | 4.91 | 183 | 0 | 1 | 0.7063 | 0.6635 | 0.7518 |
| factor(X23.CS)=0 | 4.94 | 182 | 0 | 2 | 0.7063 | 0.6635 | 0.7518 |
| factor(X23.CS)=0 | 4.97 | 180 | 0 | 1 | 0.7063 | 0.6635 | 0.7518 |
| factor(X23.CS)=0 | 5 | 179 | 0 | 179 | 0.7063 | 0.6635 | 0.7518 |
| factor(X23.CS)=1 | 0.18 | 65 | 1 | 0 | 0.9846 | 0.9551 | 1.0000 |
| factor(X23.CS)=1 | 0.23 | 64 | 1 | 0 | 0.9692 | 0.9281 | 1.0000 |
| factor(X23.CS)=1 | 0.24 | 63 | 1 | 0 | 0.9538 | 0.9042 | 1.0000 |
| factor(X23.CS)=1 | 0.41 | 62 | 2 | 0 | 0.9231 | 0.8605 | 0.9902 |
| factor(X23.CS)=1 | 0.47 | 60 | 1 | 0 | 0.9077 | 0.8400 | 0.9809 |
| factor(X23.CS)=1 | 0.52 | 59 | 1 | 0 | 0.8923 | 0.8200 | 0.9709 |
| factor(X23.CS)=1 | 0.66 | 58 | 1 | 0 | 0.8769 | 0.8006 | 0.9605 |
| factor(X23.CS)=1 | 0.71 | 57 | 1 | 0 | 0.8615 | 0.7815 | 0.9497 |
| factor(X23.CS)=1 | 0.72 | 56 | 1 | 0 | 0.8462 | 0.7628 | 0.9386 |
| factor(X23.CS)=1 | 0.75 | 55 | 1 | 0 | 0.8308 | 0.7444 | 0.9271 |
| factor(X23.CS)=1 | 0.78 | 54 | 1 | 0 | 0.8154 | 0.7263 | 0.9154 |
| factor(X23.CS)=1 | 0.82 | 53 | 1 | 0 | 0.8000 | 0.7084 | 0.9034 |
| factor(X23.CS)=1 | 0.85 | 52 | 1 | 0 | 0.7846 | 0.6908 | 0.8912 |
| factor(X23.CS)=1 | 0.86 | 51 | 1 | 1 | 0.7692 | 0.6733 | 0.8788 |
| factor(X23.CS)=1 | 0.87 | 49 | 1 | 0 | 0.7535 | 0.6556 | 0.8660 |
| factor(X23.CS)=1 | 0.93 | 48 | 1 | 0 | 0.7378 | 0.6382 | 0.8531 |
| factor(X23.CS)=1 | 0.97 | 47 | 1 | 0 | 0.7221 | 0.6208 | 0.8400 |
| factor(X23.CS)=1 | 1 | 46 | 1 | 0 | 0.7064 | 0.6037 | 0.8267 |
| factor(X23.CS)=1 | 1.08 | 45 | 1 | 0 | 0.6907 | 0.5867 | 0.8132 |
| factor(X23.CS)=1 | 1.11 | 44 | 1 | 0 | 0.6750 | 0.5699 | 0.7996 |
| factor(X23.CS)=1 | 1.12 | 43 | 1 | 0 | 0.6593 | 0.5532 | 0.7858 |
| factor(X23.CS)=1 | 1.16 | 42 | 1 | 0 | 0.6436 | 0.5367 | 0.7719 |
| factor(X23.CS)=1 | 1.35 | 41 | 1 | 0 | 0.6279 | 0.5203 | 0.7579 |
| factor(X23.CS)=1 | 1.36 | 40 | 1 | 0 | 0.6122 | 0.5040 | 0.7437 |
| factor(X23.CS)=1 | 1.5 | 39 | 1 | 0 | 0.5965 | 0.4879 | 0.7294 |
| factor(X23.CS)=1 | 1.79 | 38 | 1 | 0 | 0.5808 | 0.4719 | 0.7150 |
| factor(X23.CS)=1 | 1.92 | 37 | 1 | 0 | 0.5651 | 0.4560 | 0.7004 |
| factor(X23.CS)=1 | 2.09 | 36 | 1 | 0 | 0.5495 | 0.4403 | 0.6857 |
| factor(X23.CS)=1 | 2.32 | 35 | 0 | 1 | 0.5495 | 0.4403 | 0.6857 |
| factor(X23.CS)=1 | 2.95 | 34 | 0 | 1 | 0.5495 | 0.4403 | 0.6857 |
| factor(X23.CS)=1 | 2.99 | 33 | 1 | 0 | 0.5328 | 0.4235 | 0.6703 |
| factor(X23.CS)=1 | 3.25 | 32 | 0 | 1 | 0.5328 | 0.4235 | 0.6703 |
| factor(X23.CS)=1 | 3.39 | 31 | 0 | 1 | 0.5328 | 0.4235 | 0.6703 |
| factor(X23.CS)=1 | 4.16 | 30 | 0 | 1 | 0.5328 | 0.4235 | 0.6703 |
| factor(X23.CS)=1 | 4.41 | 29 | 0 | 1 | 0.5328 | 0.4235 | 0.6703 |
| factor(X23.CS)=1 | 4.47 | 28 | 0 | 1 | 0.5328 | 0.4235 | 0.6703 |
| factor(X23.CS)=1 | 4.48 | 27 | 0 | 1 | 0.5328 | 0.4235 | 0.6703 |
| factor(X23.CS)=1 | 4.55 | 26 | 1 | 0 | 0.5123 | 0.4021 | 0.6527 |
| factor(X23.CS)=1 | 4.68 | 25 | 1 | 0 | 0.4918 | 0.3811 | 0.6347 |
| factor(X23.CS)=1 | 4.89 | 24 | 0 | 1 | 0.4918 | 0.3811 | 0.6347 |
| factor(X23.CS)=1 | 5 | 23 | 0 | 23 | 0.4918 | 0.3811 | 0.6347 |

|  |  |  |  |  |  |  |  |  |  |
| --- | --- | --- | --- | --- | --- | --- | --- | --- | --- |
| X23.CS | records | n.max | n.start | events | \*rmean | \*se(rmean) | median | 0.95LCL | 0.95UCL |
| factor(X23.CS)=0 | 418 | 418 | 418 | 121 | 3.86 | 0.088 | NA | NA | NA |
| factor(X23.CS)=1 | 65 | 65 | 65 | 32 | 3.117 | 0.252 | 4.68 | 1.5 | NA |

Landmark analysis for time segment: EFS(years) > 5
Log rank test: implements the G-rho family of Harrington and Fleming (1982), with weights on each death of S(t)^rho, where S is the Kaplan-Meier estimate of survival. With rho = 0 this is the log-rank or Mantel-Haenszel test.

|  |  |  |  |
| --- | --- | --- | --- |
|  | N | Observed | Expected |
| factor(X23.CS)=0 | 178 | 1 | 2.5760 |
| factor(X23.CS)=1 | 23 | 2 | 0.4240 |

Chisq=
6.8591
on
1
degree of freedom, p=
0.0088
Survival table

|  |  |  |  |  |  |  |  |
| --- | --- | --- | --- | --- | --- | --- | --- |
|  | EFS(years) | N.Risk | N.Event | N.Censor | Survival | 95%CI Low | 95%CI Upp |
| factor(X23.CS)=0 | 5.03 | 178 | 0 | 1 | 1.0000 | 1.0000 | 1.0000 |
| factor(X23.CS)=0 | 5.05 | 177 | 0 | 1 | 1.0000 | 1.0000 | 1.0000 |
| factor(X23.CS)=0 | 5.08 | 176 | 0 | 1 | 1.0000 | 1.0000 | 1.0000 |
| factor(X23.CS)=0 | 5.1 | 175 | 0 | 1 | 1.0000 | 1.0000 | 1.0000 |
| factor(X23.CS)=0 | 5.12 | 174 | 0 | 1 | 1.0000 | 1.0000 | 1.0000 |
| factor(X23.CS)=0 | 5.15 | 173 | 0 | 2 | 1.0000 | 1.0000 | 1.0000 |
| factor(X23.CS)=0 | 5.16 | 171 | 0 | 1 | 1.0000 | 1.0000 | 1.0000 |
| factor(X23.CS)=0 | 5.18 | 170 | 0 | 2 | 1.0000 | 1.0000 | 1.0000 |
| factor(X23.CS)=0 | 5.22 | 168 | 0 | 1 | 1.0000 | 1.0000 | 1.0000 |
| factor(X23.CS)=0 | 5.25 | 167 | 0 | 2 | 1.0000 | 1.0000 | 1.0000 |
| factor(X23.CS)=0 | 5.26 | 165 | 0 | 1 | 1.0000 | 1.0000 | 1.0000 |
| factor(X23.CS)=0 | 5.29 | 164 | 0 | 2 | 1.0000 | 1.0000 | 1.0000 |
| factor(X23.CS)=0 | 5.31 | 162 | 0 | 1 | 1.0000 | 1.0000 | 1.0000 |
| factor(X23.CS)=0 | 5.32 | 161 | 0 | 1 | 1.0000 | 1.0000 | 1.0000 |
| factor(X23.CS)=0 | 5.33 | 160 | 0 | 1 | 1.0000 | 1.0000 | 1.0000 |
| factor(X23.CS)=0 | 5.34 | 159 | 0 | 1 | 1.0000 | 1.0000 | 1.0000 |
| factor(X23.CS)=0 | 5.35 | 158 | 0 | 4 | 1.0000 | 1.0000 | 1.0000 |
| factor(X23.CS)=0 | 5.36 | 154 | 0 | 1 | 1.0000 | 1.0000 | 1.0000 |
| factor(X23.CS)=0 | 5.38 | 153 | 0 | 1 | 1.0000 | 1.0000 | 1.0000 |
| factor(X23.CS)=0 | 5.39 | 152 | 0 | 1 | 1.0000 | 1.0000 | 1.0000 |
| factor(X23.CS)=0 | 5.4 | 151 | 0 | 1 | 1.0000 | 1.0000 | 1.0000 |
| factor(X23.CS)=0 | 5.41 | 150 | 0 | 1 | 1.0000 | 1.0000 | 1.0000 |
| factor(X23.CS)=0 | 5.42 | 149 | 0 | 3 | 1.0000 | 1.0000 | 1.0000 |
| factor(X23.CS)=0 | 5.43 | 146 | 0 | 1 | 1.0000 | 1.0000 | 1.0000 |
| factor(X23.CS)=0 | 5.44 | 145 | 0 | 2 | 1.0000 | 1.0000 | 1.0000 |
| factor(X23.CS)=0 | 5.45 | 143 | 0 | 1 | 1.0000 | 1.0000 | 1.0000 |
| factor(X23.CS)=0 | 5.46 | 142 | 0 | 1 | 1.0000 | 1.0000 | 1.0000 |
| factor(X23.CS)=0 | 5.48 | 141 | 0 | 2 | 1.0000 | 1.0000 | 1.0000 |
| factor(X23.CS)=0 | 5.49 | 139 | 0 | 1 | 1.0000 | 1.0000 | 1.0000 |
| factor(X23.CS)=0 | 5.5 | 138 | 0 | 1 | 1.0000 | 1.0000 | 1.0000 |
| factor(X23.CS)=0 | 5.51 | 137 | 0 | 2 | 1.0000 | 1.0000 | 1.0000 |
| factor(X23.CS)=0 | 5.52 | 135 | 0 | 1 | 1.0000 | 1.0000 | 1.0000 |
| factor(X23.CS)=0 | 5.54 | 134 | 0 | 2 | 1.0000 | 1.0000 | 1.0000 |
| factor(X23.CS)=0 | 5.55 | 132 | 0 | 1 | 1.0000 | 1.0000 | 1.0000 |
| factor(X23.CS)=0 | 5.56 | 131 | 0 | 2 | 1.0000 | 1.0000 | 1.0000 |
| factor(X23.CS)=0 | 5.58 | 129 | 0 | 1 | 1.0000 | 1.0000 | 1.0000 |
| factor(X23.CS)=0 | 5.63 | 128 | 0 | 1 | 1.0000 | 1.0000 | 1.0000 |
| factor(X23.CS)=0 | 5.65 | 127 | 0 | 1 | 1.0000 | 1.0000 | 1.0000 |
| factor(X23.CS)=0 | 5.66 | 126 | 0 | 1 | 1.0000 | 1.0000 | 1.0000 |
| factor(X23.CS)=0 | 5.67 | 125 | 0 | 2 | 1.0000 | 1.0000 | 1.0000 |
| factor(X23.CS)=0 | 5.7 | 123 | 0 | 1 | 1.0000 | 1.0000 | 1.0000 |
| factor(X23.CS)=0 | 5.71 | 122 | 0 | 2 | 1.0000 | 1.0000 | 1.0000 |
| factor(X23.CS)=0 | 5.72 | 120 | 0 | 1 | 1.0000 | 1.0000 | 1.0000 |
| factor(X23.CS)=0 | 5.73 | 119 | 0 | 2 | 1.0000 | 1.0000 | 1.0000 |
| factor(X23.CS)=0 | 5.75 | 117 | 0 | 1 | 1.0000 | 1.0000 | 1.0000 |
| factor(X23.CS)=0 | 5.77 | 116 | 0 | 1 | 1.0000 | 1.0000 | 1.0000 |
| factor(X23.CS)=0 | 5.78 | 115 | 0 | 2 | 1.0000 | 1.0000 | 1.0000 |
| factor(X23.CS)=0 | 5.84 | 113 | 0 | 1 | 1.0000 | 1.0000 | 1.0000 |
| factor(X23.CS)=0 | 5.85 | 112 | 0 | 1 | 1.0000 | 1.0000 | 1.0000 |
| factor(X23.CS)=0 | 5.88 | 111 | 0 | 1 | 1.0000 | 1.0000 | 1.0000 |
| factor(X23.CS)=0 | 5.89 | 110 | 0 | 1 | 1.0000 | 1.0000 | 1.0000 |
| factor(X23.CS)=0 | 5.93 | 109 | 0 | 1 | 1.0000 | 1.0000 | 1.0000 |
| factor(X23.CS)=0 | 5.94 | 108 | 0 | 1 | 1.0000 | 1.0000 | 1.0000 |
| factor(X23.CS)=0 | 5.95 | 107 | 0 | 2 | 1.0000 | 1.0000 | 1.0000 |
| factor(X23.CS)=0 | 5.96 | 105 | 0 | 1 | 1.0000 | 1.0000 | 1.0000 |
| factor(X23.CS)=0 | 5.98 | 104 | 0 | 1 | 1.0000 | 1.0000 | 1.0000 |
| factor(X23.CS)=0 | 6 | 103 | 0 | 4 | 1.0000 | 1.0000 | 1.0000 |
| factor(X23.CS)=0 | 6.01 | 99 | 0 | 1 | 1.0000 | 1.0000 | 1.0000 |
| factor(X23.CS)=0 | 6.02 | 98 | 0 | 1 | 1.0000 | 1.0000 | 1.0000 |
| factor(X23.CS)=0 | 6.07 | 97 | 0 | 1 | 1.0000 | 1.0000 | 1.0000 |
| factor(X23.CS)=0 | 6.08 | 96 | 0 | 1 | 1.0000 | 1.0000 | 1.0000 |
| factor(X23.CS)=0 | 6.12 | 95 | 0 | 1 | 1.0000 | 1.0000 | 1.0000 |
| factor(X23.CS)=0 | 6.15 | 94 | 0 | 2 | 1.0000 | 1.0000 | 1.0000 |
| factor(X23.CS)=0 | 6.18 | 92 | 0 | 2 | 1.0000 | 1.0000 | 1.0000 |
| factor(X23.CS)=0 | 6.22 | 90 | 0 | 1 | 1.0000 | 1.0000 | 1.0000 |
| factor(X23.CS)=0 | 6.25 | 89 | 0 | 2 | 1.0000 | 1.0000 | 1.0000 |
| factor(X23.CS)=0 | 6.27 | 87 | 0 | 1 | 1.0000 | 1.0000 | 1.0000 |
| factor(X23.CS)=0 | 6.28 | 86 | 0 | 1 | 1.0000 | 1.0000 | 1.0000 |
| factor(X23.CS)=0 | 6.29 | 85 | 0 | 1 | 1.0000 | 1.0000 | 1.0000 |
| factor(X23.CS)=0 | 6.32 | 84 | 0 | 1 | 1.0000 | 1.0000 | 1.0000 |
| factor(X23.CS)=0 | 6.35 | 83 | 0 | 1 | 1.0000 | 1.0000 | 1.0000 |
| factor(X23.CS)=0 | 6.36 | 82 | 0 | 1 | 1.0000 | 1.0000 | 1.0000 |
| factor(X23.CS)=0 | 6.37 | 81 | 0 | 1 | 1.0000 | 1.0000 | 1.0000 |
| factor(X23.CS)=0 | 6.38 | 80 | 0 | 1 | 1.0000 | 1.0000 | 1.0000 |
| factor(X23.CS)=0 | 6.39 | 79 | 0 | 3 | 1.0000 | 1.0000 | 1.0000 |
| factor(X23.CS)=0 | 6.42 | 76 | 0 | 3 | 1.0000 | 1.0000 | 1.0000 |
| factor(X23.CS)=0 | 6.46 | 73 | 0 | 1 | 1.0000 | 1.0000 | 1.0000 |
| factor(X23.CS)=0 | 6.47 | 72 | 0 | 3 | 1.0000 | 1.0000 | 1.0000 |
| factor(X23.CS)=0 | 6.48 | 69 | 0 | 1 | 1.0000 | 1.0000 | 1.0000 |
| factor(X23.CS)=0 | 6.54 | 68 | 0 | 1 | 1.0000 | 1.0000 | 1.0000 |
| factor(X23.CS)=0 | 6.55 | 67 | 0 | 1 | 1.0000 | 1.0000 | 1.0000 |
| factor(X23.CS)=0 | 6.57 | 66 | 0 | 1 | 1.0000 | 1.0000 | 1.0000 |
| factor(X23.CS)=0 | 6.6 | 65 | 0 | 1 | 1.0000 | 1.0000 | 1.0000 |
| factor(X23.CS)=0 | 6.64 | 64 | 0 | 1 | 1.0000 | 1.0000 | 1.0000 |
| factor(X23.CS)=0 | 6.65 | 63 | 0 | 1 | 1.0000 | 1.0000 | 1.0000 |
| factor(X23.CS)=0 | 6.68 | 62 | 0 | 1 | 1.0000 | 1.0000 | 1.0000 |
| factor(X23.CS)=0 | 6.69 | 61 | 0 | 1 | 1.0000 | 1.0000 | 1.0000 |
| factor(X23.CS)=0 | 6.73 | 60 | 0 | 1 | 1.0000 | 1.0000 | 1.0000 |
| factor(X23.CS)=0 | 6.75 | 59 | 0 | 2 | 1.0000 | 1.0000 | 1.0000 |
| factor(X23.CS)=0 | 6.77 | 57 | 0 | 1 | 1.0000 | 1.0000 | 1.0000 |
| factor(X23.CS)=0 | 6.78 | 56 | 0 | 1 | 1.0000 | 1.0000 | 1.0000 |
| factor(X23.CS)=0 | 6.81 | 55 | 0 | 1 | 1.0000 | 1.0000 | 1.0000 |
| factor(X23.CS)=0 | 6.85 | 54 | 0 | 1 | 1.0000 | 1.0000 | 1.0000 |
| factor(X23.CS)=0 | 6.89 | 53 | 0 | 1 | 1.0000 | 1.0000 | 1.0000 |
| factor(X23.CS)=0 | 6.91 | 52 | 0 | 1 | 1.0000 | 1.0000 | 1.0000 |
| factor(X23.CS)=0 | 6.96 | 51 | 0 | 3 | 1.0000 | 1.0000 | 1.0000 |
| factor(X23.CS)=0 | 6.98 | 48 | 0 | 1 | 1.0000 | 1.0000 | 1.0000 |
| factor(X23.CS)=0 | 7.14 | 47 | 0 | 1 | 1.0000 | 1.0000 | 1.0000 |
| factor(X23.CS)=0 | 7.16 | 46 | 0 | 1 | 1.0000 | 1.0000 | 1.0000 |
| factor(X23.CS)=0 | 7.2 | 45 | 0 | 1 | 1.0000 | 1.0000 | 1.0000 |
| factor(X23.CS)=0 | 7.26 | 44 | 0 | 2 | 1.0000 | 1.0000 | 1.0000 |
| factor(X23.CS)=0 | 7.29 | 42 | 0 | 1 | 1.0000 | 1.0000 | 1.0000 |
| factor(X23.CS)=0 | 7.35 | 41 | 0 | 2 | 1.0000 | 1.0000 | 1.0000 |
| factor(X23.CS)=0 | 7.36 | 39 | 0 | 3 | 1.0000 | 1.0000 | 1.0000 |
| factor(X23.CS)=0 | 7.37 | 36 | 0 | 1 | 1.0000 | 1.0000 | 1.0000 |
| factor(X23.CS)=0 | 7.39 | 35 | 0 | 2 | 1.0000 | 1.0000 | 1.0000 |
| factor(X23.CS)=0 | 7.4 | 33 | 0 | 1 | 1.0000 | 1.0000 | 1.0000 |
| factor(X23.CS)=0 | 7.42 | 32 | 0 | 1 | 1.0000 | 1.0000 | 1.0000 |
| factor(X23.CS)=0 | 7.47 | 31 | 0 | 1 | 1.0000 | 1.0000 | 1.0000 |
| factor(X23.CS)=0 | 7.48 | 30 | 0 | 1 | 1.0000 | 1.0000 | 1.0000 |
| factor(X23.CS)=0 | 7.5 | 29 | 0 | 1 | 1.0000 | 1.0000 | 1.0000 |
| factor(X23.CS)=0 | 7.58 | 28 | 0 | 3 | 1.0000 | 1.0000 | 1.0000 |
| factor(X23.CS)=0 | 7.59 | 25 | 0 | 1 | 1.0000 | 1.0000 | 1.0000 |
| factor(X23.CS)=0 | 7.6 | 24 | 0 | 2 | 1.0000 | 1.0000 | 1.0000 |
| factor(X23.CS)=0 | 7.61 | 22 | 0 | 1 | 1.0000 | 1.0000 | 1.0000 |
| factor(X23.CS)=0 | 7.67 | 21 | 0 | 1 | 1.0000 | 1.0000 | 1.0000 |
| factor(X23.CS)=0 | 7.69 | 20 | 0 | 2 | 1.0000 | 1.0000 | 1.0000 |
| factor(X23.CS)=0 | 7.73 | 18 | 0 | 2 | 1.0000 | 1.0000 | 1.0000 |
| factor(X23.CS)=0 | 7.81 | 16 | 0 | 1 | 1.0000 | 1.0000 | 1.0000 |
| factor(X23.CS)=0 | 7.82 | 15 | 0 | 1 | 1.0000 | 1.0000 | 1.0000 |
| factor(X23.CS)=0 | 7.99 | 14 | 1 | 1 | 0.9286 | 0.8030 | 1.0000 |
| factor(X23.CS)=0 | 8.07 | 12 | 0 | 1 | 0.9286 | 0.8030 | 1.0000 |
| factor(X23.CS)=0 | 8.26 | 11 | 0 | 1 | 0.9286 | 0.8030 | 1.0000 |
| factor(X23.CS)=0 | 8.29 | 10 | 0 | 1 | 0.9286 | 0.8030 | 1.0000 |
| factor(X23.CS)=0 | 8.5 | 9 | 0 | 1 | 0.9286 | 0.8030 | 1.0000 |
| factor(X23.CS)=0 | 8.53 | 8 | 0 | 1 | 0.9286 | 0.8030 | 1.0000 |
| factor(X23.CS)=0 | 8.88 | 7 | 0 | 2 | 0.9286 | 0.8030 | 1.0000 |
| factor(X23.CS)=0 | 9.04 | 5 | 0 | 1 | 0.9286 | 0.8030 | 1.0000 |
| factor(X23.CS)=0 | 9.06 | 4 | 0 | 2 | 0.9286 | 0.8030 | 1.0000 |
| factor(X23.CS)=0 | 9.08 | 2 | 0 | 1 | 0.9286 | 0.8030 | 1.0000 |
| factor(X23.CS)=0 | 9.61 | 1 | 0 | 1 | 0.9286 | 0.8030 | 1.0000 |
| factor(X23.CS)=1 | 5.12 | 23 | 1 | 0 | 0.9565 | 0.8767 | 1.0000 |
| factor(X23.CS)=1 | 5.26 | 22 | 0 | 1 | 0.9565 | 0.8767 | 1.0000 |
| factor(X23.CS)=1 | 5.34 | 21 | 0 | 1 | 0.9565 | 0.8767 | 1.0000 |
| factor(X23.CS)=1 | 5.4 | 20 | 0 | 1 | 0.9565 | 0.8767 | 1.0000 |
| factor(X23.CS)=1 | 5.42 | 19 | 0 | 1 | 0.9565 | 0.8767 | 1.0000 |
| factor(X23.CS)=1 | 5.48 | 18 | 0 | 1 | 0.9565 | 0.8767 | 1.0000 |
| factor(X23.CS)=1 | 5.81 | 17 | 1 | 0 | 0.9003 | 0.7769 | 1.0000 |
| factor(X23.CS)=1 | 5.89 | 16 | 0 | 1 | 0.9003 | 0.7769 | 1.0000 |
| factor(X23.CS)=1 | 6.16 | 15 | 0 | 1 | 0.9003 | 0.7769 | 1.0000 |
| factor(X23.CS)=1 | 6.2 | 14 | 0 | 1 | 0.9003 | 0.7769 | 1.0000 |
| factor(X23.CS)=1 | 6.38 | 13 | 0 | 1 | 0.9003 | 0.7769 | 1.0000 |
| factor(X23.CS)=1 | 6.51 | 12 | 0 | 1 | 0.9003 | 0.7769 | 1.0000 |
| factor(X23.CS)=1 | 6.56 | 11 | 0 | 1 | 0.9003 | 0.7769 | 1.0000 |
| factor(X23.CS)=1 | 6.87 | 10 | 0 | 1 | 0.9003 | 0.7769 | 1.0000 |
| factor(X23.CS)=1 | 7.27 | 9 | 0 | 1 | 0.9003 | 0.7769 | 1.0000 |
| factor(X23.CS)=1 | 7.48 | 8 | 0 | 1 | 0.9003 | 0.7769 | 1.0000 |
| factor(X23.CS)=1 | 7.72 | 7 | 0 | 1 | 0.9003 | 0.7769 | 1.0000 |
| factor(X23.CS)=1 | 7.74 | 6 | 0 | 2 | 0.9003 | 0.7769 | 1.0000 |
| factor(X23.CS)=1 | 7.9 | 4 | 0 | 1 | 0.9003 | 0.7769 | 1.0000 |
| factor(X23.CS)=1 | 8.06 | 3 | 0 | 1 | 0.9003 | 0.7769 | 1.0000 |
| factor(X23.CS)=1 | 8.69 | 2 | 0 | 1 | 0.9003 | 0.7769 | 1.0000 |
| factor(X23.CS)=1 | 8.88 | 1 | 0 | 1 | 0.9003 | 0.7769 | 1.0000 |

|  |  |  |  |  |  |  |  |  |  |
| --- | --- | --- | --- | --- | --- | --- | --- | --- | --- |
| X23.CS | records | n.max | n.start | events | \*rmean | \*se(rmean) | median | 0.95LCL | 0.95UCL |
| factor(X23.CS)=0 | 178 | 178 | 178 | 1 | 9.155 | 0.086 | NA | NA | NA |
| factor(X23.CS)=1 | 23 | 23 | 23 | 2 | 8.872 | 0.251 | NA | NA | NA |

Use subset of data: (!is.na(X5) & (X5==1))
Created by EmpowerStats (www.empowerstats.com) and R on 2025-10-07
