# Supplementary material for: Optimal minimal residual disease threshold in pediatric acute myeloid leukemia: A retrospective cohort study based on the TARGET database
Source: PLoS Med. 2026 May 8;23(5):e1005088. doi: 10.1371/journal.pmed.1005088 (PMC13155632; doi:10.1371/journal.pmed.1005088)
Supplement: S1 Code — (ZIP) [file pmed.1005088.s002.zip › S2 code/PROJ8_4_tbl/PROJ8_4_tbl.htm]

## Kaplan Meier Survival Curve

Outcome: First Event
Time: EFS(years)
Landmark analysis for time segment: EFS(years) <= 5
Log rank test: implements the G-rho family of Harrington and Fleming (1982), with weights on each death of S(t)^rho, where S is the Kaplan-Meier estimate of survival. With rho = 0 this is the log-rank or Mantel-Haenszel test.

|  |  |  |  |
| --- | --- | --- | --- |
|  | N | Observed | Expected |
| factor(X22)=0 | 924 | 393 | 503.3186 |
| factor(X22)=1 | 281 | 210 | 99.6814 |

Chisq=
147.7647
on
1
degree of freedom, p=
<0.0001
Survival table

|  |  |  |  |  |  |  |  |
| --- | --- | --- | --- | --- | --- | --- | --- |
|  | EFS(years) | N.Risk | N.Event | N.Censor | Survival | 95%CI Low | 95%CI Upp |
| factor(X22)=0 | 0.16 | 924 | 1 | 0 | 0.9989 | 0.9968 | 1.0000 |
| factor(X22)=0 | 0.17 | 923 | 3 | 0 | 0.9957 | 0.9914 | 0.9999 |
| factor(X22)=0 | 0.18 | 920 | 2 | 0 | 0.9935 | 0.9883 | 0.9987 |
| factor(X22)=0 | 0.19 | 918 | 1 | 1 | 0.9924 | 0.9868 | 0.9980 |
| factor(X22)=0 | 0.2 | 916 | 4 | 0 | 0.9881 | 0.9811 | 0.9951 |
| factor(X22)=0 | 0.21 | 912 | 4 | 0 | 0.9838 | 0.9756 | 0.9919 |
| factor(X22)=0 | 0.22 | 908 | 1 | 0 | 0.9827 | 0.9743 | 0.9911 |
| factor(X22)=0 | 0.23 | 907 | 2 | 1 | 0.9805 | 0.9716 | 0.9895 |
| factor(X22)=0 | 0.24 | 904 | 1 | 0 | 0.9794 | 0.9703 | 0.9886 |
| factor(X22)=0 | 0.26 | 903 | 4 | 0 | 0.9751 | 0.9651 | 0.9852 |
| factor(X22)=0 | 0.27 | 899 | 2 | 1 | 0.9729 | 0.9625 | 0.9834 |
| factor(X22)=0 | 0.28 | 896 | 4 | 0 | 0.9686 | 0.9574 | 0.9799 |
| factor(X22)=0 | 0.29 | 892 | 3 | 0 | 0.9653 | 0.9536 | 0.9772 |
| factor(X22)=0 | 0.31 | 889 | 1 | 0 | 0.9642 | 0.9523 | 0.9763 |
| factor(X22)=0 | 0.32 | 888 | 2 | 0 | 0.9621 | 0.9498 | 0.9745 |
| factor(X22)=0 | 0.33 | 886 | 2 | 0 | 0.9599 | 0.9473 | 0.9726 |
| factor(X22)=0 | 0.37 | 884 | 2 | 0 | 0.9577 | 0.9448 | 0.9708 |
| factor(X22)=0 | 0.38 | 882 | 2 | 0 | 0.9555 | 0.9423 | 0.9689 |
| factor(X22)=0 | 0.4 | 880 | 4 | 0 | 0.9512 | 0.9374 | 0.9652 |
| factor(X22)=0 | 0.41 | 876 | 4 | 0 | 0.9469 | 0.9325 | 0.9614 |
| factor(X22)=0 | 0.42 | 872 | 2 | 0 | 0.9447 | 0.9300 | 0.9596 |
| factor(X22)=0 | 0.44 | 870 | 5 | 0 | 0.9393 | 0.9240 | 0.9548 |
| factor(X22)=0 | 0.45 | 865 | 2 | 0 | 0.9371 | 0.9215 | 0.9529 |
| factor(X22)=0 | 0.46 | 863 | 2 | 0 | 0.9349 | 0.9191 | 0.9510 |
| factor(X22)=0 | 0.47 | 861 | 3 | 0 | 0.9317 | 0.9155 | 0.9481 |
| factor(X22)=0 | 0.5 | 858 | 3 | 0 | 0.9284 | 0.9119 | 0.9452 |
| factor(X22)=0 | 0.51 | 855 | 1 | 0 | 0.9273 | 0.9107 | 0.9442 |
| factor(X22)=0 | 0.52 | 854 | 3 | 0 | 0.9241 | 0.9071 | 0.9413 |
| factor(X22)=0 | 0.53 | 851 | 5 | 0 | 0.9186 | 0.9011 | 0.9364 |
| factor(X22)=0 | 0.54 | 846 | 2 | 0 | 0.9165 | 0.8988 | 0.9345 |
| factor(X22)=0 | 0.55 | 844 | 3 | 0 | 0.9132 | 0.8952 | 0.9316 |
| factor(X22)=0 | 0.56 | 841 | 6 | 0 | 0.9067 | 0.8881 | 0.9257 |
| factor(X22)=0 | 0.57 | 835 | 4 | 0 | 0.9023 | 0.8834 | 0.9217 |
| factor(X22)=0 | 0.58 | 831 | 4 | 0 | 0.8980 | 0.8787 | 0.9177 |
| factor(X22)=0 | 0.59 | 827 | 4 | 0 | 0.8936 | 0.8740 | 0.9138 |
| factor(X22)=0 | 0.6 | 823 | 4 | 0 | 0.8893 | 0.8693 | 0.9098 |
| factor(X22)=0 | 0.61 | 819 | 1 | 0 | 0.8882 | 0.8681 | 0.9088 |
| factor(X22)=0 | 0.62 | 818 | 2 | 0 | 0.8860 | 0.8658 | 0.9068 |
| factor(X22)=0 | 0.63 | 816 | 1 | 0 | 0.8850 | 0.8646 | 0.9058 |
| factor(X22)=0 | 0.64 | 815 | 4 | 0 | 0.8806 | 0.8599 | 0.9018 |
| factor(X22)=0 | 0.65 | 811 | 4 | 0 | 0.8763 | 0.8553 | 0.8978 |
| factor(X22)=0 | 0.66 | 807 | 1 | 0 | 0.8752 | 0.8541 | 0.8968 |
| factor(X22)=0 | 0.67 | 806 | 3 | 0 | 0.8719 | 0.8506 | 0.8938 |
| factor(X22)=0 | 0.68 | 803 | 5 | 0 | 0.8665 | 0.8448 | 0.8887 |
| factor(X22)=0 | 0.69 | 798 | 4 | 0 | 0.8622 | 0.8402 | 0.8847 |
| factor(X22)=0 | 0.7 | 794 | 5 | 0 | 0.8567 | 0.8344 | 0.8797 |
| factor(X22)=0 | 0.71 | 789 | 3 | 0 | 0.8535 | 0.8309 | 0.8766 |
| factor(X22)=0 | 0.72 | 786 | 5 | 0 | 0.8480 | 0.8252 | 0.8715 |
| factor(X22)=0 | 0.73 | 781 | 4 | 0 | 0.8437 | 0.8206 | 0.8675 |
| factor(X22)=0 | 0.74 | 777 | 4 | 0 | 0.8394 | 0.8160 | 0.8634 |
| factor(X22)=0 | 0.75 | 773 | 2 | 0 | 0.8372 | 0.8137 | 0.8614 |
| factor(X22)=0 | 0.76 | 771 | 10 | 0 | 0.8263 | 0.8022 | 0.8512 |
| factor(X22)=0 | 0.77 | 761 | 1 | 0 | 0.8252 | 0.8011 | 0.8501 |
| factor(X22)=0 | 0.78 | 760 | 2 | 0 | 0.8231 | 0.7988 | 0.8481 |
| factor(X22)=0 | 0.79 | 758 | 6 | 0 | 0.8166 | 0.7919 | 0.8419 |
| factor(X22)=0 | 0.8 | 752 | 4 | 0 | 0.8122 | 0.7874 | 0.8378 |
| factor(X22)=0 | 0.81 | 748 | 5 | 0 | 0.8068 | 0.7817 | 0.8327 |
| factor(X22)=0 | 0.82 | 743 | 6 | 0 | 0.8003 | 0.7749 | 0.8265 |
| factor(X22)=0 | 0.83 | 737 | 4 | 0 | 0.7959 | 0.7703 | 0.8224 |
| factor(X22)=0 | 0.84 | 733 | 5 | 0 | 0.7905 | 0.7646 | 0.8172 |
| factor(X22)=0 | 0.85 | 728 | 1 | 0 | 0.7894 | 0.7635 | 0.8162 |
| factor(X22)=0 | 0.86 | 727 | 2 | 0 | 0.7872 | 0.7612 | 0.8141 |
| factor(X22)=0 | 0.87 | 725 | 1 | 0 | 0.7861 | 0.7601 | 0.8131 |
| factor(X22)=0 | 0.88 | 724 | 7 | 0 | 0.7785 | 0.7522 | 0.8058 |
| factor(X22)=0 | 0.89 | 717 | 2 | 0 | 0.7764 | 0.7499 | 0.8038 |
| factor(X22)=0 | 0.9 | 715 | 3 | 0 | 0.7731 | 0.7465 | 0.8006 |
| factor(X22)=0 | 0.91 | 712 | 2 | 0 | 0.7709 | 0.7443 | 0.7986 |
| factor(X22)=0 | 0.92 | 710 | 4 | 0 | 0.7666 | 0.7398 | 0.7944 |
| factor(X22)=0 | 0.93 | 706 | 8 | 0 | 0.7579 | 0.7308 | 0.7861 |
| factor(X22)=0 | 0.94 | 698 | 2 | 0 | 0.7557 | 0.7285 | 0.7840 |
| factor(X22)=0 | 0.95 | 696 | 1 | 0 | 0.7547 | 0.7274 | 0.7830 |
| factor(X22)=0 | 0.96 | 695 | 2 | 1 | 0.7525 | 0.7251 | 0.7809 |
| factor(X22)=0 | 0.97 | 692 | 1 | 0 | 0.7514 | 0.7240 | 0.7798 |
| factor(X22)=0 | 0.99 | 691 | 2 | 1 | 0.7492 | 0.7217 | 0.7777 |
| factor(X22)=0 | 1 | 688 | 4 | 0 | 0.7449 | 0.7172 | 0.7736 |
| factor(X22)=0 | 1.01 | 684 | 3 | 0 | 0.7416 | 0.7139 | 0.7704 |
| factor(X22)=0 | 1.02 | 681 | 3 | 1 | 0.7383 | 0.7105 | 0.7673 |
| factor(X22)=0 | 1.03 | 677 | 3 | 0 | 0.7351 | 0.7071 | 0.7641 |
| factor(X22)=0 | 1.05 | 674 | 6 | 0 | 0.7285 | 0.7004 | 0.7578 |
| factor(X22)=0 | 1.06 | 668 | 3 | 1 | 0.7252 | 0.6970 | 0.7547 |
| factor(X22)=0 | 1.07 | 664 | 4 | 0 | 0.7209 | 0.6925 | 0.7504 |
| factor(X22)=0 | 1.08 | 660 | 4 | 0 | 0.7165 | 0.6880 | 0.7462 |
| factor(X22)=0 | 1.09 | 656 | 2 | 0 | 0.7143 | 0.6857 | 0.7441 |
| factor(X22)=0 | 1.1 | 654 | 2 | 0 | 0.7121 | 0.6835 | 0.7420 |
| factor(X22)=0 | 1.11 | 652 | 0 | 2 | 0.7121 | 0.6835 | 0.7420 |
| factor(X22)=0 | 1.12 | 650 | 3 | 1 | 0.7089 | 0.6801 | 0.7388 |
| factor(X22)=0 | 1.13 | 646 | 4 | 0 | 0.7045 | 0.6756 | 0.7346 |
| factor(X22)=0 | 1.14 | 642 | 2 | 1 | 0.7023 | 0.6733 | 0.7324 |
| factor(X22)=0 | 1.15 | 639 | 1 | 1 | 0.7012 | 0.6722 | 0.7314 |
| factor(X22)=0 | 1.16 | 637 | 1 | 0 | 0.7001 | 0.6711 | 0.7303 |
| factor(X22)=0 | 1.17 | 636 | 1 | 0 | 0.6990 | 0.6699 | 0.7293 |
| factor(X22)=0 | 1.18 | 635 | 1 | 0 | 0.6979 | 0.6688 | 0.7282 |
| factor(X22)=0 | 1.19 | 634 | 2 | 0 | 0.6957 | 0.6666 | 0.7261 |
| factor(X22)=0 | 1.2 | 632 | 3 | 0 | 0.6924 | 0.6632 | 0.7228 |
| factor(X22)=0 | 1.21 | 629 | 1 | 1 | 0.6913 | 0.6620 | 0.7218 |
| factor(X22)=0 | 1.24 | 627 | 1 | 0 | 0.6902 | 0.6609 | 0.7207 |
| factor(X22)=0 | 1.25 | 626 | 0 | 1 | 0.6902 | 0.6609 | 0.7207 |
| factor(X22)=0 | 1.26 | 625 | 1 | 0 | 0.6891 | 0.6598 | 0.7196 |
| factor(X22)=0 | 1.27 | 624 | 4 | 0 | 0.6846 | 0.6552 | 0.7154 |
| factor(X22)=0 | 1.28 | 620 | 2 | 1 | 0.6824 | 0.6530 | 0.7132 |
| factor(X22)=0 | 1.3 | 617 | 3 | 0 | 0.6791 | 0.6496 | 0.7100 |
| factor(X22)=0 | 1.31 | 614 | 2 | 0 | 0.6769 | 0.6473 | 0.7078 |
| factor(X22)=0 | 1.32 | 612 | 1 | 0 | 0.6758 | 0.6462 | 0.7068 |
| factor(X22)=0 | 1.34 | 611 | 3 | 1 | 0.6725 | 0.6428 | 0.7035 |
| factor(X22)=0 | 1.35 | 607 | 1 | 0 | 0.6714 | 0.6417 | 0.7025 |
| factor(X22)=0 | 1.36 | 606 | 2 | 0 | 0.6692 | 0.6394 | 0.7003 |
| factor(X22)=0 | 1.37 | 604 | 2 | 1 | 0.6669 | 0.6371 | 0.6981 |
| factor(X22)=0 | 1.38 | 601 | 2 | 0 | 0.6647 | 0.6349 | 0.6960 |
| factor(X22)=0 | 1.39 | 599 | 2 | 0 | 0.6625 | 0.6326 | 0.6938 |
| factor(X22)=0 | 1.4 | 597 | 1 | 0 | 0.6614 | 0.6315 | 0.6927 |
| factor(X22)=0 | 1.41 | 596 | 1 | 0 | 0.6603 | 0.6303 | 0.6917 |
| factor(X22)=0 | 1.42 | 595 | 4 | 0 | 0.6558 | 0.6258 | 0.6873 |
| factor(X22)=0 | 1.43 | 591 | 4 | 0 | 0.6514 | 0.6213 | 0.6830 |
| factor(X22)=0 | 1.45 | 587 | 1 | 0 | 0.6503 | 0.6201 | 0.6819 |
| factor(X22)=0 | 1.46 | 586 | 1 | 0 | 0.6492 | 0.6190 | 0.6808 |
| factor(X22)=0 | 1.48 | 585 | 2 | 0 | 0.6470 | 0.6167 | 0.6787 |
| factor(X22)=0 | 1.49 | 583 | 3 | 0 | 0.6436 | 0.6134 | 0.6754 |
| factor(X22)=0 | 1.51 | 580 | 1 | 0 | 0.6425 | 0.6122 | 0.6743 |
| factor(X22)=0 | 1.53 | 579 | 1 | 0 | 0.6414 | 0.6111 | 0.6732 |
| factor(X22)=0 | 1.54 | 578 | 1 | 0 | 0.6403 | 0.6100 | 0.6722 |
| factor(X22)=0 | 1.55 | 577 | 1 | 0 | 0.6392 | 0.6088 | 0.6711 |
| factor(X22)=0 | 1.56 | 576 | 1 | 0 | 0.6381 | 0.6077 | 0.6700 |
| factor(X22)=0 | 1.58 | 575 | 4 | 0 | 0.6336 | 0.6032 | 0.6656 |
| factor(X22)=0 | 1.6 | 571 | 2 | 0 | 0.6314 | 0.6009 | 0.6635 |
| factor(X22)=0 | 1.61 | 569 | 0 | 1 | 0.6314 | 0.6009 | 0.6635 |
| factor(X22)=0 | 1.62 | 568 | 2 | 0 | 0.6292 | 0.5987 | 0.6613 |
| factor(X22)=0 | 1.64 | 566 | 1 | 0 | 0.6281 | 0.5975 | 0.6602 |
| factor(X22)=0 | 1.65 | 565 | 2 | 0 | 0.6259 | 0.5953 | 0.6580 |
| factor(X22)=0 | 1.66 | 563 | 1 | 0 | 0.6248 | 0.5942 | 0.6569 |
| factor(X22)=0 | 1.67 | 562 | 1 | 0 | 0.6236 | 0.5930 | 0.6558 |
| factor(X22)=0 | 1.71 | 561 | 0 | 1 | 0.6236 | 0.5930 | 0.6558 |
| factor(X22)=0 | 1.72 | 560 | 2 | 0 | 0.6214 | 0.5908 | 0.6537 |
| factor(X22)=0 | 1.75 | 558 | 0 | 1 | 0.6214 | 0.5908 | 0.6537 |
| factor(X22)=0 | 1.77 | 557 | 1 | 0 | 0.6203 | 0.5896 | 0.6526 |
| factor(X22)=0 | 1.78 | 556 | 1 | 0 | 0.6192 | 0.5885 | 0.6515 |
| factor(X22)=0 | 1.81 | 555 | 1 | 0 | 0.6181 | 0.5874 | 0.6504 |
| factor(X22)=0 | 1.83 | 554 | 0 | 1 | 0.6181 | 0.5874 | 0.6504 |
| factor(X22)=0 | 1.87 | 553 | 1 | 1 | 0.6170 | 0.5862 | 0.6493 |
| factor(X22)=0 | 1.89 | 551 | 2 | 0 | 0.6147 | 0.5840 | 0.6471 |
| factor(X22)=0 | 1.9 | 549 | 2 | 0 | 0.6125 | 0.5817 | 0.6449 |
| factor(X22)=0 | 1.92 | 547 | 1 | 1 | 0.6114 | 0.5806 | 0.6438 |
| factor(X22)=0 | 1.94 | 545 | 1 | 2 | 0.6102 | 0.5794 | 0.6427 |
| factor(X22)=0 | 1.96 | 542 | 0 | 1 | 0.6102 | 0.5794 | 0.6427 |
| factor(X22)=0 | 1.98 | 541 | 0 | 1 | 0.6102 | 0.5794 | 0.6427 |
| factor(X22)=0 | 1.99 | 540 | 1 | 0 | 0.6091 | 0.5783 | 0.6416 |
| factor(X22)=0 | 2.03 | 539 | 1 | 0 | 0.6080 | 0.5771 | 0.6405 |
| factor(X22)=0 | 2.05 | 538 | 1 | 0 | 0.6068 | 0.5760 | 0.6394 |
| factor(X22)=0 | 2.07 | 537 | 1 | 0 | 0.6057 | 0.5748 | 0.6383 |
| factor(X22)=0 | 2.08 | 536 | 1 | 0 | 0.6046 | 0.5737 | 0.6371 |
| factor(X22)=0 | 2.09 | 535 | 1 | 0 | 0.6035 | 0.5725 | 0.6360 |
| factor(X22)=0 | 2.1 | 534 | 0 | 1 | 0.6035 | 0.5725 | 0.6360 |
| factor(X22)=0 | 2.11 | 533 | 1 | 0 | 0.6023 | 0.5714 | 0.6349 |
| factor(X22)=0 | 2.13 | 532 | 1 | 0 | 0.6012 | 0.5702 | 0.6338 |
| factor(X22)=0 | 2.14 | 531 | 1 | 0 | 0.6001 | 0.5691 | 0.6327 |
| factor(X22)=0 | 2.15 | 530 | 1 | 0 | 0.5989 | 0.5680 | 0.6316 |
| factor(X22)=0 | 2.18 | 529 | 0 | 1 | 0.5989 | 0.5680 | 0.6316 |
| factor(X22)=0 | 2.2 | 528 | 1 | 0 | 0.5978 | 0.5668 | 0.6305 |
| factor(X22)=0 | 2.22 | 527 | 2 | 1 | 0.5955 | 0.5645 | 0.6282 |
| factor(X22)=0 | 2.23 | 524 | 1 | 1 | 0.5944 | 0.5634 | 0.6271 |
| factor(X22)=0 | 2.27 | 522 | 0 | 2 | 0.5944 | 0.5634 | 0.6271 |
| factor(X22)=0 | 2.3 | 520 | 1 | 0 | 0.5932 | 0.5622 | 0.6260 |
| factor(X22)=0 | 2.31 | 519 | 1 | 0 | 0.5921 | 0.5610 | 0.6249 |
| factor(X22)=0 | 2.32 | 518 | 0 | 1 | 0.5921 | 0.5610 | 0.6249 |
| factor(X22)=0 | 2.34 | 517 | 1 | 0 | 0.5910 | 0.5599 | 0.6238 |
| factor(X22)=0 | 2.36 | 516 | 1 | 0 | 0.5898 | 0.5587 | 0.6226 |
| factor(X22)=0 | 2.37 | 515 | 1 | 1 | 0.5887 | 0.5576 | 0.6215 |
| factor(X22)=0 | 2.39 | 513 | 0 | 1 | 0.5887 | 0.5576 | 0.6215 |
| factor(X22)=0 | 2.4 | 512 | 0 | 1 | 0.5887 | 0.5576 | 0.6215 |
| factor(X22)=0 | 2.41 | 511 | 0 | 1 | 0.5887 | 0.5576 | 0.6215 |
| factor(X22)=0 | 2.42 | 510 | 1 | 0 | 0.5875 | 0.5564 | 0.6204 |
| factor(X22)=0 | 2.43 | 509 | 0 | 1 | 0.5875 | 0.5564 | 0.6204 |
| factor(X22)=0 | 2.44 | 508 | 0 | 2 | 0.5875 | 0.5564 | 0.6204 |
| factor(X22)=0 | 2.46 | 506 | 0 | 1 | 0.5875 | 0.5564 | 0.6204 |
| factor(X22)=0 | 2.47 | 505 | 0 | 1 | 0.5875 | 0.5564 | 0.6204 |
| factor(X22)=0 | 2.48 | 504 | 1 | 0 | 0.5863 | 0.5552 | 0.6192 |
| factor(X22)=0 | 2.49 | 503 | 0 | 1 | 0.5863 | 0.5552 | 0.6192 |
| factor(X22)=0 | 2.5 | 502 | 0 | 4 | 0.5863 | 0.5552 | 0.6192 |
| factor(X22)=0 | 2.51 | 498 | 1 | 1 | 0.5852 | 0.5540 | 0.6181 |
| factor(X22)=0 | 2.52 | 496 | 1 | 0 | 0.5840 | 0.5528 | 0.6169 |
| factor(X22)=0 | 2.53 | 495 | 0 | 1 | 0.5840 | 0.5528 | 0.6169 |
| factor(X22)=0 | 2.55 | 494 | 0 | 1 | 0.5840 | 0.5528 | 0.6169 |
| factor(X22)=0 | 2.56 | 493 | 0 | 1 | 0.5840 | 0.5528 | 0.6169 |
| factor(X22)=0 | 2.6 | 492 | 0 | 1 | 0.5840 | 0.5528 | 0.6169 |
| factor(X22)=0 | 2.61 | 491 | 1 | 1 | 0.5828 | 0.5516 | 0.6157 |
| factor(X22)=0 | 2.62 | 489 | 2 | 0 | 0.5804 | 0.5492 | 0.6134 |
| factor(X22)=0 | 2.63 | 487 | 0 | 1 | 0.5804 | 0.5492 | 0.6134 |
| factor(X22)=0 | 2.64 | 486 | 0 | 1 | 0.5804 | 0.5492 | 0.6134 |
| factor(X22)=0 | 2.68 | 485 | 0 | 1 | 0.5804 | 0.5492 | 0.6134 |
| factor(X22)=0 | 2.7 | 484 | 1 | 1 | 0.5792 | 0.5480 | 0.6122 |
| factor(X22)=0 | 2.73 | 482 | 0 | 1 | 0.5792 | 0.5480 | 0.6122 |
| factor(X22)=0 | 2.75 | 481 | 0 | 1 | 0.5792 | 0.5480 | 0.6122 |
| factor(X22)=0 | 2.78 | 480 | 0 | 2 | 0.5792 | 0.5480 | 0.6122 |
| factor(X22)=0 | 2.79 | 478 | 0 | 2 | 0.5792 | 0.5480 | 0.6122 |
| factor(X22)=0 | 2.83 | 476 | 0 | 1 | 0.5792 | 0.5480 | 0.6122 |
| factor(X22)=0 | 2.89 | 475 | 0 | 1 | 0.5792 | 0.5480 | 0.6122 |
| factor(X22)=0 | 2.93 | 474 | 0 | 1 | 0.5792 | 0.5480 | 0.6122 |
| factor(X22)=0 | 2.98 | 473 | 0 | 1 | 0.5792 | 0.5480 | 0.6122 |
| factor(X22)=0 | 3.01 | 472 | 0 | 1 | 0.5792 | 0.5480 | 0.6122 |
| factor(X22)=0 | 3.02 | 471 | 0 | 1 | 0.5792 | 0.5480 | 0.6122 |
| factor(X22)=0 | 3.03 | 470 | 0 | 1 | 0.5792 | 0.5480 | 0.6122 |
| factor(X22)=0 | 3.04 | 469 | 0 | 2 | 0.5792 | 0.5480 | 0.6122 |
| factor(X22)=0 | 3.05 | 467 | 0 | 1 | 0.5792 | 0.5480 | 0.6122 |
| factor(X22)=0 | 3.07 | 466 | 0 | 1 | 0.5792 | 0.5480 | 0.6122 |
| factor(X22)=0 | 3.11 | 465 | 0 | 1 | 0.5792 | 0.5480 | 0.6122 |
| factor(X22)=0 | 3.14 | 464 | 0 | 1 | 0.5792 | 0.5480 | 0.6122 |
| factor(X22)=0 | 3.15 | 463 | 0 | 1 | 0.5792 | 0.5480 | 0.6122 |
| factor(X22)=0 | 3.16 | 462 | 0 | 2 | 0.5792 | 0.5480 | 0.6122 |
| factor(X22)=0 | 3.17 | 460 | 0 | 1 | 0.5792 | 0.5480 | 0.6122 |
| factor(X22)=0 | 3.18 | 459 | 1 | 2 | 0.5780 | 0.5467 | 0.6110 |
| factor(X22)=0 | 3.19 | 456 | 0 | 1 | 0.5780 | 0.5467 | 0.6110 |
| factor(X22)=0 | 3.2 | 455 | 0 | 1 | 0.5780 | 0.5467 | 0.6110 |
| factor(X22)=0 | 3.22 | 454 | 0 | 1 | 0.5780 | 0.5467 | 0.6110 |
| factor(X22)=0 | 3.23 | 453 | 0 | 1 | 0.5780 | 0.5467 | 0.6110 |
| factor(X22)=0 | 3.24 | 452 | 0 | 1 | 0.5780 | 0.5467 | 0.6110 |
| factor(X22)=0 | 3.25 | 451 | 0 | 1 | 0.5780 | 0.5467 | 0.6110 |
| factor(X22)=0 | 3.28 | 450 | 0 | 3 | 0.5780 | 0.5467 | 0.6110 |
| factor(X22)=0 | 3.29 | 447 | 0 | 1 | 0.5780 | 0.5467 | 0.6110 |
| factor(X22)=0 | 3.3 | 446 | 0 | 2 | 0.5780 | 0.5467 | 0.6110 |
| factor(X22)=0 | 3.32 | 444 | 0 | 1 | 0.5780 | 0.5467 | 0.6110 |
| factor(X22)=0 | 3.33 | 443 | 0 | 3 | 0.5780 | 0.5467 | 0.6110 |
| factor(X22)=0 | 3.34 | 440 | 0 | 2 | 0.5780 | 0.5467 | 0.6110 |
| factor(X22)=0 | 3.35 | 438 | 0 | 3 | 0.5780 | 0.5467 | 0.6110 |
| factor(X22)=0 | 3.36 | 435 | 0 | 2 | 0.5780 | 0.5467 | 0.6110 |
| factor(X22)=0 | 3.38 | 433 | 0 | 3 | 0.5780 | 0.5467 | 0.6110 |
| factor(X22)=0 | 3.39 | 430 | 1 | 2 | 0.5766 | 0.5453 | 0.6097 |
| factor(X22)=0 | 3.4 | 427 | 0 | 1 | 0.5766 | 0.5453 | 0.6097 |
| factor(X22)=0 | 3.41 | 426 | 0 | 4 | 0.5766 | 0.5453 | 0.6097 |
| factor(X22)=0 | 3.43 | 422 | 0 | 1 | 0.5766 | 0.5453 | 0.6097 |
| factor(X22)=0 | 3.44 | 421 | 0 | 4 | 0.5766 | 0.5453 | 0.6097 |
| factor(X22)=0 | 3.45 | 417 | 0 | 2 | 0.5766 | 0.5453 | 0.6097 |
| factor(X22)=0 | 3.46 | 415 | 0 | 1 | 0.5766 | 0.5453 | 0.6097 |
| factor(X22)=0 | 3.48 | 414 | 0 | 2 | 0.5766 | 0.5453 | 0.6097 |
| factor(X22)=0 | 3.5 | 412 | 0 | 3 | 0.5766 | 0.5453 | 0.6097 |
| factor(X22)=0 | 3.52 | 409 | 0 | 1 | 0.5766 | 0.5453 | 0.6097 |
| factor(X22)=0 | 3.54 | 408 | 1 | 1 | 0.5752 | 0.5439 | 0.6083 |
| factor(X22)=0 | 3.56 | 406 | 0 | 4 | 0.5752 | 0.5439 | 0.6083 |
| factor(X22)=0 | 3.58 | 402 | 0 | 1 | 0.5752 | 0.5439 | 0.6083 |
| factor(X22)=0 | 3.62 | 401 | 0 | 1 | 0.5752 | 0.5439 | 0.6083 |
| factor(X22)=0 | 3.63 | 400 | 0 | 1 | 0.5752 | 0.5439 | 0.6083 |
| factor(X22)=0 | 3.65 | 399 | 0 | 2 | 0.5752 | 0.5439 | 0.6083 |
| factor(X22)=0 | 3.66 | 397 | 0 | 1 | 0.5752 | 0.5439 | 0.6083 |
| factor(X22)=0 | 3.74 | 396 | 0 | 1 | 0.5752 | 0.5439 | 0.6083 |
| factor(X22)=0 | 3.79 | 395 | 0 | 1 | 0.5752 | 0.5439 | 0.6083 |
| factor(X22)=0 | 3.81 | 394 | 0 | 2 | 0.5752 | 0.5439 | 0.6083 |
| factor(X22)=0 | 3.83 | 392 | 0 | 1 | 0.5752 | 0.5439 | 0.6083 |
| factor(X22)=0 | 3.84 | 391 | 0 | 2 | 0.5752 | 0.5439 | 0.6083 |
| factor(X22)=0 | 3.89 | 389 | 0 | 1 | 0.5752 | 0.5439 | 0.6083 |
| factor(X22)=0 | 3.9 | 388 | 0 | 2 | 0.5752 | 0.5439 | 0.6083 |
| factor(X22)=0 | 3.95 | 386 | 0 | 1 | 0.5752 | 0.5439 | 0.6083 |
| factor(X22)=0 | 3.96 | 385 | 0 | 1 | 0.5752 | 0.5439 | 0.6083 |
| factor(X22)=0 | 3.97 | 384 | 0 | 1 | 0.5752 | 0.5439 | 0.6083 |
| factor(X22)=0 | 3.99 | 383 | 1 | 0 | 0.5737 | 0.5423 | 0.6069 |
| factor(X22)=0 | 4.04 | 382 | 0 | 1 | 0.5737 | 0.5423 | 0.6069 |
| factor(X22)=0 | 4.05 | 381 | 0 | 2 | 0.5737 | 0.5423 | 0.6069 |
| factor(X22)=0 | 4.06 | 379 | 0 | 2 | 0.5737 | 0.5423 | 0.6069 |
| factor(X22)=0 | 4.07 | 377 | 1 | 1 | 0.5722 | 0.5408 | 0.6054 |
| factor(X22)=0 | 4.08 | 375 | 0 | 2 | 0.5722 | 0.5408 | 0.6054 |
| factor(X22)=0 | 4.13 | 373 | 0 | 2 | 0.5722 | 0.5408 | 0.6054 |
| factor(X22)=0 | 4.15 | 371 | 0 | 2 | 0.5722 | 0.5408 | 0.6054 |
| factor(X22)=0 | 4.19 | 369 | 0 | 2 | 0.5722 | 0.5408 | 0.6054 |
| factor(X22)=0 | 4.22 | 367 | 0 | 1 | 0.5722 | 0.5408 | 0.6054 |
| factor(X22)=0 | 4.23 | 366 | 1 | 0 | 0.5706 | 0.5391 | 0.6039 |
| factor(X22)=0 | 4.26 | 365 | 0 | 1 | 0.5706 | 0.5391 | 0.6039 |
| factor(X22)=0 | 4.27 | 364 | 0 | 2 | 0.5706 | 0.5391 | 0.6039 |
| factor(X22)=0 | 4.31 | 362 | 0 | 2 | 0.5706 | 0.5391 | 0.6039 |
| factor(X22)=0 | 4.32 | 360 | 0 | 1 | 0.5706 | 0.5391 | 0.6039 |
| factor(X22)=0 | 4.33 | 359 | 0 | 1 | 0.5706 | 0.5391 | 0.6039 |
| factor(X22)=0 | 4.34 | 358 | 0 | 1 | 0.5706 | 0.5391 | 0.6039 |
| factor(X22)=0 | 4.35 | 357 | 0 | 4 | 0.5706 | 0.5391 | 0.6039 |
| factor(X22)=0 | 4.36 | 353 | 0 | 4 | 0.5706 | 0.5391 | 0.6039 |
| factor(X22)=0 | 4.38 | 349 | 0 | 3 | 0.5706 | 0.5391 | 0.6039 |
| factor(X22)=0 | 4.4 | 346 | 0 | 1 | 0.5706 | 0.5391 | 0.6039 |
| factor(X22)=0 | 4.41 | 345 | 0 | 1 | 0.5706 | 0.5391 | 0.6039 |
| factor(X22)=0 | 4.42 | 344 | 0 | 2 | 0.5706 | 0.5391 | 0.6039 |
| factor(X22)=0 | 4.44 | 342 | 0 | 2 | 0.5706 | 0.5391 | 0.6039 |
| factor(X22)=0 | 4.45 | 340 | 0 | 1 | 0.5706 | 0.5391 | 0.6039 |
| factor(X22)=0 | 4.47 | 339 | 0 | 1 | 0.5706 | 0.5391 | 0.6039 |
| factor(X22)=0 | 4.48 | 338 | 0 | 1 | 0.5706 | 0.5391 | 0.6039 |
| factor(X22)=0 | 4.49 | 337 | 0 | 2 | 0.5706 | 0.5391 | 0.6039 |
| factor(X22)=0 | 4.5 | 335 | 0 | 1 | 0.5706 | 0.5391 | 0.6039 |
| factor(X22)=0 | 4.53 | 334 | 0 | 1 | 0.5706 | 0.5391 | 0.6039 |
| factor(X22)=0 | 4.54 | 333 | 0 | 1 | 0.5706 | 0.5391 | 0.6039 |
| factor(X22)=0 | 4.55 | 332 | 1 | 0 | 0.5689 | 0.5374 | 0.6023 |
| factor(X22)=0 | 4.58 | 331 | 0 | 2 | 0.5689 | 0.5374 | 0.6023 |
| factor(X22)=0 | 4.59 | 329 | 0 | 1 | 0.5689 | 0.5374 | 0.6023 |
| factor(X22)=0 | 4.6 | 328 | 0 | 3 | 0.5689 | 0.5374 | 0.6023 |
| factor(X22)=0 | 4.61 | 325 | 0 | 3 | 0.5689 | 0.5374 | 0.6023 |
| factor(X22)=0 | 4.62 | 322 | 0 | 1 | 0.5689 | 0.5374 | 0.6023 |
| factor(X22)=0 | 4.64 | 321 | 0 | 1 | 0.5689 | 0.5374 | 0.6023 |
| factor(X22)=0 | 4.68 | 320 | 1 | 0 | 0.5671 | 0.5355 | 0.6006 |
| factor(X22)=0 | 4.72 | 319 | 1 | 1 | 0.5653 | 0.5336 | 0.5989 |
| factor(X22)=0 | 4.73 | 317 | 0 | 2 | 0.5653 | 0.5336 | 0.5989 |
| factor(X22)=0 | 4.75 | 315 | 0 | 1 | 0.5653 | 0.5336 | 0.5989 |
| factor(X22)=0 | 4.79 | 314 | 0 | 1 | 0.5653 | 0.5336 | 0.5989 |
| factor(X22)=0 | 4.81 | 313 | 0 | 1 | 0.5653 | 0.5336 | 0.5989 |
| factor(X22)=0 | 4.82 | 312 | 0 | 1 | 0.5653 | 0.5336 | 0.5989 |
| factor(X22)=0 | 4.85 | 311 | 0 | 4 | 0.5653 | 0.5336 | 0.5989 |
| factor(X22)=0 | 4.87 | 307 | 0 | 2 | 0.5653 | 0.5336 | 0.5989 |
| factor(X22)=0 | 4.9 | 305 | 0 | 1 | 0.5653 | 0.5336 | 0.5989 |
| factor(X22)=0 | 4.91 | 304 | 0 | 1 | 0.5653 | 0.5336 | 0.5989 |
| factor(X22)=0 | 4.94 | 303 | 0 | 2 | 0.5653 | 0.5336 | 0.5989 |
| factor(X22)=0 | 4.97 | 301 | 0 | 1 | 0.5653 | 0.5336 | 0.5989 |
| factor(X22)=0 | 4.99 | 300 | 0 | 1 | 0.5653 | 0.5336 | 0.5989 |
| factor(X22)=0 | 5 | 299 | 0 | 299 | 0.5653 | 0.5336 | 0.5989 |
| factor(X22)=1 | 0.13 | 281 | 2 | 0 | 0.9929 | 0.9831 | 1.0000 |
| factor(X22)=1 | 0.15 | 279 | 2 | 0 | 0.9858 | 0.9720 | 0.9997 |
| factor(X22)=1 | 0.16 | 277 | 5 | 0 | 0.9680 | 0.9476 | 0.9888 |
| factor(X22)=1 | 0.17 | 272 | 3 | 0 | 0.9573 | 0.9339 | 0.9812 |
| factor(X22)=1 | 0.18 | 269 | 4 | 0 | 0.9431 | 0.9164 | 0.9705 |
| factor(X22)=1 | 0.19 | 265 | 11 | 0 | 0.9039 | 0.8701 | 0.9390 |
| factor(X22)=1 | 0.2 | 254 | 4 | 0 | 0.8897 | 0.8538 | 0.9271 |
| factor(X22)=1 | 0.21 | 250 | 11 | 0 | 0.8505 | 0.8099 | 0.8933 |
| factor(X22)=1 | 0.22 | 239 | 10 | 0 | 0.8149 | 0.7708 | 0.8616 |
| factor(X22)=1 | 0.23 | 229 | 8 | 0 | 0.7865 | 0.7400 | 0.8359 |
| factor(X22)=1 | 0.24 | 221 | 3 | 0 | 0.7758 | 0.7285 | 0.8261 |
| factor(X22)=1 | 0.25 | 218 | 3 | 0 | 0.7651 | 0.7171 | 0.8163 |
| factor(X22)=1 | 0.27 | 215 | 2 | 0 | 0.7580 | 0.7095 | 0.8098 |
| factor(X22)=1 | 0.28 | 213 | 3 | 0 | 0.7473 | 0.6982 | 0.7999 |
| factor(X22)=1 | 0.29 | 210 | 1 | 0 | 0.7438 | 0.6944 | 0.7966 |
| factor(X22)=1 | 0.3 | 209 | 3 | 0 | 0.7331 | 0.6832 | 0.7867 |
| factor(X22)=1 | 0.32 | 206 | 2 | 0 | 0.7260 | 0.6757 | 0.7800 |
| factor(X22)=1 | 0.33 | 204 | 1 | 1 | 0.7224 | 0.6719 | 0.7767 |
| factor(X22)=1 | 0.34 | 202 | 1 | 0 | 0.7188 | 0.6682 | 0.7734 |
| factor(X22)=1 | 0.35 | 201 | 2 | 0 | 0.7117 | 0.6606 | 0.7667 |
| factor(X22)=1 | 0.36 | 199 | 1 | 0 | 0.7081 | 0.6569 | 0.7633 |
| factor(X22)=1 | 0.37 | 198 | 1 | 0 | 0.7045 | 0.6532 | 0.7600 |
| factor(X22)=1 | 0.42 | 197 | 1 | 0 | 0.7010 | 0.6494 | 0.7566 |
| factor(X22)=1 | 0.43 | 196 | 1 | 0 | 0.6974 | 0.6457 | 0.7532 |
| factor(X22)=1 | 0.45 | 195 | 1 | 0 | 0.6938 | 0.6419 | 0.7499 |
| factor(X22)=1 | 0.47 | 194 | 2 | 0 | 0.6867 | 0.6345 | 0.7431 |
| factor(X22)=1 | 0.48 | 192 | 2 | 0 | 0.6795 | 0.6271 | 0.7363 |
| factor(X22)=1 | 0.49 | 190 | 3 | 0 | 0.6688 | 0.6159 | 0.7262 |
| factor(X22)=1 | 0.5 | 187 | 1 | 0 | 0.6652 | 0.6122 | 0.7228 |
| factor(X22)=1 | 0.52 | 186 | 2 | 0 | 0.6580 | 0.6048 | 0.7160 |
| factor(X22)=1 | 0.53 | 184 | 3 | 0 | 0.6473 | 0.5938 | 0.7057 |
| factor(X22)=1 | 0.54 | 181 | 1 | 0 | 0.6437 | 0.5901 | 0.7023 |
| factor(X22)=1 | 0.55 | 180 | 2 | 0 | 0.6366 | 0.5827 | 0.6954 |
| factor(X22)=1 | 0.56 | 178 | 1 | 0 | 0.6330 | 0.5790 | 0.6920 |
| factor(X22)=1 | 0.57 | 177 | 1 | 0 | 0.6294 | 0.5754 | 0.6886 |
| factor(X22)=1 | 0.58 | 176 | 1 | 0 | 0.6259 | 0.5717 | 0.6851 |
| factor(X22)=1 | 0.59 | 175 | 1 | 0 | 0.6223 | 0.5681 | 0.6817 |
| factor(X22)=1 | 0.6 | 174 | 1 | 0 | 0.6187 | 0.5644 | 0.6782 |
| factor(X22)=1 | 0.62 | 173 | 1 | 0 | 0.6151 | 0.5607 | 0.6748 |
| factor(X22)=1 | 0.64 | 172 | 1 | 0 | 0.6116 | 0.5571 | 0.6713 |
| factor(X22)=1 | 0.66 | 171 | 1 | 0 | 0.6080 | 0.5534 | 0.6679 |
| factor(X22)=1 | 0.68 | 170 | 3 | 0 | 0.5972 | 0.5425 | 0.6575 |
| factor(X22)=1 | 0.69 | 167 | 2 | 0 | 0.5901 | 0.5352 | 0.6506 |
| factor(X22)=1 | 0.7 | 165 | 2 | 0 | 0.5829 | 0.5280 | 0.6436 |
| factor(X22)=1 | 0.71 | 163 | 1 | 0 | 0.5794 | 0.5244 | 0.6401 |
| factor(X22)=1 | 0.72 | 162 | 3 | 0 | 0.5686 | 0.5135 | 0.6297 |
| factor(X22)=1 | 0.74 | 159 | 2 | 0 | 0.5615 | 0.5063 | 0.6227 |
| factor(X22)=1 | 0.75 | 157 | 2 | 0 | 0.5543 | 0.4991 | 0.6157 |
| factor(X22)=1 | 0.77 | 155 | 2 | 0 | 0.5472 | 0.4919 | 0.6087 |
| factor(X22)=1 | 0.78 | 153 | 3 | 1 | 0.5365 | 0.4811 | 0.5981 |
| factor(X22)=1 | 0.79 | 149 | 2 | 0 | 0.5292 | 0.4739 | 0.5910 |
| factor(X22)=1 | 0.81 | 147 | 3 | 0 | 0.5184 | 0.4631 | 0.5804 |
| factor(X22)=1 | 0.82 | 144 | 3 | 0 | 0.5076 | 0.4523 | 0.5697 |
| factor(X22)=1 | 0.84 | 141 | 4 | 0 | 0.4932 | 0.4380 | 0.5554 |
| factor(X22)=1 | 0.85 | 137 | 1 | 0 | 0.4896 | 0.4344 | 0.5519 |
| factor(X22)=1 | 0.86 | 136 | 1 | 1 | 0.4860 | 0.4309 | 0.5483 |
| factor(X22)=1 | 0.87 | 134 | 2 | 0 | 0.4788 | 0.4237 | 0.5411 |
| factor(X22)=1 | 0.88 | 132 | 1 | 0 | 0.4752 | 0.4201 | 0.5375 |
| factor(X22)=1 | 0.9 | 131 | 1 | 0 | 0.4715 | 0.4165 | 0.5338 |
| factor(X22)=1 | 0.91 | 130 | 4 | 0 | 0.4570 | 0.4022 | 0.5193 |
| factor(X22)=1 | 0.92 | 126 | 1 | 0 | 0.4534 | 0.3986 | 0.5157 |
| factor(X22)=1 | 0.93 | 125 | 1 | 0 | 0.4498 | 0.3950 | 0.5121 |
| factor(X22)=1 | 0.95 | 124 | 1 | 0 | 0.4461 | 0.3915 | 0.5084 |
| factor(X22)=1 | 0.97 | 123 | 2 | 0 | 0.4389 | 0.3844 | 0.5012 |
| factor(X22)=1 | 0.98 | 121 | 1 | 0 | 0.4353 | 0.3808 | 0.4975 |
| factor(X22)=1 | 0.99 | 120 | 2 | 0 | 0.4280 | 0.3737 | 0.4902 |
| factor(X22)=1 | 1 | 118 | 1 | 0 | 0.4244 | 0.3702 | 0.4865 |
| factor(X22)=1 | 1.02 | 117 | 1 | 0 | 0.4208 | 0.3666 | 0.4829 |
| factor(X22)=1 | 1.07 | 116 | 1 | 0 | 0.4171 | 0.3631 | 0.4792 |
| factor(X22)=1 | 1.08 | 115 | 2 | 0 | 0.4099 | 0.3560 | 0.4719 |
| factor(X22)=1 | 1.11 | 113 | 1 | 0 | 0.4062 | 0.3525 | 0.4682 |
| factor(X22)=1 | 1.12 | 112 | 1 | 0 | 0.4026 | 0.3490 | 0.4645 |
| factor(X22)=1 | 1.15 | 111 | 2 | 0 | 0.3954 | 0.3419 | 0.4572 |
| factor(X22)=1 | 1.16 | 109 | 1 | 0 | 0.3917 | 0.3384 | 0.4535 |
| factor(X22)=1 | 1.17 | 108 | 2 | 0 | 0.3845 | 0.3314 | 0.4461 |
| factor(X22)=1 | 1.18 | 106 | 1 | 0 | 0.3809 | 0.3279 | 0.4424 |
| factor(X22)=1 | 1.2 | 105 | 2 | 0 | 0.3736 | 0.3209 | 0.4350 |
| factor(X22)=1 | 1.25 | 103 | 2 | 0 | 0.3663 | 0.3139 | 0.4276 |
| factor(X22)=1 | 1.3 | 101 | 1 | 0 | 0.3627 | 0.3104 | 0.4239 |
| factor(X22)=1 | 1.35 | 100 | 2 | 0 | 0.3555 | 0.3034 | 0.4164 |
| factor(X22)=1 | 1.36 | 98 | 1 | 0 | 0.3518 | 0.3000 | 0.4127 |
| factor(X22)=1 | 1.38 | 97 | 2 | 0 | 0.3446 | 0.2930 | 0.4052 |
| factor(X22)=1 | 1.39 | 95 | 2 | 1 | 0.3373 | 0.2861 | 0.3978 |
| factor(X22)=1 | 1.42 | 92 | 1 | 0 | 0.3337 | 0.2826 | 0.3940 |
| factor(X22)=1 | 1.45 | 91 | 1 | 0 | 0.3300 | 0.2791 | 0.3902 |
| factor(X22)=1 | 1.5 | 90 | 2 | 0 | 0.3227 | 0.2721 | 0.3826 |
| factor(X22)=1 | 1.55 | 88 | 2 | 0 | 0.3153 | 0.2651 | 0.3750 |
| factor(X22)=1 | 1.58 | 86 | 1 | 0 | 0.3117 | 0.2616 | 0.3712 |
| factor(X22)=1 | 1.72 | 85 | 1 | 0 | 0.3080 | 0.2582 | 0.3674 |
| factor(X22)=1 | 1.74 | 84 | 1 | 0 | 0.3043 | 0.2547 | 0.3636 |
| factor(X22)=1 | 1.79 | 83 | 1 | 0 | 0.3007 | 0.2512 | 0.3598 |
| factor(X22)=1 | 1.92 | 82 | 1 | 0 | 0.2970 | 0.2478 | 0.3560 |
| factor(X22)=1 | 1.98 | 81 | 4 | 0 | 0.2823 | 0.2340 | 0.3407 |
| factor(X22)=1 | 2.09 | 77 | 1 | 0 | 0.2787 | 0.2305 | 0.3368 |
| factor(X22)=1 | 2.26 | 76 | 1 | 0 | 0.2750 | 0.2271 | 0.3330 |
| factor(X22)=1 | 2.32 | 75 | 0 | 1 | 0.2750 | 0.2271 | 0.3330 |
| factor(X22)=1 | 2.53 | 74 | 1 | 0 | 0.2713 | 0.2236 | 0.3291 |
| factor(X22)=1 | 2.71 | 73 | 0 | 1 | 0.2713 | 0.2236 | 0.3291 |
| factor(X22)=1 | 2.72 | 72 | 3 | 0 | 0.2600 | 0.2130 | 0.3173 |
| factor(X22)=1 | 2.83 | 69 | 1 | 0 | 0.2562 | 0.2095 | 0.3133 |
| factor(X22)=1 | 2.95 | 68 | 0 | 1 | 0.2562 | 0.2095 | 0.3133 |
| factor(X22)=1 | 2.99 | 67 | 1 | 0 | 0.2524 | 0.2060 | 0.3093 |
| factor(X22)=1 | 3 | 66 | 0 | 1 | 0.2524 | 0.2060 | 0.3093 |
| factor(X22)=1 | 3.05 | 65 | 0 | 1 | 0.2524 | 0.2060 | 0.3093 |
| factor(X22)=1 | 3.18 | 64 | 0 | 1 | 0.2524 | 0.2060 | 0.3093 |
| factor(X22)=1 | 3.25 | 63 | 0 | 1 | 0.2524 | 0.2060 | 0.3093 |
| factor(X22)=1 | 3.39 | 62 | 2 | 0 | 0.2442 | 0.1983 | 0.3008 |
| factor(X22)=1 | 3.53 | 60 | 0 | 1 | 0.2442 | 0.1983 | 0.3008 |
| factor(X22)=1 | 3.68 | 59 | 0 | 1 | 0.2442 | 0.1983 | 0.3008 |
| factor(X22)=1 | 3.87 | 58 | 0 | 1 | 0.2442 | 0.1983 | 0.3008 |
| factor(X22)=1 | 3.88 | 57 | 0 | 1 | 0.2442 | 0.1983 | 0.3008 |
| factor(X22)=1 | 4.04 | 56 | 0 | 1 | 0.2442 | 0.1983 | 0.3008 |
| factor(X22)=1 | 4.16 | 55 | 0 | 1 | 0.2442 | 0.1983 | 0.3008 |
| factor(X22)=1 | 4.22 | 54 | 0 | 1 | 0.2442 | 0.1983 | 0.3008 |
| factor(X22)=1 | 4.41 | 53 | 0 | 1 | 0.2442 | 0.1983 | 0.3008 |
| factor(X22)=1 | 4.44 | 52 | 0 | 1 | 0.2442 | 0.1983 | 0.3008 |
| factor(X22)=1 | 4.47 | 51 | 0 | 1 | 0.2442 | 0.1983 | 0.3008 |
| factor(X22)=1 | 4.48 | 50 | 0 | 1 | 0.2442 | 0.1983 | 0.3008 |
| factor(X22)=1 | 4.53 | 49 | 0 | 2 | 0.2442 | 0.1983 | 0.3008 |
| factor(X22)=1 | 4.55 | 47 | 0 | 1 | 0.2442 | 0.1983 | 0.3008 |
| factor(X22)=1 | 4.6 | 46 | 0 | 1 | 0.2442 | 0.1983 | 0.3008 |
| factor(X22)=1 | 4.61 | 45 | 0 | 1 | 0.2442 | 0.1983 | 0.3008 |
| factor(X22)=1 | 4.65 | 44 | 0 | 1 | 0.2442 | 0.1983 | 0.3008 |
| factor(X22)=1 | 4.7 | 43 | 0 | 1 | 0.2442 | 0.1983 | 0.3008 |
| factor(X22)=1 | 4.81 | 42 | 0 | 1 | 0.2442 | 0.1983 | 0.3008 |
| factor(X22)=1 | 4.88 | 41 | 0 | 1 | 0.2442 | 0.1983 | 0.3008 |
| factor(X22)=1 | 4.89 | 40 | 0 | 1 | 0.2442 | 0.1983 | 0.3008 |
| factor(X22)=1 | 5 | 39 | 0 | 39 | 0.2442 | 0.1983 | 0.3008 |

|  |  |  |  |  |  |  |  |  |  |
| --- | --- | --- | --- | --- | --- | --- | --- | --- | --- |
| X22 | records | n.max | n.start | events | \*rmean | \*se(rmean) | median | 0.95LCL | 0.95UCL |
| factor(X22)=0 | 924 | 924 | 924 | 393 | 3.296 | 0.067 | NA | NA | NA |
| factor(X22)=1 | 281 | 281 | 281 | 210 | 1.796 | 0.115 | 0.84 | 0.75 | 0.98 |

Landmark analysis for time segment: EFS(years) > 5
Log rank test: implements the G-rho family of Harrington and Fleming (1982), with weights on each death of S(t)^rho, where S is the Kaplan-Meier estimate of survival. With rho = 0 this is the log-rank or Mantel-Haenszel test.

|  |  |  |  |
| --- | --- | --- | --- |
|  | N | Observed | Expected |
| factor(X22)=0 | 298 | 6 | 7.0011 |
| factor(X22)=1 | 39 | 2 | 0.9989 |

Chisq=
1.1534
on
1
degree of freedom, p=
0.2828
Survival table

|  |  |  |  |  |  |  |  |
| --- | --- | --- | --- | --- | --- | --- | --- |
|  | EFS(years) | N.Risk | N.Event | N.Censor | Survival | 95%CI Low | 95%CI Upp |
| factor(X22)=0 | 5.02 | 298 | 0 | 1 | 1.0000 | 1.0000 | 1.0000 |
| factor(X22)=0 | 5.03 | 297 | 0 | 1 | 1.0000 | 1.0000 | 1.0000 |
| factor(X22)=0 | 5.04 | 296 | 0 | 1 | 1.0000 | 1.0000 | 1.0000 |
| factor(X22)=0 | 5.05 | 295 | 0 | 2 | 1.0000 | 1.0000 | 1.0000 |
| factor(X22)=0 | 5.08 | 293 | 0 | 1 | 1.0000 | 1.0000 | 1.0000 |
| factor(X22)=0 | 5.1 | 292 | 0 | 1 | 1.0000 | 1.0000 | 1.0000 |
| factor(X22)=0 | 5.12 | 291 | 0 | 1 | 1.0000 | 1.0000 | 1.0000 |
| factor(X22)=0 | 5.14 | 290 | 0 | 1 | 1.0000 | 1.0000 | 1.0000 |
| factor(X22)=0 | 5.15 | 289 | 0 | 2 | 1.0000 | 1.0000 | 1.0000 |
| factor(X22)=0 | 5.16 | 287 | 0 | 1 | 1.0000 | 1.0000 | 1.0000 |
| factor(X22)=0 | 5.17 | 286 | 0 | 1 | 1.0000 | 1.0000 | 1.0000 |
| factor(X22)=0 | 5.18 | 285 | 0 | 3 | 1.0000 | 1.0000 | 1.0000 |
| factor(X22)=0 | 5.2 | 282 | 0 | 1 | 1.0000 | 1.0000 | 1.0000 |
| factor(X22)=0 | 5.22 | 281 | 0 | 1 | 1.0000 | 1.0000 | 1.0000 |
| factor(X22)=0 | 5.25 | 280 | 0 | 2 | 1.0000 | 1.0000 | 1.0000 |
| factor(X22)=0 | 5.26 | 278 | 0 | 1 | 1.0000 | 1.0000 | 1.0000 |
| factor(X22)=0 | 5.27 | 277 | 0 | 2 | 1.0000 | 1.0000 | 1.0000 |
| factor(X22)=0 | 5.29 | 275 | 0 | 2 | 1.0000 | 1.0000 | 1.0000 |
| factor(X22)=0 | 5.3 | 273 | 0 | 1 | 1.0000 | 1.0000 | 1.0000 |
| factor(X22)=0 | 5.31 | 272 | 0 | 1 | 1.0000 | 1.0000 | 1.0000 |
| factor(X22)=0 | 5.32 | 271 | 0 | 2 | 1.0000 | 1.0000 | 1.0000 |
| factor(X22)=0 | 5.33 | 269 | 0 | 2 | 1.0000 | 1.0000 | 1.0000 |
| factor(X22)=0 | 5.34 | 267 | 0 | 1 | 1.0000 | 1.0000 | 1.0000 |
| factor(X22)=0 | 5.35 | 266 | 0 | 6 | 1.0000 | 1.0000 | 1.0000 |
| factor(X22)=0 | 5.36 | 260 | 0 | 1 | 1.0000 | 1.0000 | 1.0000 |
| factor(X22)=0 | 5.37 | 259 | 0 | 2 | 1.0000 | 1.0000 | 1.0000 |
| factor(X22)=0 | 5.38 | 257 | 0 | 2 | 1.0000 | 1.0000 | 1.0000 |
| factor(X22)=0 | 5.39 | 255 | 0 | 1 | 1.0000 | 1.0000 | 1.0000 |
| factor(X22)=0 | 5.4 | 254 | 0 | 1 | 1.0000 | 1.0000 | 1.0000 |
| factor(X22)=0 | 5.41 | 253 | 0 | 2 | 1.0000 | 1.0000 | 1.0000 |
| factor(X22)=0 | 5.42 | 251 | 0 | 3 | 1.0000 | 1.0000 | 1.0000 |
| factor(X22)=0 | 5.43 | 248 | 0 | 1 | 1.0000 | 1.0000 | 1.0000 |
| factor(X22)=0 | 5.44 | 247 | 0 | 4 | 1.0000 | 1.0000 | 1.0000 |
| factor(X22)=0 | 5.45 | 243 | 0 | 1 | 1.0000 | 1.0000 | 1.0000 |
| factor(X22)=0 | 5.46 | 242 | 0 | 2 | 1.0000 | 1.0000 | 1.0000 |
| factor(X22)=0 | 5.48 | 240 | 0 | 2 | 1.0000 | 1.0000 | 1.0000 |
| factor(X22)=0 | 5.49 | 238 | 0 | 1 | 1.0000 | 1.0000 | 1.0000 |
| factor(X22)=0 | 5.5 | 237 | 0 | 1 | 1.0000 | 1.0000 | 1.0000 |
| factor(X22)=0 | 5.51 | 236 | 0 | 2 | 1.0000 | 1.0000 | 1.0000 |
| factor(X22)=0 | 5.52 | 234 | 0 | 2 | 1.0000 | 1.0000 | 1.0000 |
| factor(X22)=0 | 5.54 | 232 | 0 | 4 | 1.0000 | 1.0000 | 1.0000 |
| factor(X22)=0 | 5.55 | 228 | 0 | 1 | 1.0000 | 1.0000 | 1.0000 |
| factor(X22)=0 | 5.56 | 227 | 0 | 2 | 1.0000 | 1.0000 | 1.0000 |
| factor(X22)=0 | 5.57 | 225 | 0 | 1 | 1.0000 | 1.0000 | 1.0000 |
| factor(X22)=0 | 5.58 | 224 | 1 | 1 | 0.9955 | 0.9868 | 1.0000 |
| factor(X22)=0 | 5.6 | 222 | 0 | 1 | 0.9955 | 0.9868 | 1.0000 |
| factor(X22)=0 | 5.61 | 221 | 0 | 1 | 0.9955 | 0.9868 | 1.0000 |
| factor(X22)=0 | 5.63 | 220 | 0 | 2 | 0.9955 | 0.9868 | 1.0000 |
| factor(X22)=0 | 5.65 | 218 | 0 | 1 | 0.9955 | 0.9868 | 1.0000 |
| factor(X22)=0 | 5.66 | 217 | 0 | 1 | 0.9955 | 0.9868 | 1.0000 |
| factor(X22)=0 | 5.67 | 216 | 0 | 3 | 0.9955 | 0.9868 | 1.0000 |
| factor(X22)=0 | 5.7 | 213 | 0 | 3 | 0.9955 | 0.9868 | 1.0000 |
| factor(X22)=0 | 5.71 | 210 | 0 | 2 | 0.9955 | 0.9868 | 1.0000 |
| factor(X22)=0 | 5.72 | 208 | 0 | 1 | 0.9955 | 0.9868 | 1.0000 |
| factor(X22)=0 | 5.73 | 207 | 0 | 2 | 0.9955 | 0.9868 | 1.0000 |
| factor(X22)=0 | 5.74 | 205 | 0 | 1 | 0.9955 | 0.9868 | 1.0000 |
| factor(X22)=0 | 5.75 | 204 | 0 | 2 | 0.9955 | 0.9868 | 1.0000 |
| factor(X22)=0 | 5.77 | 202 | 0 | 1 | 0.9955 | 0.9868 | 1.0000 |
| factor(X22)=0 | 5.78 | 201 | 0 | 2 | 0.9955 | 0.9868 | 1.0000 |
| factor(X22)=0 | 5.79 | 199 | 0 | 1 | 0.9955 | 0.9868 | 1.0000 |
| factor(X22)=0 | 5.8 | 198 | 0 | 1 | 0.9955 | 0.9868 | 1.0000 |
| factor(X22)=0 | 5.81 | 197 | 0 | 2 | 0.9955 | 0.9868 | 1.0000 |
| factor(X22)=0 | 5.84 | 195 | 0 | 2 | 0.9955 | 0.9868 | 1.0000 |
| factor(X22)=0 | 5.85 | 193 | 0 | 1 | 0.9955 | 0.9868 | 1.0000 |
| factor(X22)=0 | 5.88 | 192 | 0 | 1 | 0.9955 | 0.9868 | 1.0000 |
| factor(X22)=0 | 5.89 | 191 | 0 | 1 | 0.9955 | 0.9868 | 1.0000 |
| factor(X22)=0 | 5.92 | 190 | 0 | 1 | 0.9955 | 0.9868 | 1.0000 |
| factor(X22)=0 | 5.93 | 189 | 0 | 1 | 0.9955 | 0.9868 | 1.0000 |
| factor(X22)=0 | 5.94 | 188 | 0 | 2 | 0.9955 | 0.9868 | 1.0000 |
| factor(X22)=0 | 5.95 | 186 | 0 | 3 | 0.9955 | 0.9868 | 1.0000 |
| factor(X22)=0 | 5.96 | 183 | 0 | 1 | 0.9955 | 0.9868 | 1.0000 |
| factor(X22)=0 | 5.98 | 182 | 0 | 1 | 0.9955 | 0.9868 | 1.0000 |
| factor(X22)=0 | 6 | 181 | 0 | 6 | 0.9955 | 0.9868 | 1.0000 |
| factor(X22)=0 | 6.01 | 175 | 0 | 1 | 0.9955 | 0.9868 | 1.0000 |
| factor(X22)=0 | 6.02 | 174 | 0 | 1 | 0.9955 | 0.9868 | 1.0000 |
| factor(X22)=0 | 6.04 | 173 | 0 | 1 | 0.9955 | 0.9868 | 1.0000 |
| factor(X22)=0 | 6.07 | 172 | 0 | 1 | 0.9955 | 0.9868 | 1.0000 |
| factor(X22)=0 | 6.08 | 171 | 0 | 1 | 0.9955 | 0.9868 | 1.0000 |
| factor(X22)=0 | 6.12 | 170 | 0 | 2 | 0.9955 | 0.9868 | 1.0000 |
| factor(X22)=0 | 6.15 | 168 | 0 | 3 | 0.9955 | 0.9868 | 1.0000 |
| factor(X22)=0 | 6.16 | 165 | 0 | 1 | 0.9955 | 0.9868 | 1.0000 |
| factor(X22)=0 | 6.17 | 164 | 0 | 1 | 0.9955 | 0.9868 | 1.0000 |
| factor(X22)=0 | 6.18 | 163 | 0 | 2 | 0.9955 | 0.9868 | 1.0000 |
| factor(X22)=0 | 6.22 | 161 | 0 | 1 | 0.9955 | 0.9868 | 1.0000 |
| factor(X22)=0 | 6.25 | 160 | 0 | 2 | 0.9955 | 0.9868 | 1.0000 |
| factor(X22)=0 | 6.27 | 158 | 0 | 1 | 0.9955 | 0.9868 | 1.0000 |
| factor(X22)=0 | 6.28 | 157 | 0 | 1 | 0.9955 | 0.9868 | 1.0000 |
| factor(X22)=0 | 6.29 | 156 | 0 | 1 | 0.9955 | 0.9868 | 1.0000 |
| factor(X22)=0 | 6.32 | 155 | 0 | 1 | 0.9955 | 0.9868 | 1.0000 |
| factor(X22)=0 | 6.35 | 154 | 0 | 2 | 0.9955 | 0.9868 | 1.0000 |
| factor(X22)=0 | 6.36 | 152 | 0 | 1 | 0.9955 | 0.9868 | 1.0000 |
| factor(X22)=0 | 6.37 | 151 | 0 | 2 | 0.9955 | 0.9868 | 1.0000 |
| factor(X22)=0 | 6.38 | 149 | 0 | 1 | 0.9955 | 0.9868 | 1.0000 |
| factor(X22)=0 | 6.39 | 148 | 0 | 3 | 0.9955 | 0.9868 | 1.0000 |
| factor(X22)=0 | 6.4 | 145 | 0 | 2 | 0.9955 | 0.9868 | 1.0000 |
| factor(X22)=0 | 6.42 | 143 | 0 | 3 | 0.9955 | 0.9868 | 1.0000 |
| factor(X22)=0 | 6.45 | 140 | 3 | 1 | 0.9742 | 0.9492 | 0.9999 |
| factor(X22)=0 | 6.46 | 136 | 0 | 2 | 0.9742 | 0.9492 | 0.9999 |
| factor(X22)=0 | 6.47 | 134 | 0 | 3 | 0.9742 | 0.9492 | 0.9999 |
| factor(X22)=0 | 6.48 | 131 | 0 | 2 | 0.9742 | 0.9492 | 0.9999 |
| factor(X22)=0 | 6.49 | 129 | 0 | 1 | 0.9742 | 0.9492 | 0.9999 |
| factor(X22)=0 | 6.5 | 128 | 0 | 2 | 0.9742 | 0.9492 | 0.9999 |
| factor(X22)=0 | 6.51 | 126 | 0 | 2 | 0.9742 | 0.9492 | 0.9999 |
| factor(X22)=0 | 6.52 | 124 | 0 | 1 | 0.9742 | 0.9492 | 0.9999 |
| factor(X22)=0 | 6.53 | 123 | 0 | 1 | 0.9742 | 0.9492 | 0.9999 |
| factor(X22)=0 | 6.54 | 122 | 0 | 1 | 0.9742 | 0.9492 | 0.9999 |
| factor(X22)=0 | 6.55 | 121 | 0 | 1 | 0.9742 | 0.9492 | 0.9999 |
| factor(X22)=0 | 6.56 | 120 | 1 | 0 | 0.9661 | 0.9368 | 0.9963 |
| factor(X22)=0 | 6.57 | 119 | 0 | 2 | 0.9661 | 0.9368 | 0.9963 |
| factor(X22)=0 | 6.58 | 117 | 0 | 2 | 0.9661 | 0.9368 | 0.9963 |
| factor(X22)=0 | 6.59 | 115 | 0 | 1 | 0.9661 | 0.9368 | 0.9963 |
| factor(X22)=0 | 6.6 | 114 | 0 | 1 | 0.9661 | 0.9368 | 0.9963 |
| factor(X22)=0 | 6.63 | 113 | 0 | 1 | 0.9661 | 0.9368 | 0.9963 |
| factor(X22)=0 | 6.64 | 112 | 0 | 2 | 0.9661 | 0.9368 | 0.9963 |
| factor(X22)=0 | 6.65 | 110 | 0 | 3 | 0.9661 | 0.9368 | 0.9963 |
| factor(X22)=0 | 6.68 | 107 | 0 | 1 | 0.9661 | 0.9368 | 0.9963 |
| factor(X22)=0 | 6.69 | 106 | 0 | 1 | 0.9661 | 0.9368 | 0.9963 |
| factor(X22)=0 | 6.73 | 105 | 0 | 1 | 0.9661 | 0.9368 | 0.9963 |
| factor(X22)=0 | 6.75 | 104 | 0 | 3 | 0.9661 | 0.9368 | 0.9963 |
| factor(X22)=0 | 6.77 | 101 | 0 | 1 | 0.9661 | 0.9368 | 0.9963 |
| factor(X22)=0 | 6.78 | 100 | 0 | 1 | 0.9661 | 0.9368 | 0.9963 |
| factor(X22)=0 | 6.79 | 99 | 0 | 1 | 0.9661 | 0.9368 | 0.9963 |
| factor(X22)=0 | 6.81 | 98 | 0 | 2 | 0.9661 | 0.9368 | 0.9963 |
| factor(X22)=0 | 6.85 | 96 | 0 | 1 | 0.9661 | 0.9368 | 0.9963 |
| factor(X22)=0 | 6.89 | 95 | 0 | 2 | 0.9661 | 0.9368 | 0.9963 |
| factor(X22)=0 | 6.91 | 93 | 0 | 1 | 0.9661 | 0.9368 | 0.9963 |
| factor(X22)=0 | 6.92 | 92 | 0 | 1 | 0.9661 | 0.9368 | 0.9963 |
| factor(X22)=0 | 6.94 | 91 | 0 | 1 | 0.9661 | 0.9368 | 0.9963 |
| factor(X22)=0 | 6.96 | 90 | 0 | 4 | 0.9661 | 0.9368 | 0.9963 |
| factor(X22)=0 | 6.98 | 86 | 0 | 1 | 0.9661 | 0.9368 | 0.9963 |
| factor(X22)=0 | 7 | 85 | 0 | 3 | 0.9661 | 0.9368 | 0.9963 |
| factor(X22)=0 | 7.08 | 82 | 0 | 1 | 0.9661 | 0.9368 | 0.9963 |
| factor(X22)=0 | 7.14 | 81 | 0 | 1 | 0.9661 | 0.9368 | 0.9963 |
| factor(X22)=0 | 7.16 | 80 | 0 | 1 | 0.9661 | 0.9368 | 0.9963 |
| factor(X22)=0 | 7.2 | 79 | 0 | 1 | 0.9661 | 0.9368 | 0.9963 |
| factor(X22)=0 | 7.21 | 78 | 0 | 1 | 0.9661 | 0.9368 | 0.9963 |
| factor(X22)=0 | 7.26 | 77 | 0 | 3 | 0.9661 | 0.9368 | 0.9963 |
| factor(X22)=0 | 7.29 | 74 | 0 | 1 | 0.9661 | 0.9368 | 0.9963 |
| factor(X22)=0 | 7.35 | 73 | 0 | 2 | 0.9661 | 0.9368 | 0.9963 |
| factor(X22)=0 | 7.36 | 71 | 0 | 4 | 0.9661 | 0.9368 | 0.9963 |
| factor(X22)=0 | 7.37 | 67 | 0 | 1 | 0.9661 | 0.9368 | 0.9963 |
| factor(X22)=0 | 7.38 | 66 | 0 | 1 | 0.9661 | 0.9368 | 0.9963 |
| factor(X22)=0 | 7.39 | 65 | 0 | 2 | 0.9661 | 0.9368 | 0.9963 |
| factor(X22)=0 | 7.4 | 63 | 0 | 1 | 0.9661 | 0.9368 | 0.9963 |
| factor(X22)=0 | 7.41 | 62 | 0 | 1 | 0.9661 | 0.9368 | 0.9963 |
| factor(X22)=0 | 7.42 | 61 | 0 | 1 | 0.9661 | 0.9368 | 0.9963 |
| factor(X22)=0 | 7.45 | 60 | 0 | 1 | 0.9661 | 0.9368 | 0.9963 |
| factor(X22)=0 | 7.47 | 59 | 0 | 1 | 0.9661 | 0.9368 | 0.9963 |
| factor(X22)=0 | 7.48 | 58 | 0 | 2 | 0.9661 | 0.9368 | 0.9963 |
| factor(X22)=0 | 7.5 | 56 | 0 | 1 | 0.9661 | 0.9368 | 0.9963 |
| factor(X22)=0 | 7.53 | 55 | 0 | 1 | 0.9661 | 0.9368 | 0.9963 |
| factor(X22)=0 | 7.54 | 54 | 0 | 1 | 0.9661 | 0.9368 | 0.9963 |
| factor(X22)=0 | 7.55 | 53 | 0 | 1 | 0.9661 | 0.9368 | 0.9963 |
| factor(X22)=0 | 7.56 | 52 | 0 | 2 | 0.9661 | 0.9368 | 0.9963 |
| factor(X22)=0 | 7.58 | 50 | 0 | 4 | 0.9661 | 0.9368 | 0.9963 |
| factor(X22)=0 | 7.59 | 46 | 0 | 1 | 0.9661 | 0.9368 | 0.9963 |
| factor(X22)=0 | 7.6 | 45 | 0 | 2 | 0.9661 | 0.9368 | 0.9963 |
| factor(X22)=0 | 7.61 | 43 | 0 | 1 | 0.9661 | 0.9368 | 0.9963 |
| factor(X22)=0 | 7.62 | 42 | 0 | 1 | 0.9661 | 0.9368 | 0.9963 |
| factor(X22)=0 | 7.67 | 41 | 0 | 1 | 0.9661 | 0.9368 | 0.9963 |
| factor(X22)=0 | 7.68 | 40 | 0 | 1 | 0.9661 | 0.9368 | 0.9963 |
| factor(X22)=0 | 7.69 | 39 | 0 | 2 | 0.9661 | 0.9368 | 0.9963 |
| factor(X22)=0 | 7.73 | 37 | 0 | 2 | 0.9661 | 0.9368 | 0.9963 |
| factor(X22)=0 | 7.74 | 35 | 0 | 3 | 0.9661 | 0.9368 | 0.9963 |
| factor(X22)=0 | 7.81 | 32 | 0 | 1 | 0.9661 | 0.9368 | 0.9963 |
| factor(X22)=0 | 7.82 | 31 | 0 | 1 | 0.9661 | 0.9368 | 0.9963 |
| factor(X22)=0 | 7.94 | 30 | 0 | 2 | 0.9661 | 0.9368 | 0.9963 |
| factor(X22)=0 | 7.98 | 28 | 0 | 1 | 0.9661 | 0.9368 | 0.9963 |
| factor(X22)=0 | 7.99 | 27 | 1 | 1 | 0.9303 | 0.8587 | 1.0000 |
| factor(X22)=0 | 8.01 | 25 | 0 | 1 | 0.9303 | 0.8587 | 1.0000 |
| factor(X22)=0 | 8.07 | 24 | 0 | 1 | 0.9303 | 0.8587 | 1.0000 |
| factor(X22)=0 | 8.15 | 23 | 0 | 1 | 0.9303 | 0.8587 | 1.0000 |
| factor(X22)=0 | 8.17 | 22 | 0 | 1 | 0.9303 | 0.8587 | 1.0000 |
| factor(X22)=0 | 8.21 | 21 | 0 | 1 | 0.9303 | 0.8587 | 1.0000 |
| factor(X22)=0 | 8.26 | 20 | 0 | 1 | 0.9303 | 0.8587 | 1.0000 |
| factor(X22)=0 | 8.27 | 19 | 0 | 1 | 0.9303 | 0.8587 | 1.0000 |
| factor(X22)=0 | 8.28 | 18 | 0 | 1 | 0.9303 | 0.8587 | 1.0000 |
| factor(X22)=0 | 8.29 | 17 | 0 | 1 | 0.9303 | 0.8587 | 1.0000 |
| factor(X22)=0 | 8.48 | 16 | 0 | 1 | 0.9303 | 0.8587 | 1.0000 |
| factor(X22)=0 | 8.49 | 15 | 0 | 2 | 0.9303 | 0.8587 | 1.0000 |
| factor(X22)=0 | 8.5 | 13 | 0 | 1 | 0.9303 | 0.8587 | 1.0000 |
| factor(X22)=0 | 8.53 | 12 | 0 | 1 | 0.9303 | 0.8587 | 1.0000 |
| factor(X22)=0 | 8.72 | 11 | 0 | 1 | 0.9303 | 0.8587 | 1.0000 |
| factor(X22)=0 | 8.84 | 10 | 0 | 1 | 0.9303 | 0.8587 | 1.0000 |
| factor(X22)=0 | 8.88 | 9 | 0 | 2 | 0.9303 | 0.8587 | 1.0000 |
| factor(X22)=0 | 9.04 | 7 | 0 | 1 | 0.9303 | 0.8587 | 1.0000 |
| factor(X22)=0 | 9.06 | 6 | 0 | 2 | 0.9303 | 0.8587 | 1.0000 |
| factor(X22)=0 | 9.08 | 4 | 0 | 1 | 0.9303 | 0.8587 | 1.0000 |
| factor(X22)=0 | 9.26 | 3 | 0 | 1 | 0.9303 | 0.8587 | 1.0000 |
| factor(X22)=0 | 9.61 | 2 | 0 | 1 | 0.9303 | 0.8587 | 1.0000 |
| factor(X22)=0 | 9.95 | 1 | 0 | 1 | 0.9303 | 0.8587 | 1.0000 |
| factor(X22)=1 | 5.12 | 39 | 1 | 0 | 0.9744 | 0.9260 | 1.0000 |
| factor(X22)=1 | 5.2 | 38 | 0 | 1 | 0.9744 | 0.9260 | 1.0000 |
| factor(X22)=1 | 5.26 | 37 | 0 | 1 | 0.9744 | 0.9260 | 1.0000 |
| factor(X22)=1 | 5.33 | 36 | 0 | 1 | 0.9744 | 0.9260 | 1.0000 |
| factor(X22)=1 | 5.34 | 35 | 0 | 1 | 0.9744 | 0.9260 | 1.0000 |
| factor(X22)=1 | 5.36 | 34 | 0 | 1 | 0.9744 | 0.9260 | 1.0000 |
| factor(X22)=1 | 5.38 | 33 | 0 | 1 | 0.9744 | 0.9260 | 1.0000 |
| factor(X22)=1 | 5.4 | 32 | 0 | 1 | 0.9744 | 0.9260 | 1.0000 |
| factor(X22)=1 | 5.42 | 31 | 0 | 1 | 0.9744 | 0.9260 | 1.0000 |
| factor(X22)=1 | 5.48 | 30 | 0 | 1 | 0.9744 | 0.9260 | 1.0000 |
| factor(X22)=1 | 5.55 | 29 | 0 | 1 | 0.9744 | 0.9260 | 1.0000 |
| factor(X22)=1 | 5.81 | 28 | 1 | 0 | 0.9396 | 0.8608 | 1.0000 |
| factor(X22)=1 | 5.89 | 27 | 0 | 1 | 0.9396 | 0.8608 | 1.0000 |
| factor(X22)=1 | 6.04 | 26 | 0 | 1 | 0.9396 | 0.8608 | 1.0000 |
| factor(X22)=1 | 6.16 | 25 | 0 | 1 | 0.9396 | 0.8608 | 1.0000 |
| factor(X22)=1 | 6.19 | 24 | 0 | 1 | 0.9396 | 0.8608 | 1.0000 |
| factor(X22)=1 | 6.2 | 23 | 0 | 1 | 0.9396 | 0.8608 | 1.0000 |
| factor(X22)=1 | 6.38 | 22 | 0 | 1 | 0.9396 | 0.8608 | 1.0000 |
| factor(X22)=1 | 6.39 | 21 | 0 | 1 | 0.9396 | 0.8608 | 1.0000 |
| factor(X22)=1 | 6.4 | 20 | 0 | 1 | 0.9396 | 0.8608 | 1.0000 |
| factor(X22)=1 | 6.51 | 19 | 0 | 1 | 0.9396 | 0.8608 | 1.0000 |
| factor(X22)=1 | 6.56 | 18 | 0 | 1 | 0.9396 | 0.8608 | 1.0000 |
| factor(X22)=1 | 6.79 | 17 | 0 | 1 | 0.9396 | 0.8608 | 1.0000 |
| factor(X22)=1 | 6.87 | 16 | 0 | 1 | 0.9396 | 0.8608 | 1.0000 |
| factor(X22)=1 | 7.12 | 15 | 0 | 1 | 0.9396 | 0.8608 | 1.0000 |
| factor(X22)=1 | 7.27 | 14 | 0 | 1 | 0.9396 | 0.8608 | 1.0000 |
| factor(X22)=1 | 7.36 | 13 | 0 | 2 | 0.9396 | 0.8608 | 1.0000 |
| factor(X22)=1 | 7.46 | 11 | 0 | 1 | 0.9396 | 0.8608 | 1.0000 |
| factor(X22)=1 | 7.48 | 10 | 0 | 1 | 0.9396 | 0.8608 | 1.0000 |
| factor(X22)=1 | 7.72 | 9 | 0 | 1 | 0.9396 | 0.8608 | 1.0000 |
| factor(X22)=1 | 7.74 | 8 | 0 | 2 | 0.9396 | 0.8608 | 1.0000 |
| factor(X22)=1 | 7.9 | 6 | 0 | 1 | 0.9396 | 0.8608 | 1.0000 |
| factor(X22)=1 | 8.06 | 5 | 0 | 1 | 0.9396 | 0.8608 | 1.0000 |
| factor(X22)=1 | 8.1 | 4 | 0 | 1 | 0.9396 | 0.8608 | 1.0000 |
| factor(X22)=1 | 8.36 | 3 | 0 | 1 | 0.9396 | 0.8608 | 1.0000 |
| factor(X22)=1 | 8.69 | 2 | 0 | 1 | 0.9396 | 0.8608 | 1.0000 |
| factor(X22)=1 | 8.88 | 1 | 0 | 1 | 0.9396 | 0.8608 | 1.0000 |

|  |  |  |  |  |  |  |  |  |  |
| --- | --- | --- | --- | --- | --- | --- | --- | --- | --- |
| X22 | records | n.max | n.start | events | \*rmean | \*se(rmean) | median | 0.95LCL | 0.95UCL |
| factor(X22)=0 | 298 | 298 | 298 | 6 | 9.26 | 0.067 | NA | NA | NA |
| factor(X22)=1 | 39 | 39 | 39 | 2 | 9.179 | 0.162 | NA | NA | NA |

Created by EmpowerStats (www.empowerstats.com) and R on 2025-10-07
