# Supplementary material for: Optimal minimal residual disease threshold in pediatric acute myeloid leukemia: A retrospective cohort study based on the TARGET database
Source: PLoS Med. 2026 May 8;23(5):e1005088. doi: 10.1371/journal.pmed.1005088 (PMC13155632; doi:10.1371/journal.pmed.1005088)
Supplement: S1 Code — (ZIP) [file pmed.1005088.s002.zip › S2 code/PROJ8_6_tbl/PROJ8_6_tbl.htm]

## µ¥ÒòËØ·ÖÎö

|  |  |  |
| --- | --- | --- |
|  | Statistics | First Event |
| MRD % at end of course 1 | 3.1 ± 11.6 | 1.0 (1.0, 1.1) <0.001 |
| MRD % at end of course 1 ·Ö×é |  |  |
| <0.05 | 906 (75.2%) | 1.0 |
| >=0.05 | 299 (24.8%) | 2.8 (2.3, 3.3) <0.001 |
| MRD at end of course 1 |  |  |
| Negative | 924 (76.7%) | 1.0 |
| Positive | 281 (23.3%) | 2.7 (2.3, 3.2) <0.001 |
| MRD at end of course 2 |  |  |
| Negative | 1073 (89.0%) | 1.0 |
| Positive | 132 (11.0%) | 3.7 (3.0, 4.6) <0.001 |
| MRD % at end of course 2 ·Ö×é |  |  |
| <0.05 | 1068 (88.6%) | 1.0 |
| >=0.05 | 137 (11.4%) | 3.7 (3.0, 4.6) <0.001 |

±íÖÐÊý¾Ý£ºHR (95% CI) Pvalue \*P<0.05 \*\*P<0.01 \*\*\*P<0.001
½á¹û±äÁ¿: First Event
±©Â¶±äÁ¿: MRD % at end of course 1; MRD % at end of course 1 ·Ö×é; MRD at end of course 1; MRD at end of course 2; MRD % at end of course 2 ·Ö×é
µ÷Õû±äÁ¿: None
Cox model Ê±¼ä±äÁ¿:
X28
´Ë±íÓÃÒ×õÍ³¼ÆÈí¼þ (www.empowerstats.com) ºÍRÈí¼þÉú³É£¬Éú³ÉÈÕÆÚ£º 2025-10-06
¸÷Ä£ÐÍËùÓÃµÄÑù±¾Á¿

|  |  |
| --- | --- |
| Exposure | First Event |
| MRD % at end of course 1 | 1205 |
| MRD % at end of course 1 ·Ö×é | 1205 |
| MRD at end of course 1 | 1205 |
| MRD at end of course 2 | 1205 |
| MRD % at end of course 2 ·Ö×é | 1205 |
