# Supplementary figures and images for: Optimal minimal residual disease threshold in pediatric acute myeloid leukemia: A retrospective cohort study based on the TARGET database (part 2 of 2)
Source: PLoS Med. 2026 May 8;23(5):e1005088. doi: 10.1371/journal.pmed.1005088 (PMC13155632; doi:10.1371/journal.pmed.1005088)

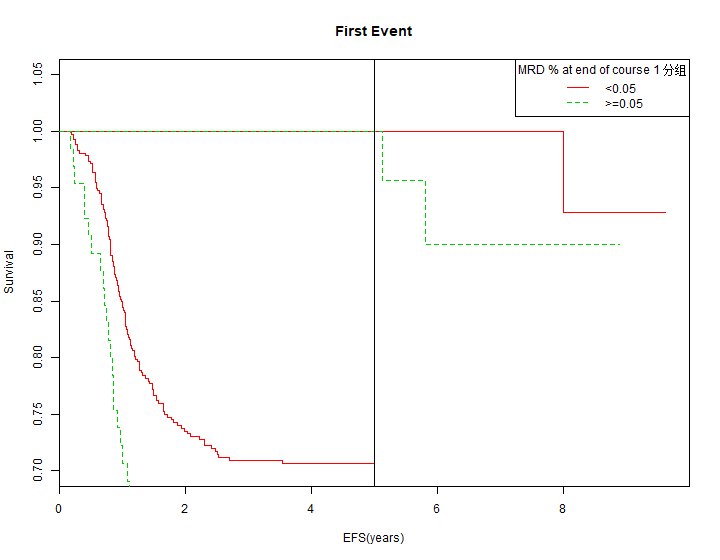

Supplement: S1 Code — (ZIP) [file pmed.1005088.s002.zip › S2 code/PROJ8_12_tbl1/PROJ8_12_tbl1.png]

## First Event

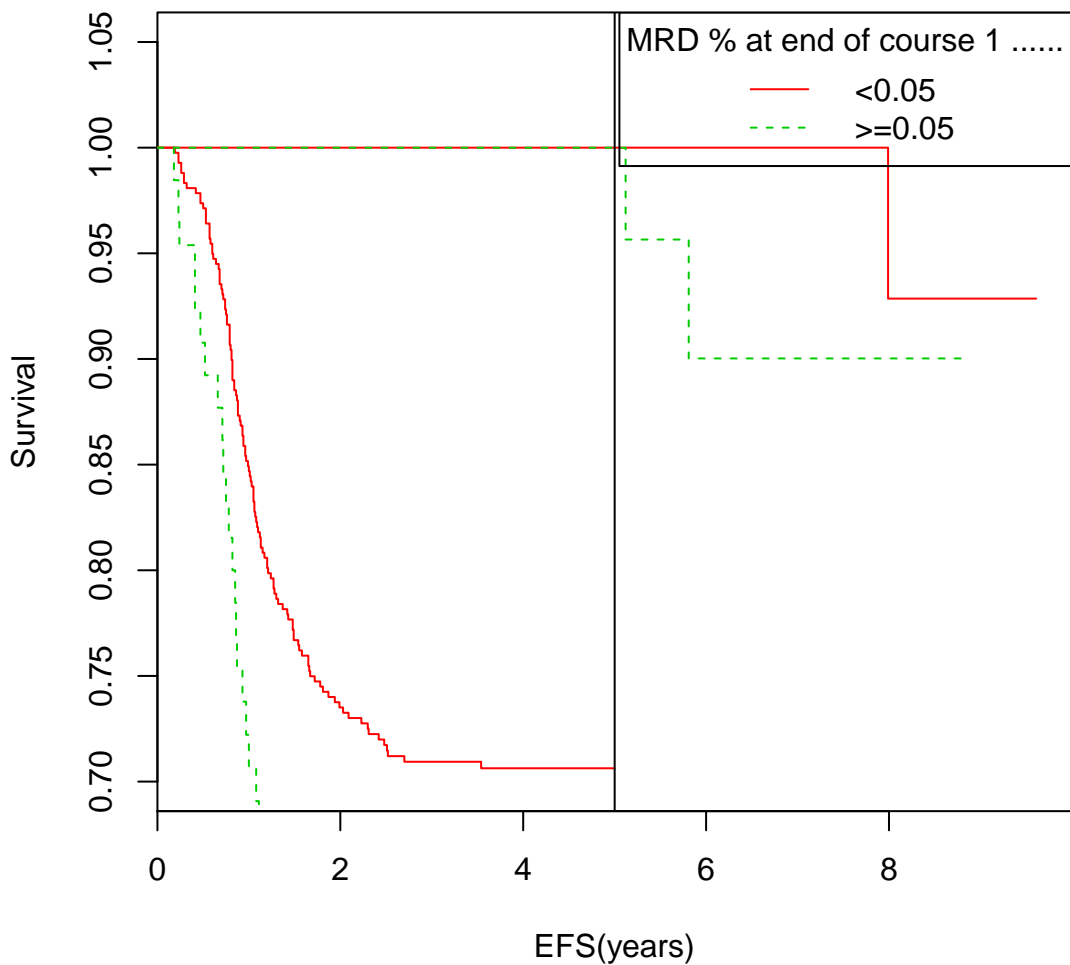

Supplement: S1 Code — (ZIP) [file pmed.1005088.s002.zip › S2 code/PROJ8_12_tbl1/PROJ8_12_tbl1.pdf]

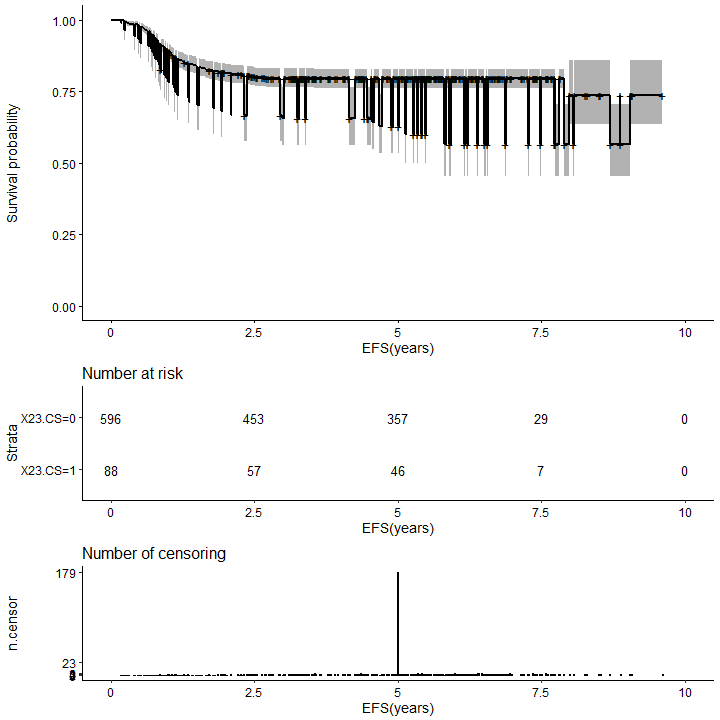

Supplement: S1 Code — (ZIP) [file pmed.1005088.s002.zip › S2 code/PROJ8_12_tbl1/PROJ8_12_tbl1_0.png]

MRD at end of course 1    —    <0.1    - -    =0.1

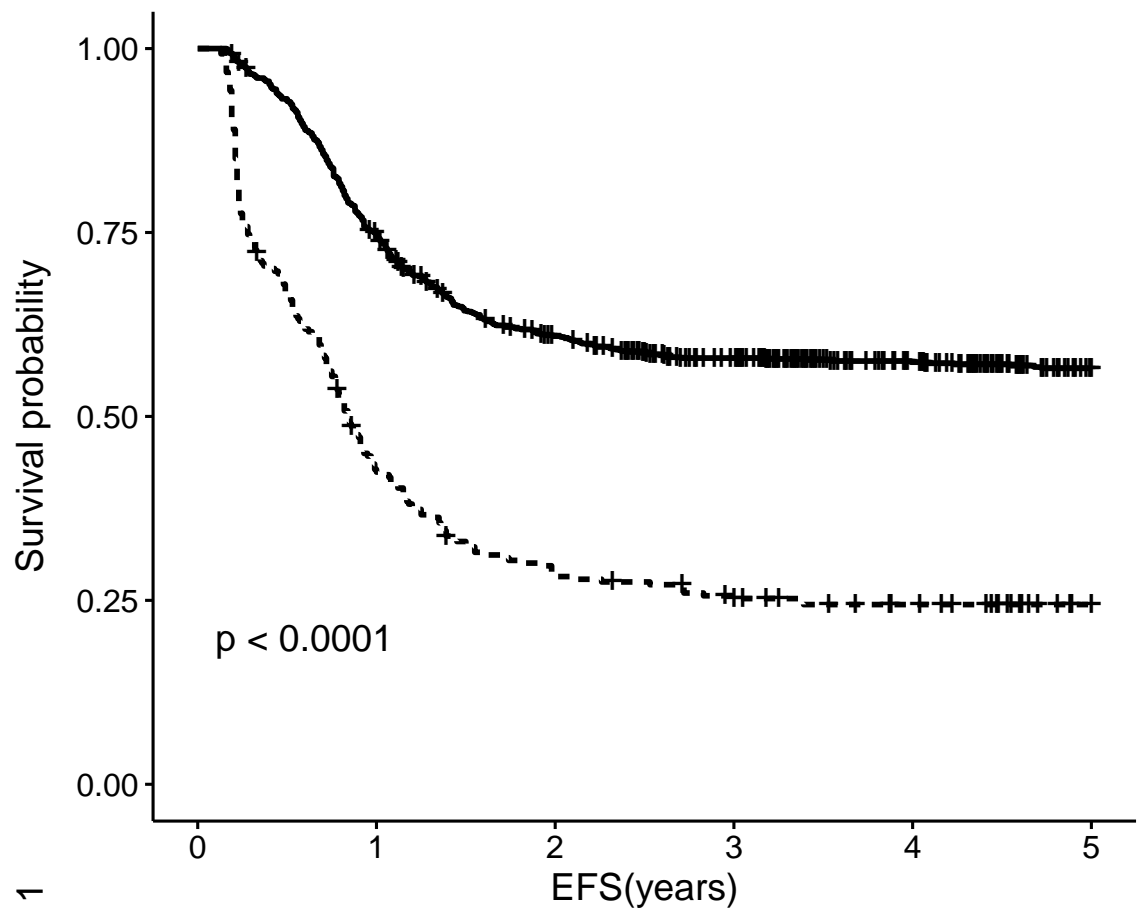

MRD at end of course 1

Number at risk

|      | 0   | 1   | 2   | 3   | 4   | 5   |
|------|-----|-----|-----|-----|-----|-----|
| <0.1 | 924 | 688 | 539 | 472 | 382 | 299 |
| =0.1 | 281 | 118 | 77  | 66  | 56  | 39  |

EFS(years)

Supplement: S1 Code — (ZIP) [file pmed.1005088.s002.zip › S2 code/PROJ8_4_tbl/PROJ8_4_tbl_seg1_b.pdf]

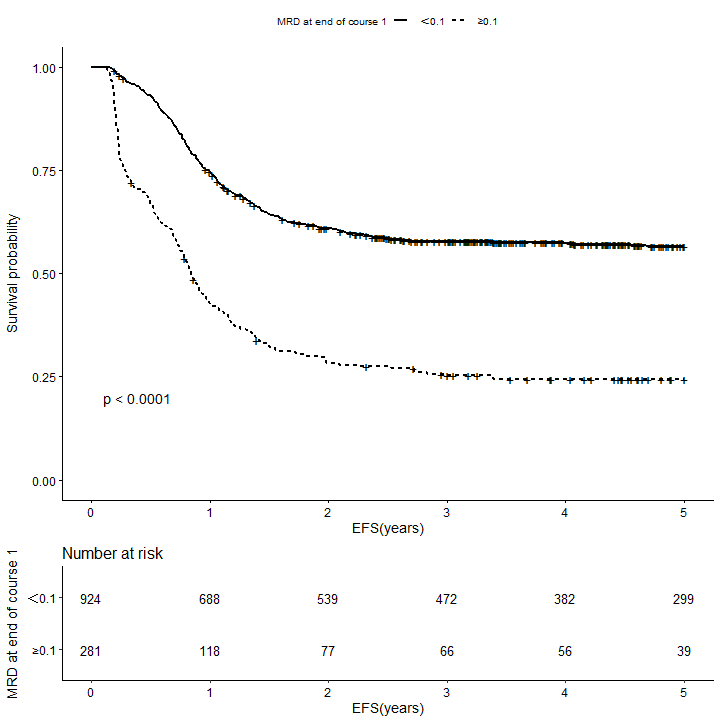

Supplement: S1 Code — (ZIP) [file pmed.1005088.s002.zip › S2 code/PROJ8_4_tbl/PROJ8_4_tbl_seg1_b.png]

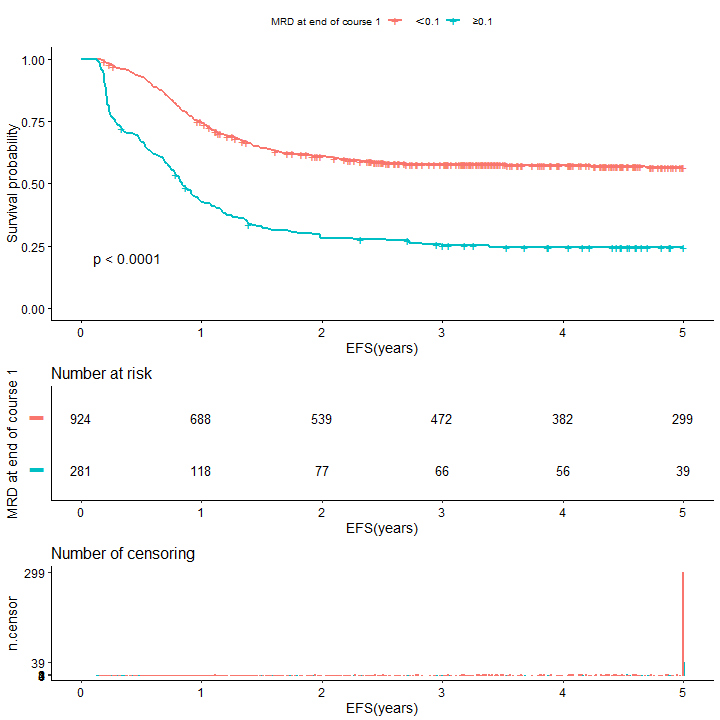

Supplement: S1 Code — (ZIP) [file pmed.1005088.s002.zip › S2 code/PROJ8_4_tbl/PROJ8_4_tbl_seg1_0.png]

## First Event

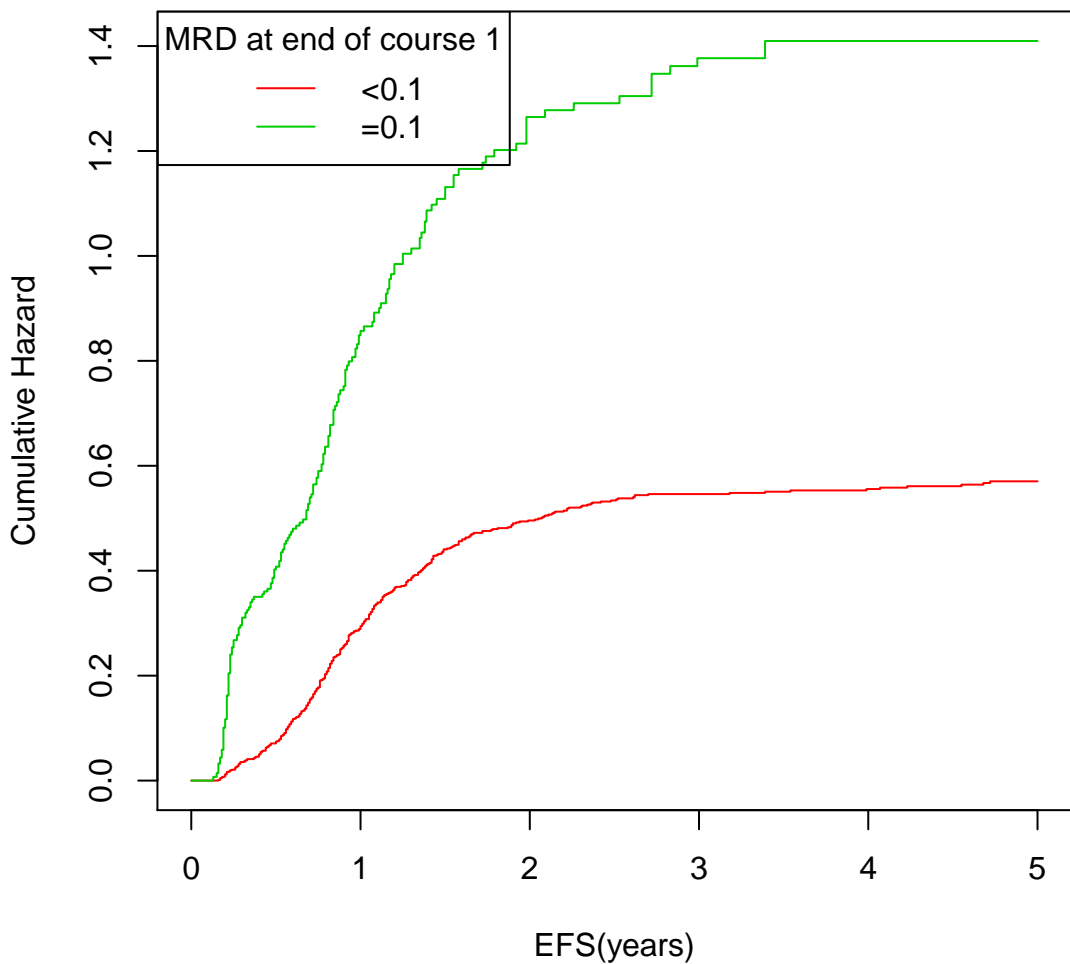

Supplement: S1 Code — (ZIP) [file pmed.1005088.s002.zip › S2 code/PROJ8_4_tbl/PROJ8_4_tbl_seg1_2.pdf]

## First Event

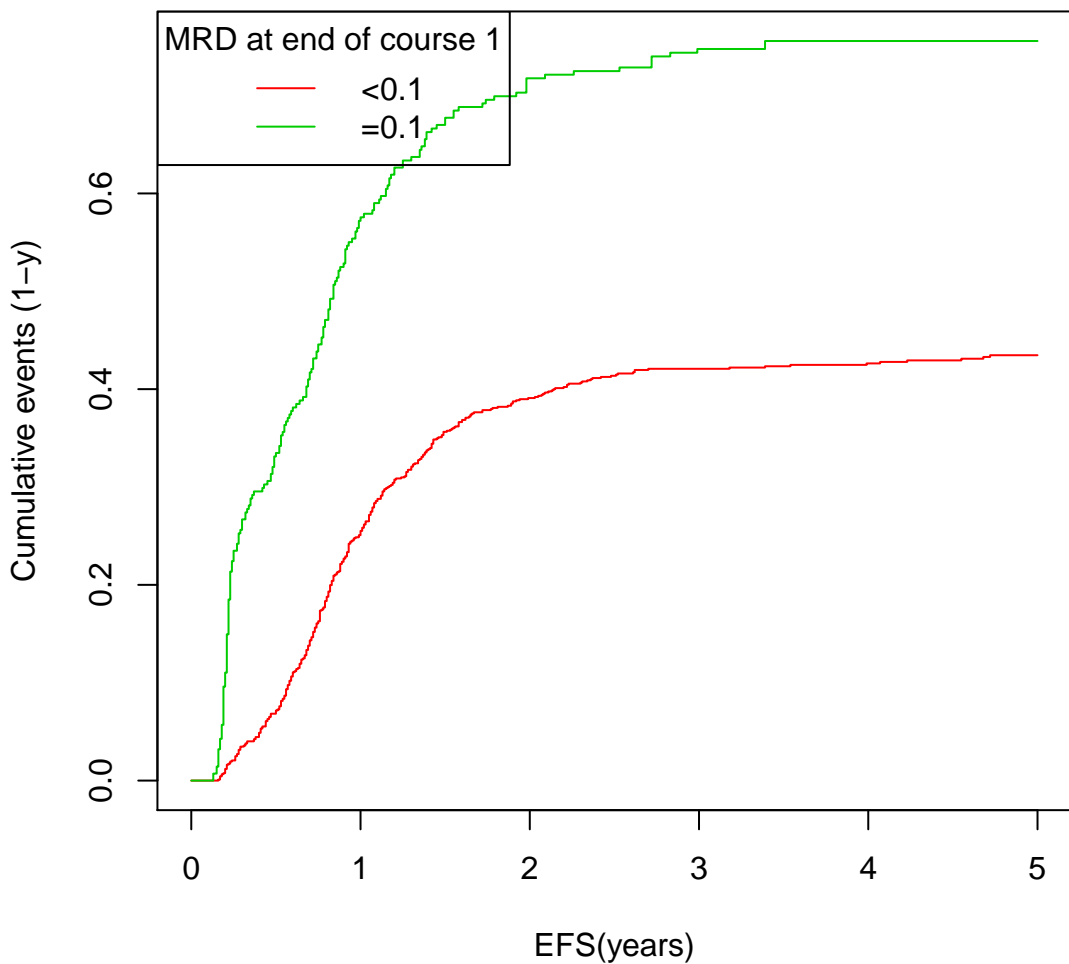

Supplement: S1 Code — (ZIP) [file pmed.1005088.s002.zip › S2 code/PROJ8_4_tbl/PROJ8_4_tbl_seg1_3.pdf]

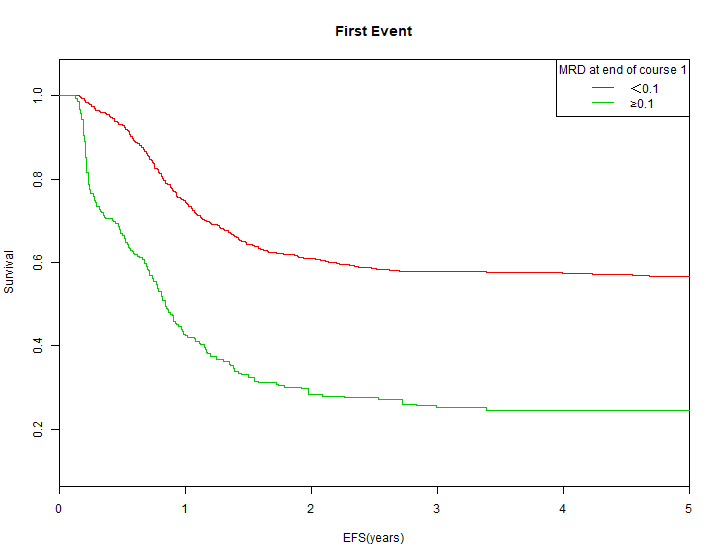

Supplement: S1 Code — (ZIP) [file pmed.1005088.s002.zip › S2 code/PROJ8_4_tbl/PROJ8_4_tbl_seg1_1.png]

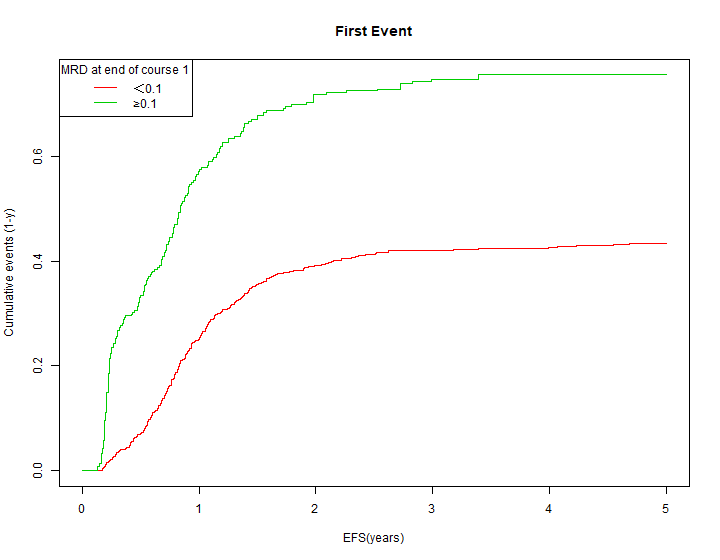

Supplement: S1 Code — (ZIP) [file pmed.1005088.s002.zip › S2 code/PROJ8_4_tbl/PROJ8_4_tbl_seg1_3.png]

## First Event

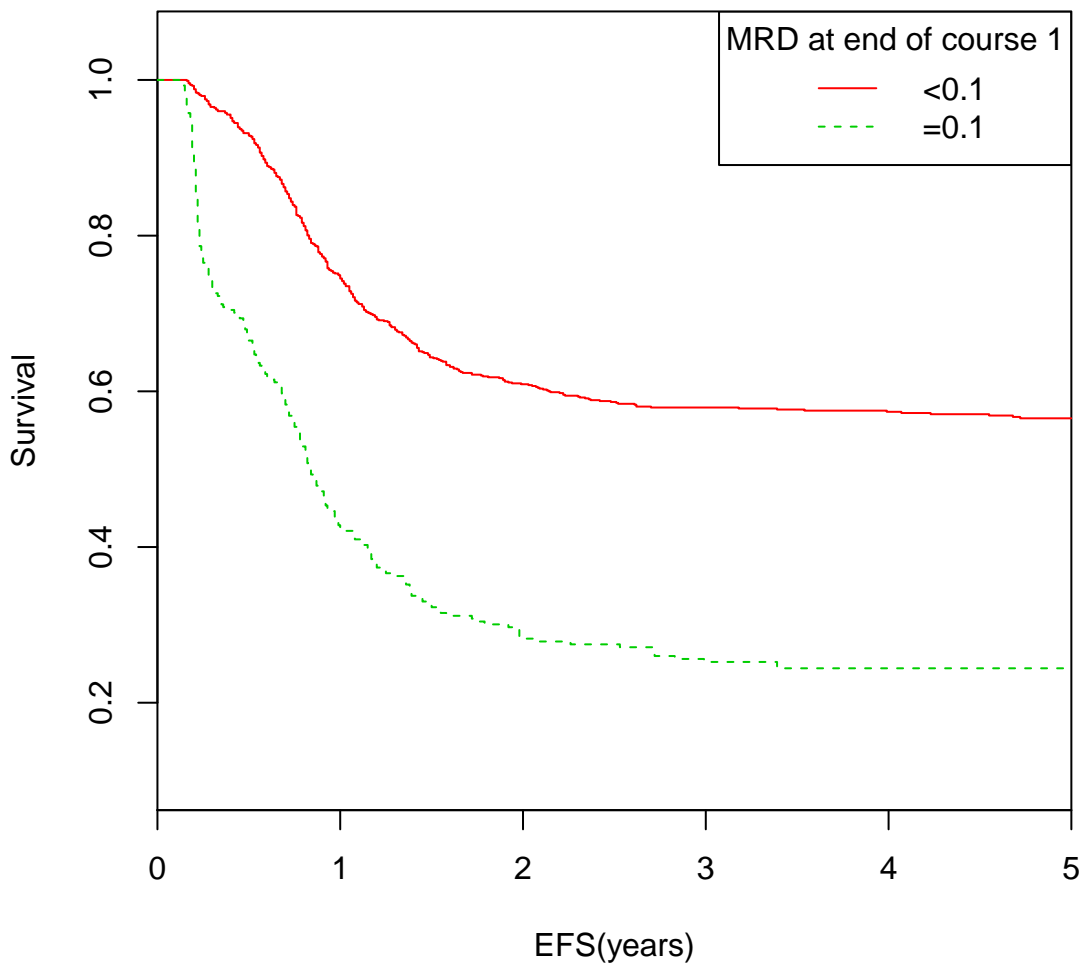

Supplement: S1 Code — (ZIP) [file pmed.1005088.s002.zip › S2 code/PROJ8_4_tbl/PROJ8_4_tbl_seg1_1.pdf]

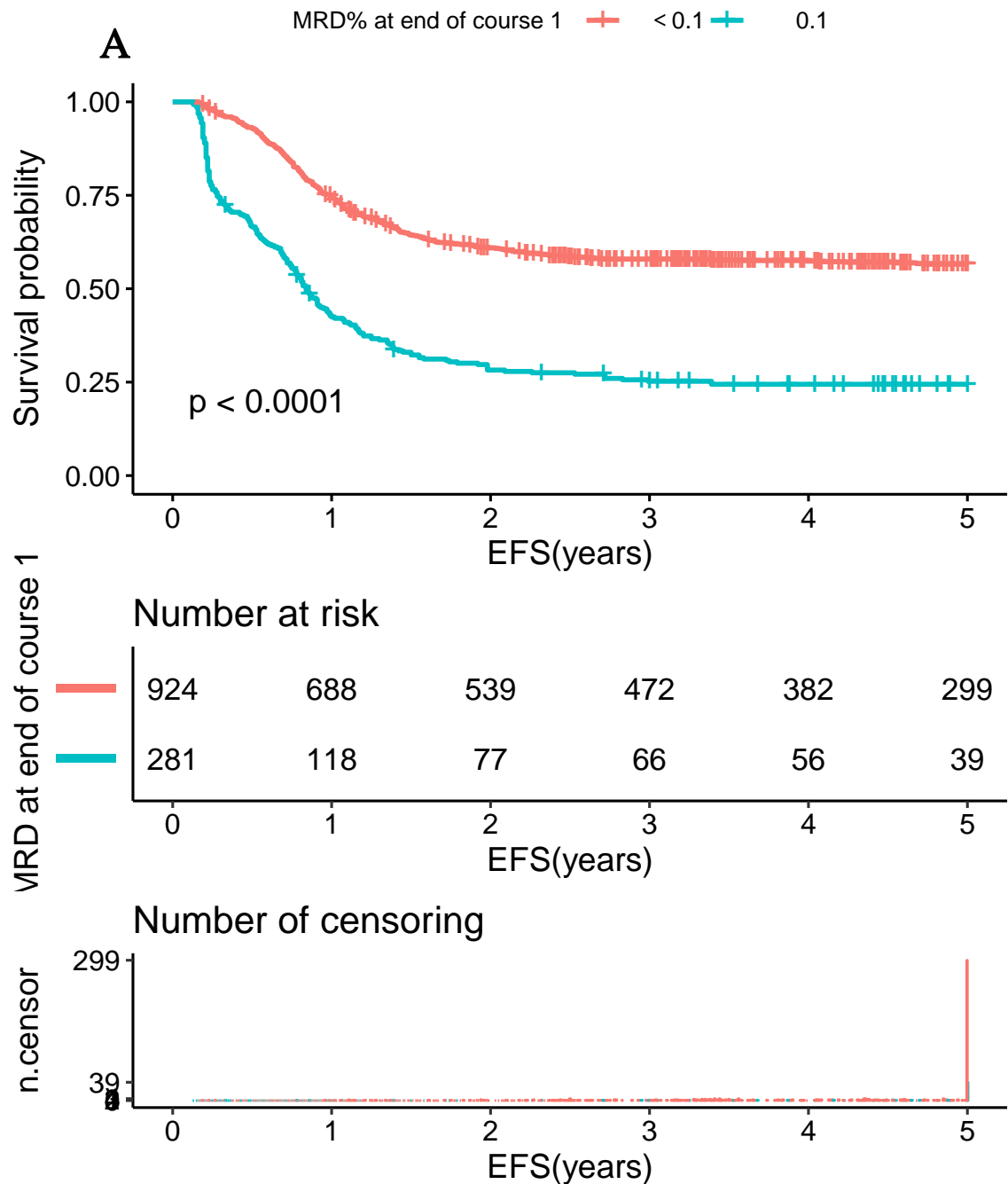

Supplement: S1 Code — (ZIP) [file pmed.1005088.s002.zip › S2 code/PROJ8_4_tbl/PROJ8_4_tbl_seg1_0.pdf]

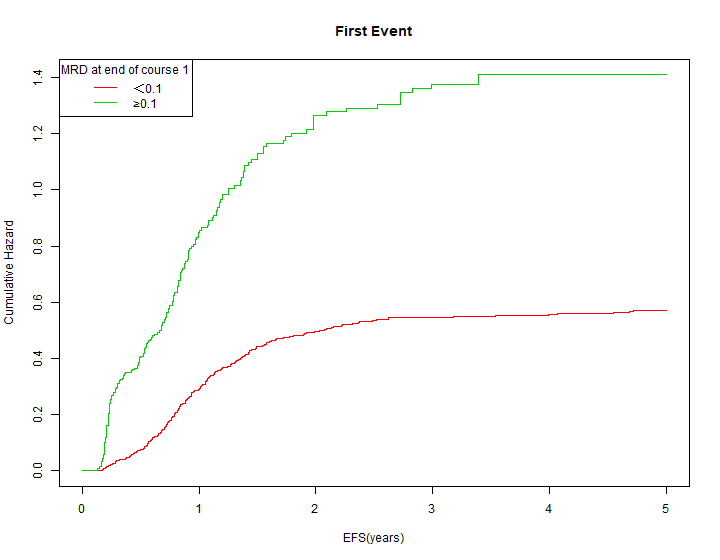

Supplement: S1 Code — (ZIP) [file pmed.1005088.s002.zip › S2 code/PROJ8_4_tbl/PROJ8_4_tbl_seg1_2.png]

Survival probability

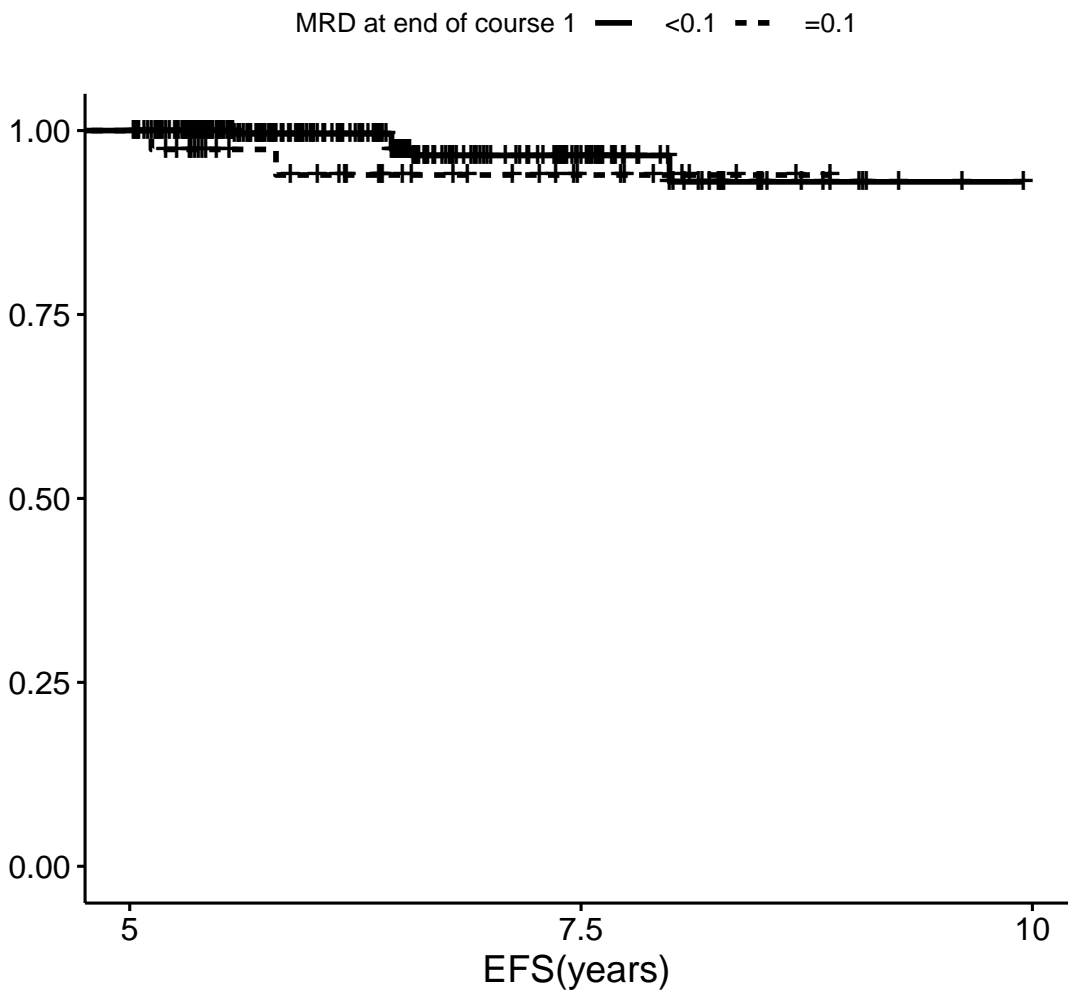

EFS(years)

Supplement: S1 Code — (ZIP) [file pmed.1005088.s002.zip › S2 code/PROJ8_4_tbl/PROJ8_4_tbl_seg2_b.pdf]

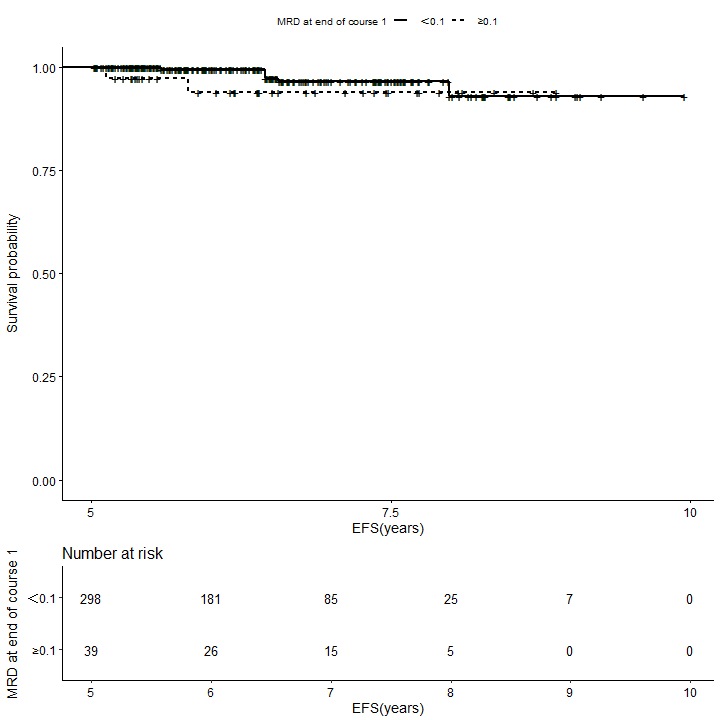

Supplement: S1 Code — (ZIP) [file pmed.1005088.s002.zip › S2 code/PROJ8_4_tbl/PROJ8_4_tbl_seg2_b.png]

## First Event

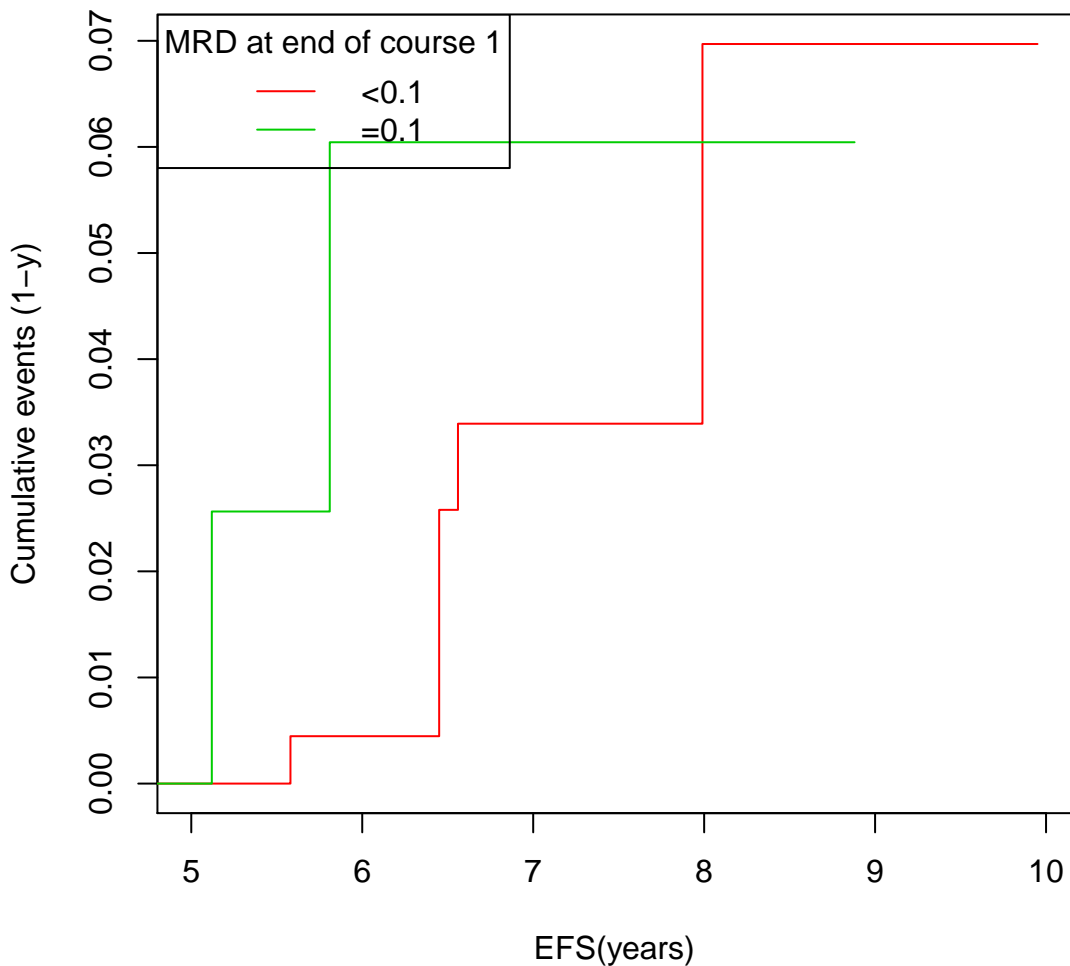

Supplement: S1 Code — (ZIP) [file pmed.1005088.s002.zip › S2 code/PROJ8_4_tbl/PROJ8_4_tbl_seg2_3.pdf]

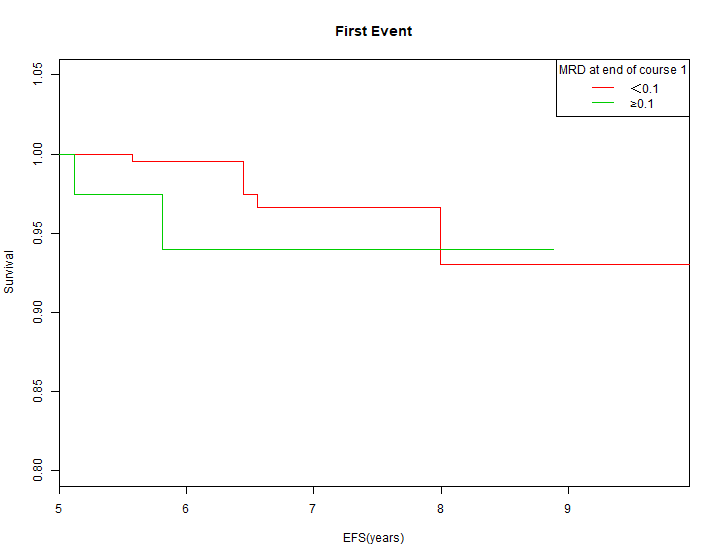

Supplement: S1 Code — (ZIP) [file pmed.1005088.s002.zip › S2 code/PROJ8_4_tbl/PROJ8_4_tbl_seg2_1.png]

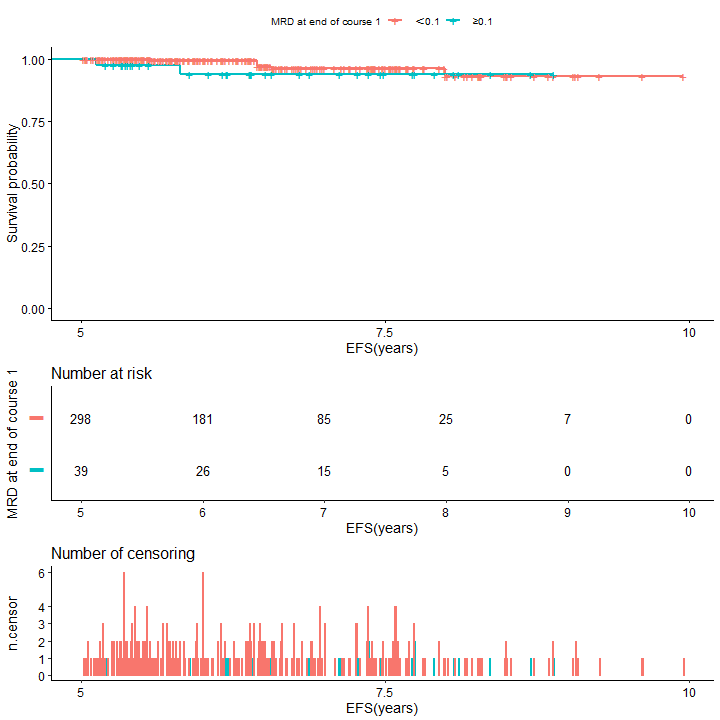

Supplement: S1 Code — (ZIP) [file pmed.1005088.s002.zip › S2 code/PROJ8_4_tbl/PROJ8_4_tbl_seg2_0.png]

## First Event

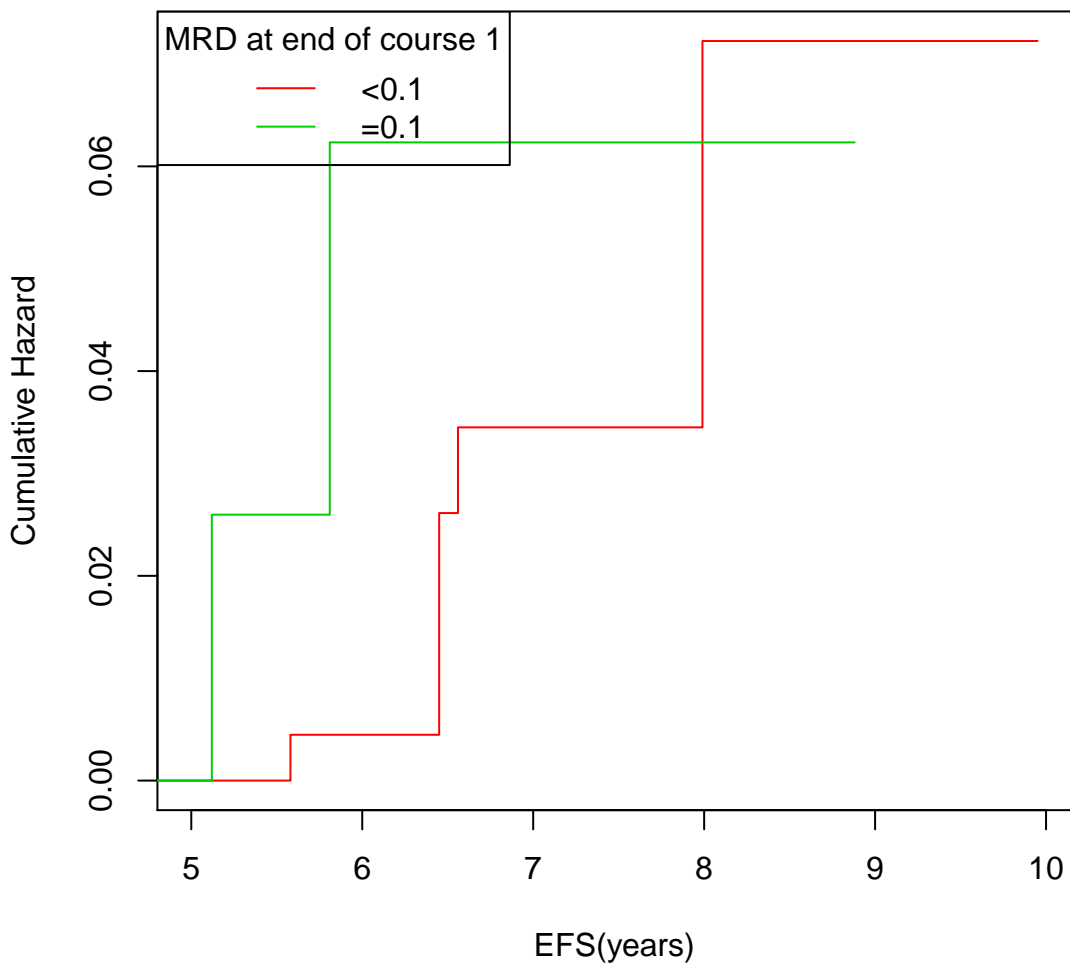

Supplement: S1 Code — (ZIP) [file pmed.1005088.s002.zip › S2 code/PROJ8_4_tbl/PROJ8_4_tbl_seg2_2.pdf]

MRD at end of course 1    + <0.1    + =0.1

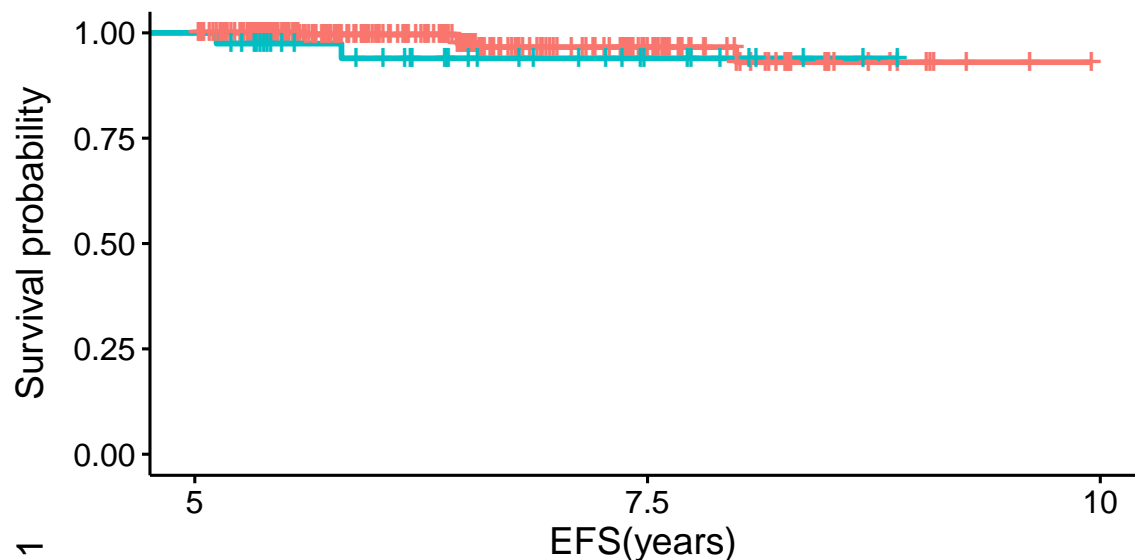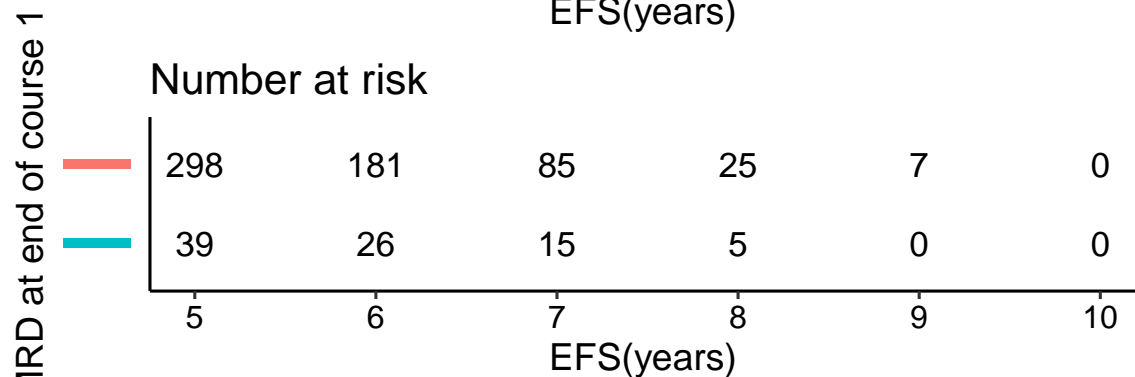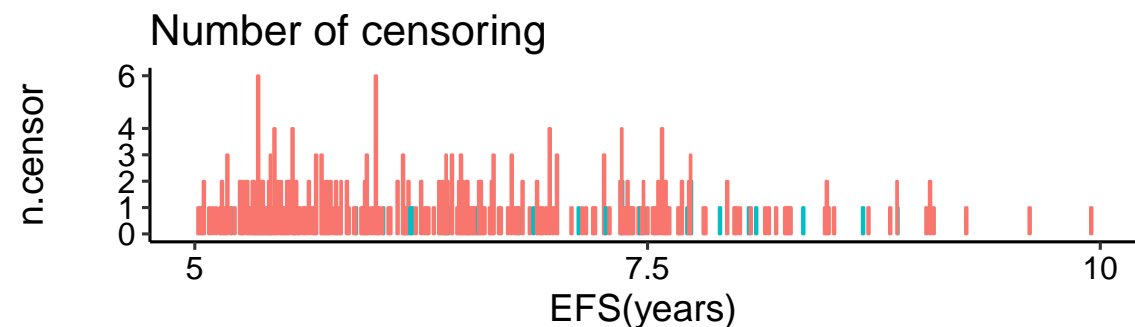

Supplement: S1 Code — (ZIP) [file pmed.1005088.s002.zip › S2 code/PROJ8_4_tbl/PROJ8_4_tbl_seg2_0.pdf]

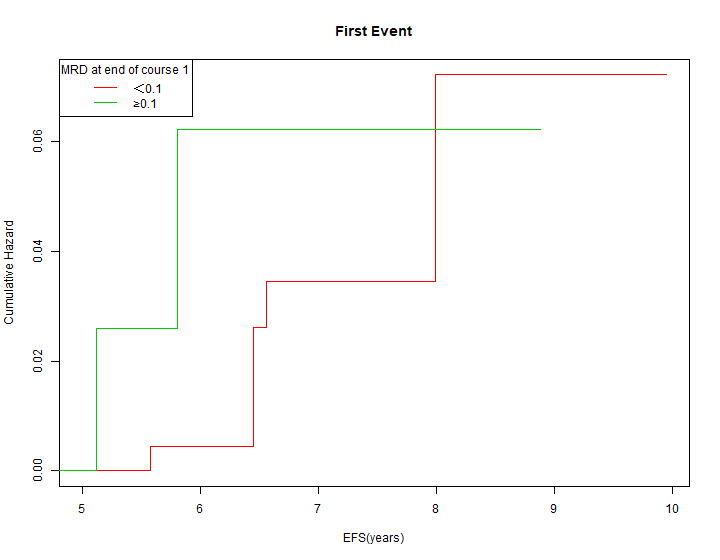

Supplement: S1 Code — (ZIP) [file pmed.1005088.s002.zip › S2 code/PROJ8_4_tbl/PROJ8_4_tbl_seg2_2.png]

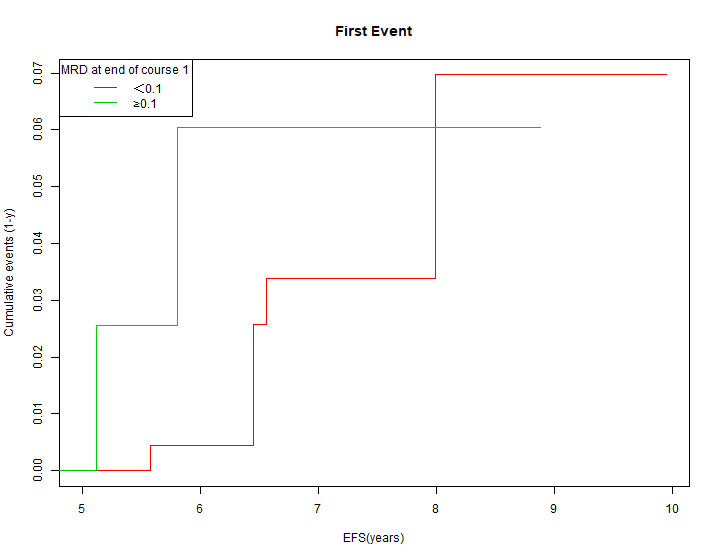

Supplement: S1 Code — (ZIP) [file pmed.1005088.s002.zip › S2 code/PROJ8_4_tbl/PROJ8_4_tbl_seg2_3.png]

## First Event

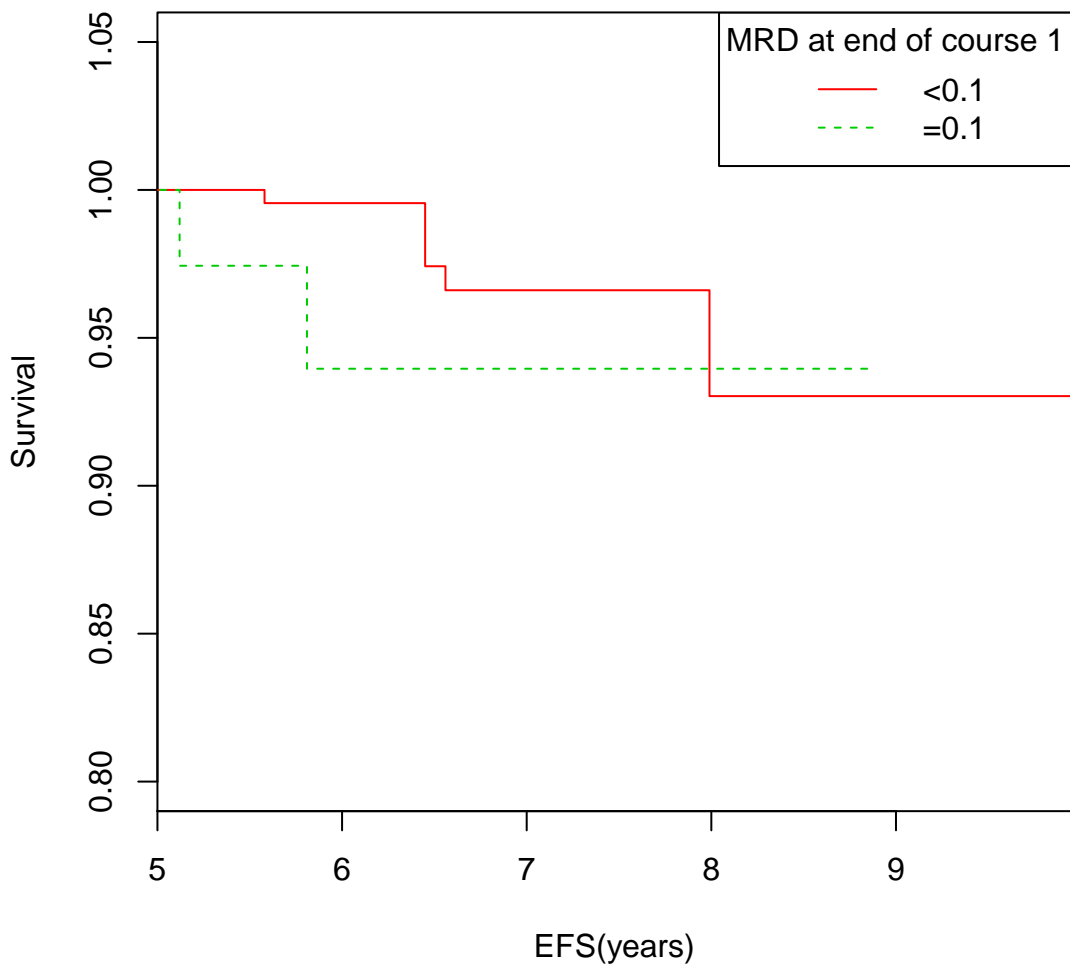

Supplement: S1 Code — (ZIP) [file pmed.1005088.s002.zip › S2 code/PROJ8_4_tbl/PROJ8_4_tbl_seg2_1.pdf]

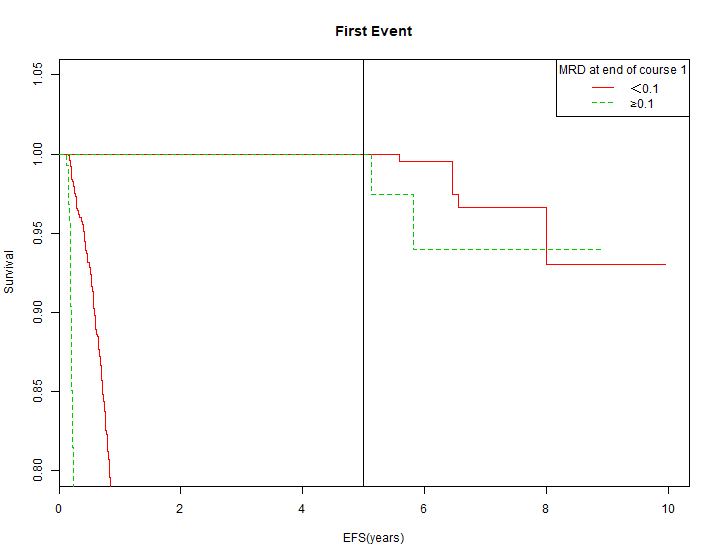

Supplement: S1 Code — (ZIP) [file pmed.1005088.s002.zip › S2 code/PROJ8_4_tbl/PROJ8_4_tbl.png]

## First Event

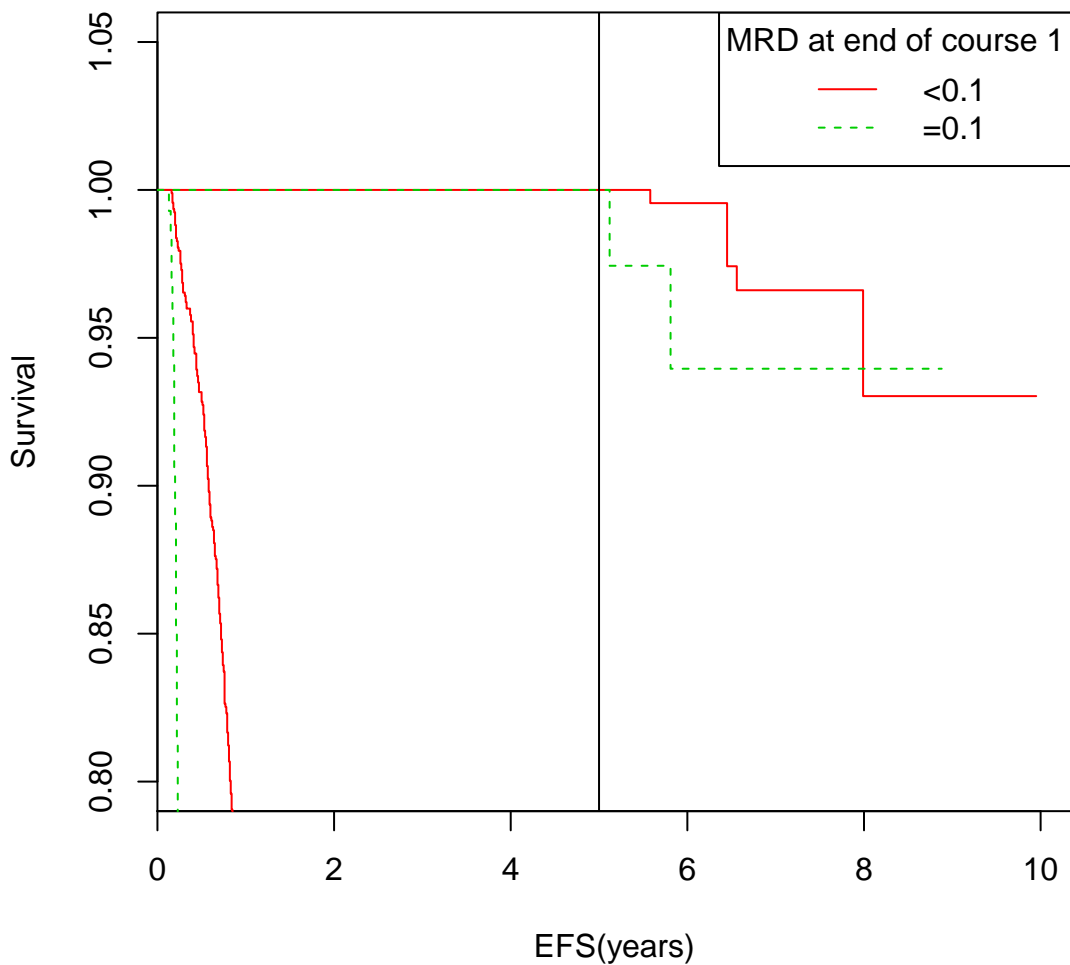

Supplement: S1 Code — (ZIP) [file pmed.1005088.s002.zip › S2 code/PROJ8_4_tbl/PROJ8_4_tbl.pdf]

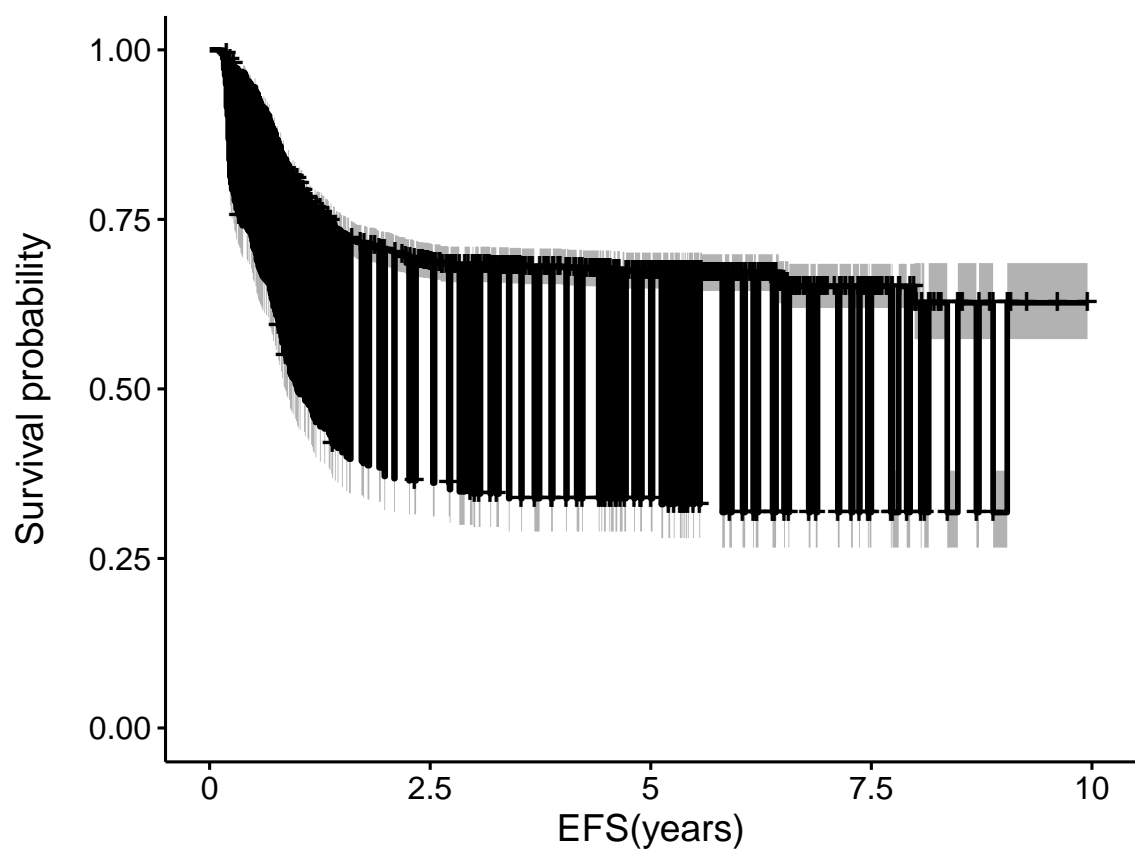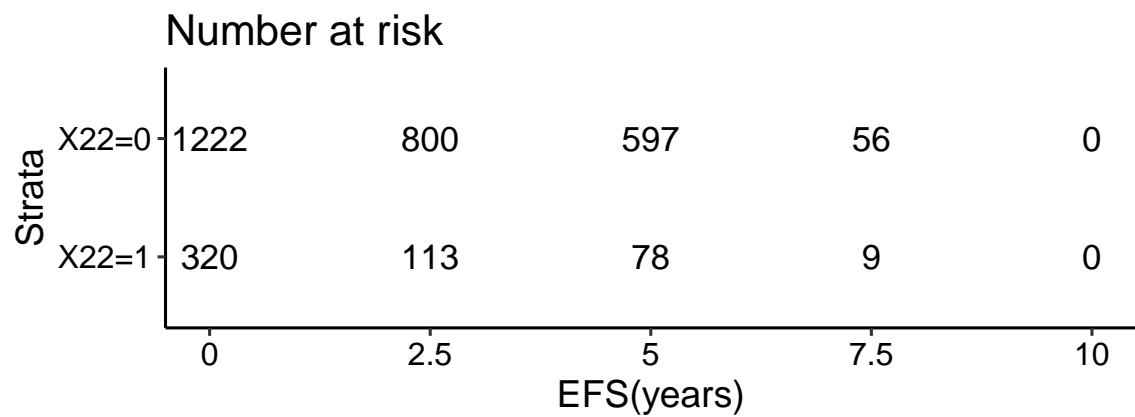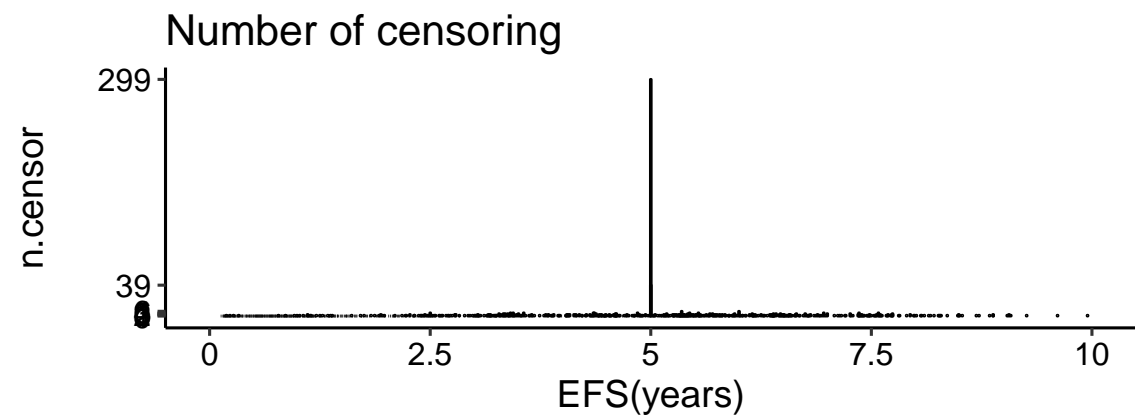

Supplement: S1 Code — (ZIP) [file pmed.1005088.s002.zip › S2 code/PROJ8_4_tbl/PROJ8_4_tbl_0.pdf]

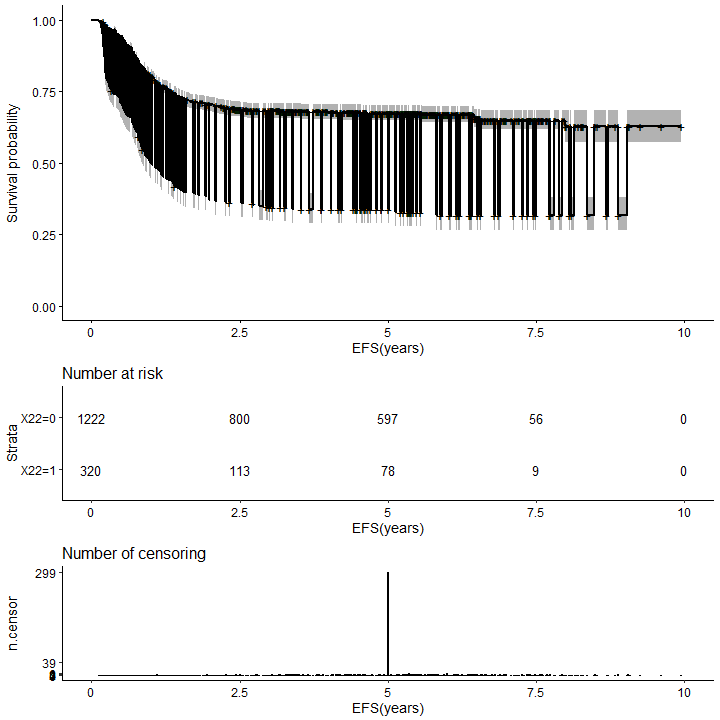

Supplement: S1 Code — (ZIP) [file pmed.1005088.s002.zip › S2 code/PROJ8_4_tbl/PROJ8_4_tbl_0.png]

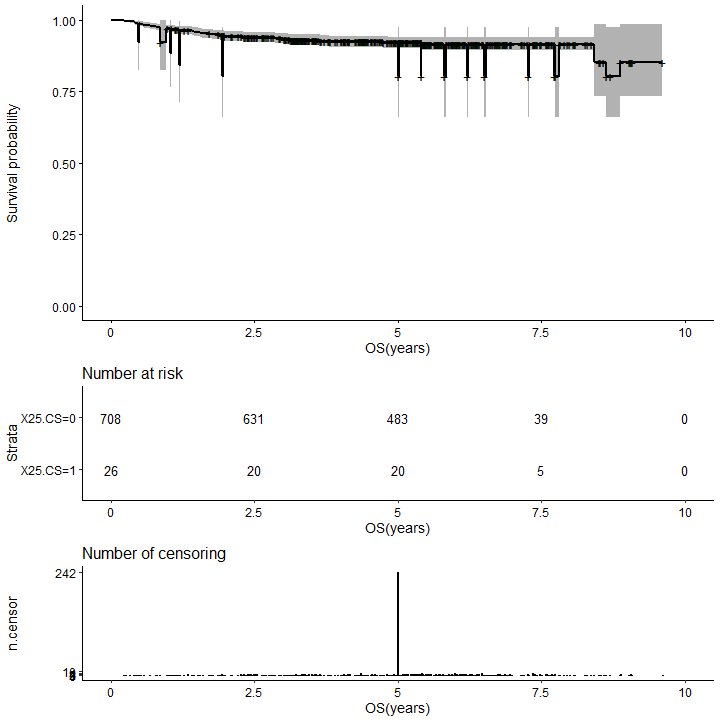

Supplement: S1 Code — (ZIP) [file pmed.1005088.s002.zip › S2 code/PROJ8_16_tbl2/PROJ8_16_tbl2_0.png]

MRD % at end of course 2 .....

— <0.05    - - - ≥0.05

Survival probability

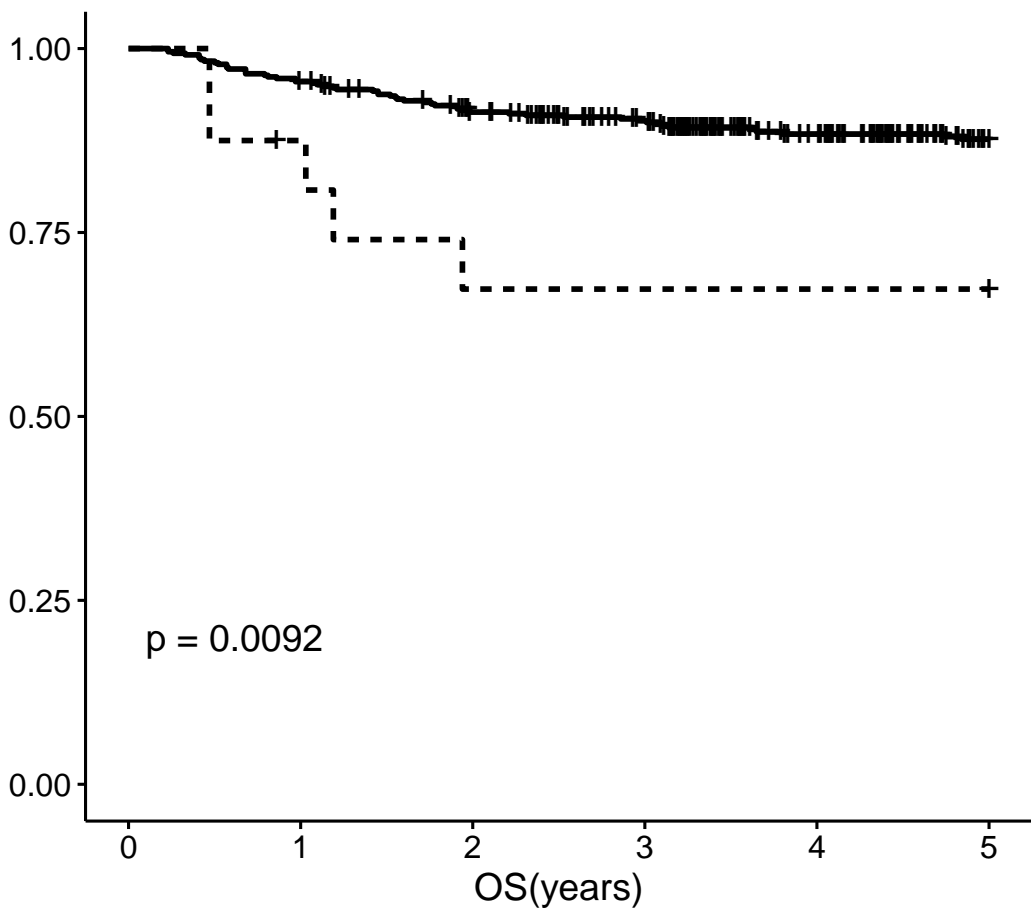

Number at risk

|       |           |     |     |     |     |     |
|-------|-----------|-----|-----|-----|-----|-----|
| <0.05 | 467       | 445 | 411 | 372 | 306 | 242 |
| ≥0.05 | 16        | 13  | 10  | 10  | 10  | 10  |
|       | 0         | 1   | 2   | 3   | 4   | 5   |
|       | OS(years) |     |     |     |     |     |

Supplement: S1 Code — (ZIP) [file pmed.1005088.s002.zip › S2 code/PROJ8_16_tbl2/PROJ8_16_tbl2_seg1_b.pdf]

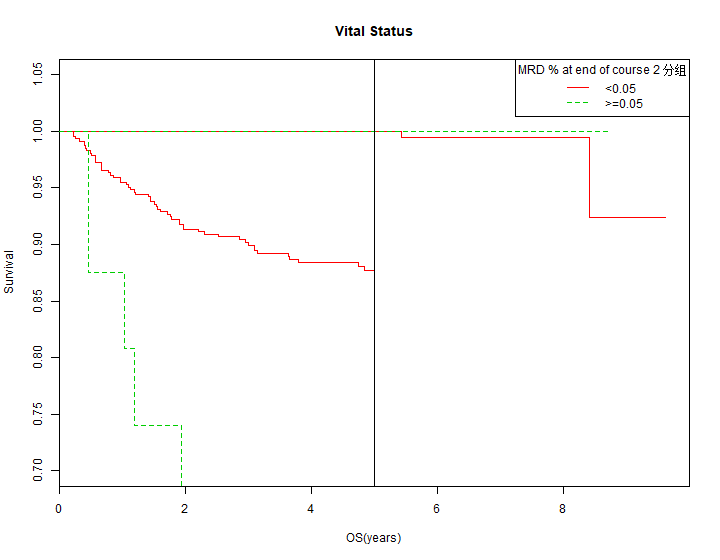

Supplement: S1 Code — (ZIP) [file pmed.1005088.s002.zip › S2 code/PROJ8_16_tbl2/PROJ8_16_tbl2.png]

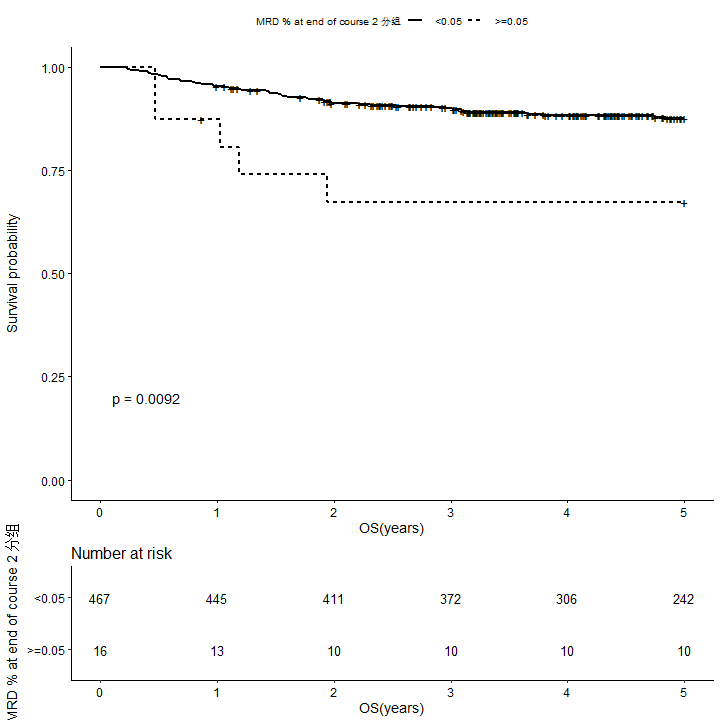

Supplement: S1 Code — (ZIP) [file pmed.1005088.s002.zip › S2 code/PROJ8_16_tbl2/PROJ8_16_tbl2_seg1_b.png]

# Vital Status

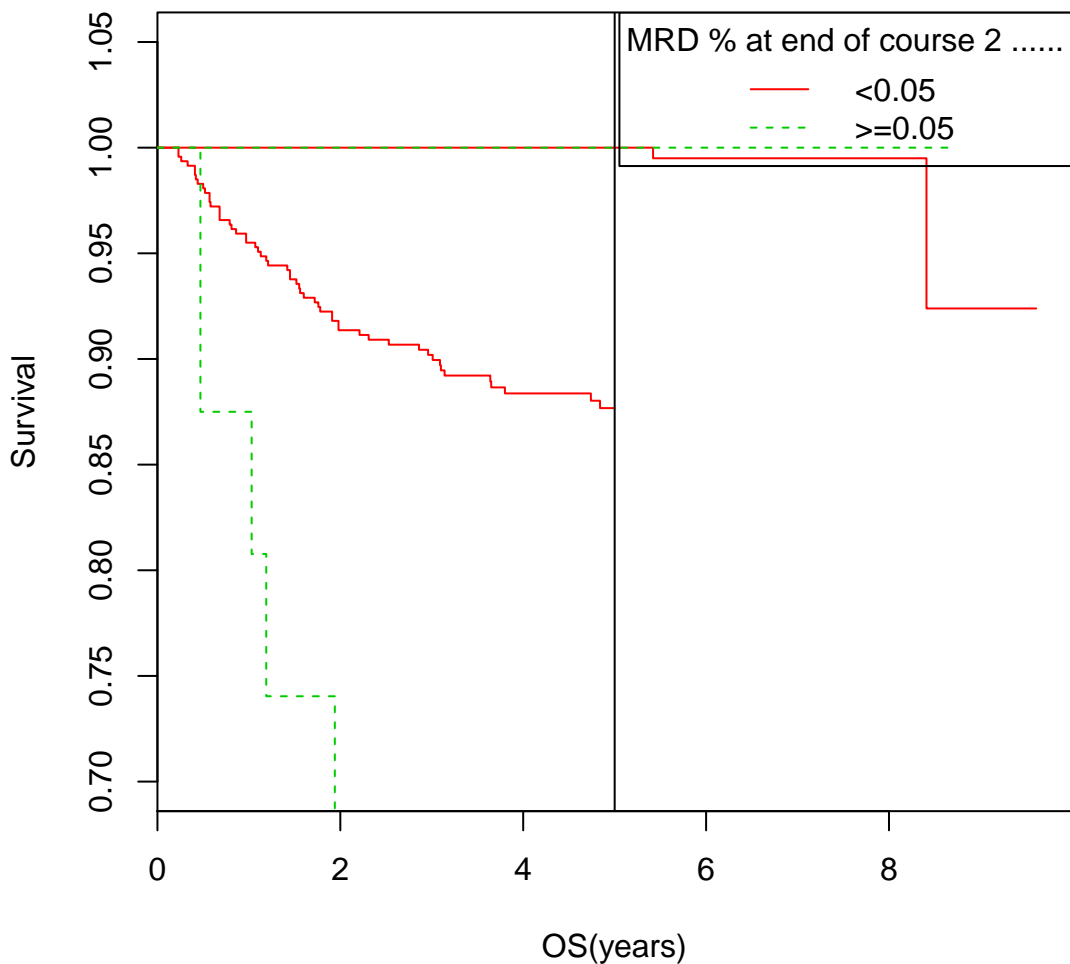

Supplement: S1 Code — (ZIP) [file pmed.1005088.s002.zip › S2 code/PROJ8_16_tbl2/PROJ8_16_tbl2.pdf]

Survival probability

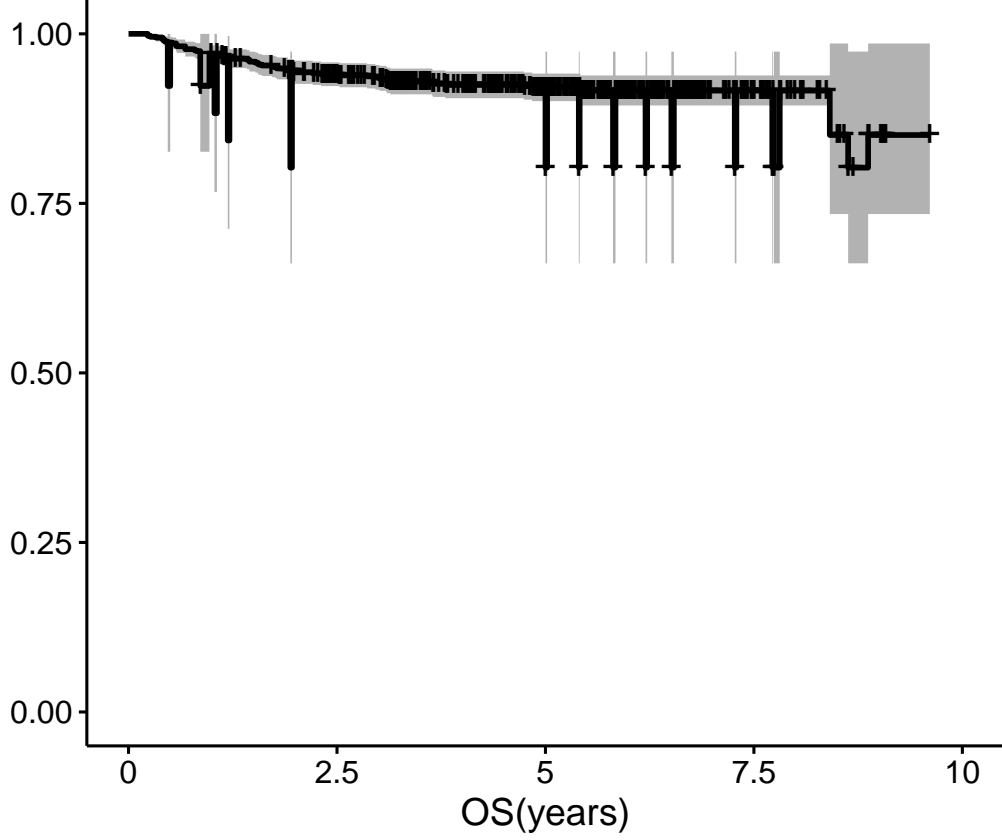

Number at risk

Strata

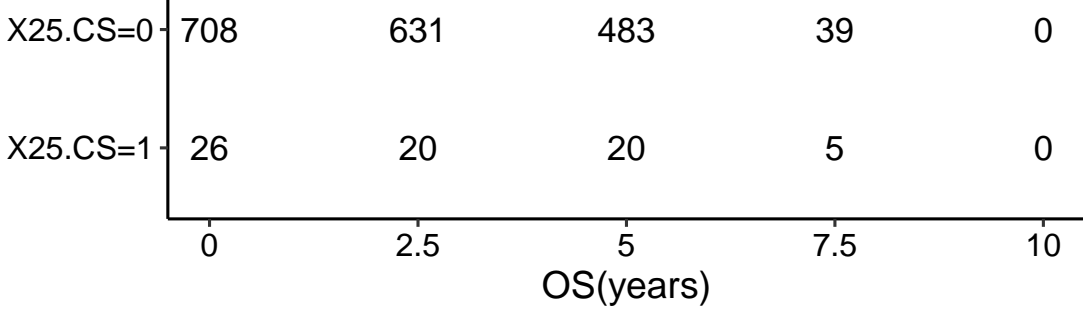

Number of censoring

n.censor

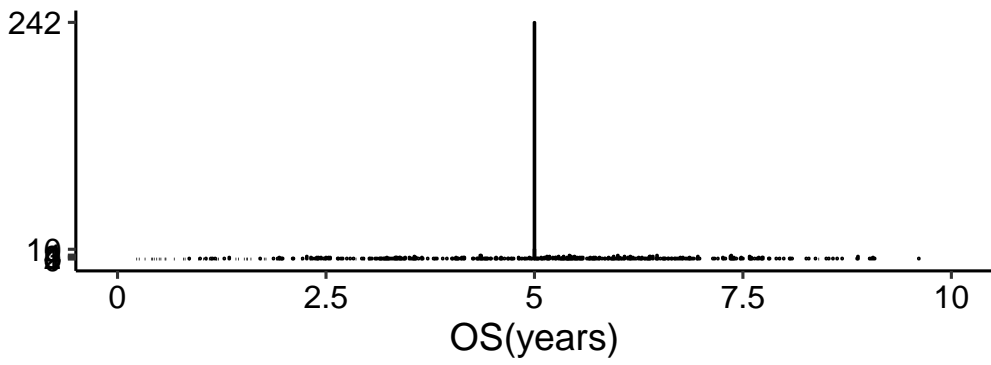

Supplement: S1 Code — (ZIP) [file pmed.1005088.s002.zip › S2 code/PROJ8_16_tbl2/PROJ8_16_tbl2_0.pdf]

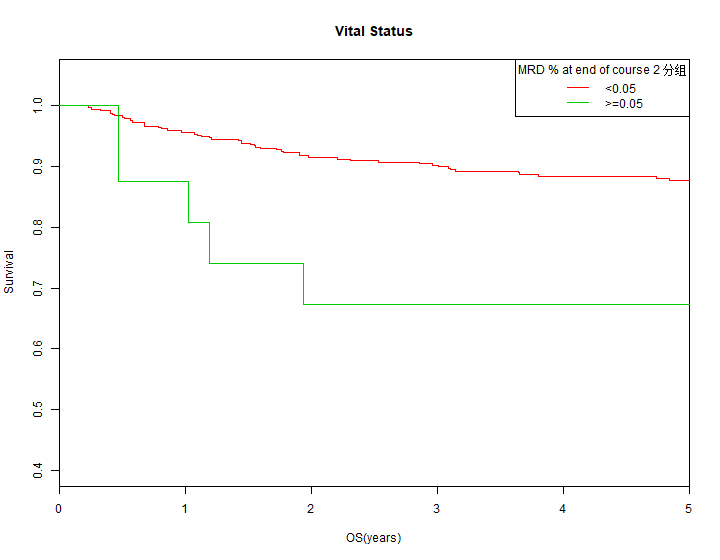

Supplement: S1 Code — (ZIP) [file pmed.1005088.s002.zip › S2 code/PROJ8_16_tbl2/PROJ8_16_tbl2_seg1_1.png]

## Vital Status

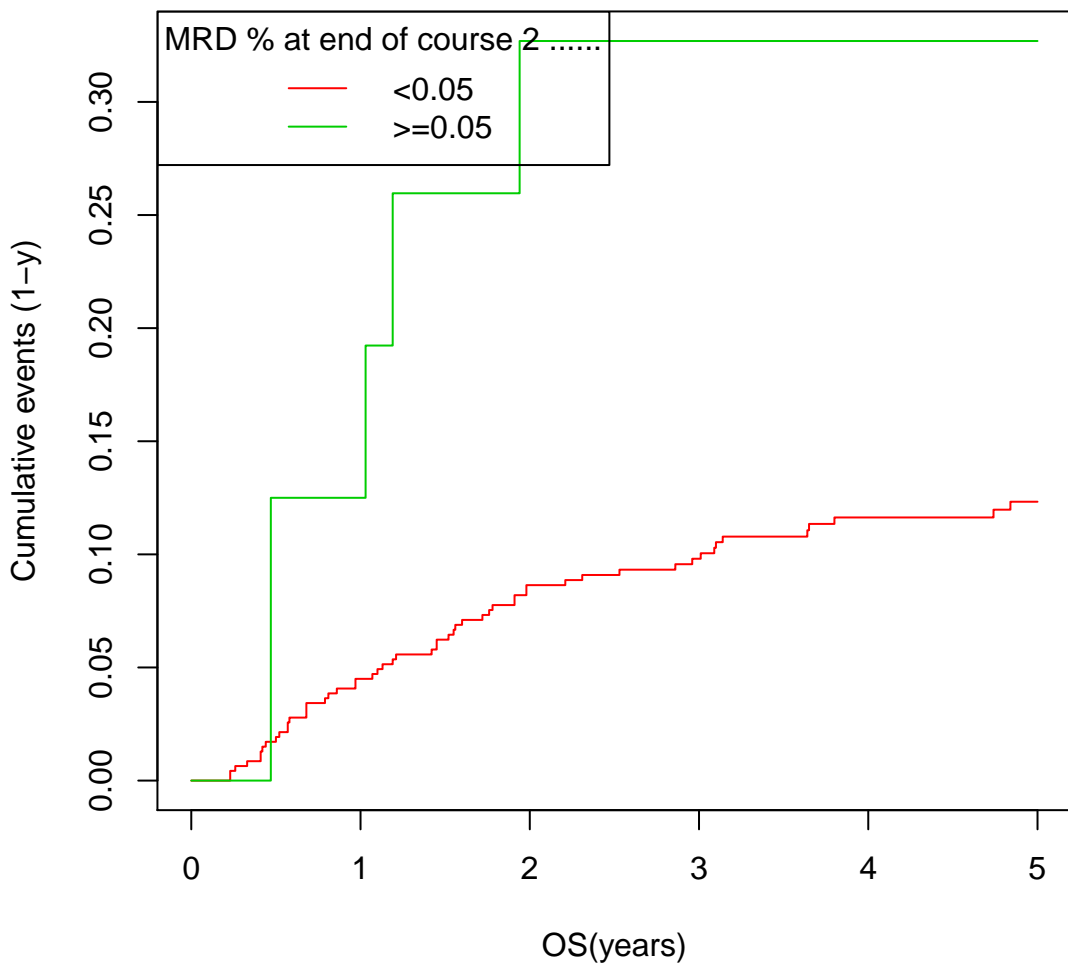

Supplement: S1 Code — (ZIP) [file pmed.1005088.s002.zip › S2 code/PROJ8_16_tbl2/PROJ8_16_tbl2_seg1_3.pdf]

## Vital Status

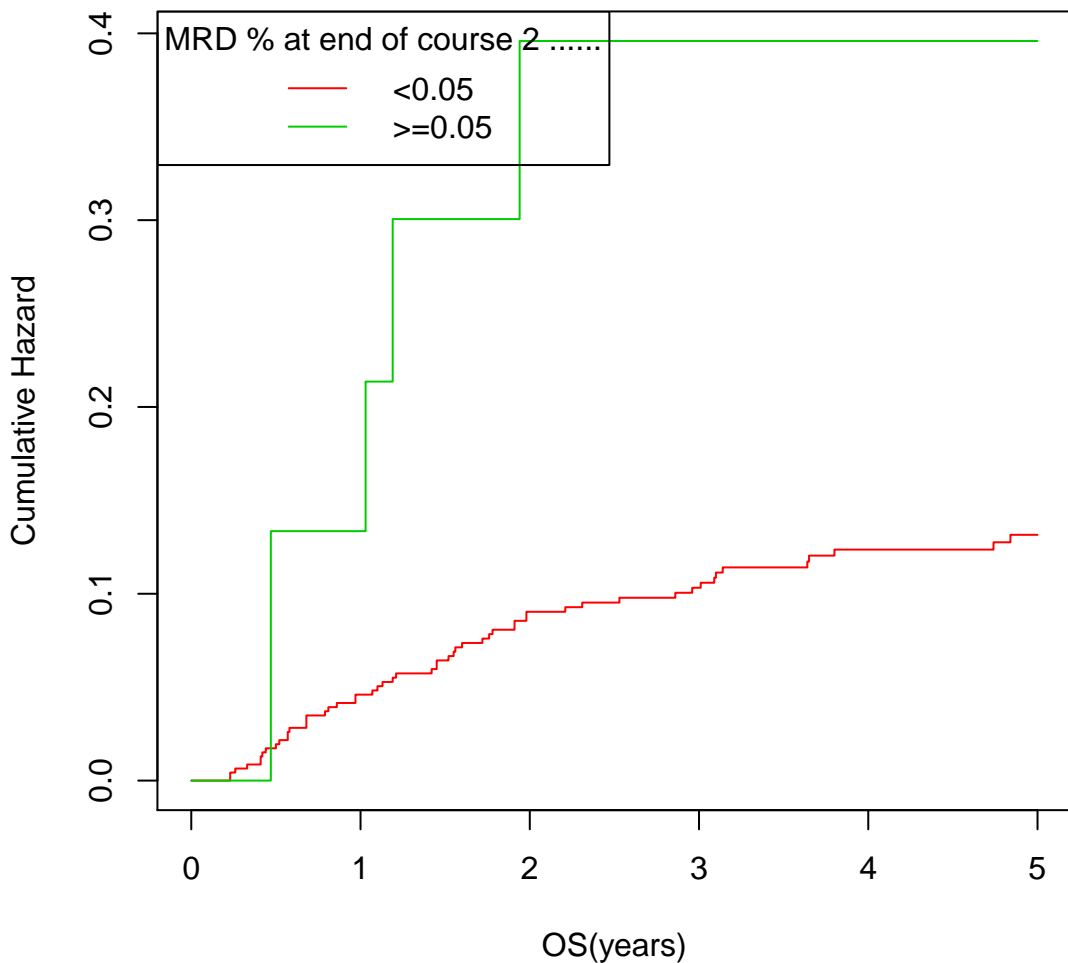

Supplement: S1 Code — (ZIP) [file pmed.1005088.s002.zip › S2 code/PROJ8_16_tbl2/PROJ8_16_tbl2_seg1_2.pdf]

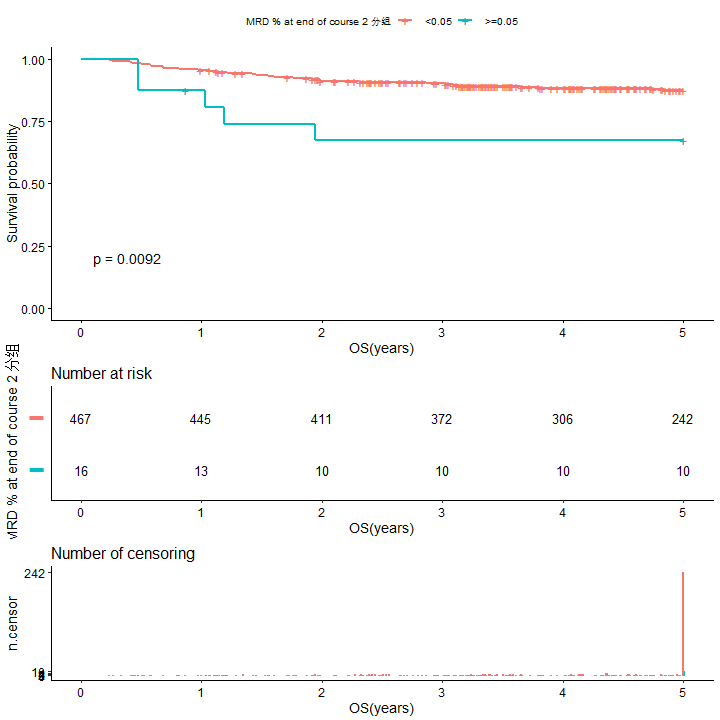

Supplement: S1 Code — (ZIP) [file pmed.1005088.s002.zip › S2 code/PROJ8_16_tbl2/PROJ8_16_tbl2_seg1_0.png]

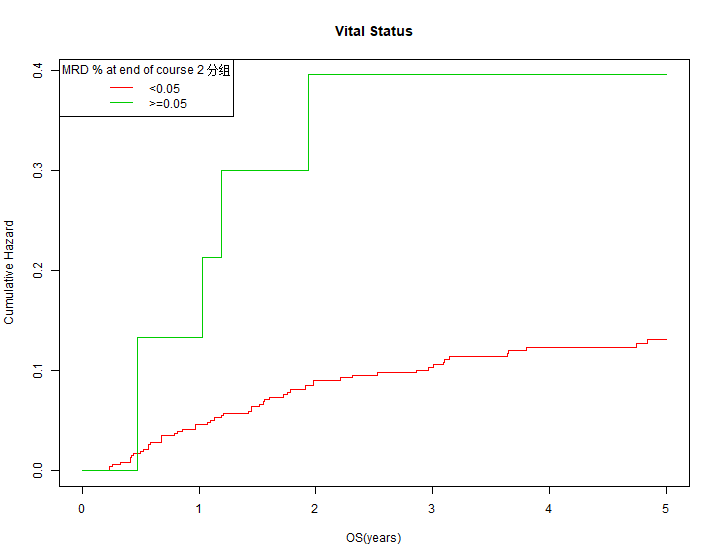

Supplement: S1 Code — (ZIP) [file pmed.1005088.s002.zip › S2 code/PROJ8_16_tbl2/PROJ8_16_tbl2_seg1_2.png]
