# Supplementary figures and images for: Optimal minimal residual disease threshold in pediatric acute myeloid leukemia: A retrospective cohort study based on the TARGET database (part 1 of 2)
Source: PLoS Med. 2026 May 8;23(5):e1005088. doi: 10.1371/journal.pmed.1005088 (PMC13155632; doi:10.1371/journal.pmed.1005088)

**Full**  
**Time = 0.3 , AUC = 0.86**

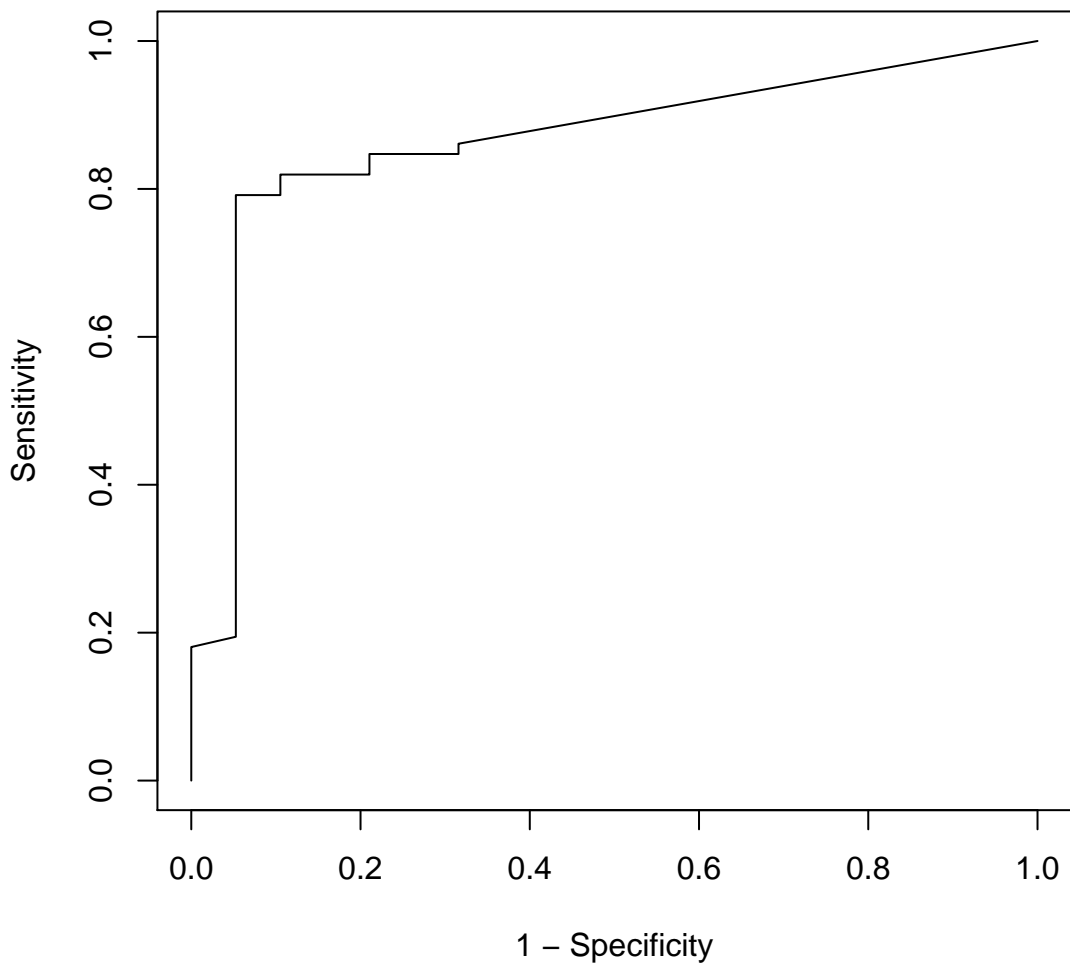

Supplement: S1 Code — (ZIP) [file pmed.1005088.s002.zip › S2 code/PROJ8_3_tbl/PROJ8_3_tbl_full_roc.pdf]

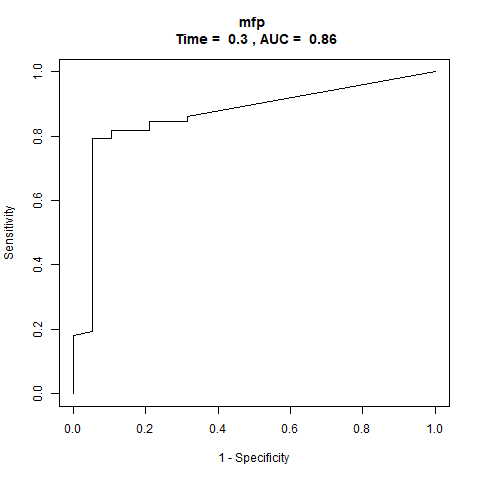

Supplement: S1 Code — (ZIP) [file pmed.1005088.s002.zip › S2 code/PROJ8_3_tbl/PROJ8_3_tbl_mfp_roc.png]

mfp  
Time = 0.3 , AUC = 0.86

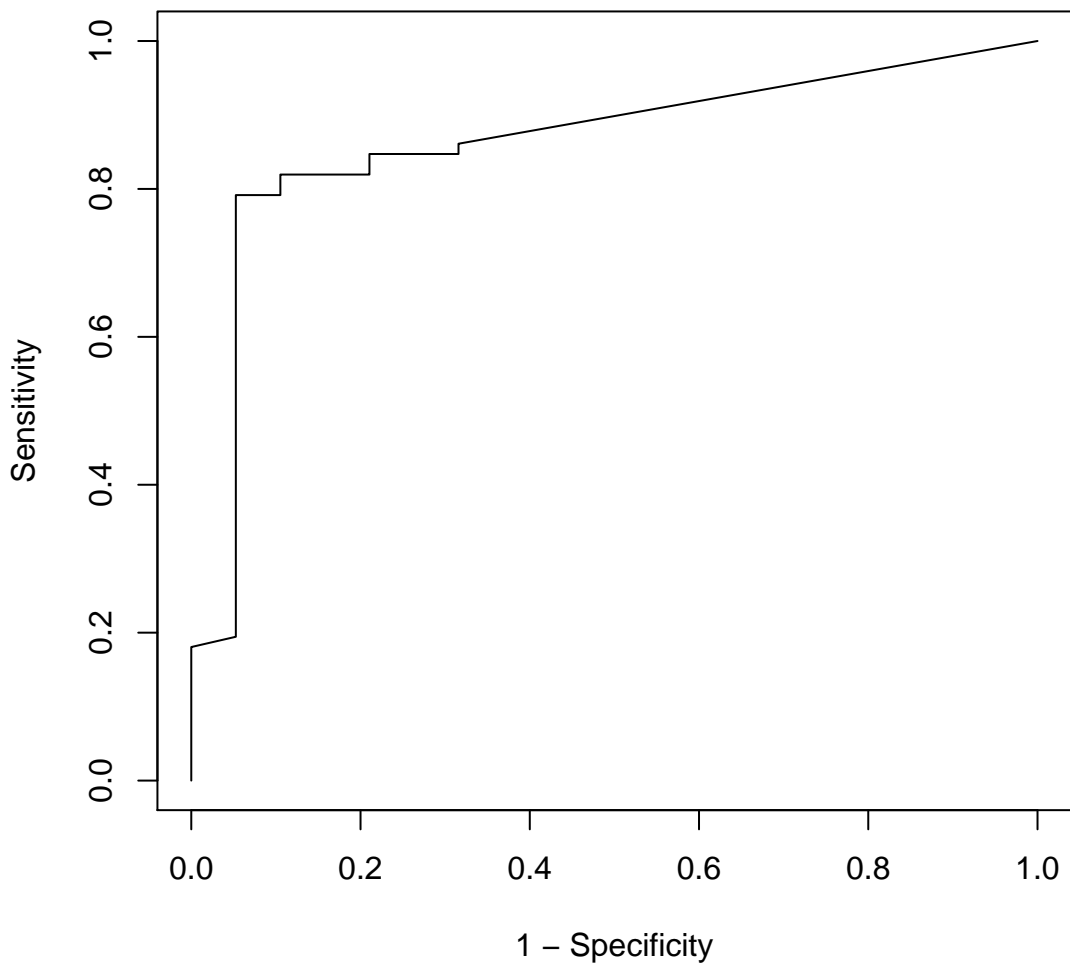

Supplement: S1 Code — (ZIP) [file pmed.1005088.s002.zip › S2 code/PROJ8_3_tbl/PROJ8_3_tbl_mfp_roc.pdf]

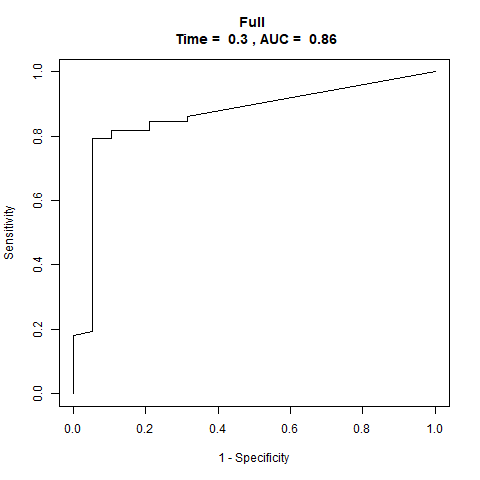

Supplement: S1 Code — (ZIP) [file pmed.1005088.s002.zip › S2 code/PROJ8_3_tbl/PROJ8_3_tbl_full_roc.png]

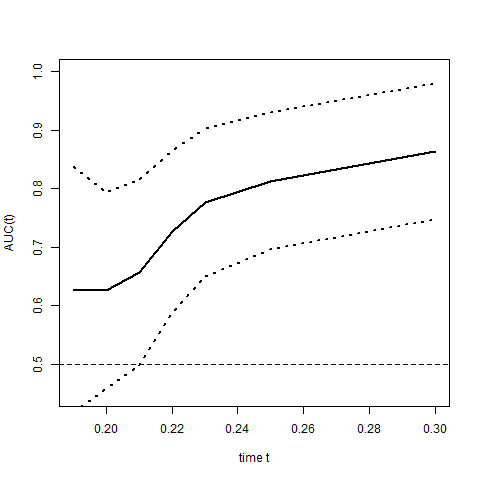

Supplement: S1 Code — (ZIP) [file pmed.1005088.s002.zip › S2 code/PROJ8_3_tbl/PROJ8_3_tbl_mfp_auc.png]

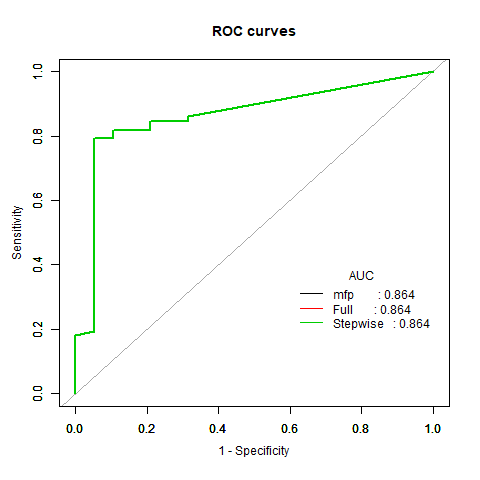

Supplement: S1 Code — (ZIP) [file pmed.1005088.s002.zip › S2 code/PROJ8_3_tbl/PROJ8_3_tbl_rocs.png]

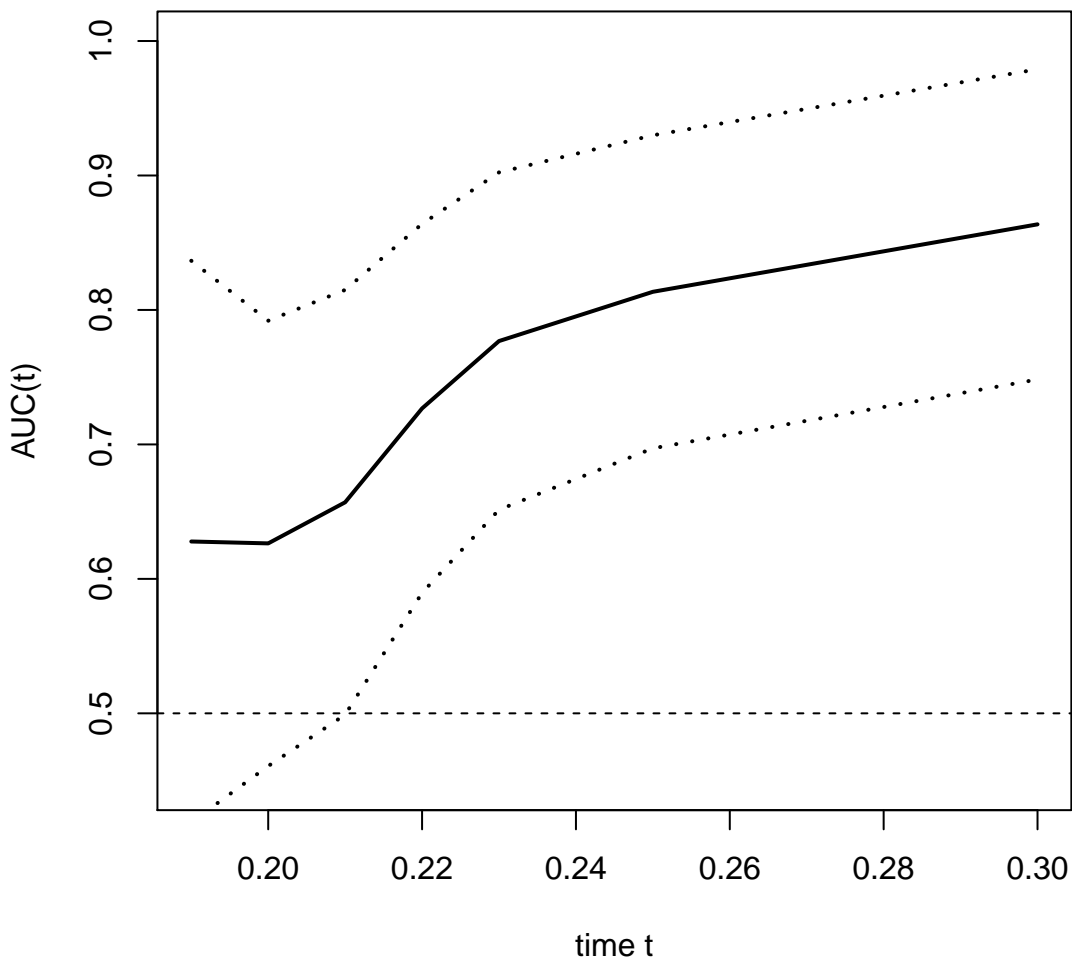

Supplement: S1 Code — (ZIP) [file pmed.1005088.s002.zip › S2 code/PROJ8_3_tbl/PROJ8_3_tbl_full_auc.pdf]

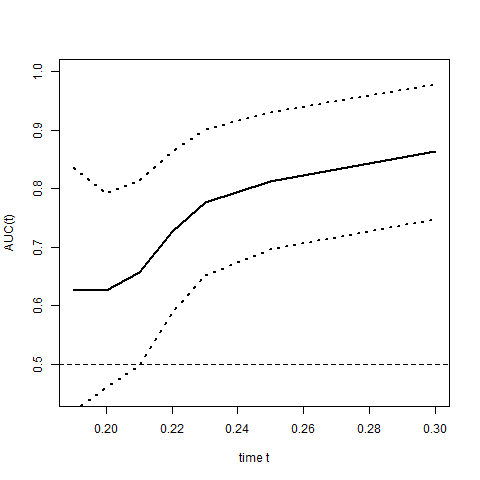

Supplement: S1 Code — (ZIP) [file pmed.1005088.s002.zip › S2 code/PROJ8_3_tbl/PROJ8_3_tbl_full_auc.png]

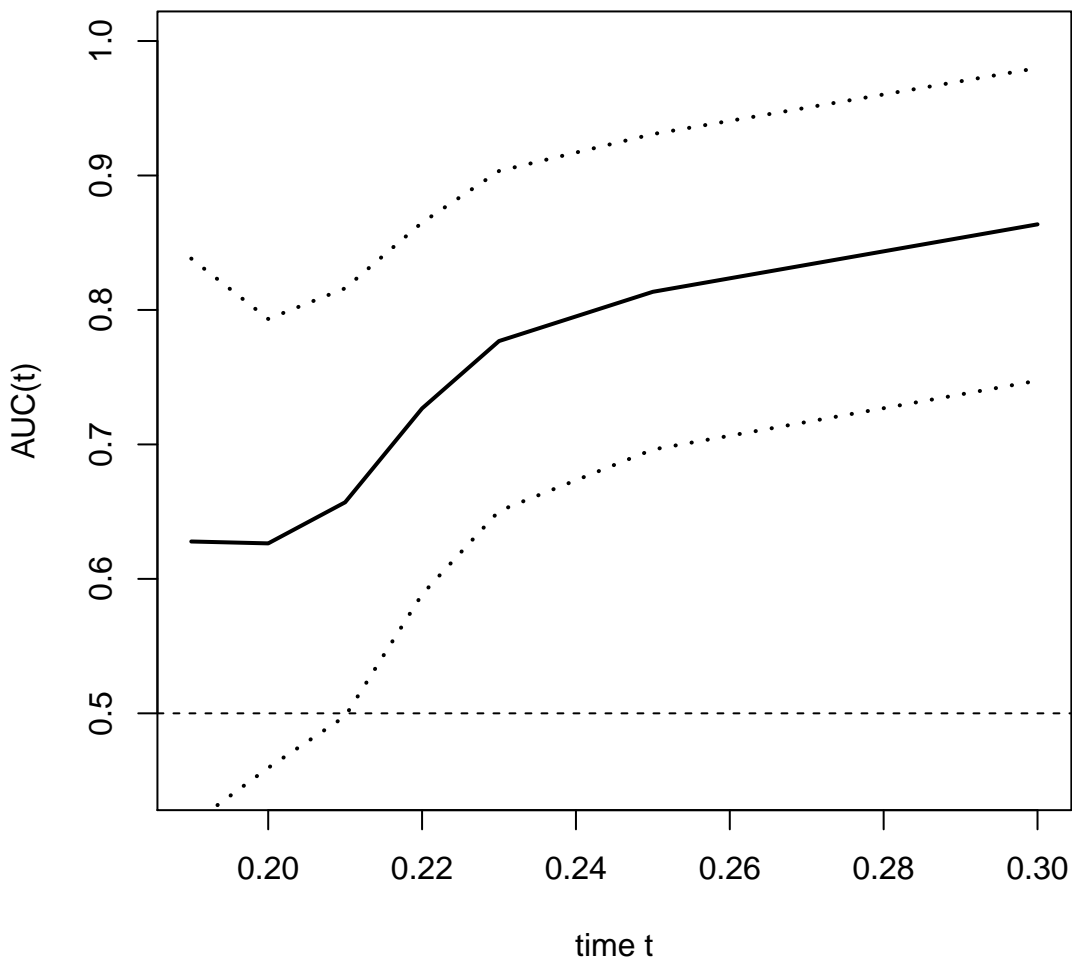

Supplement: S1 Code — (ZIP) [file pmed.1005088.s002.zip › S2 code/PROJ8_3_tbl/PROJ8_3_tbl_mfp_auc.pdf]

## ROC curves

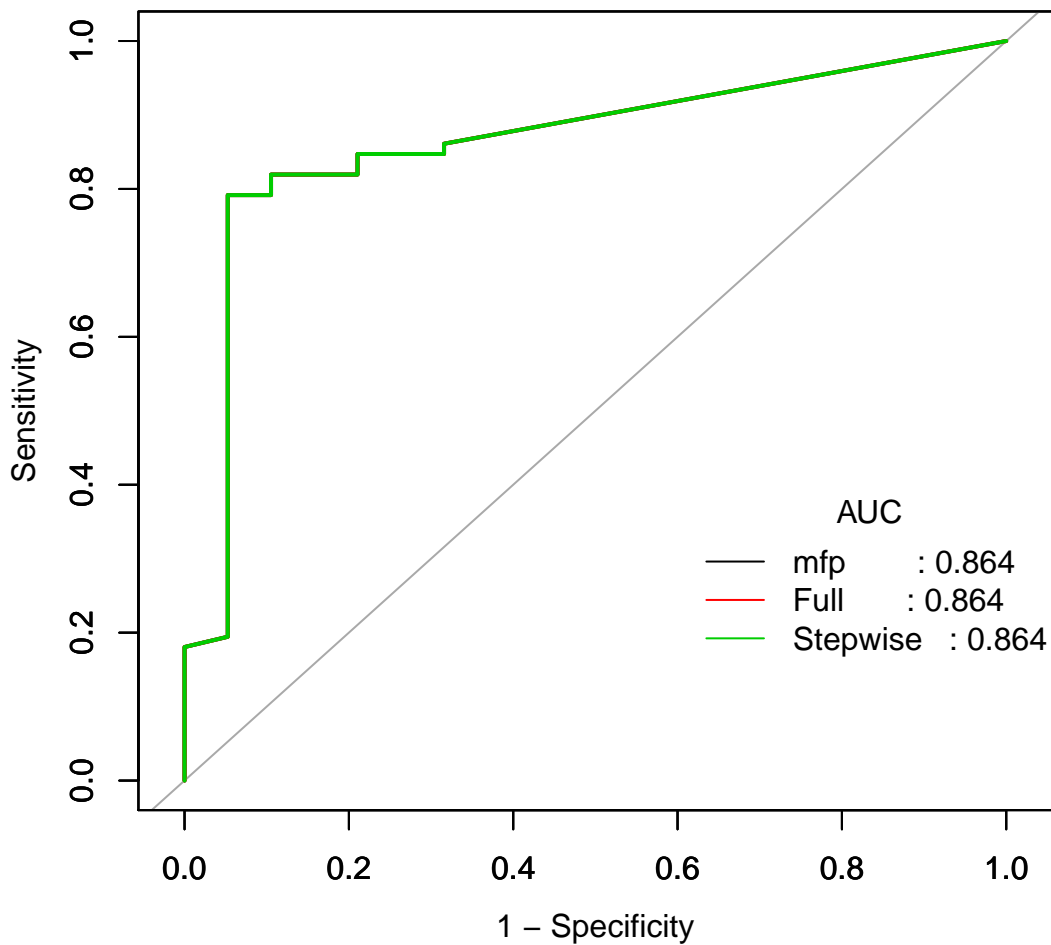

Supplement: S1 Code — (ZIP) [file pmed.1005088.s002.zip › S2 code/PROJ8_3_tbl/PROJ8_3_tbl_rocs.pdf]

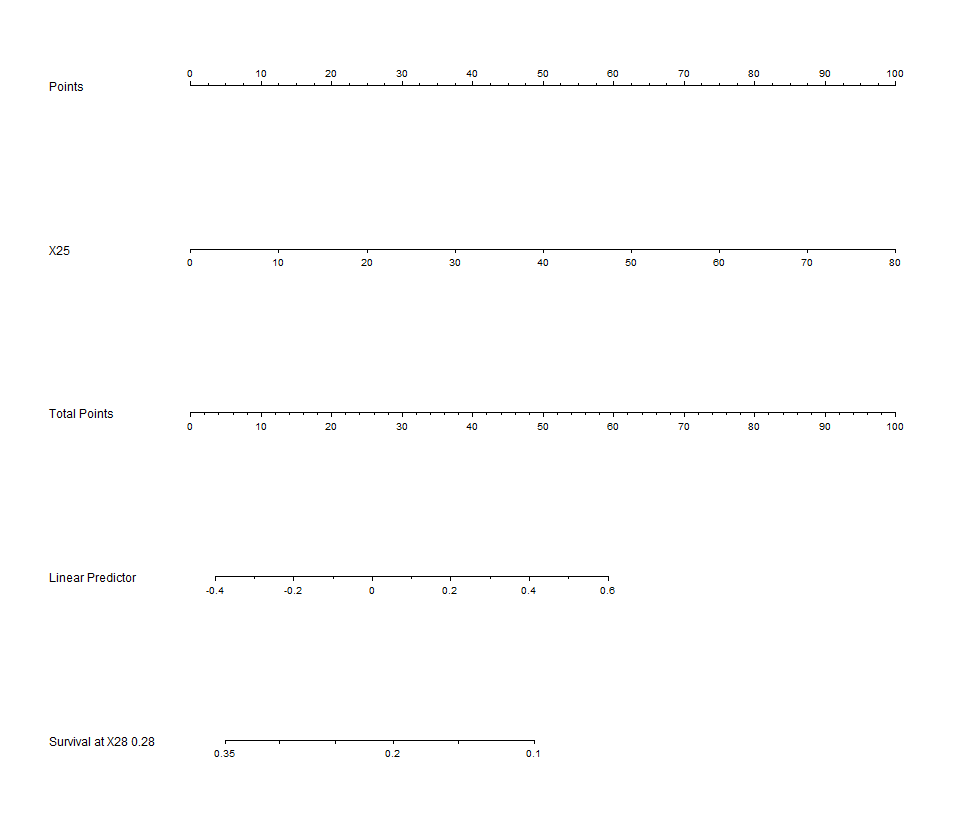

Supplement: S1 Code — (ZIP) [file pmed.1005088.s002.zip › S2 code/PROJ8_3_tbl/PROJ8_3_tbl_full_nom.png]

Points

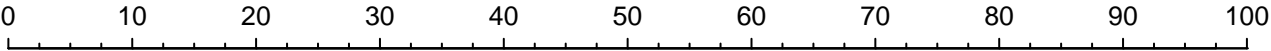

X25

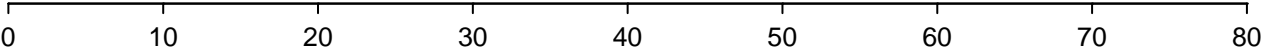

Total Points

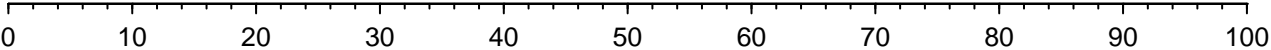

Linear Predictor

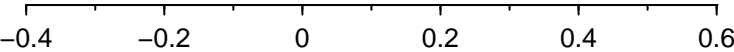

Survival at X28 0.28

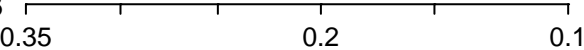

Supplement: S1 Code — (ZIP) [file pmed.1005088.s002.zip › S2 code/PROJ8_3_tbl/PROJ8_3_tbl_full_nom.pdf]

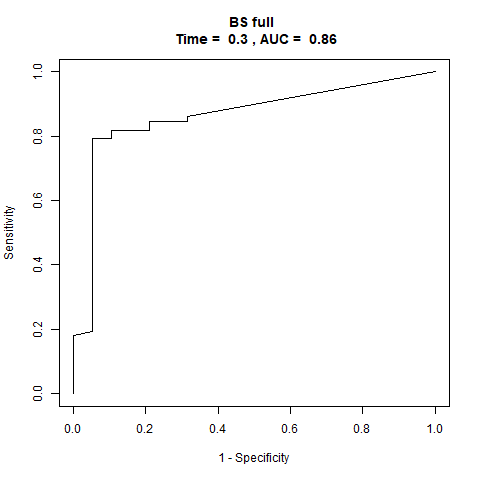

Supplement: S1 Code — (ZIP) [file pmed.1005088.s002.zip › S2 code/PROJ8_3_tbl/PROJ8_3_tbl_bootstrap_full_roc.png]

**BS full**  
**Time = 0.3 , AUC = 0.86**

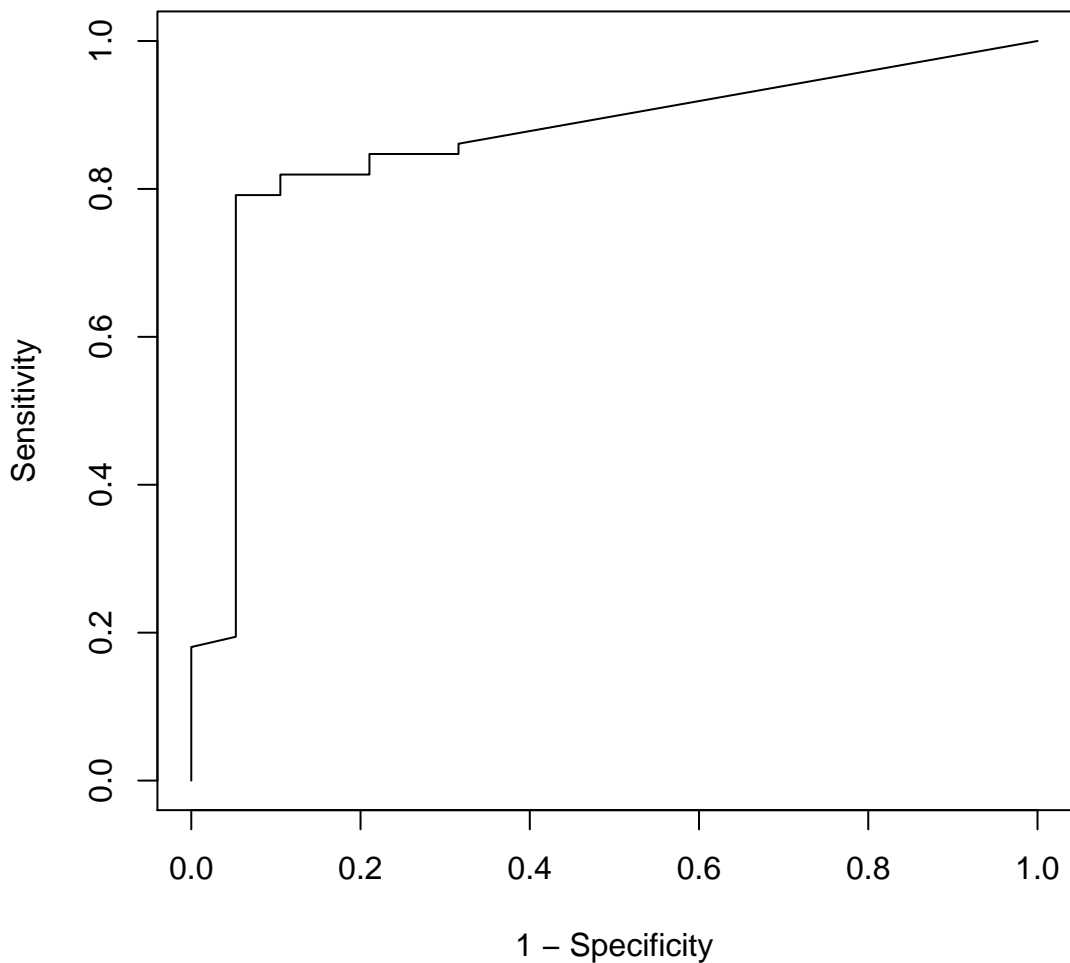

Supplement: S1 Code — (ZIP) [file pmed.1005088.s002.zip › S2 code/PROJ8_3_tbl/PROJ8_3_tbl_bootstrap_full_roc.pdf]

**Calibration curve**  
**Full**

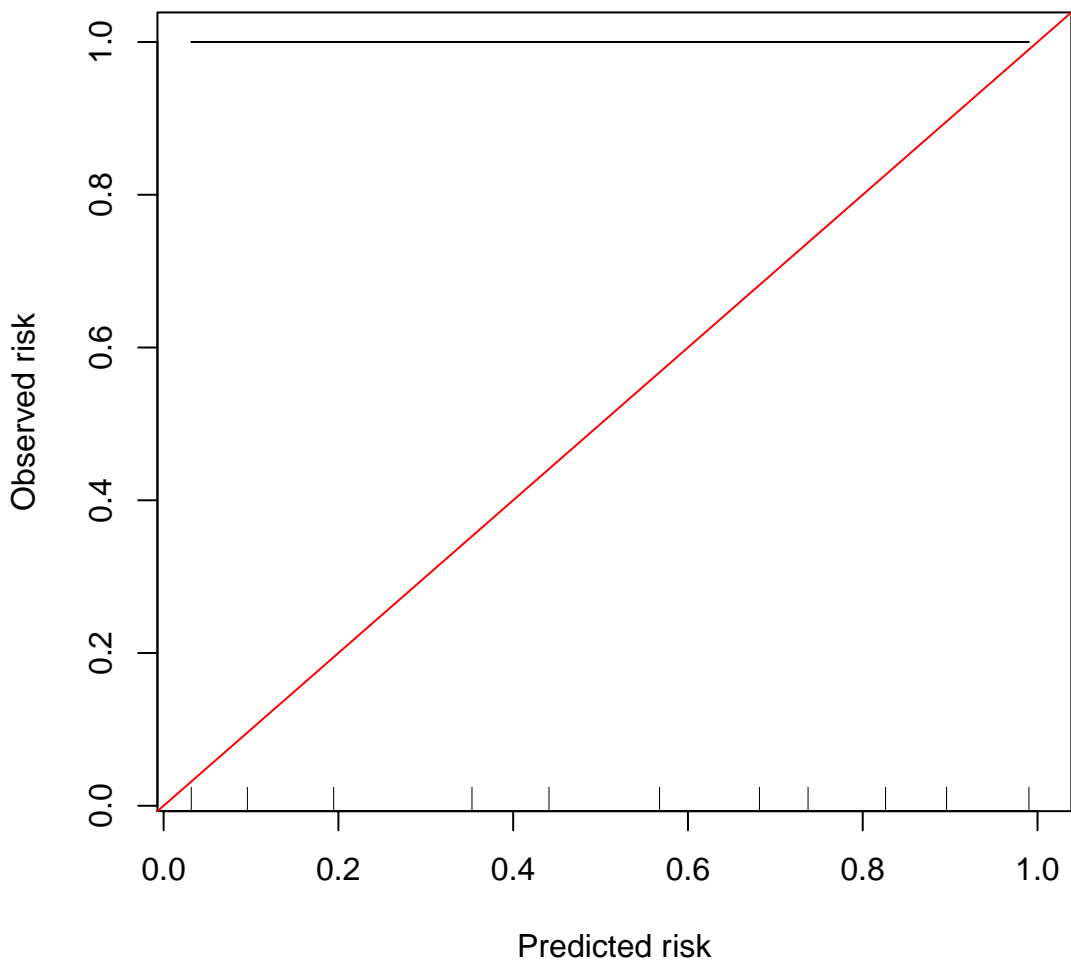

Supplement: S1 Code — (ZIP) [file pmed.1005088.s002.zip › S2 code/PROJ8_3_tbl/PROJ8_3_tbl_full_cal.pdf]

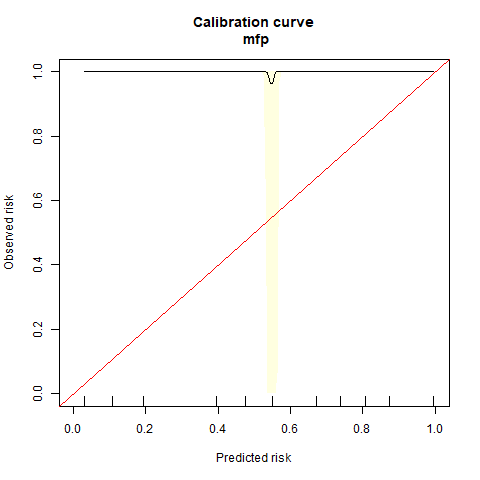

Supplement: S1 Code — (ZIP) [file pmed.1005088.s002.zip › S2 code/PROJ8_3_tbl/PROJ8_3_tbl_mfp_cal.png]

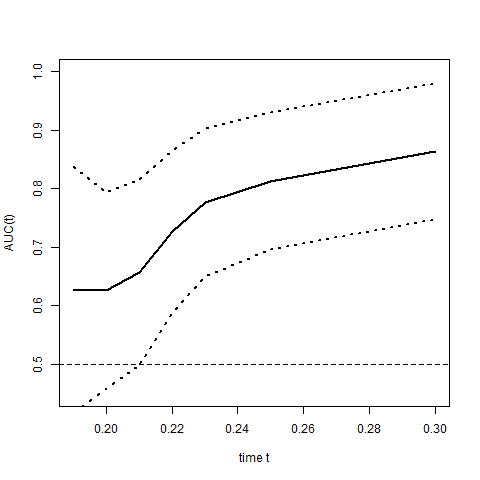

Supplement: S1 Code — (ZIP) [file pmed.1005088.s002.zip › S2 code/PROJ8_3_tbl/PROJ8_3_tbl_bootstrap_full_auc.png]

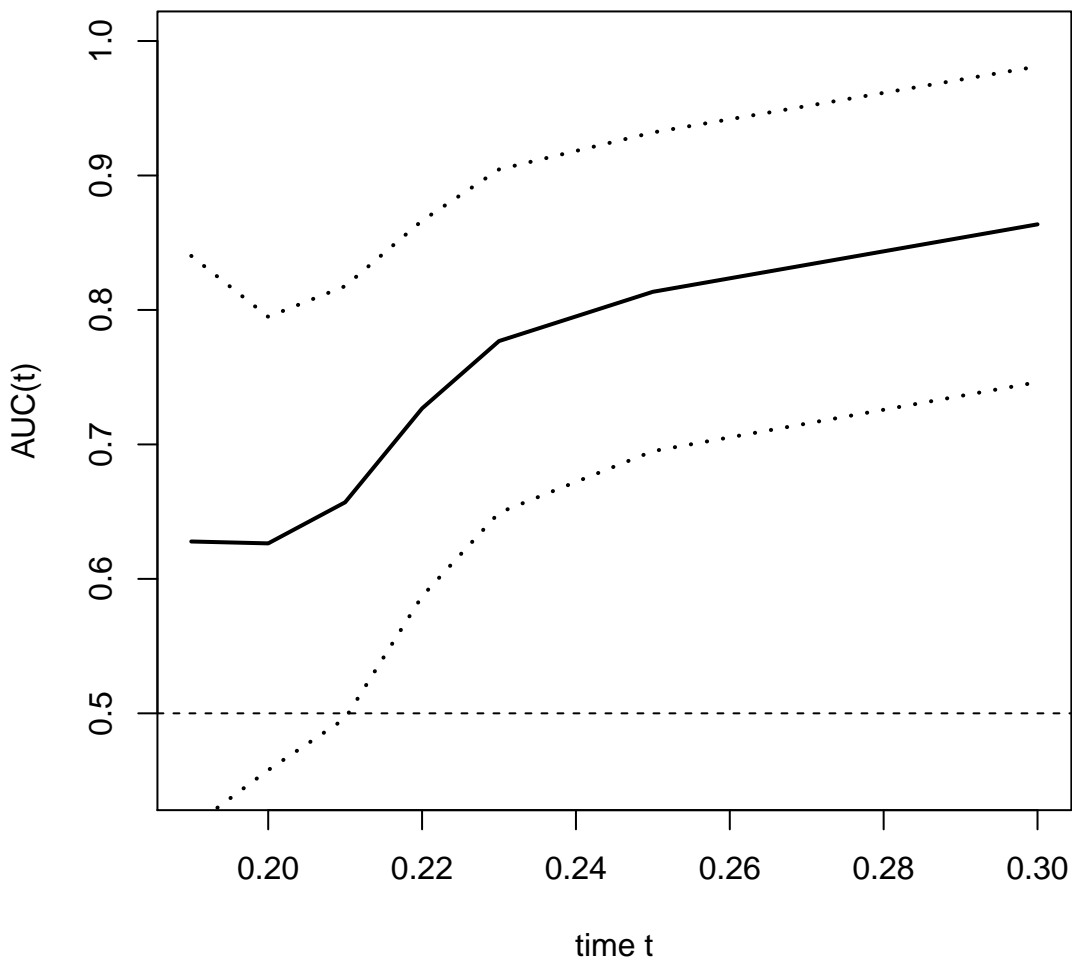

Supplement: S1 Code — (ZIP) [file pmed.1005088.s002.zip › S2 code/PROJ8_3_tbl/PROJ8_3_tbl_bootstrap_full_auc.pdf]

**Calibration curve**  
**mfp**

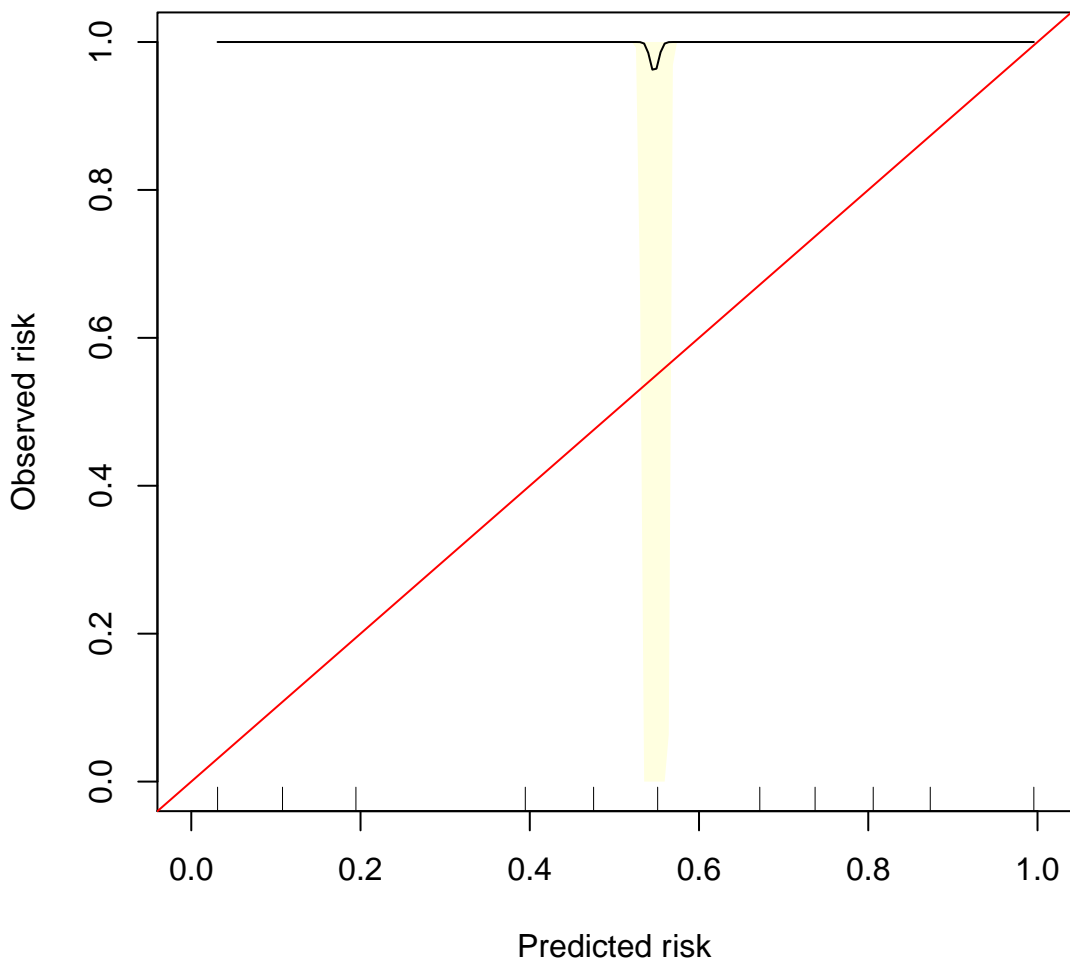

Supplement: S1 Code — (ZIP) [file pmed.1005088.s002.zip › S2 code/PROJ8_3_tbl/PROJ8_3_tbl_mfp_cal.pdf]

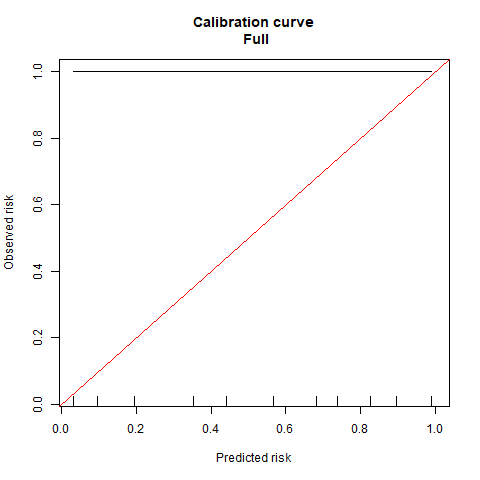

Supplement: S1 Code — (ZIP) [file pmed.1005088.s002.zip › S2 code/PROJ8_3_tbl/PROJ8_3_tbl_full_cal.png]

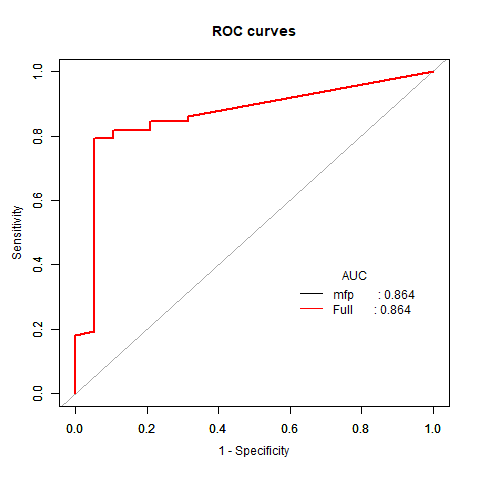

Supplement: S1 Code — (ZIP) [file pmed.1005088.s002.zip › S2 code/PROJ8_2_tbl/PROJ8_2_tbl_rocs.png]

# ROC curves

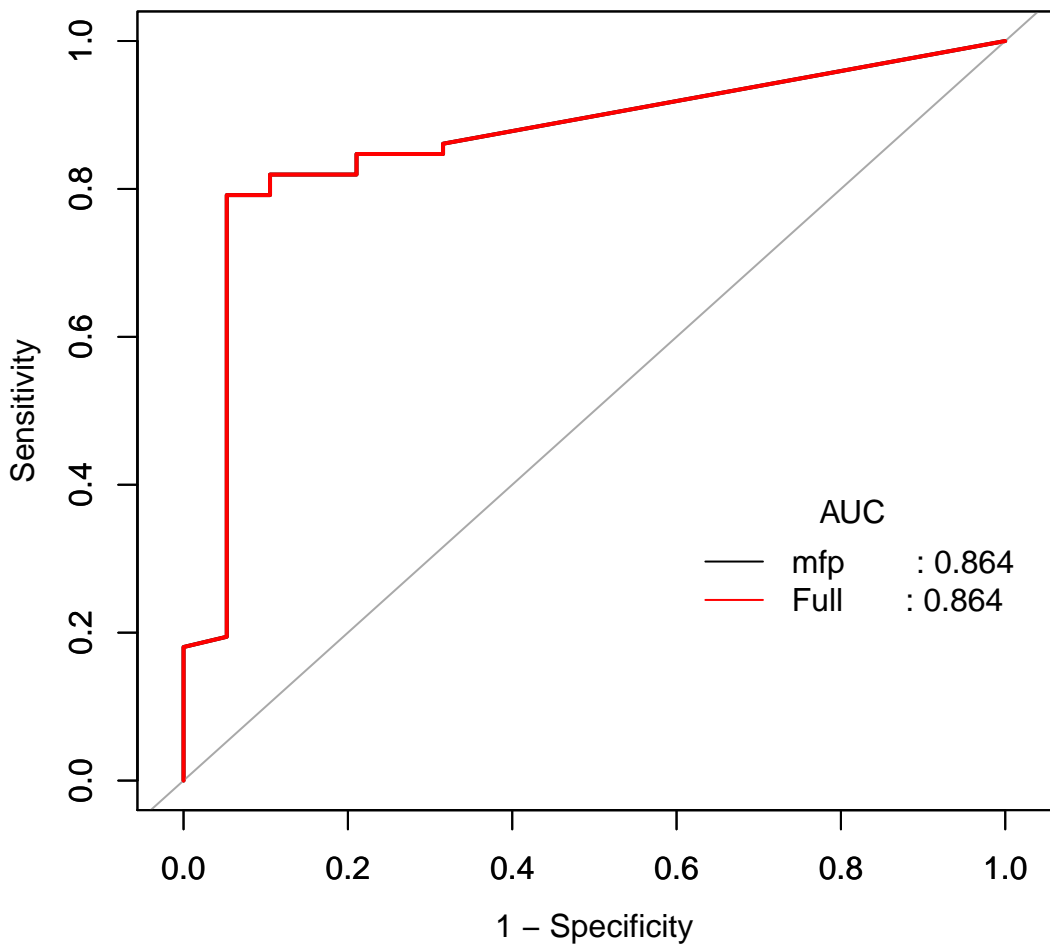

Supplement: S1 Code — (ZIP) [file pmed.1005088.s002.zip › S2 code/PROJ8_2_tbl/PROJ8_2_tbl_rocs.pdf]

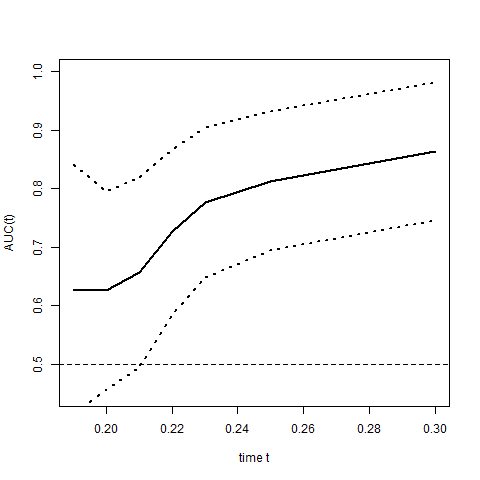

Supplement: S1 Code — (ZIP) [file pmed.1005088.s002.zip › S2 code/PROJ8_2_tbl/PROJ8_2_tbl_mfp_auc.png]

**Full**  
**Time = 0.3 , AUC = 0.86**

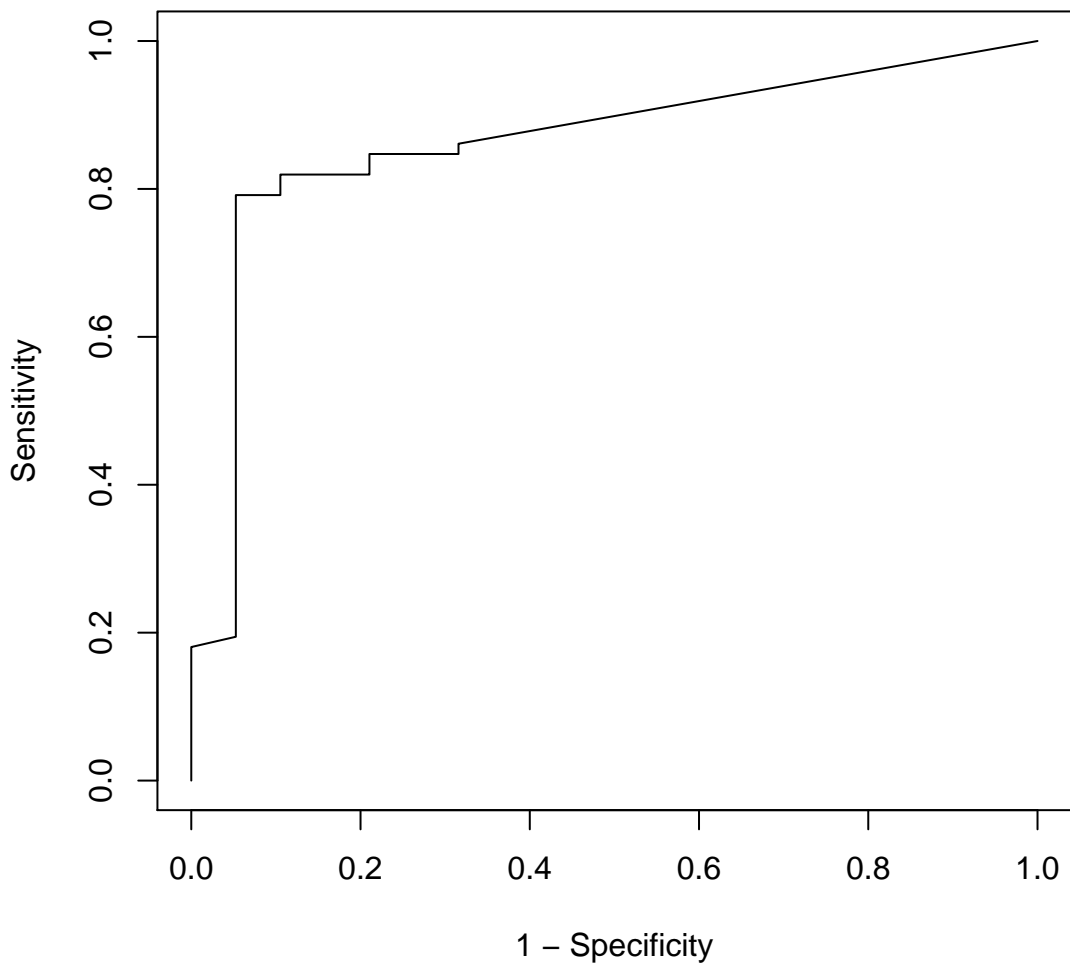

Supplement: S1 Code — (ZIP) [file pmed.1005088.s002.zip › S2 code/PROJ8_2_tbl/PROJ8_2_tbl_full_roc.pdf]

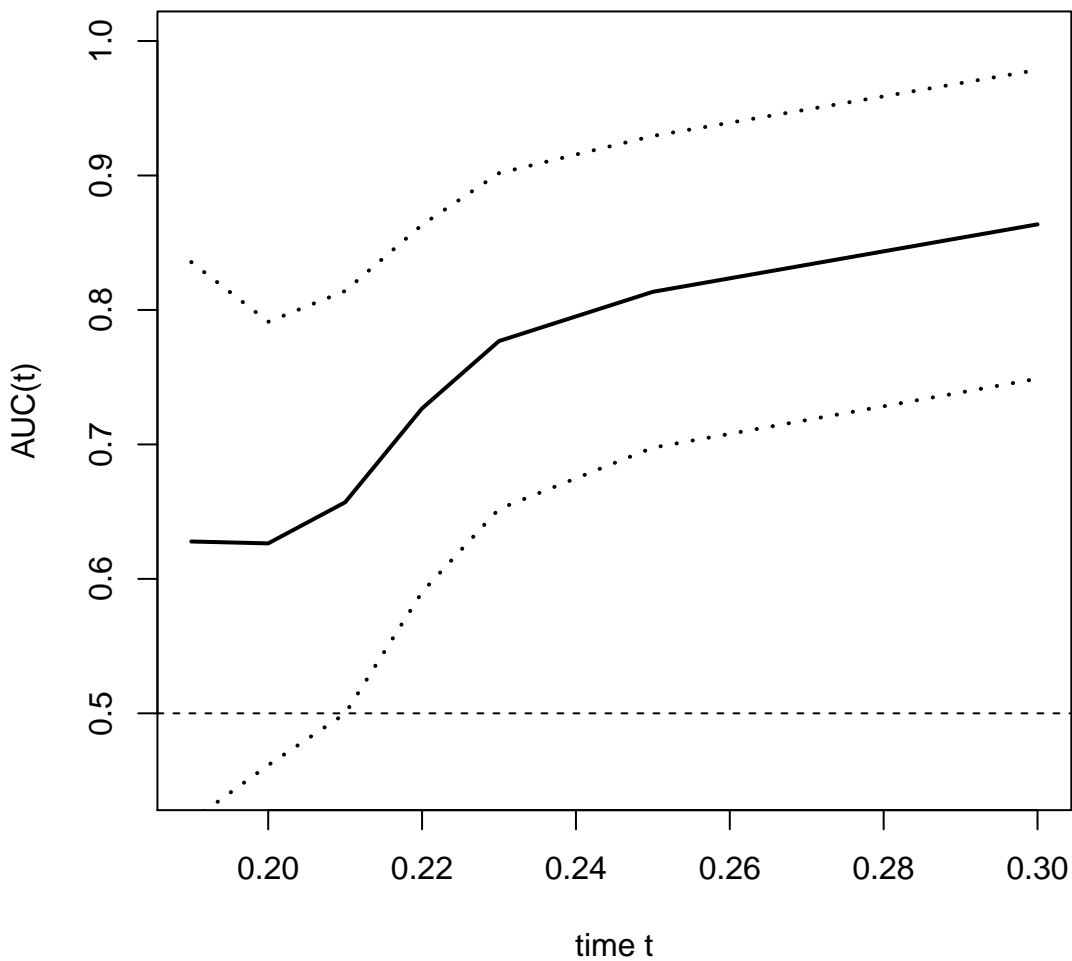

Supplement: S1 Code — (ZIP) [file pmed.1005088.s002.zip › S2 code/PROJ8_2_tbl/PROJ8_2_tbl_mfp_auc.pdf]

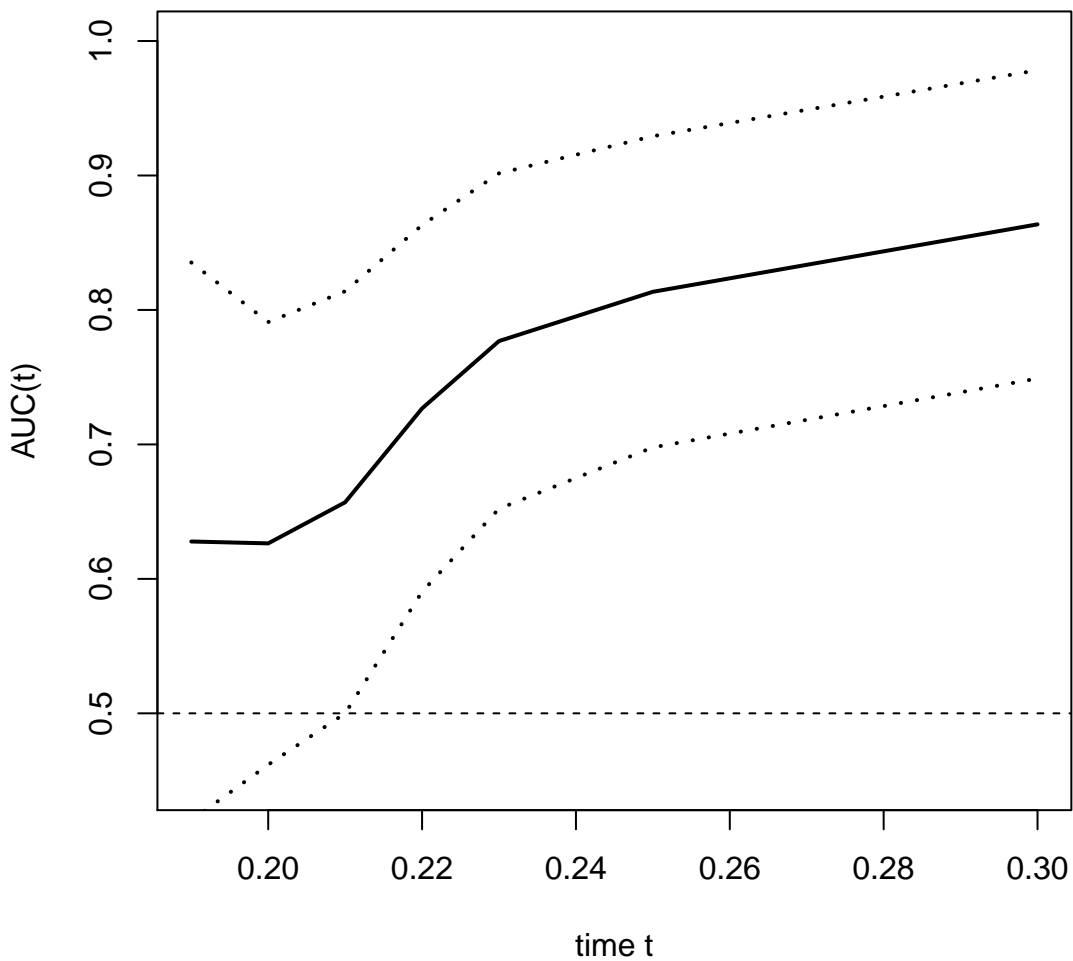

Supplement: S1 Code — (ZIP) [file pmed.1005088.s002.zip › S2 code/PROJ8_2_tbl/PROJ8_2_tbl_full_auc.pdf]

mfp  
Time = 0.3 , AUC = 0.86

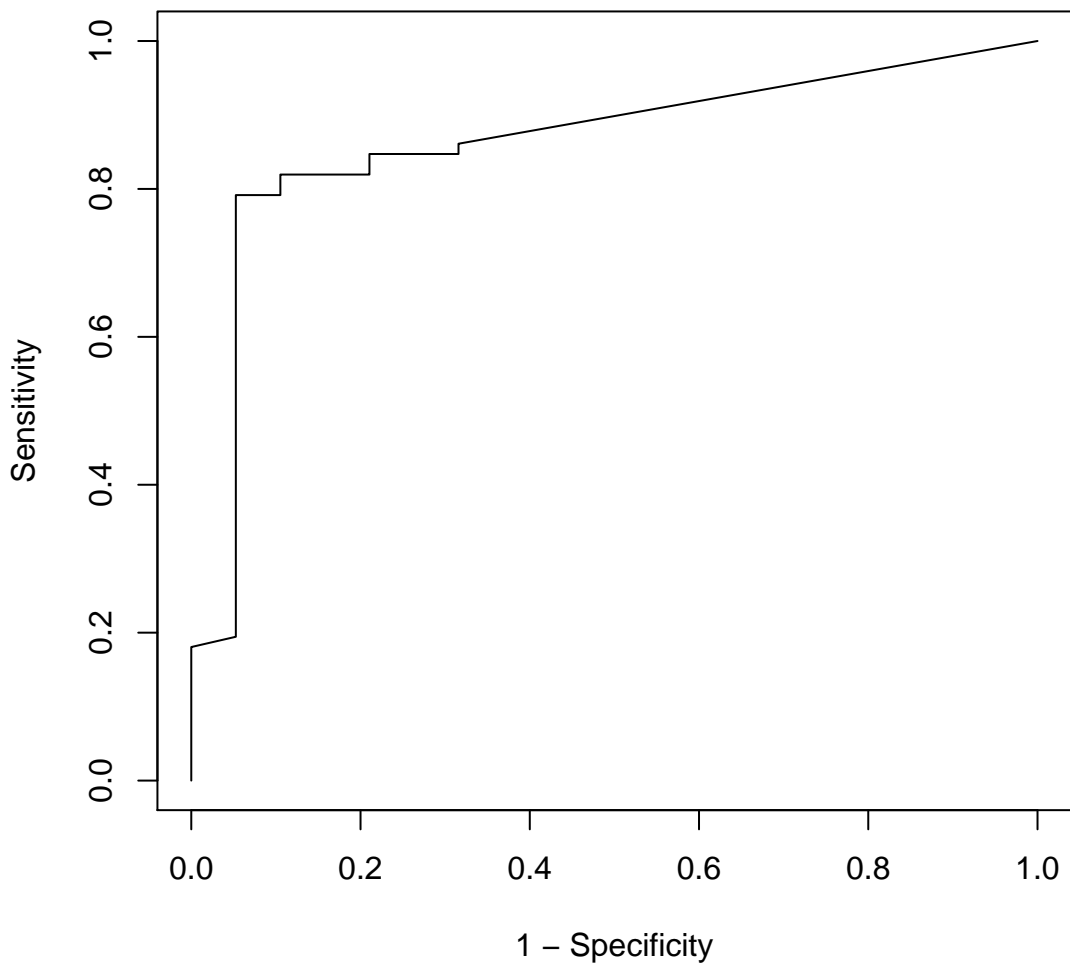

Supplement: S1 Code — (ZIP) [file pmed.1005088.s002.zip › S2 code/PROJ8_2_tbl/PROJ8_2_tbl_mfp_roc.pdf]

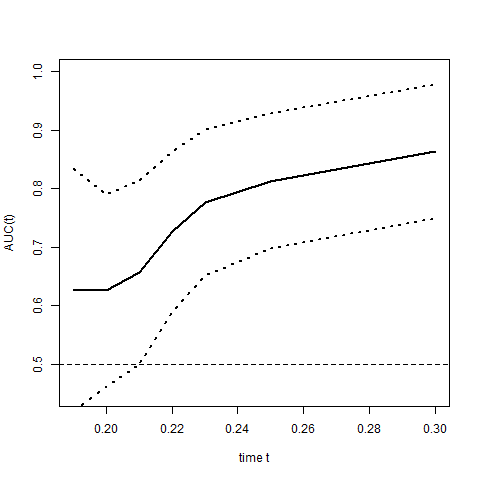

Supplement: S1 Code — (ZIP) [file pmed.1005088.s002.zip › S2 code/PROJ8_2_tbl/PROJ8_2_tbl_full_auc.png]

**Calibration curve**  
**mfp**

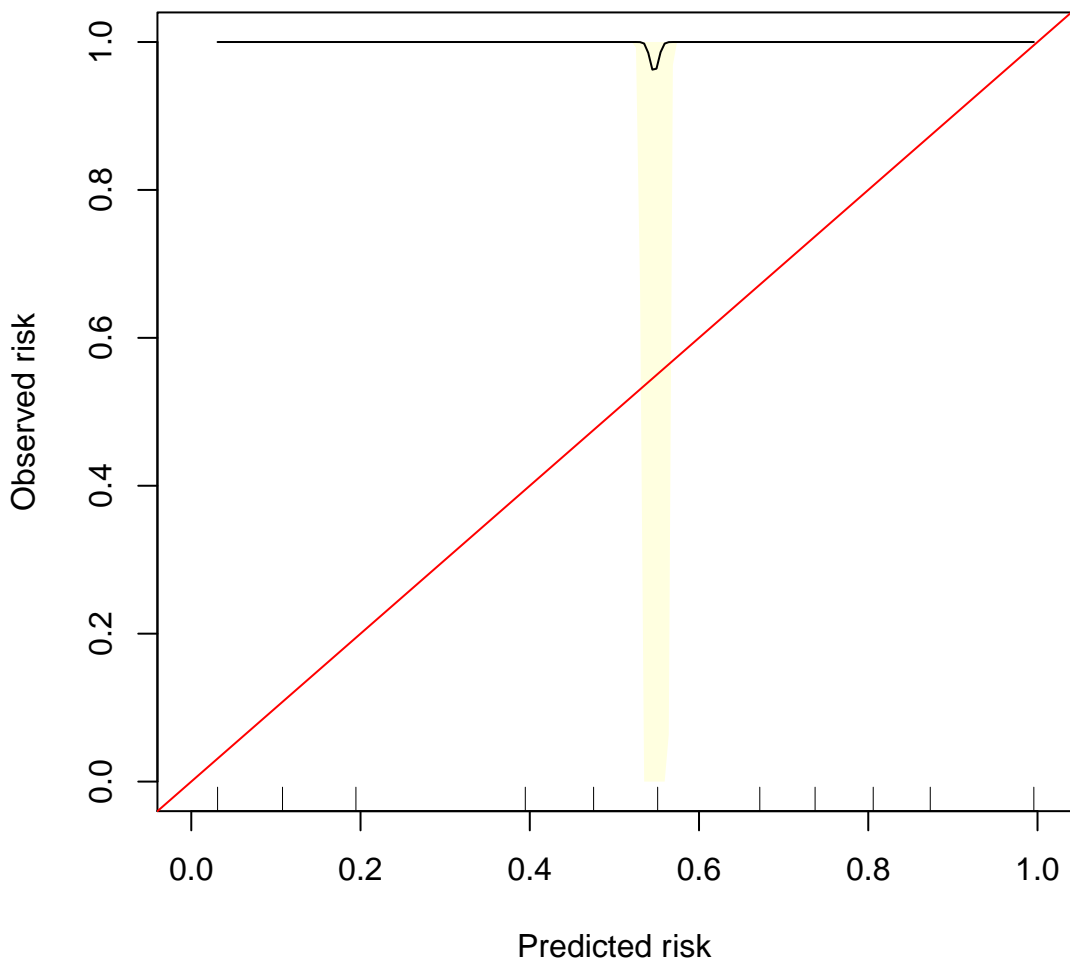

Supplement: S1 Code — (ZIP) [file pmed.1005088.s002.zip › S2 code/PROJ8_2_tbl/PROJ8_2_tbl_mfp_cal.pdf]

**Calibration curve**  
**Full**

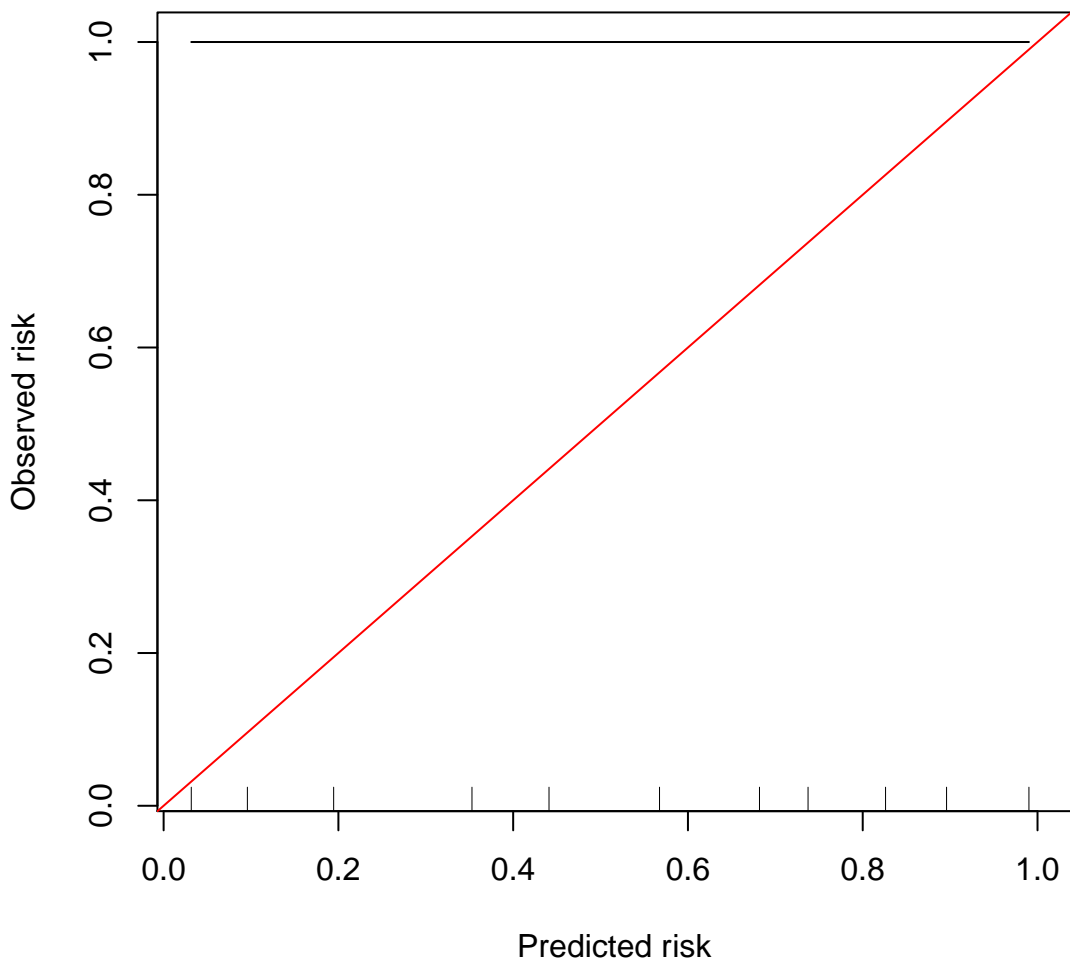

Supplement: S1 Code — (ZIP) [file pmed.1005088.s002.zip › S2 code/PROJ8_2_tbl/PROJ8_2_tbl_full_cal.pdf]

Points

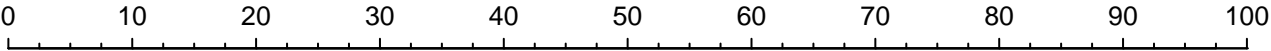

X25

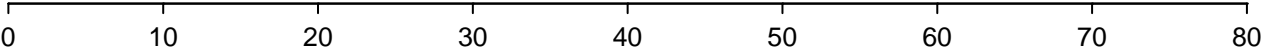

Total Points

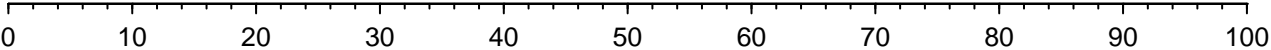

Linear Predictor

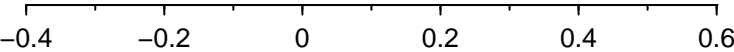

Survival at X28 0.28

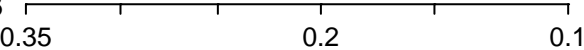

Supplement: S1 Code — (ZIP) [file pmed.1005088.s002.zip › S2 code/PROJ8_2_tbl/PROJ8_2_tbl_full_nom.pdf]

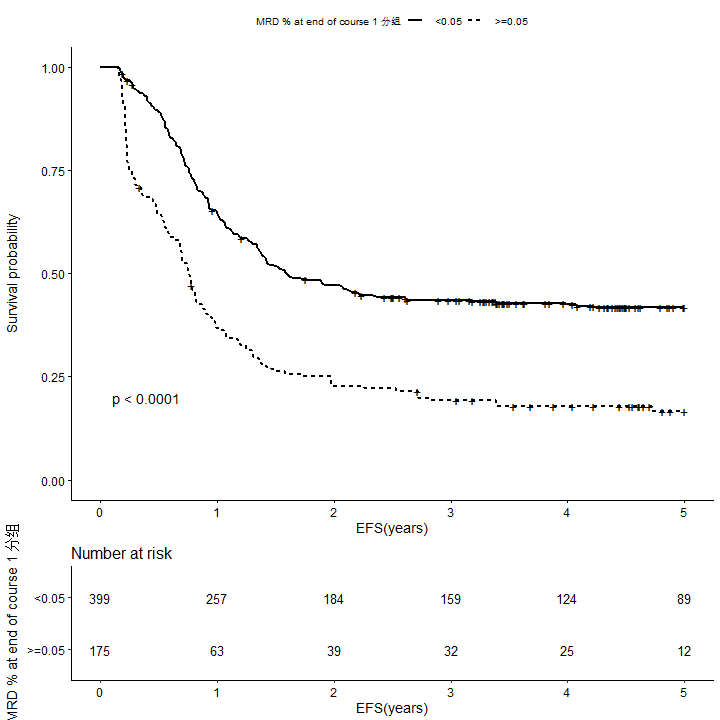

Supplement: S1 Code — (ZIP) [file pmed.1005088.s002.zip › S2 code/PROJ8_13_tbl1/PROJ8_13_tbl1_seg1_b.png]

MRD % at end of course 1 ..... — <0.05 - - - ≥0.05

Survival probability

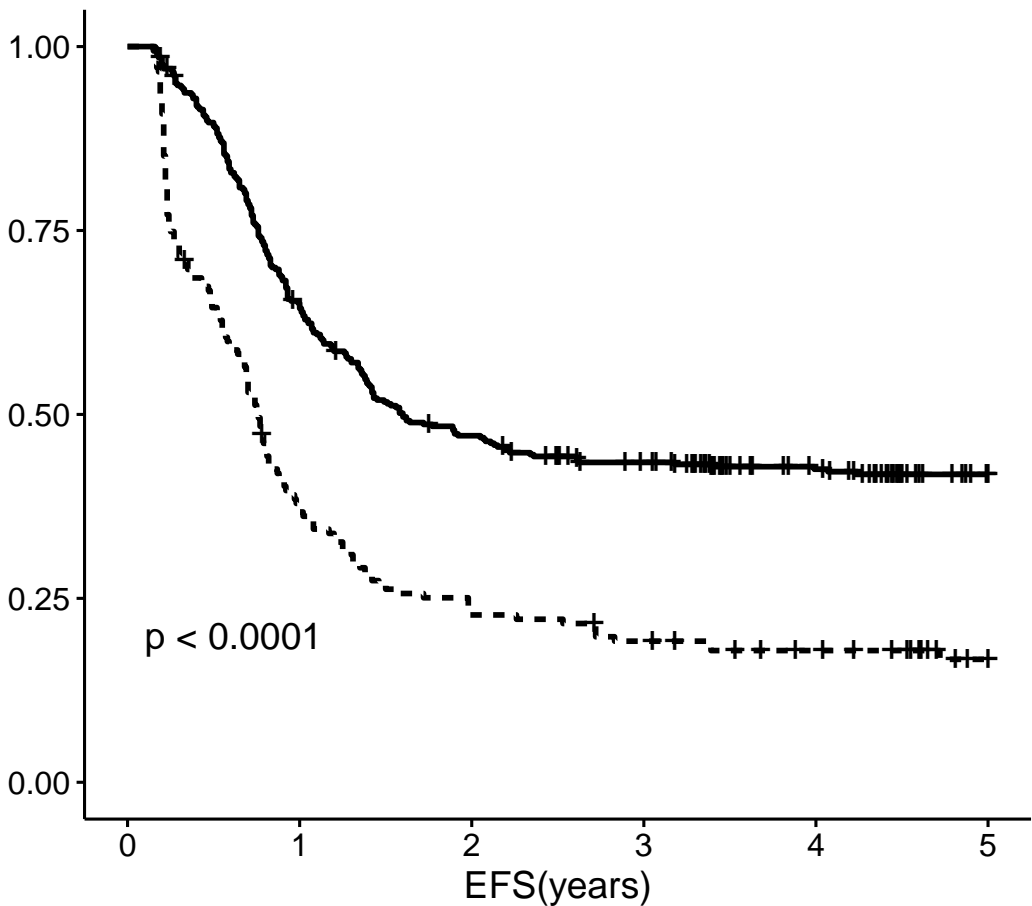

Number at risk

|       |            |     |     |     |     |    |
|-------|------------|-----|-----|-----|-----|----|
| <0.05 | 399        | 257 | 184 | 159 | 124 | 89 |
| ≥0.05 | 175        | 63  | 39  | 32  | 25  | 12 |
|       | 0          | 1   | 2   | 3   | 4   | 5  |
|       | EFS(years) |     |     |     |     |    |

Supplement: S1 Code — (ZIP) [file pmed.1005088.s002.zip › S2 code/PROJ8_13_tbl1/PROJ8_13_tbl1_seg1_b.pdf]

## First Event

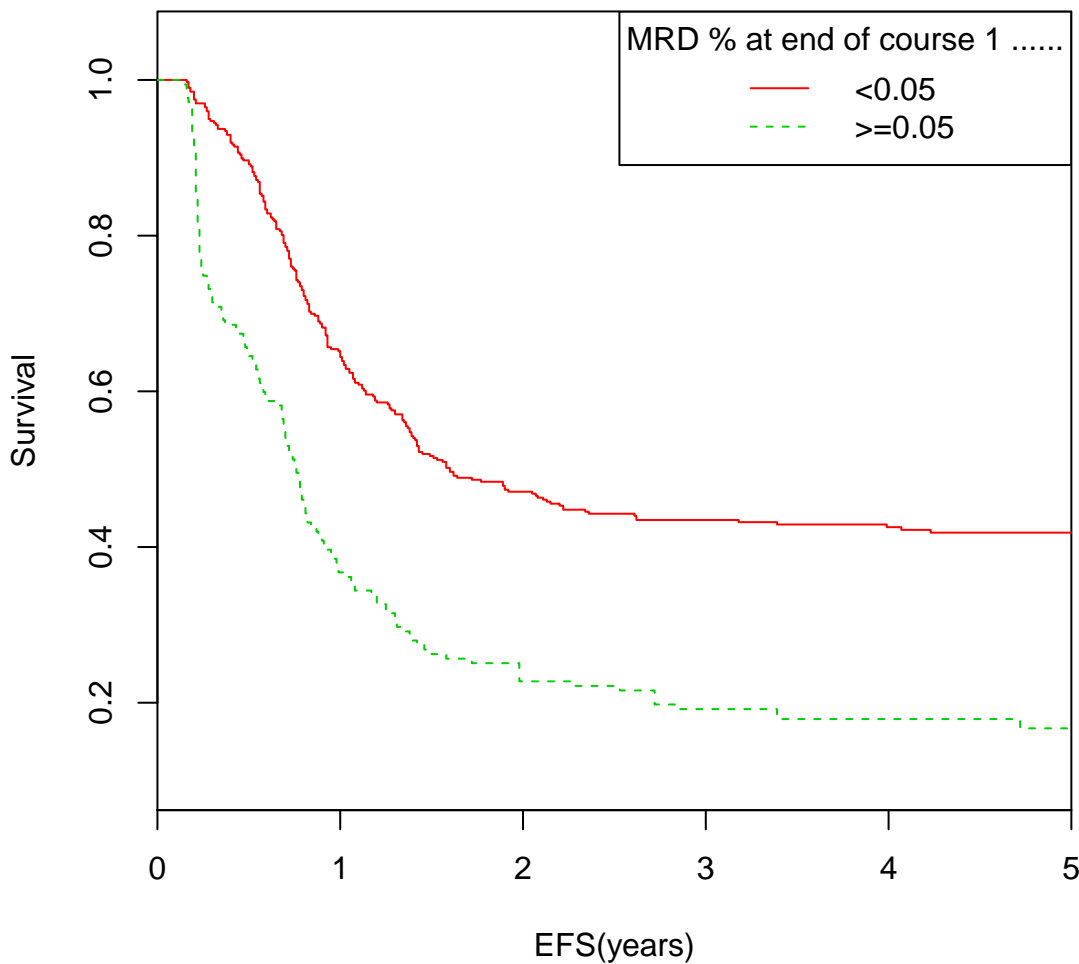

Supplement: S1 Code — (ZIP) [file pmed.1005088.s002.zip › S2 code/PROJ8_13_tbl1/PROJ8_13_tbl1_seg1_1.pdf]

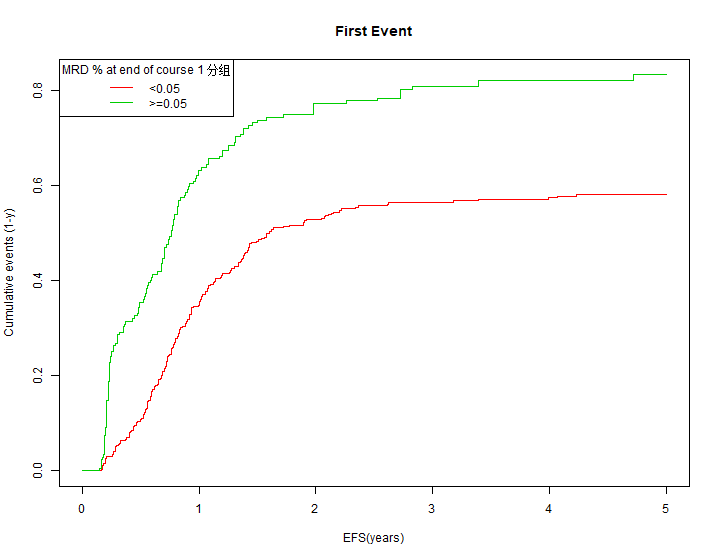

Supplement: S1 Code — (ZIP) [file pmed.1005088.s002.zip › S2 code/PROJ8_13_tbl1/PROJ8_13_tbl1_seg1_3.png]

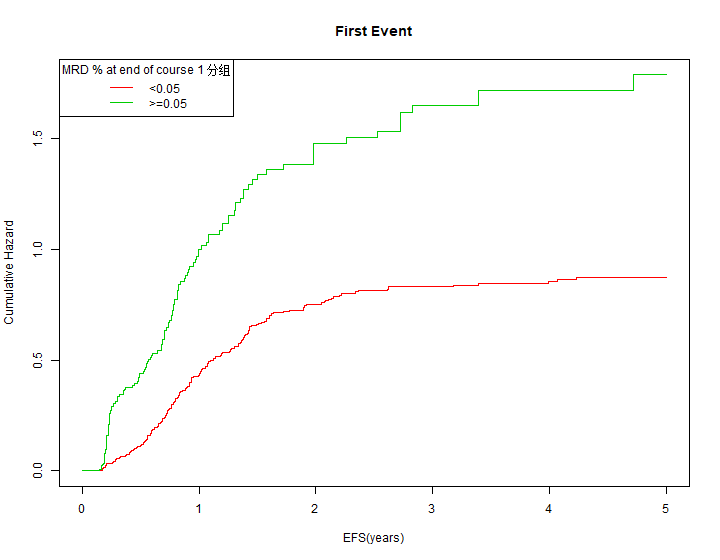

Supplement: S1 Code — (ZIP) [file pmed.1005088.s002.zip › S2 code/PROJ8_13_tbl1/PROJ8_13_tbl1_seg1_2.png]

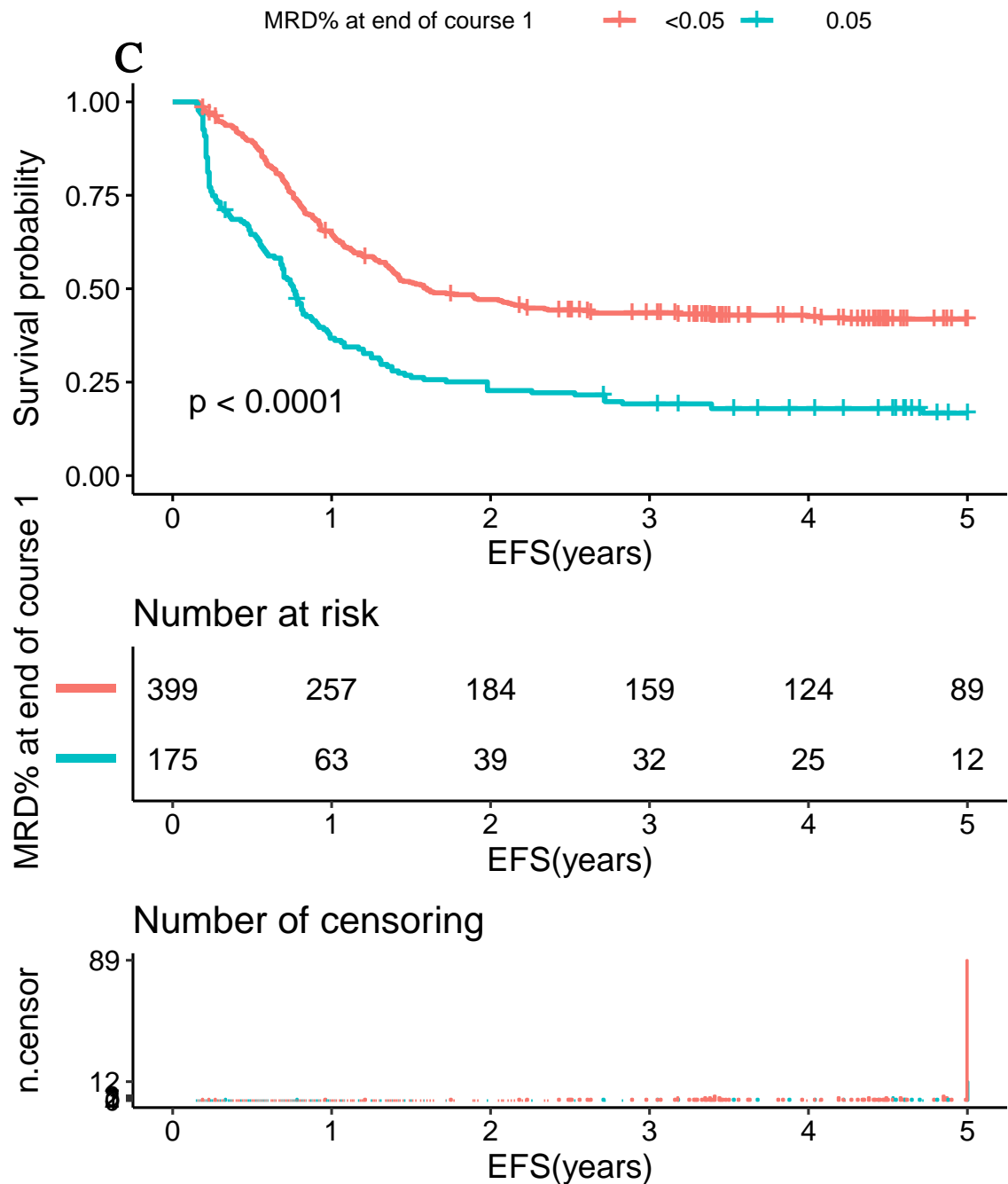

Supplement: S1 Code — (ZIP) [file pmed.1005088.s002.zip › S2 code/PROJ8_13_tbl1/PROJ8_13_tbl1_seg1_0.pdf]

## First Event

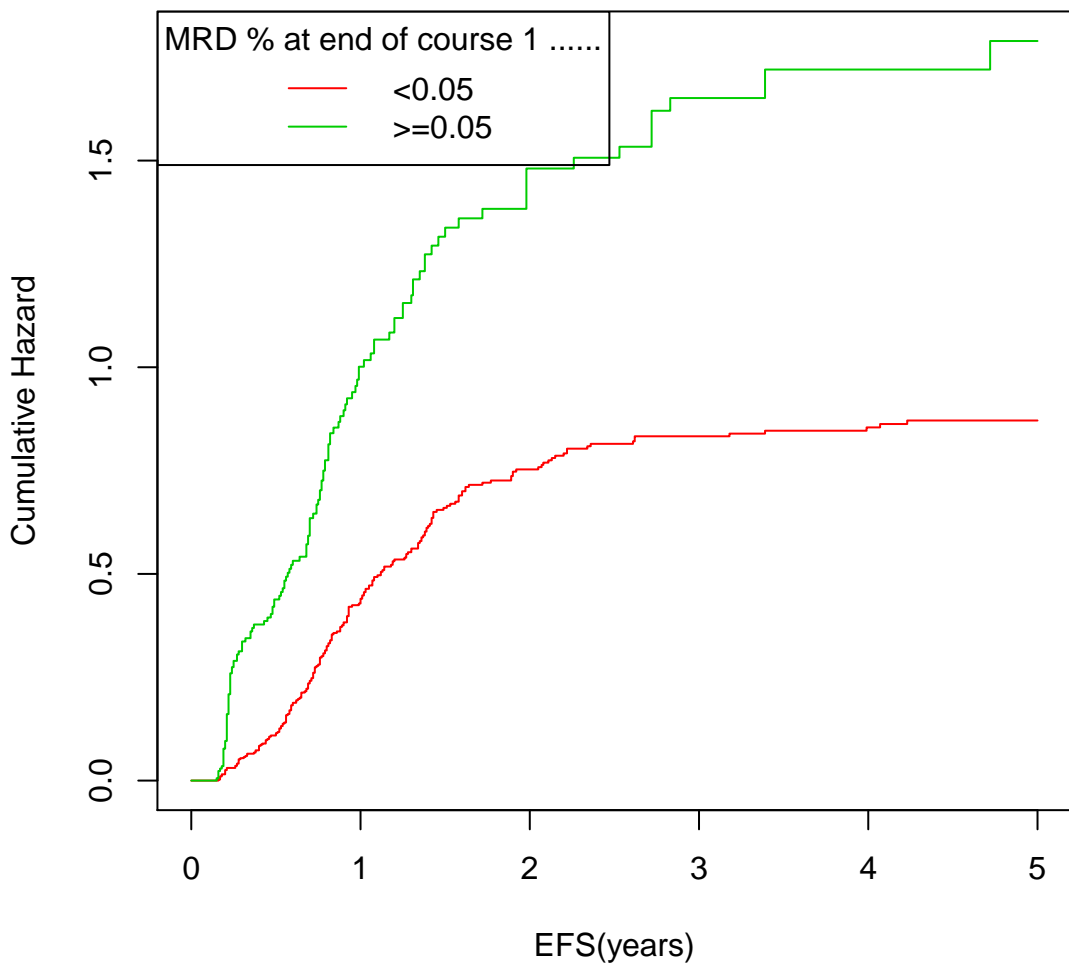

Supplement: S1 Code — (ZIP) [file pmed.1005088.s002.zip › S2 code/PROJ8_13_tbl1/PROJ8_13_tbl1_seg1_2.pdf]

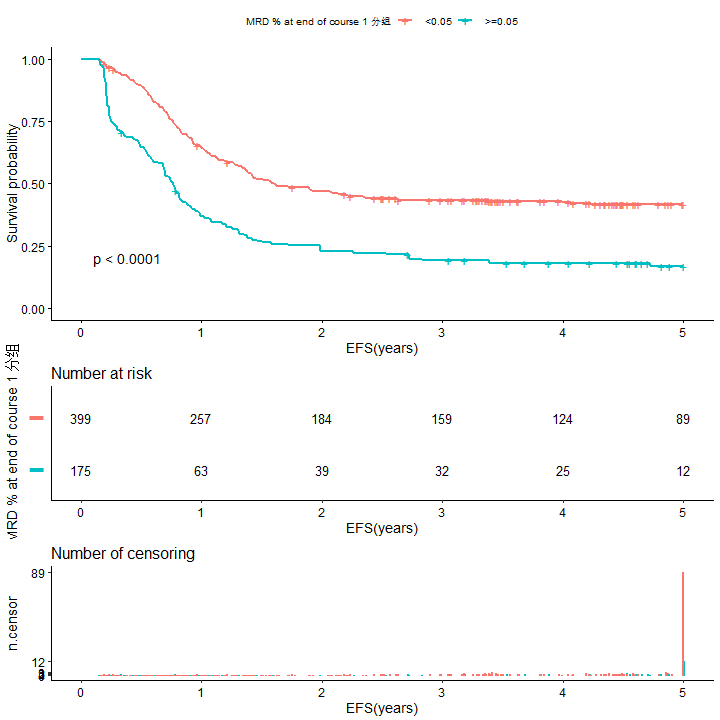

Supplement: S1 Code — (ZIP) [file pmed.1005088.s002.zip › S2 code/PROJ8_13_tbl1/PROJ8_13_tbl1_seg1_0.png]

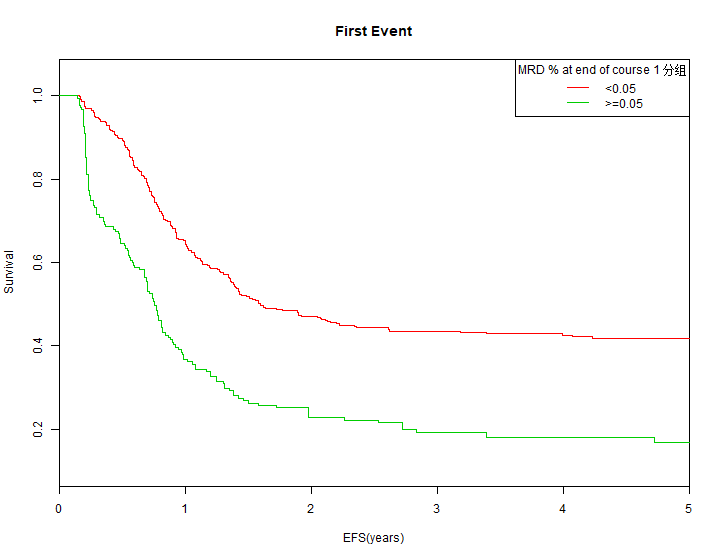

Supplement: S1 Code — (ZIP) [file pmed.1005088.s002.zip › S2 code/PROJ8_13_tbl1/PROJ8_13_tbl1_seg1_1.png]

## First Event

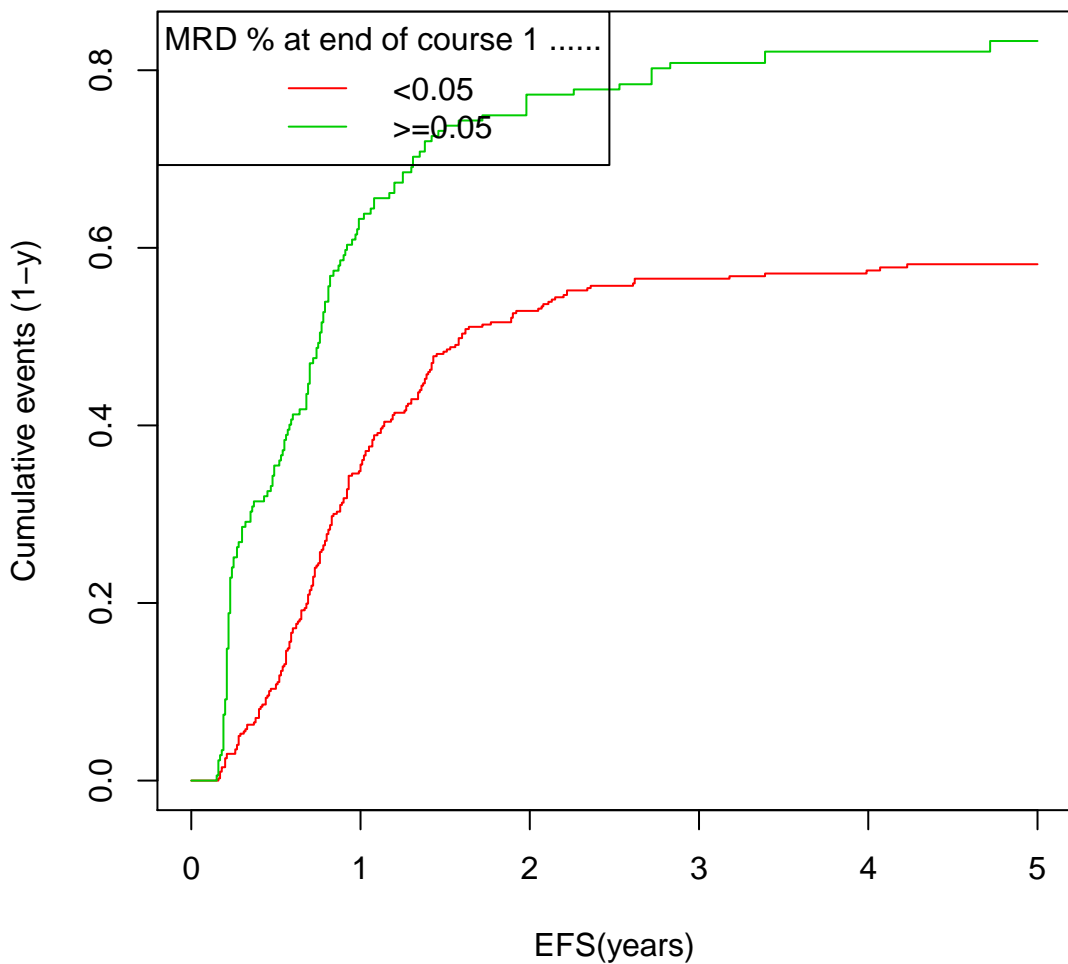

Supplement: S1 Code — (ZIP) [file pmed.1005088.s002.zip › S2 code/PROJ8_13_tbl1/PROJ8_13_tbl1_seg1_3.pdf]

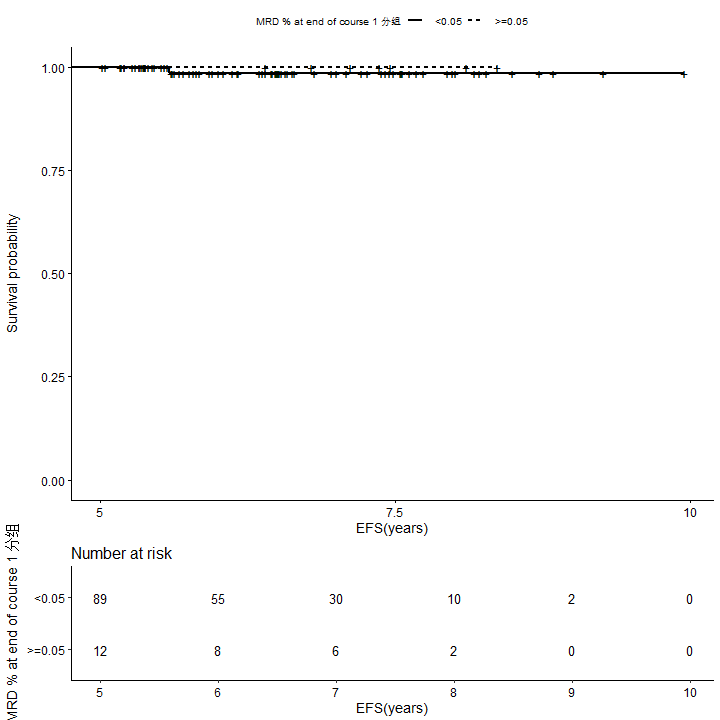

Supplement: S1 Code — (ZIP) [file pmed.1005088.s002.zip › S2 code/PROJ8_13_tbl1/PROJ8_13_tbl1_seg2_b.png]

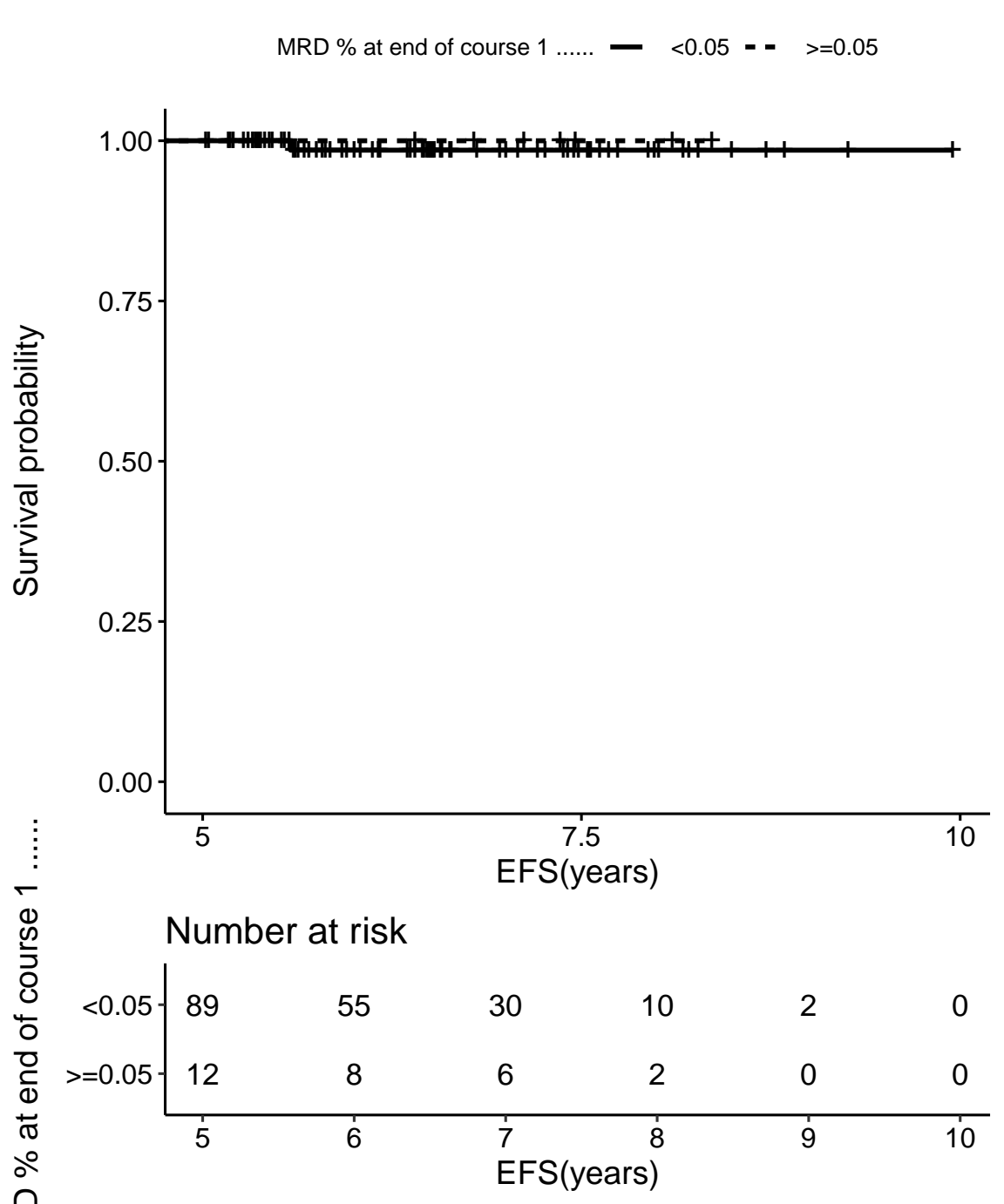

Supplement: S1 Code — (ZIP) [file pmed.1005088.s002.zip › S2 code/PROJ8_13_tbl1/PROJ8_13_tbl1_seg2_b.pdf]

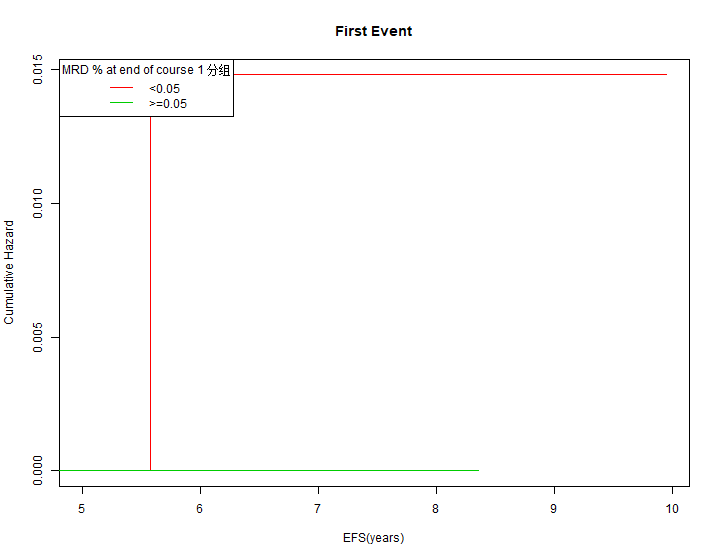

Supplement: S1 Code — (ZIP) [file pmed.1005088.s002.zip › S2 code/PROJ8_13_tbl1/PROJ8_13_tbl1_seg2_2.png]

MRD % at end of course 1 ..... + <0.05 + ≥0.05

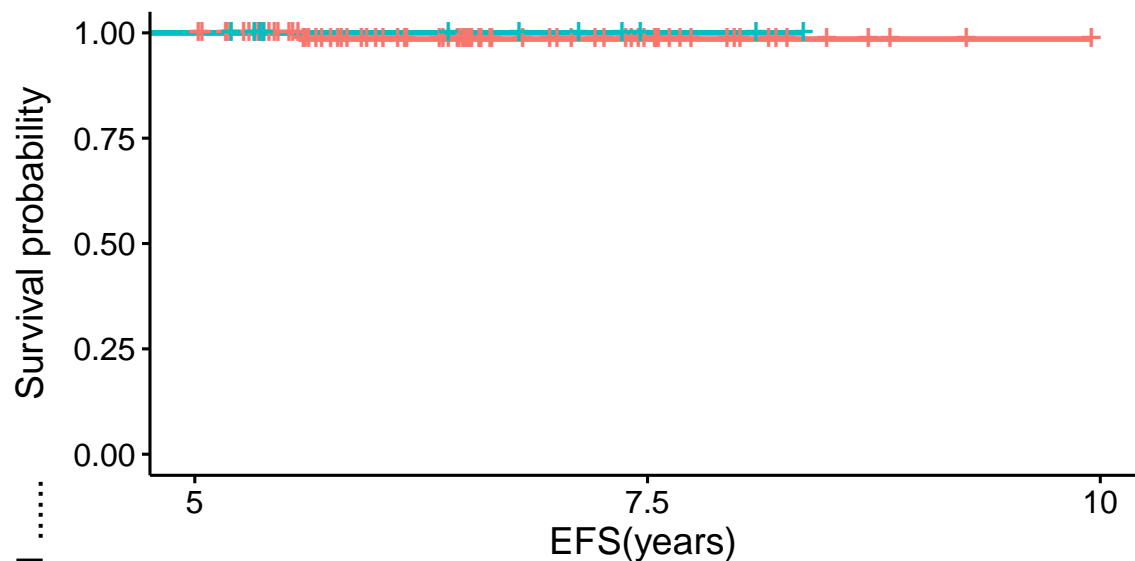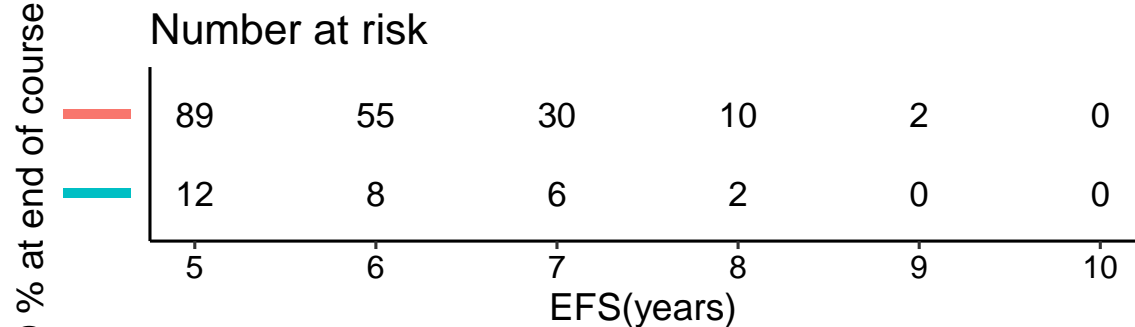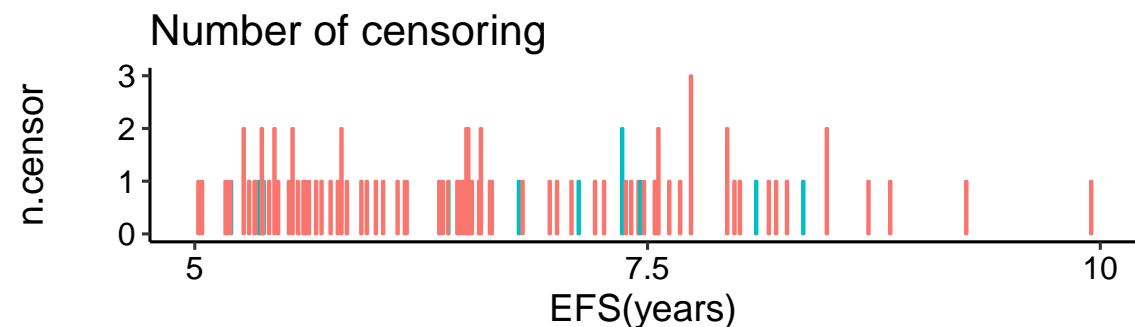

Supplement: S1 Code — (ZIP) [file pmed.1005088.s002.zip › S2 code/PROJ8_13_tbl1/PROJ8_13_tbl1_seg2_0.pdf]

Survival probability

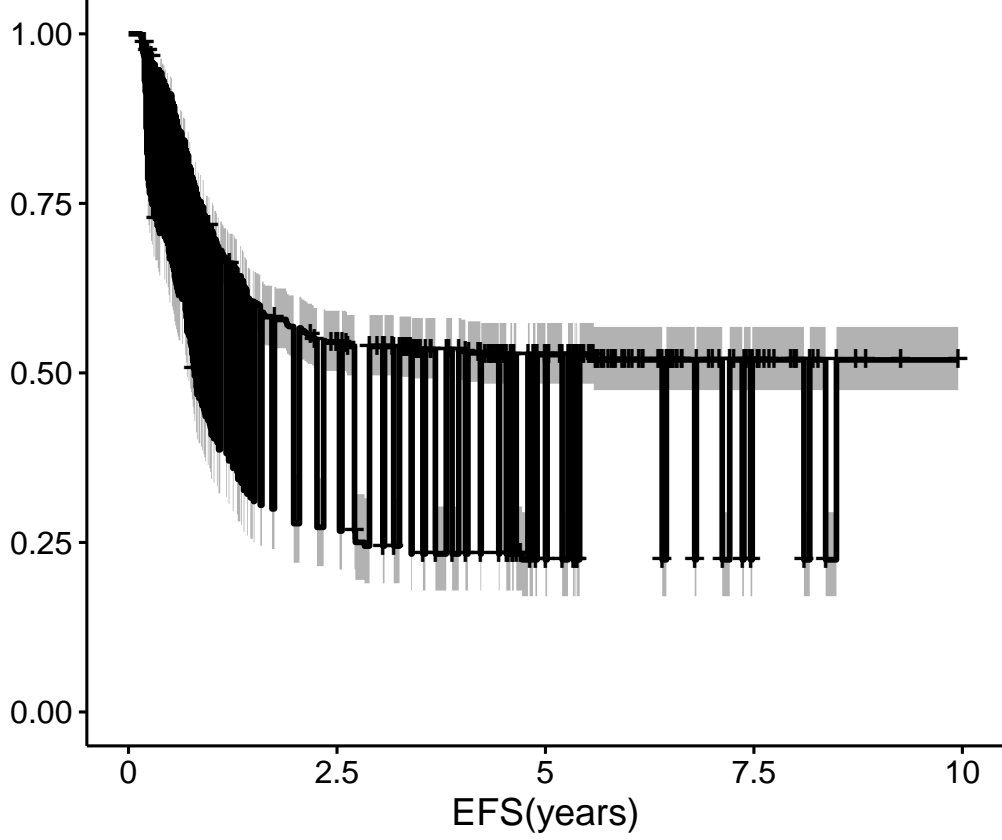

Number at risk

Strata

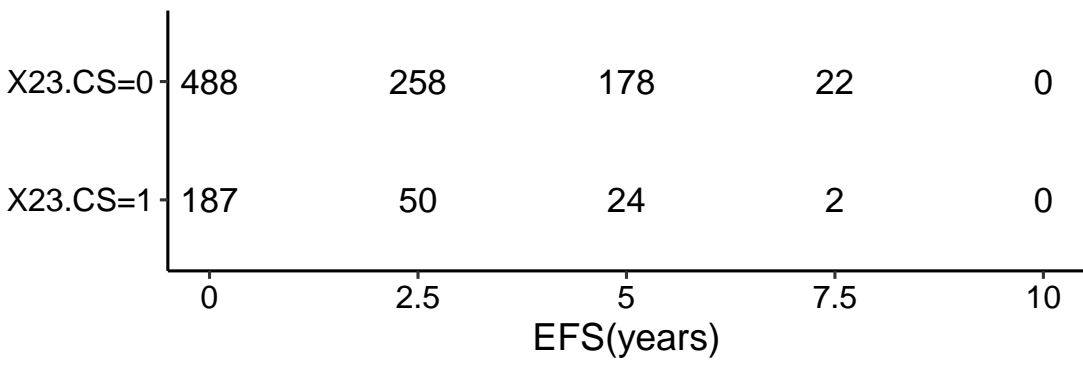

Number of censoring

n.censor

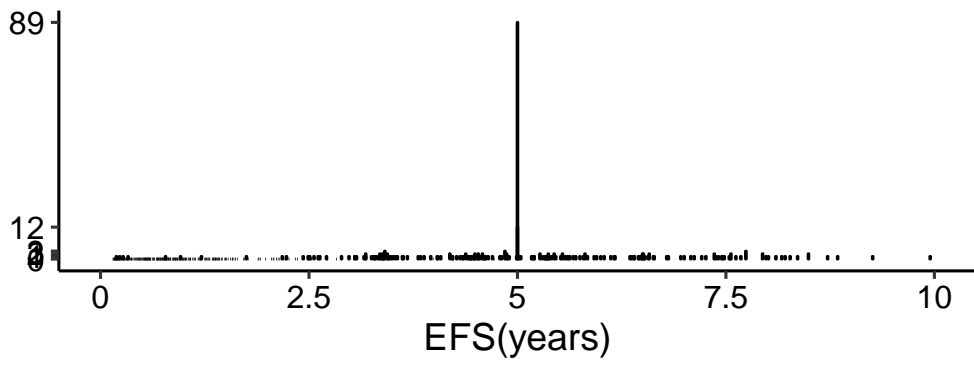

Supplement: S1 Code — (ZIP) [file pmed.1005088.s002.zip › S2 code/PROJ8_13_tbl1/PROJ8_13_tbl1_0.pdf]

## First Event

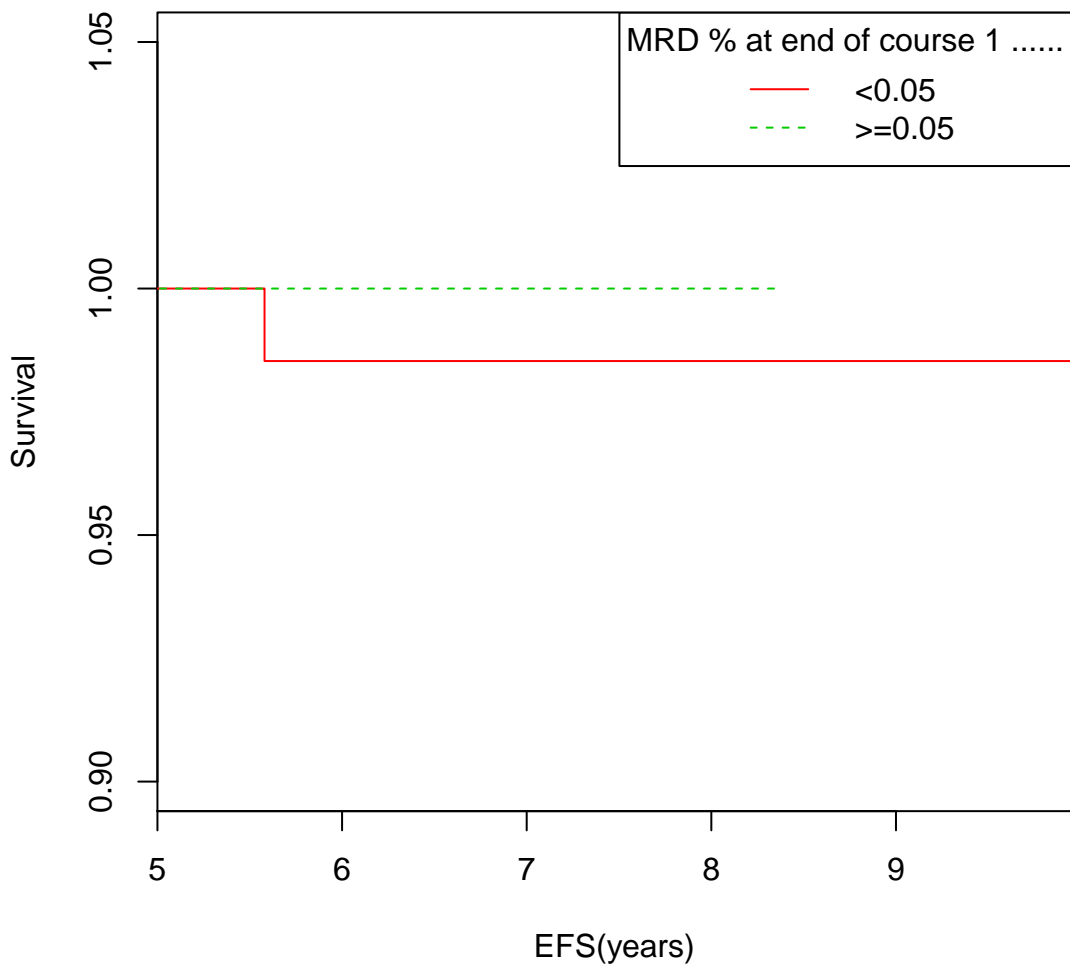

Supplement: S1 Code — (ZIP) [file pmed.1005088.s002.zip › S2 code/PROJ8_13_tbl1/PROJ8_13_tbl1_seg2_1.pdf]

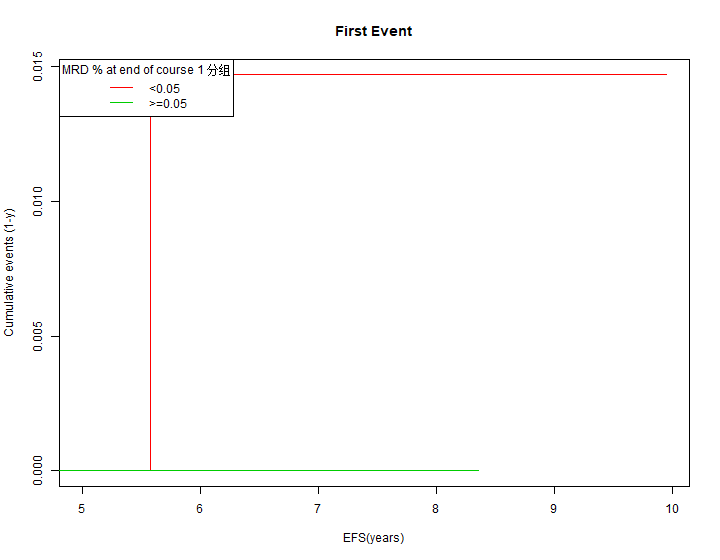

Supplement: S1 Code — (ZIP) [file pmed.1005088.s002.zip › S2 code/PROJ8_13_tbl1/PROJ8_13_tbl1_seg2_3.png]

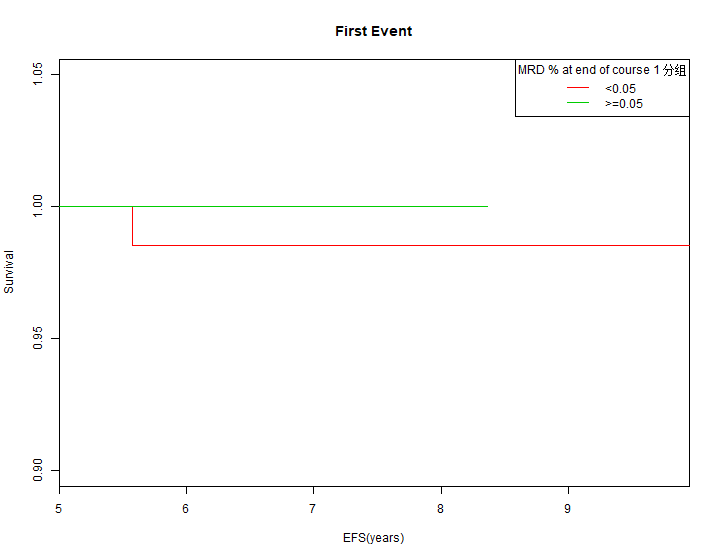

Supplement: S1 Code — (ZIP) [file pmed.1005088.s002.zip › S2 code/PROJ8_13_tbl1/PROJ8_13_tbl1_seg2_1.png]

## First Event

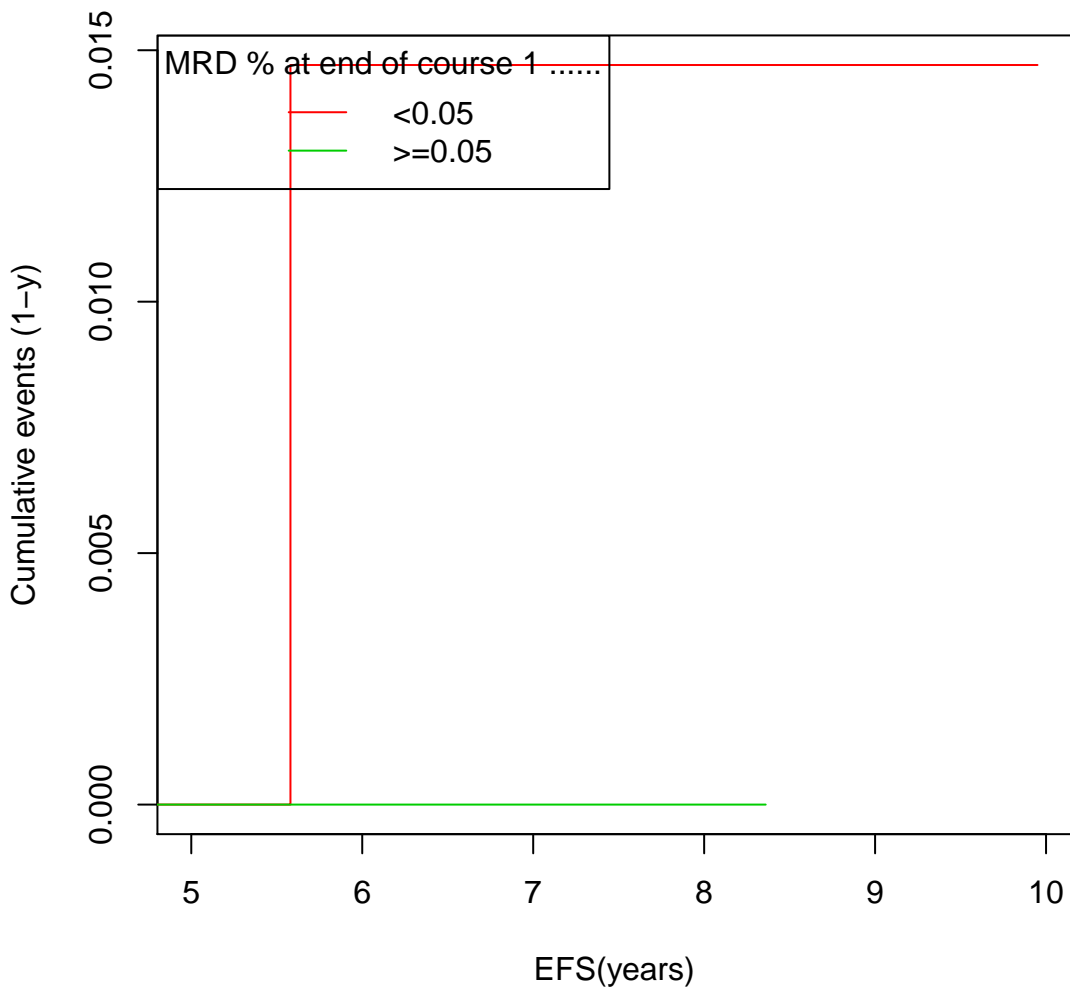

Supplement: S1 Code — (ZIP) [file pmed.1005088.s002.zip › S2 code/PROJ8_13_tbl1/PROJ8_13_tbl1_seg2_3.pdf]

## First Event

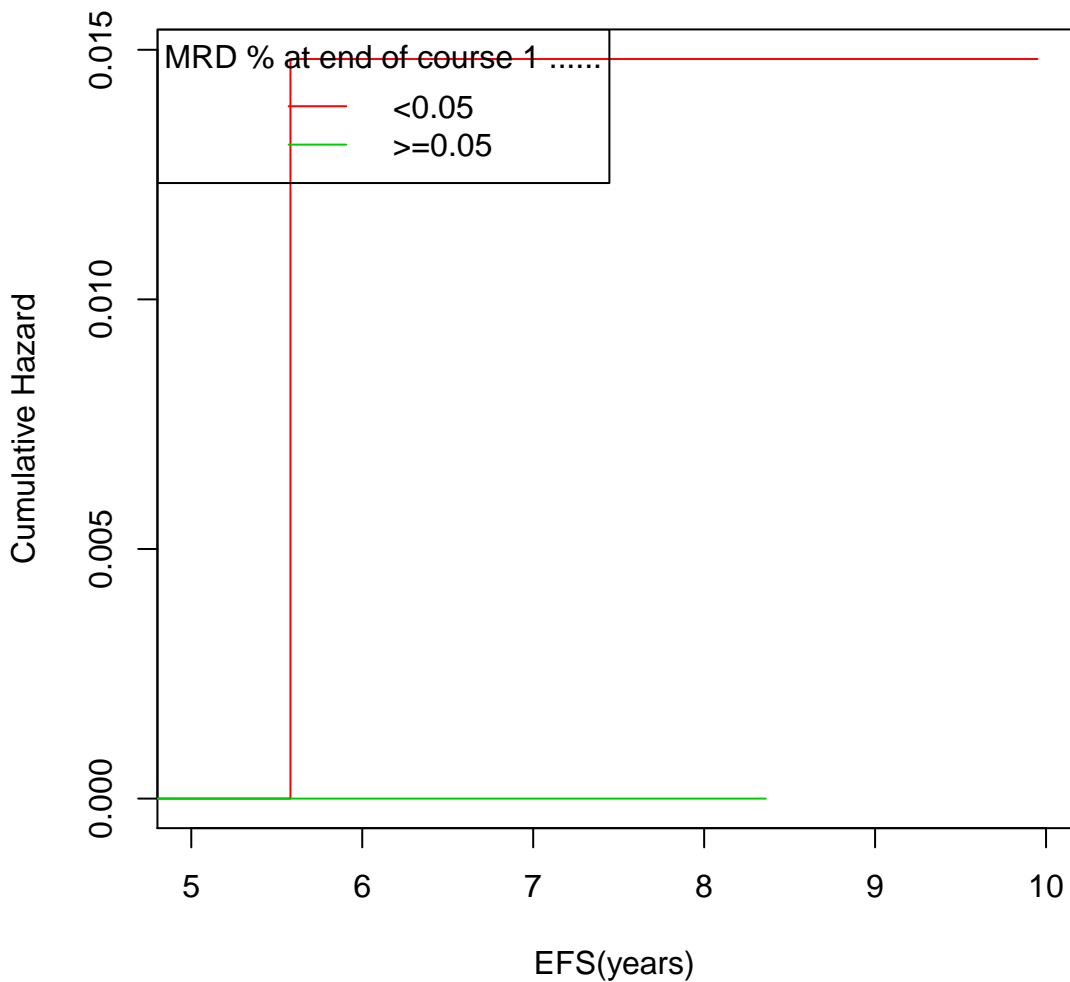

Supplement: S1 Code — (ZIP) [file pmed.1005088.s002.zip › S2 code/PROJ8_13_tbl1/PROJ8_13_tbl1_seg2_2.pdf]

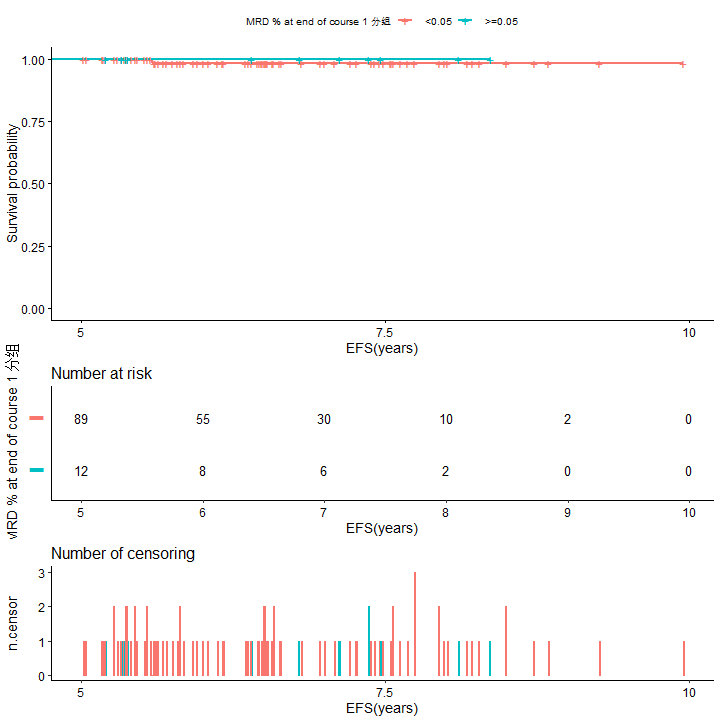

Supplement: S1 Code — (ZIP) [file pmed.1005088.s002.zip › S2 code/PROJ8_13_tbl1/PROJ8_13_tbl1_seg2_0.png]

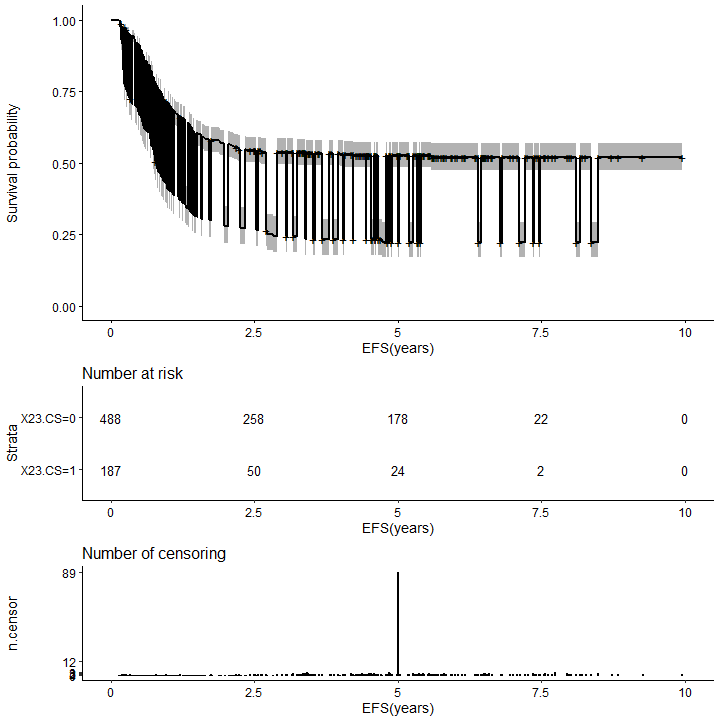

Supplement: S1 Code — (ZIP) [file pmed.1005088.s002.zip › S2 code/PROJ8_13_tbl1/PROJ8_13_tbl1_0.png]

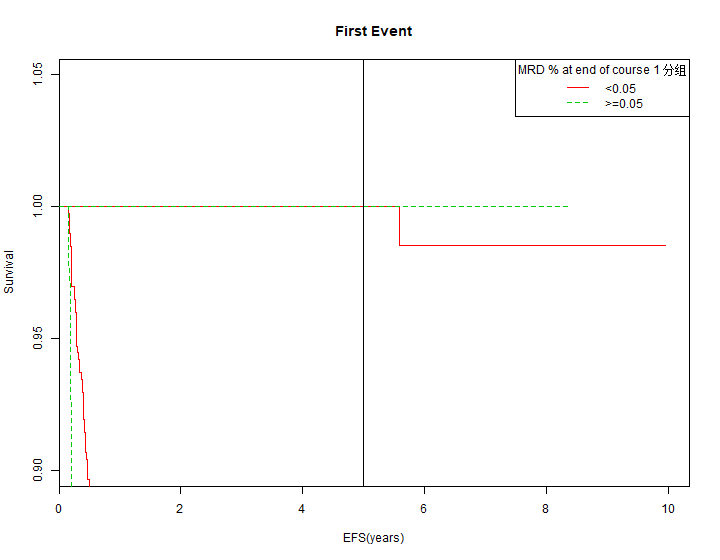

Supplement: S1 Code — (ZIP) [file pmed.1005088.s002.zip › S2 code/PROJ8_13_tbl1/PROJ8_13_tbl1.png]

## First Event

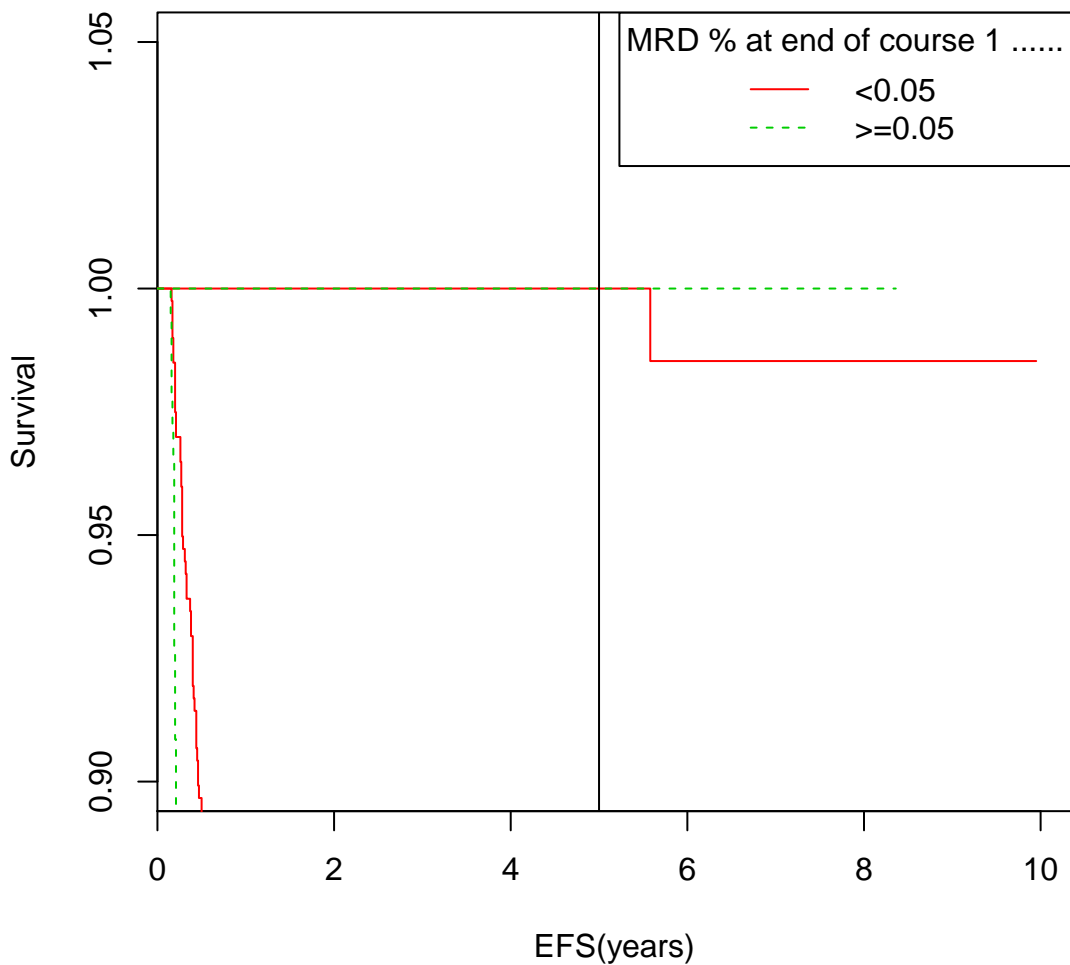

Supplement: S1 Code — (ZIP) [file pmed.1005088.s002.zip › S2 code/PROJ8_13_tbl1/PROJ8_13_tbl1.pdf]

MRD % at end of course 2 ..... + <0.05 + >=0.05

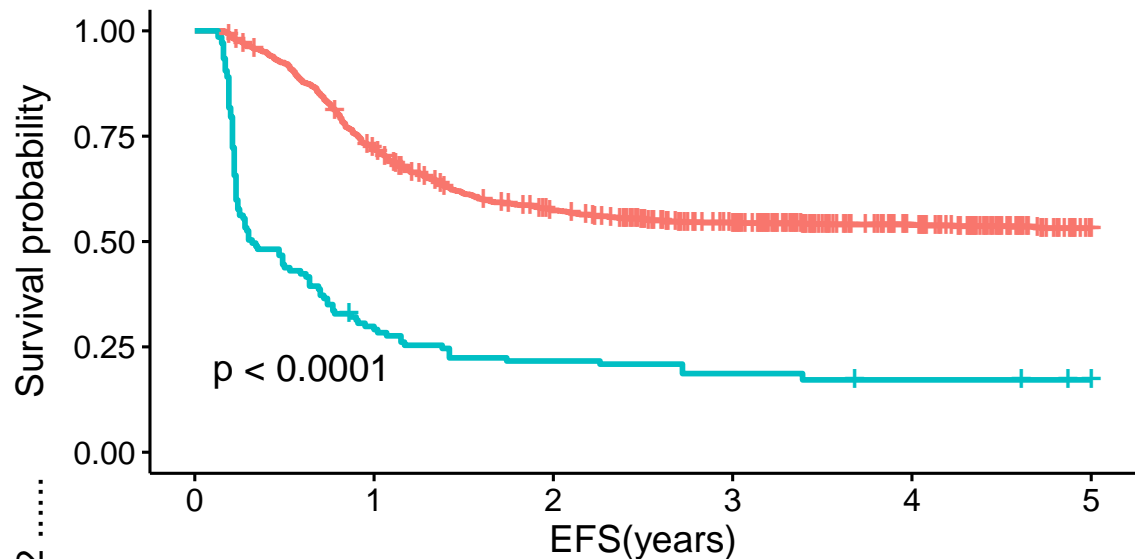

### Number at risk

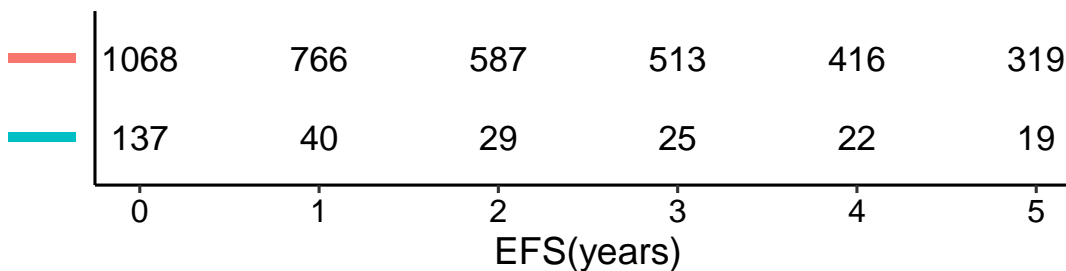

### Number of censoring

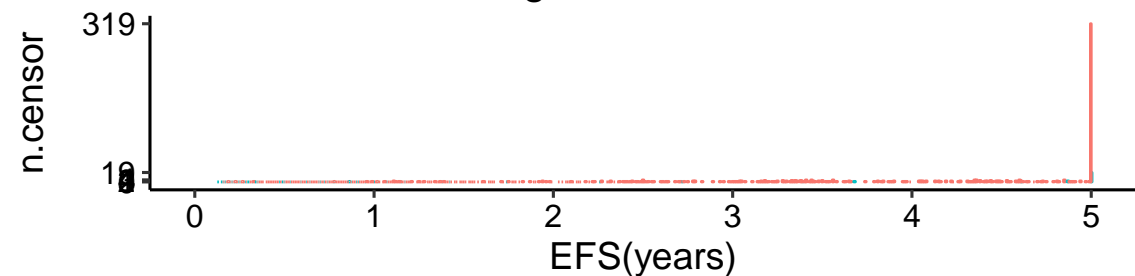

Supplement: S1 Code — (ZIP) [file pmed.1005088.s002.zip › S2 code/PROJ8_5_tbl/PROJ8_5_tbl_seg1_0.pdf]

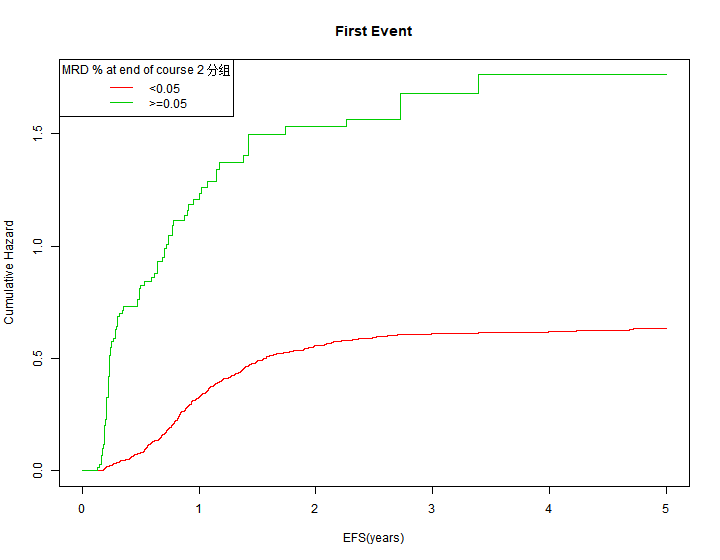

Supplement: S1 Code — (ZIP) [file pmed.1005088.s002.zip › S2 code/PROJ8_5_tbl/PROJ8_5_tbl_seg1_2.png]

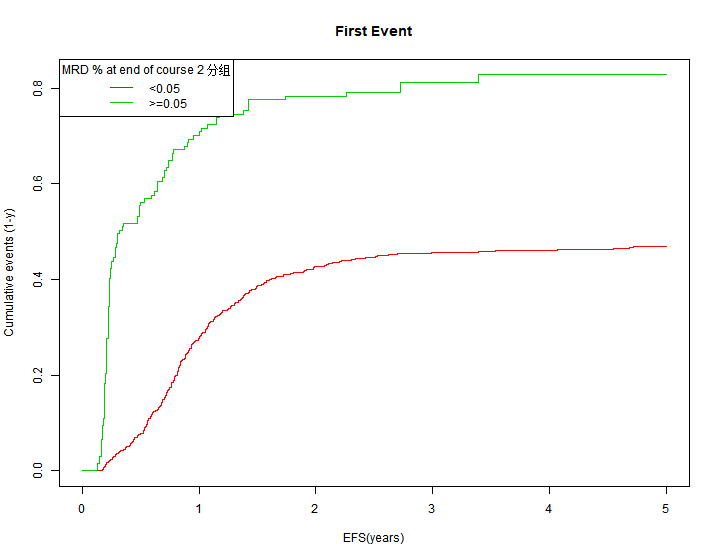

Supplement: S1 Code — (ZIP) [file pmed.1005088.s002.zip › S2 code/PROJ8_5_tbl/PROJ8_5_tbl_seg1_3.png]

## First Event

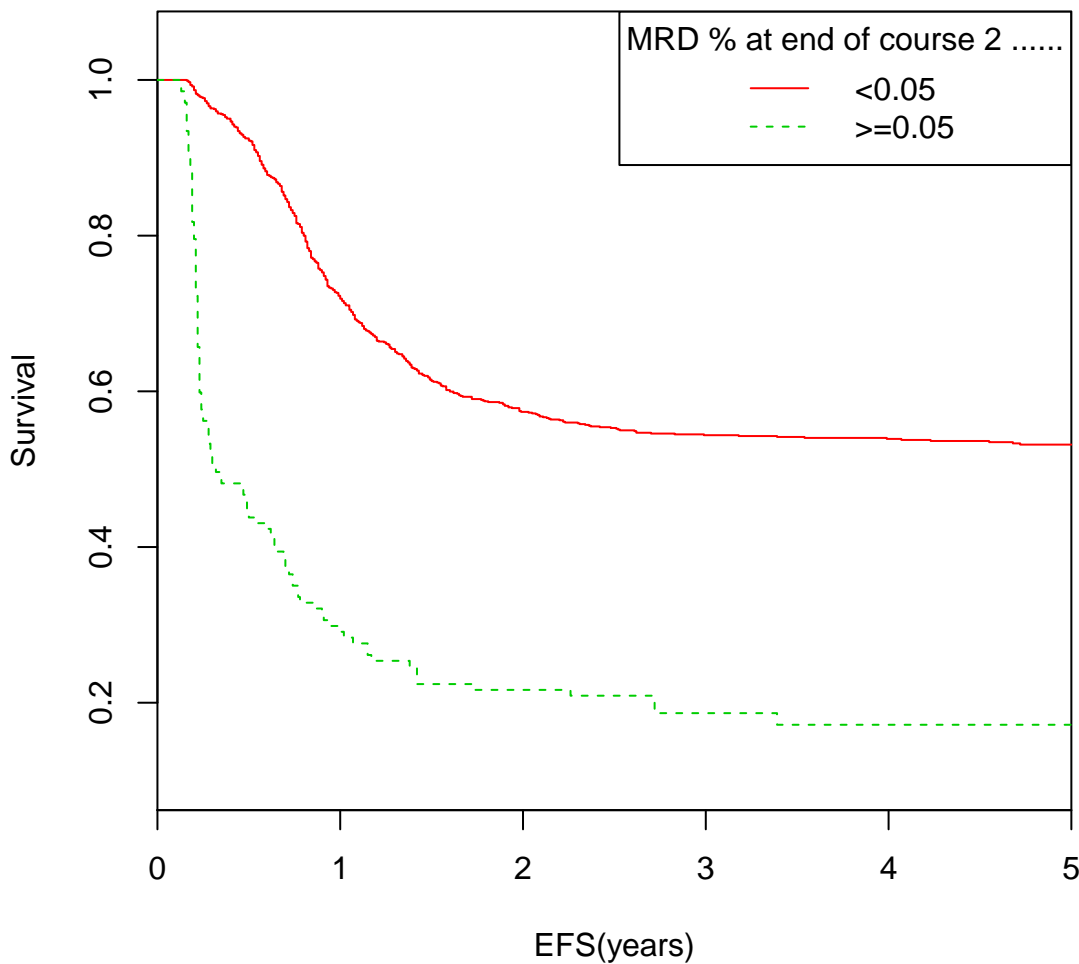

Supplement: S1 Code — (ZIP) [file pmed.1005088.s002.zip › S2 code/PROJ8_5_tbl/PROJ8_5_tbl_seg1_1.pdf]

## First Event

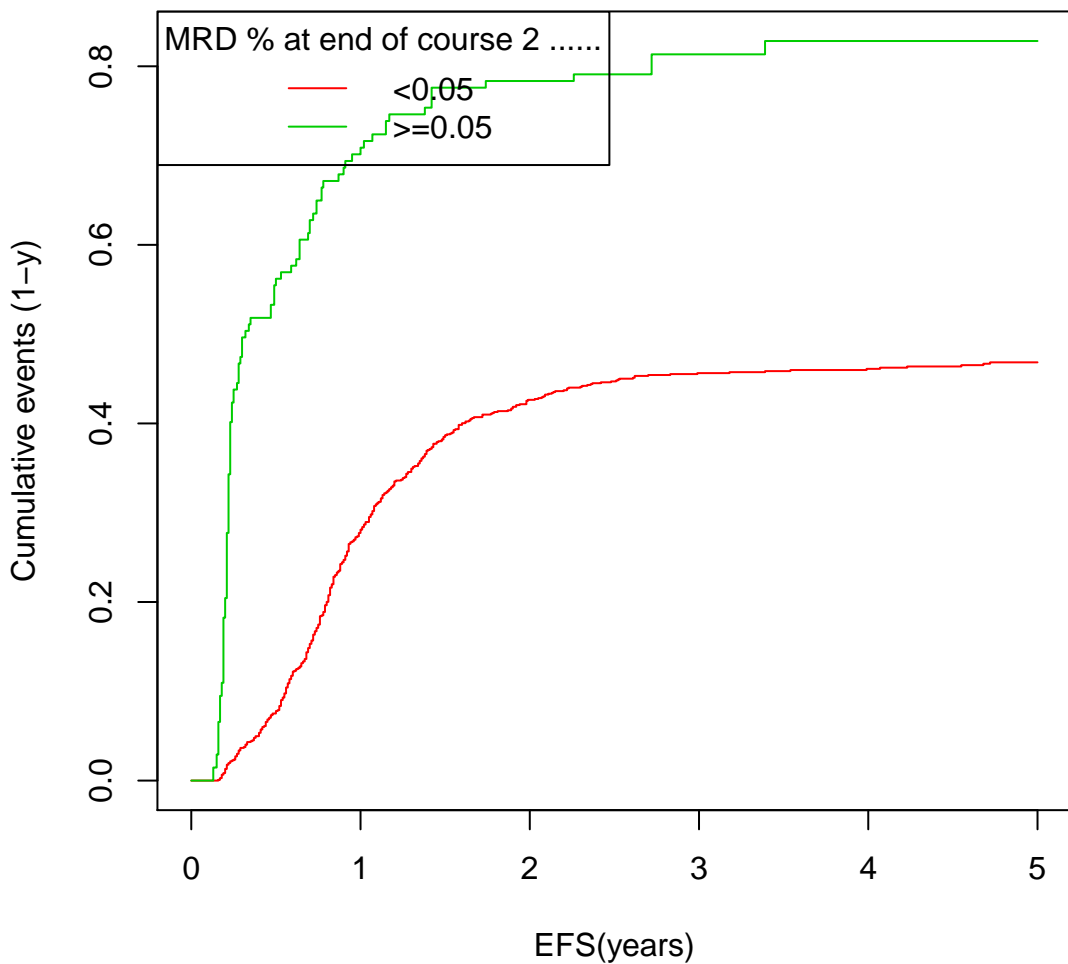

Supplement: S1 Code — (ZIP) [file pmed.1005088.s002.zip › S2 code/PROJ8_5_tbl/PROJ8_5_tbl_seg1_3.pdf]

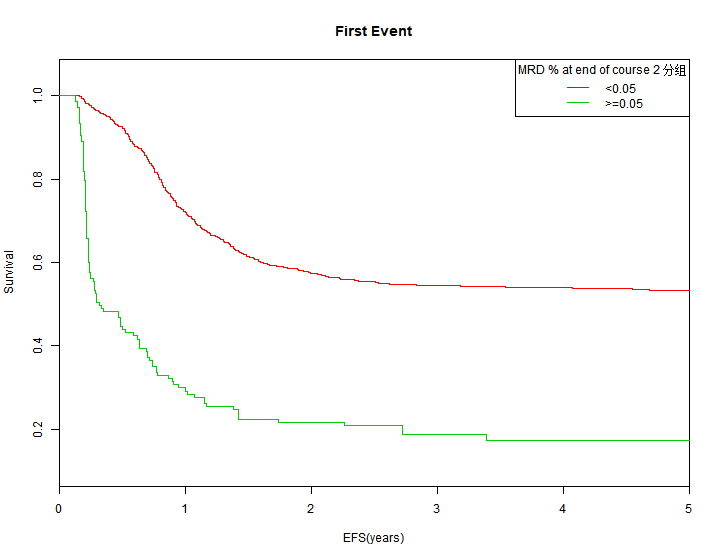

Supplement: S1 Code — (ZIP) [file pmed.1005088.s002.zip › S2 code/PROJ8_5_tbl/PROJ8_5_tbl_seg1_1.png]

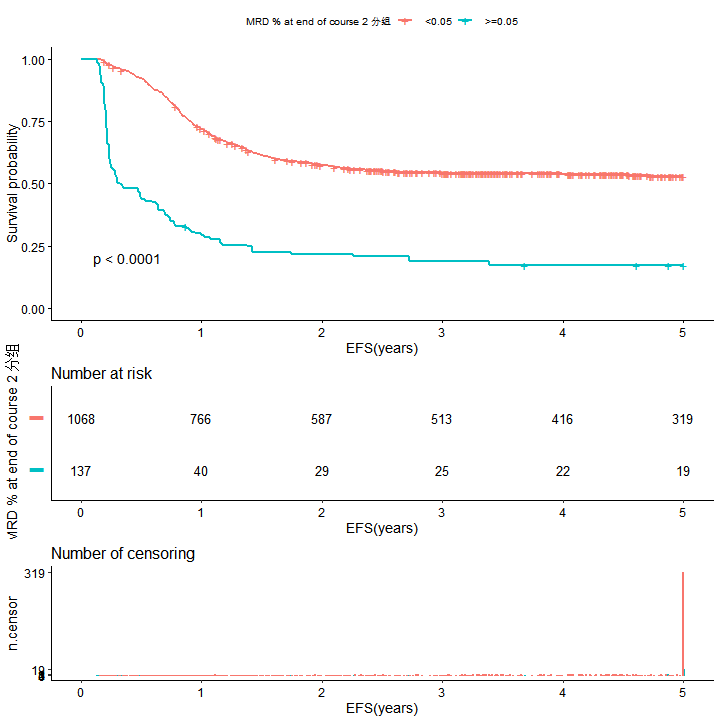

Supplement: S1 Code — (ZIP) [file pmed.1005088.s002.zip › S2 code/PROJ8_5_tbl/PROJ8_5_tbl_seg1_0.png]

## First Event

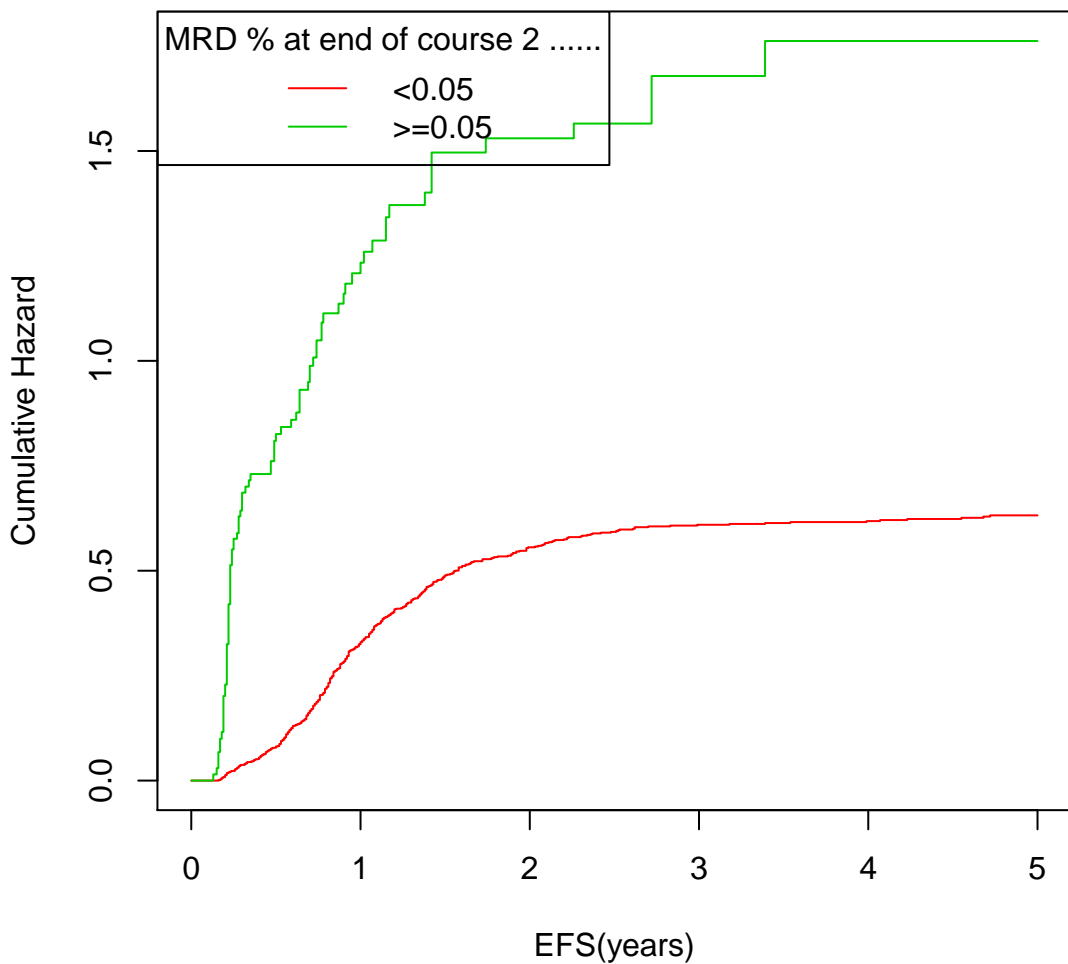

Supplement: S1 Code — (ZIP) [file pmed.1005088.s002.zip › S2 code/PROJ8_5_tbl/PROJ8_5_tbl_seg1_2.pdf]

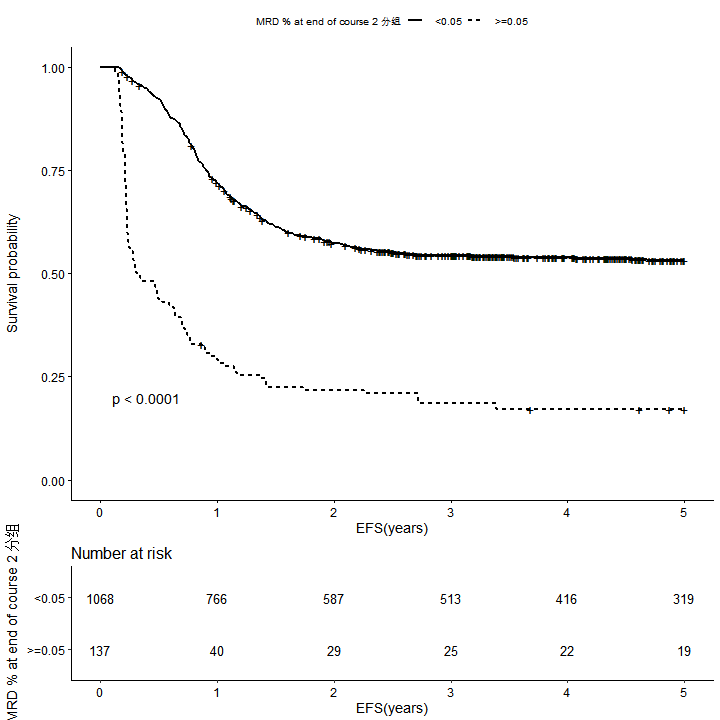

Supplement: S1 Code — (ZIP) [file pmed.1005088.s002.zip › S2 code/PROJ8_5_tbl/PROJ8_5_tbl_seg1_b.png]

MRD % at end of course 2 .....

MRD % at end of course 2 ..... — <0.05 - - - - - ≥0.05

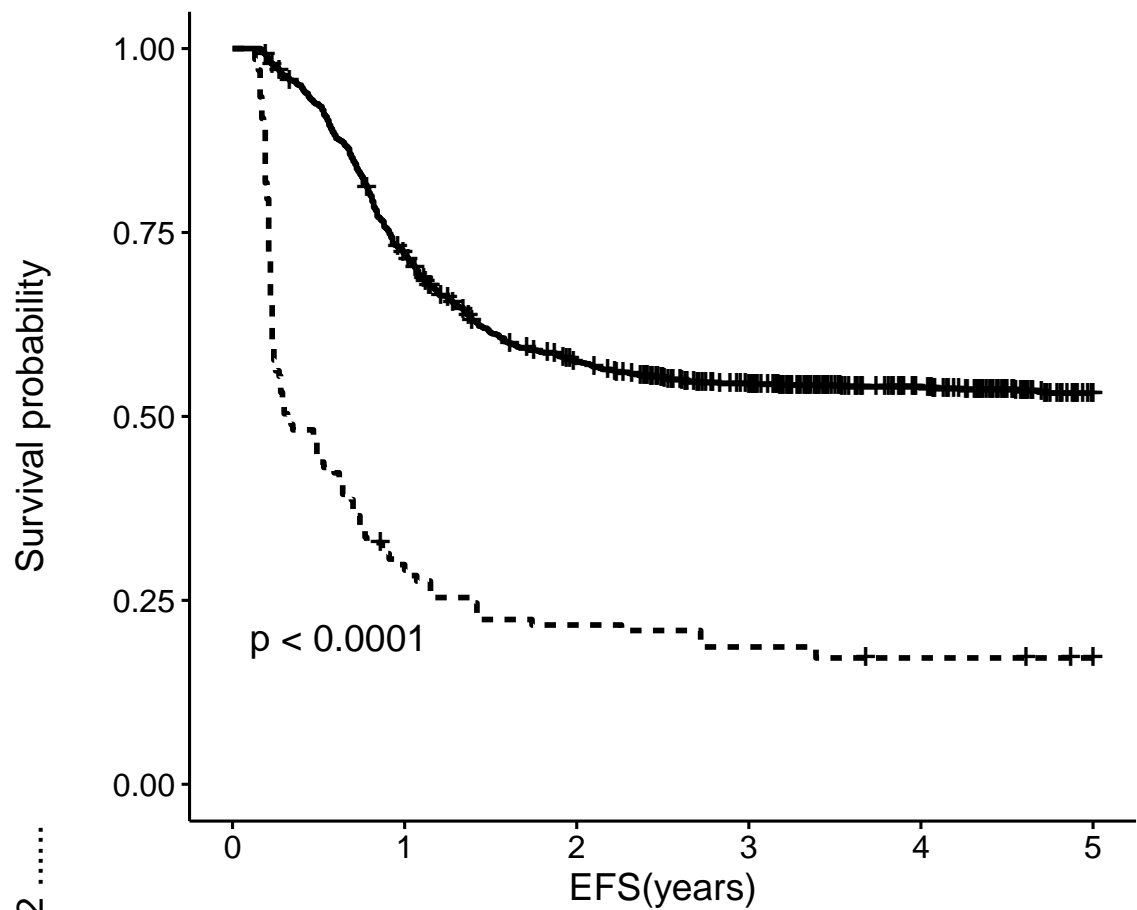

Number at risk

|       | 0    | 1   | 2   | 3   | 4   | 5   |
|-------|------|-----|-----|-----|-----|-----|
| <0.05 | 1068 | 766 | 587 | 513 | 416 | 319 |
| ≥0.05 | 137  | 40  | 29  | 25  | 22  | 19  |

EFS(years)

Supplement: S1 Code — (ZIP) [file pmed.1005088.s002.zip › S2 code/PROJ8_5_tbl/PROJ8_5_tbl_seg1_b.pdf]

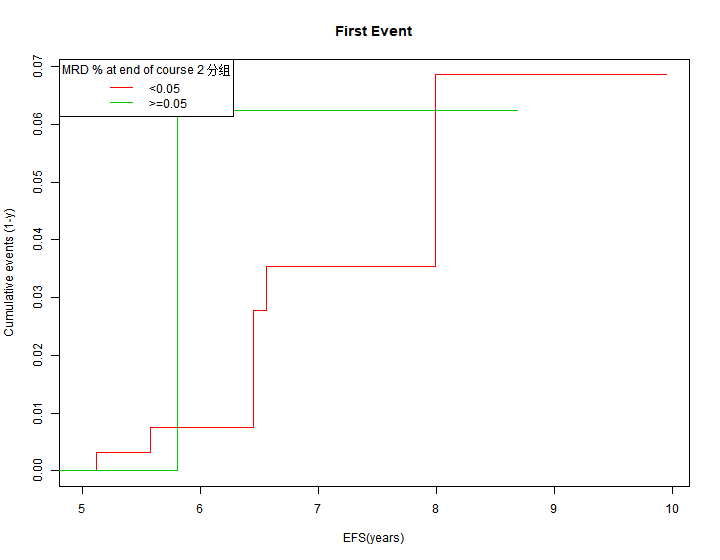

Supplement: S1 Code — (ZIP) [file pmed.1005088.s002.zip › S2 code/PROJ8_5_tbl/PROJ8_5_tbl_seg2_3.png]

## First Event

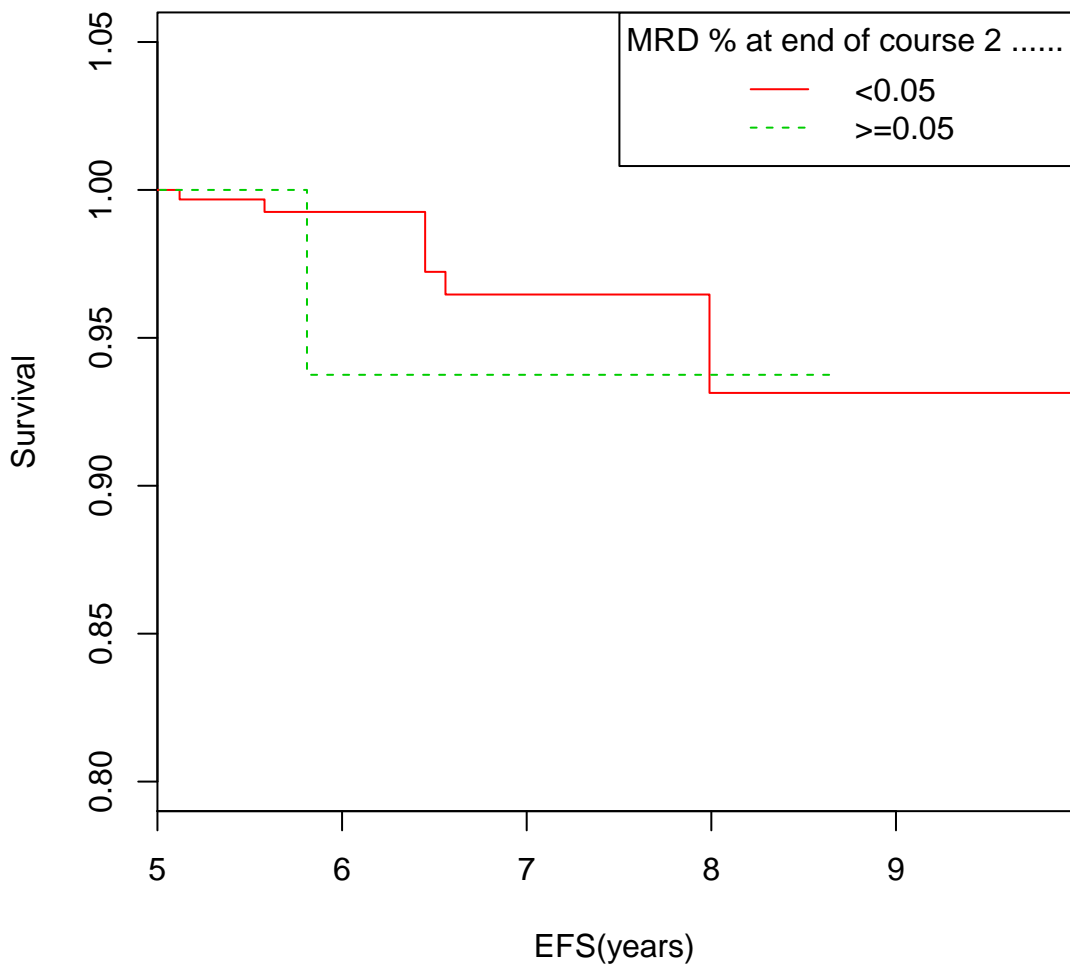

Supplement: S1 Code — (ZIP) [file pmed.1005088.s002.zip › S2 code/PROJ8_5_tbl/PROJ8_5_tbl_seg2_1.pdf]

MRD % at end of course 2 ..... + <0.05 + ≥0.05

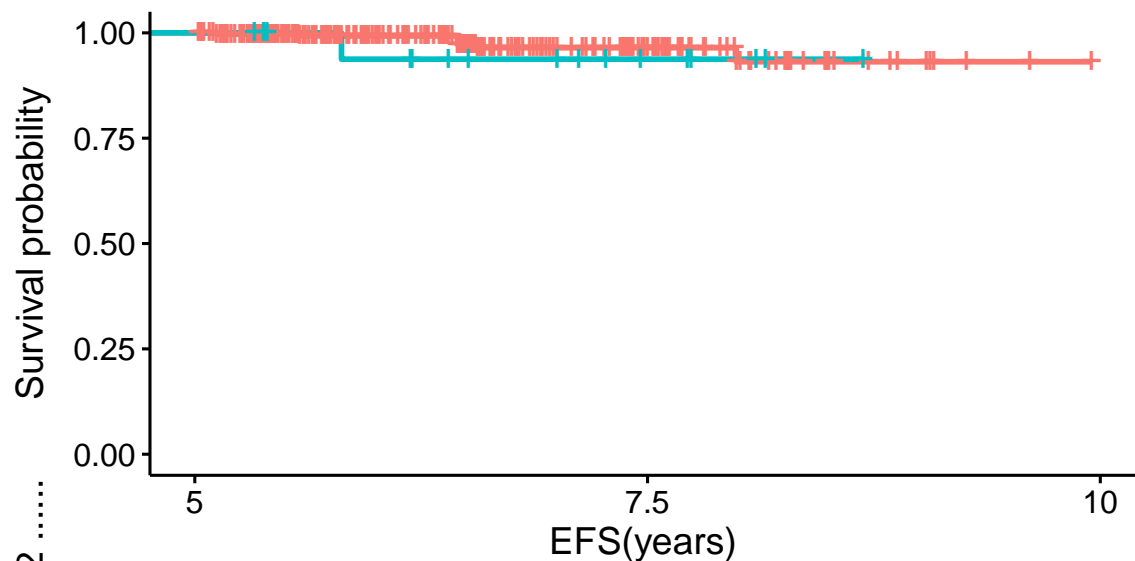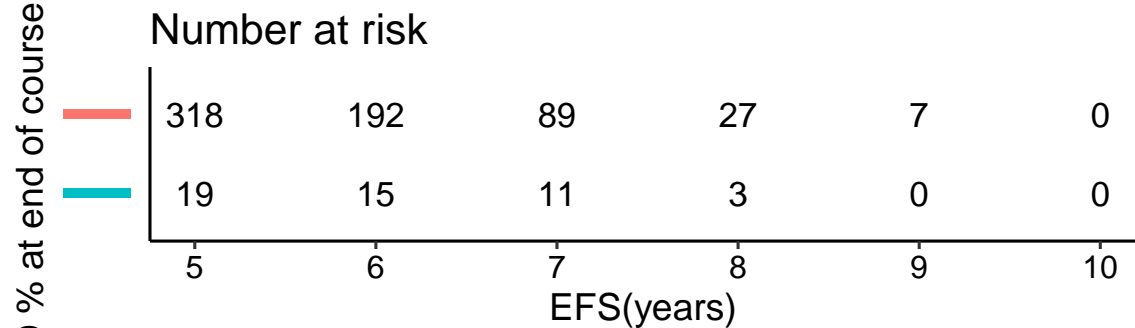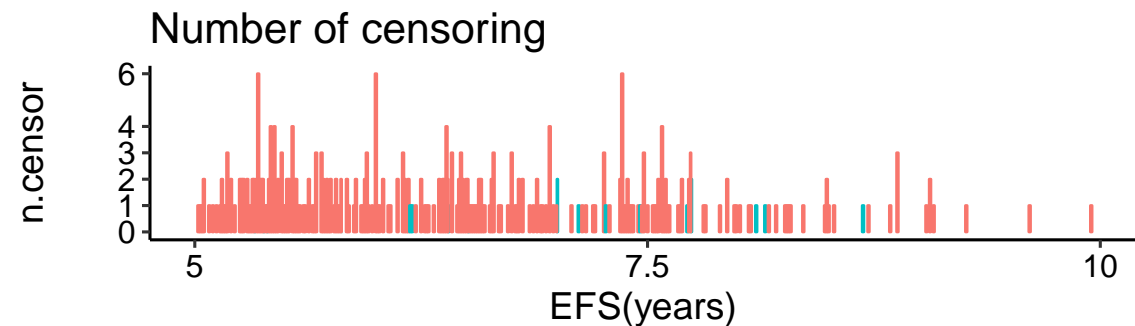

Supplement: S1 Code — (ZIP) [file pmed.1005088.s002.zip › S2 code/PROJ8_5_tbl/PROJ8_5_tbl_seg2_0.pdf]

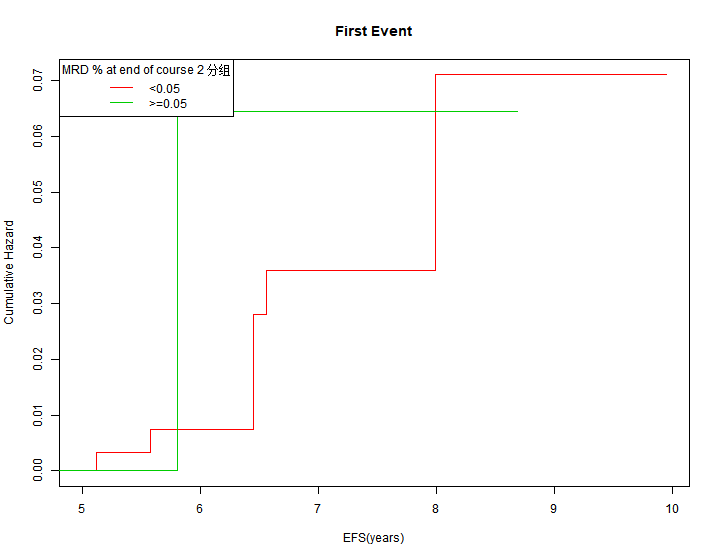

Supplement: S1 Code — (ZIP) [file pmed.1005088.s002.zip › S2 code/PROJ8_5_tbl/PROJ8_5_tbl_seg2_2.png]

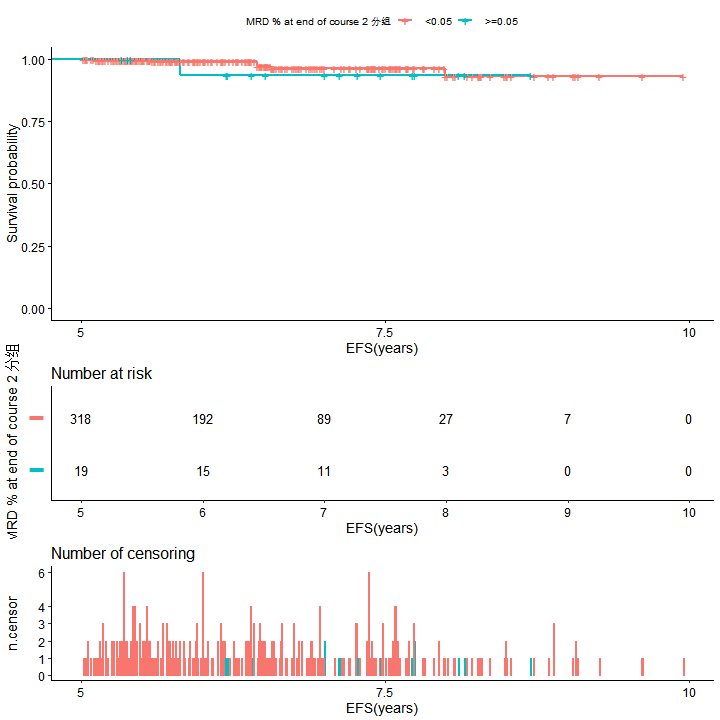

Supplement: S1 Code — (ZIP) [file pmed.1005088.s002.zip › S2 code/PROJ8_5_tbl/PROJ8_5_tbl_seg2_0.png]

## First Event

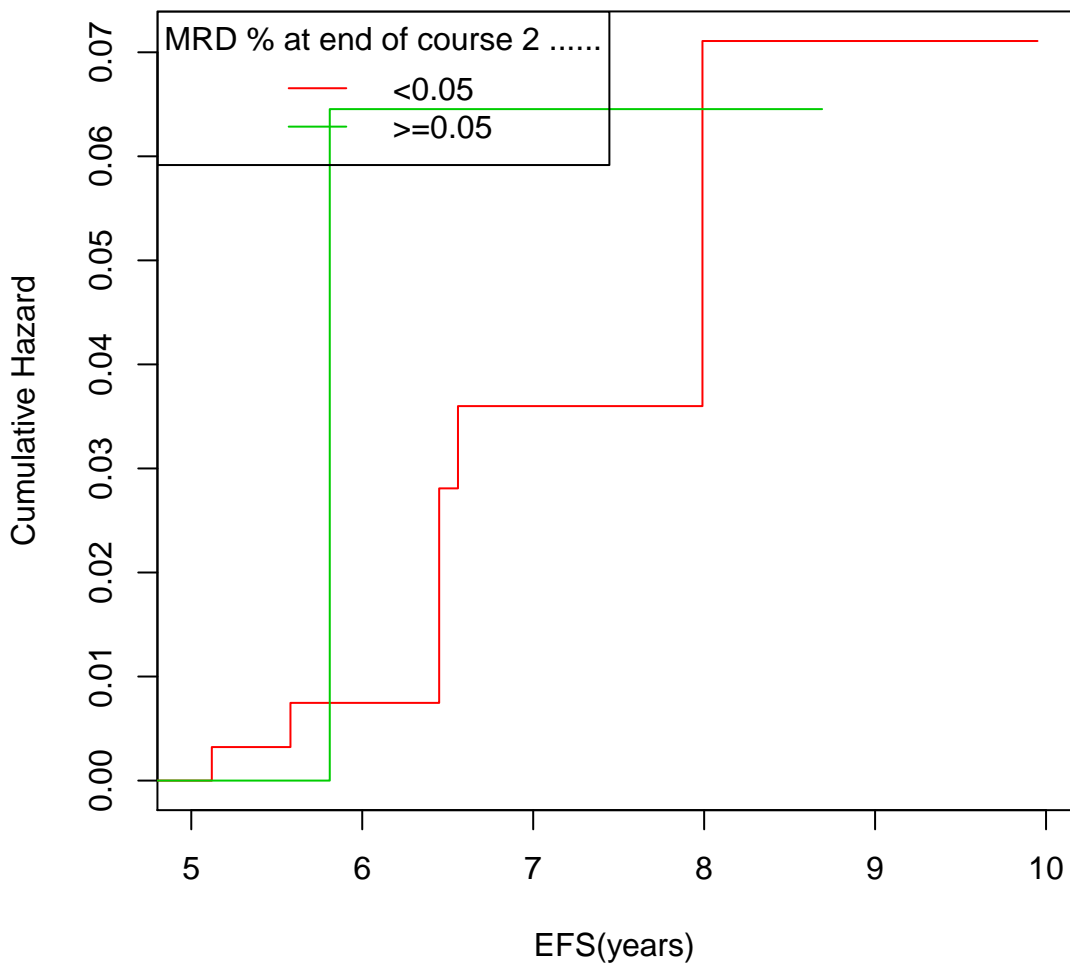

Supplement: S1 Code — (ZIP) [file pmed.1005088.s002.zip › S2 code/PROJ8_5_tbl/PROJ8_5_tbl_seg2_2.pdf]

## First Event

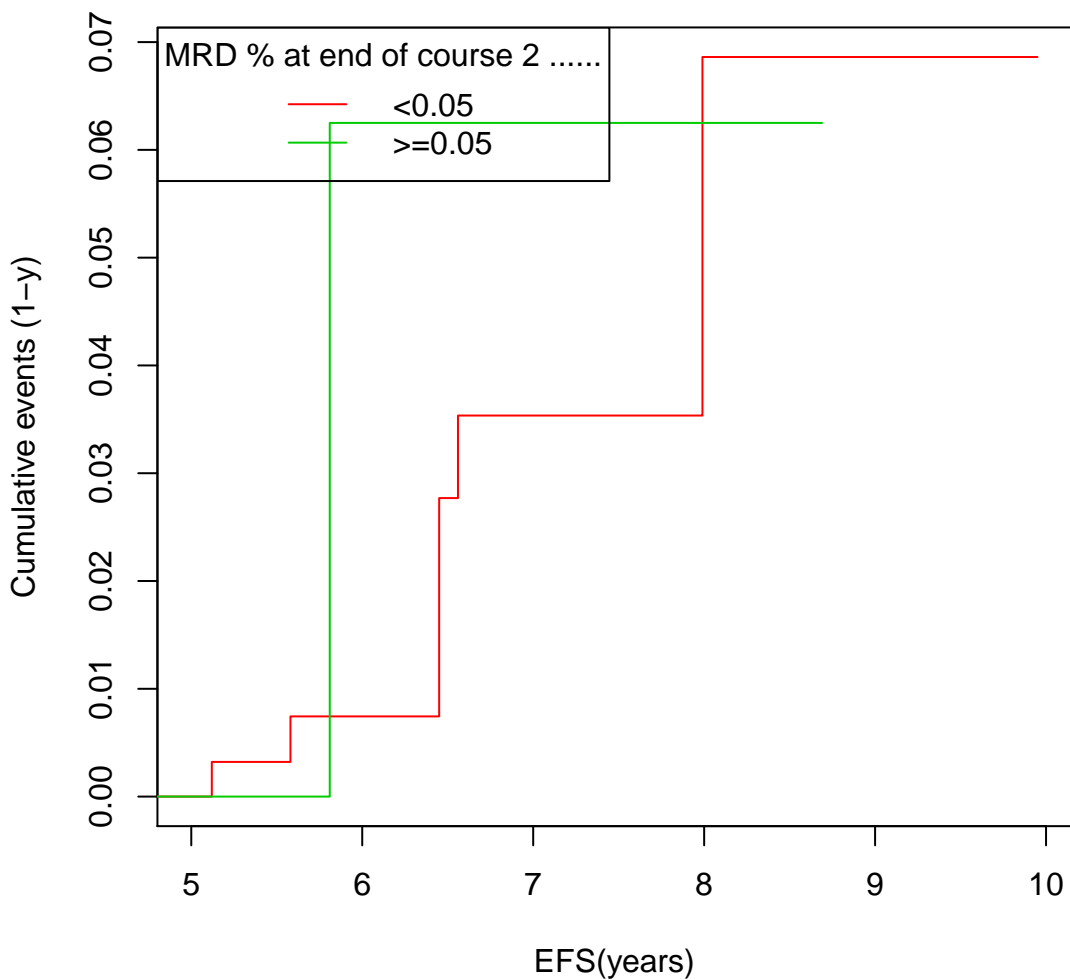

Supplement: S1 Code — (ZIP) [file pmed.1005088.s002.zip › S2 code/PROJ8_5_tbl/PROJ8_5_tbl_seg2_3.pdf]

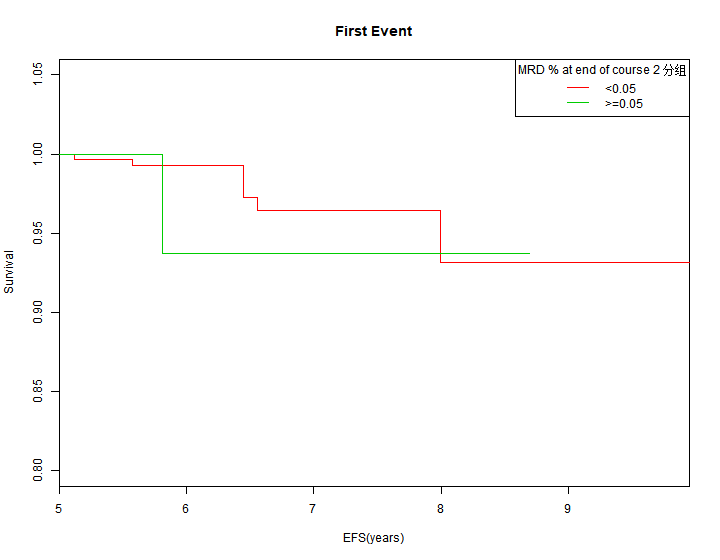

Supplement: S1 Code — (ZIP) [file pmed.1005088.s002.zip › S2 code/PROJ8_5_tbl/PROJ8_5_tbl_seg2_1.png]

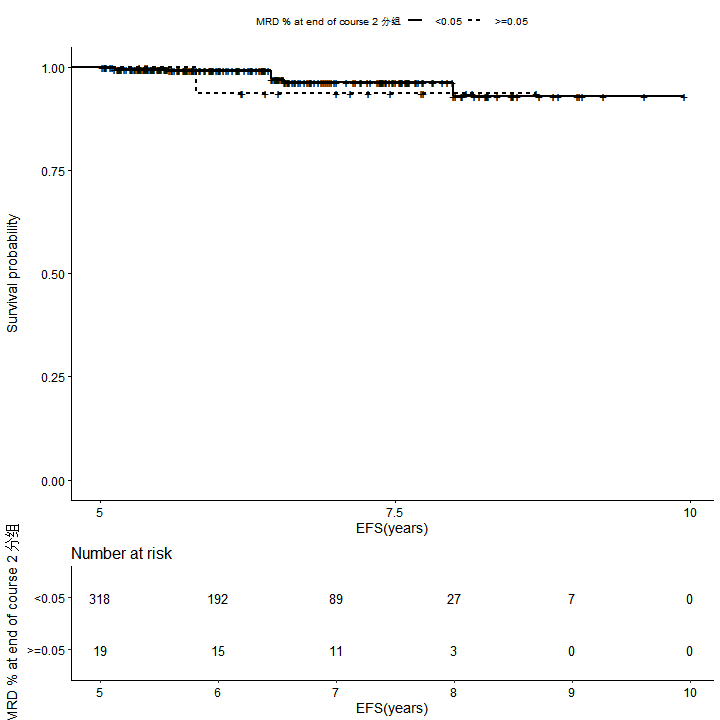

Supplement: S1 Code — (ZIP) [file pmed.1005088.s002.zip › S2 code/PROJ8_5_tbl/PROJ8_5_tbl_seg2_b.png]

Survival probability

MRD % at end of course 2 ..... — <0.05 - - - - - ≥0.05

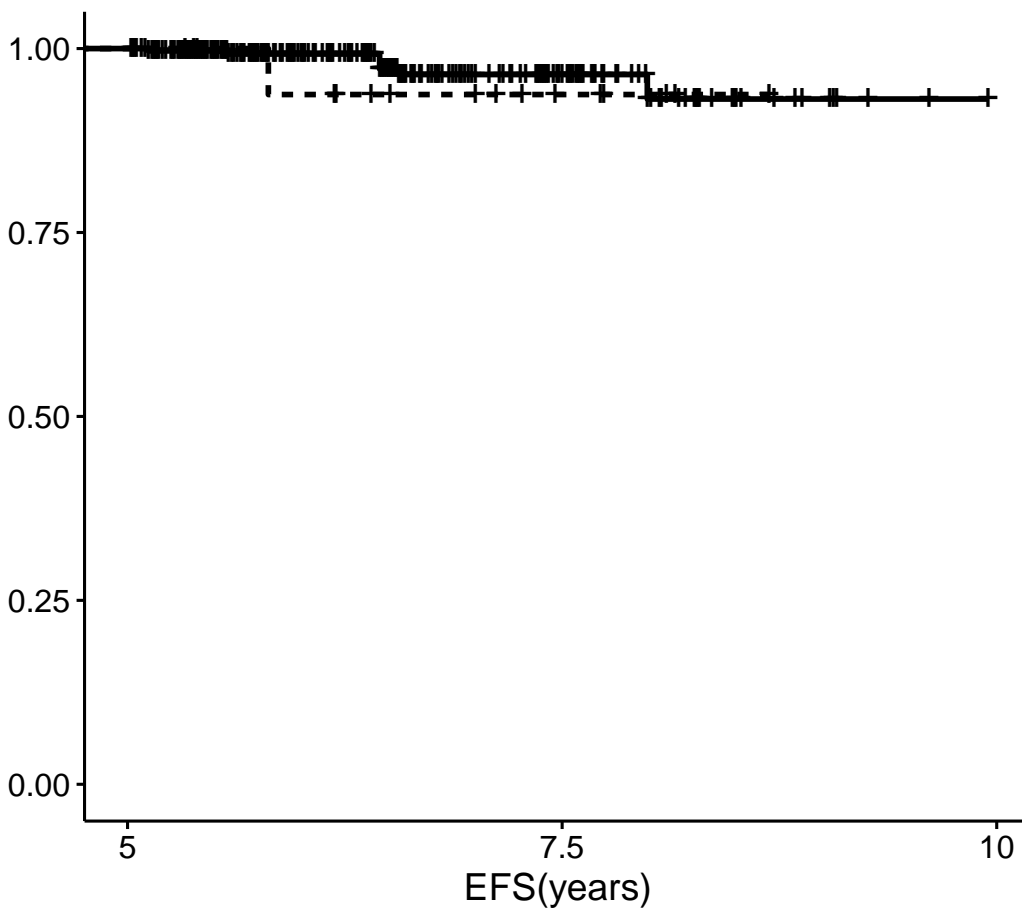

MRD % at end of course 2 .....

Number at risk

|       |            |     |    |    |   |    |
|-------|------------|-----|----|----|---|----|
| <0.05 | 318        | 192 | 89 | 27 | 7 | 0  |
| ≥0.05 | 19         | 15  | 11 | 3  | 0 | 0  |
|       | 5          | 6   | 7  | 8  | 9 | 10 |
|       | EFS(years) |     |    |    |   |    |

Supplement: S1 Code — (ZIP) [file pmed.1005088.s002.zip › S2 code/PROJ8_5_tbl/PROJ8_5_tbl_seg2_b.pdf]

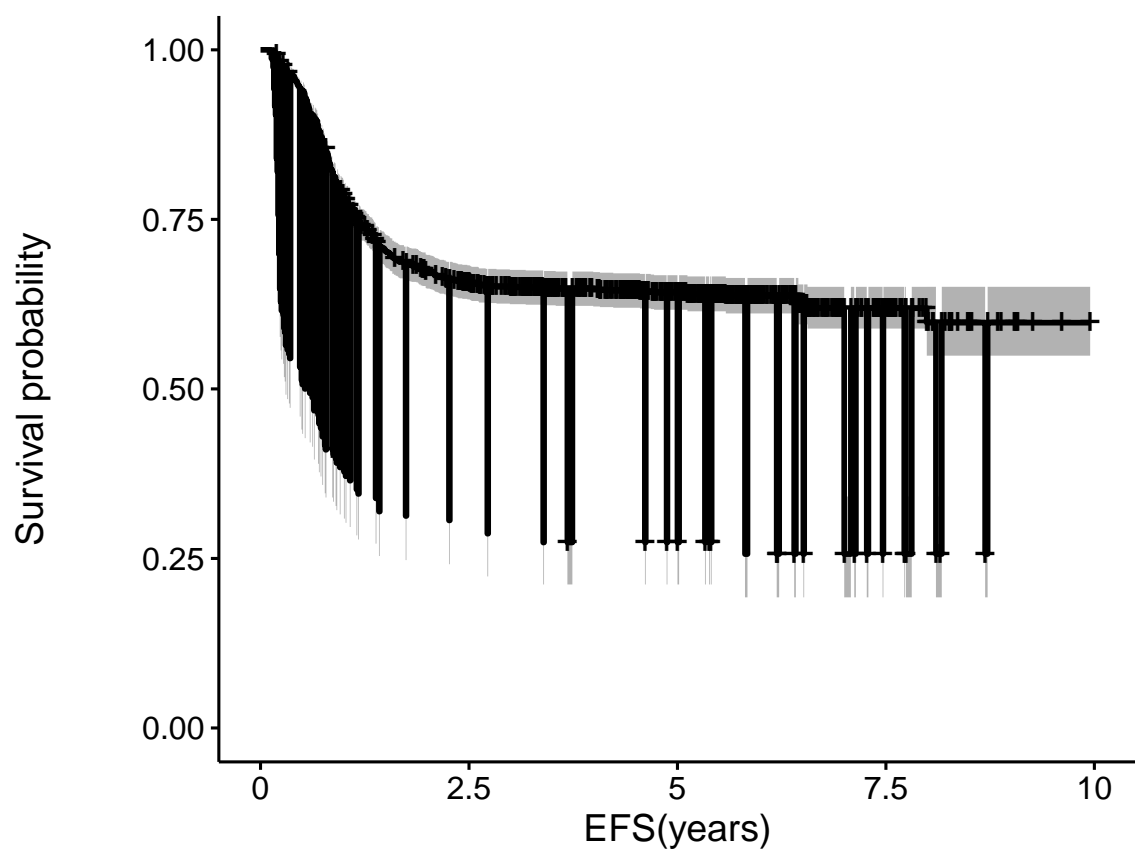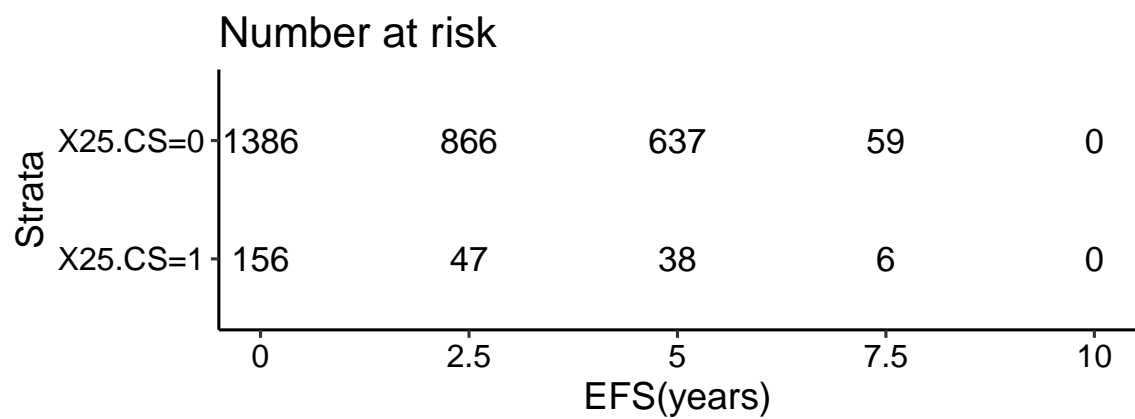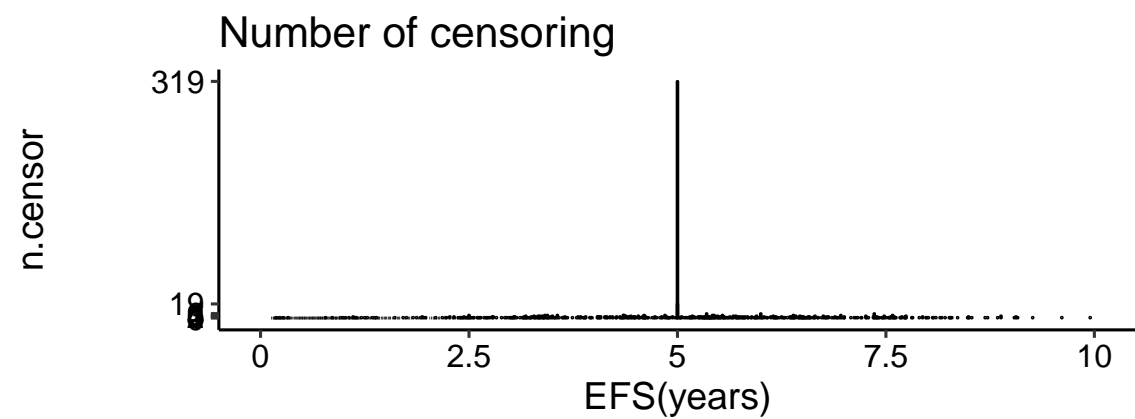

Supplement: S1 Code — (ZIP) [file pmed.1005088.s002.zip › S2 code/PROJ8_5_tbl/PROJ8_5_tbl_0.pdf]

## First Event

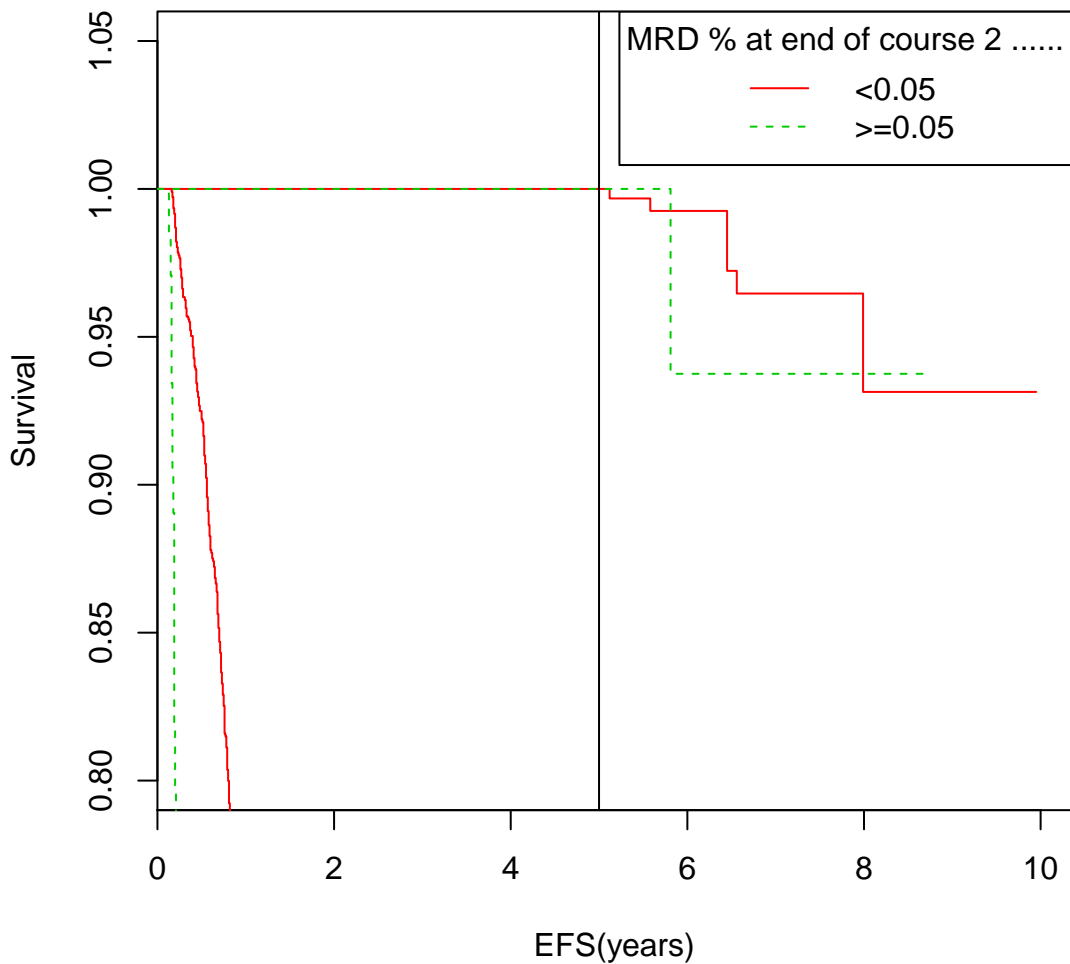

Supplement: S1 Code — (ZIP) [file pmed.1005088.s002.zip › S2 code/PROJ8_5_tbl/PROJ8_5_tbl.pdf]

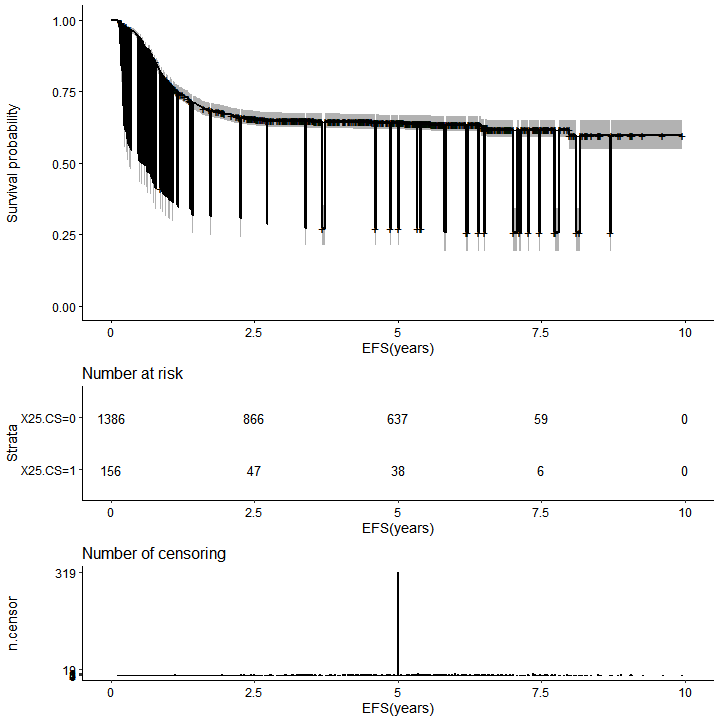

Supplement: S1 Code — (ZIP) [file pmed.1005088.s002.zip › S2 code/PROJ8_5_tbl/PROJ8_5_tbl_0.png]

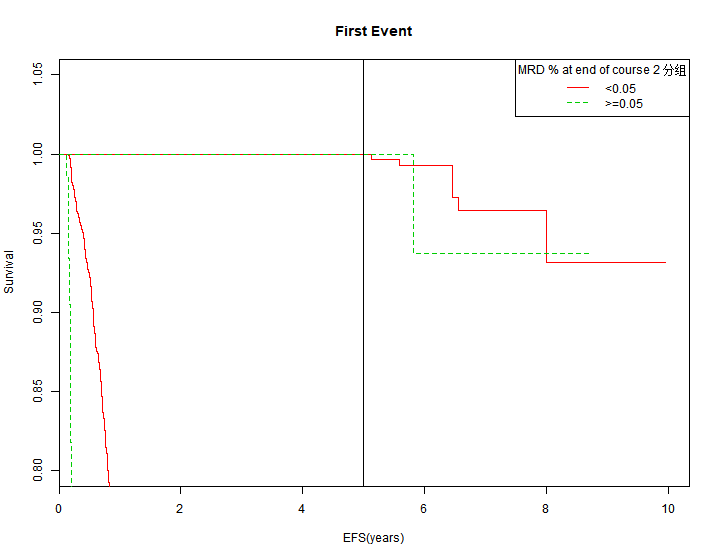

Supplement: S1 Code — (ZIP) [file pmed.1005088.s002.zip › S2 code/PROJ8_5_tbl/PROJ8_5_tbl.png]

MRD % at end of course 1 ..... + <0.05 + >=0.05

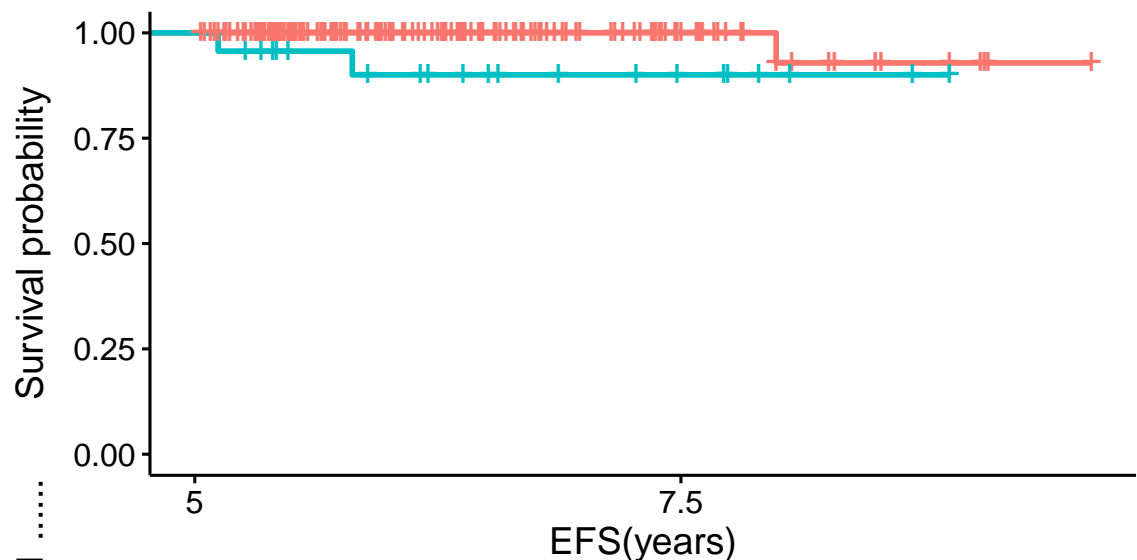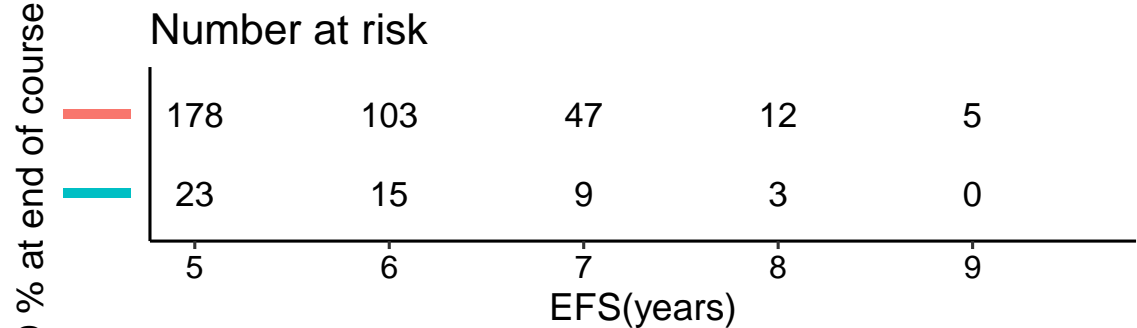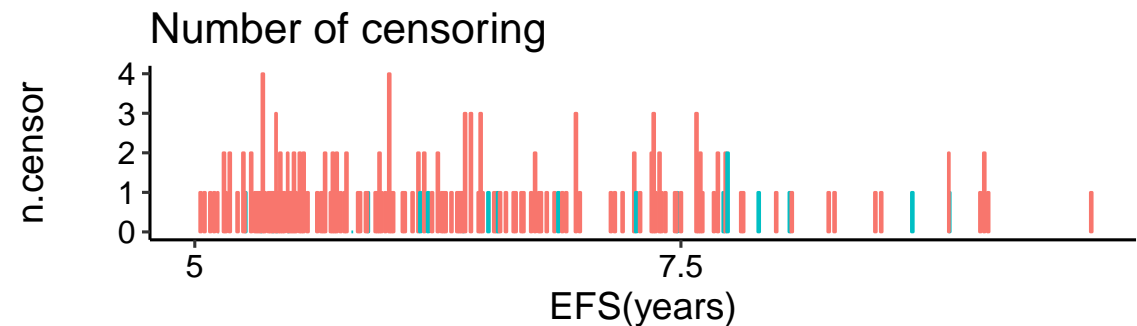

Supplement: S1 Code — (ZIP) [file pmed.1005088.s002.zip › S2 code/PROJ8_12_tbl1/PROJ8_12_tbl1_seg2_0.pdf]

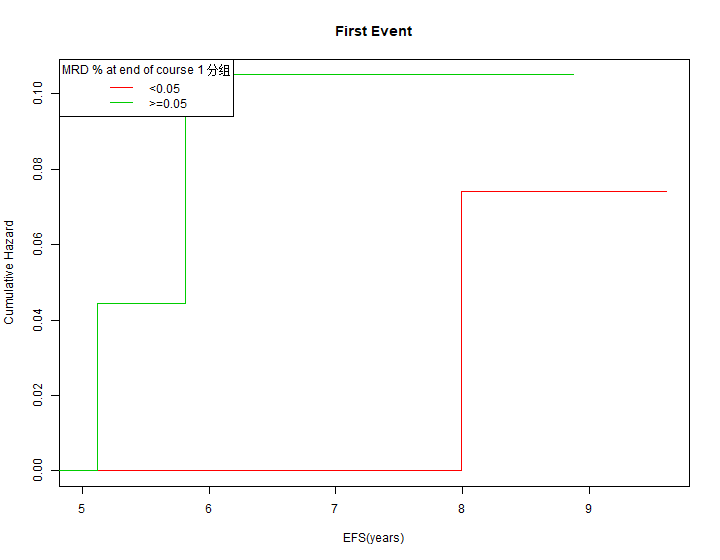

Supplement: S1 Code — (ZIP) [file pmed.1005088.s002.zip › S2 code/PROJ8_12_tbl1/PROJ8_12_tbl1_seg2_2.png]

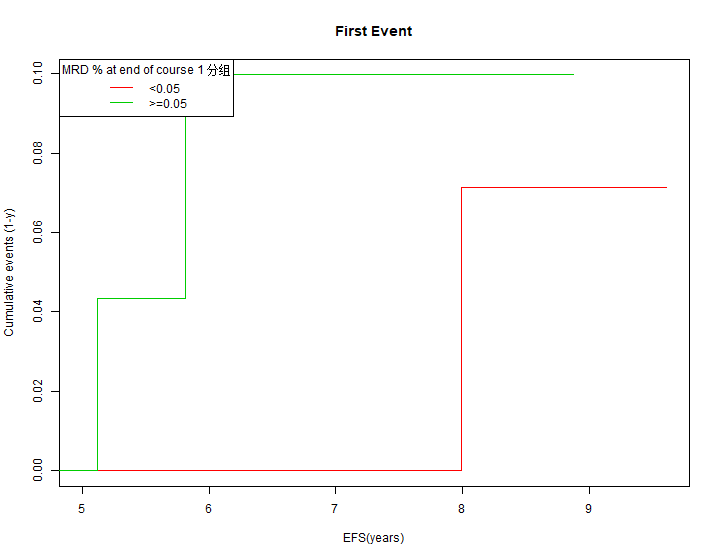

Supplement: S1 Code — (ZIP) [file pmed.1005088.s002.zip › S2 code/PROJ8_12_tbl1/PROJ8_12_tbl1_seg2_3.png]

## First Event

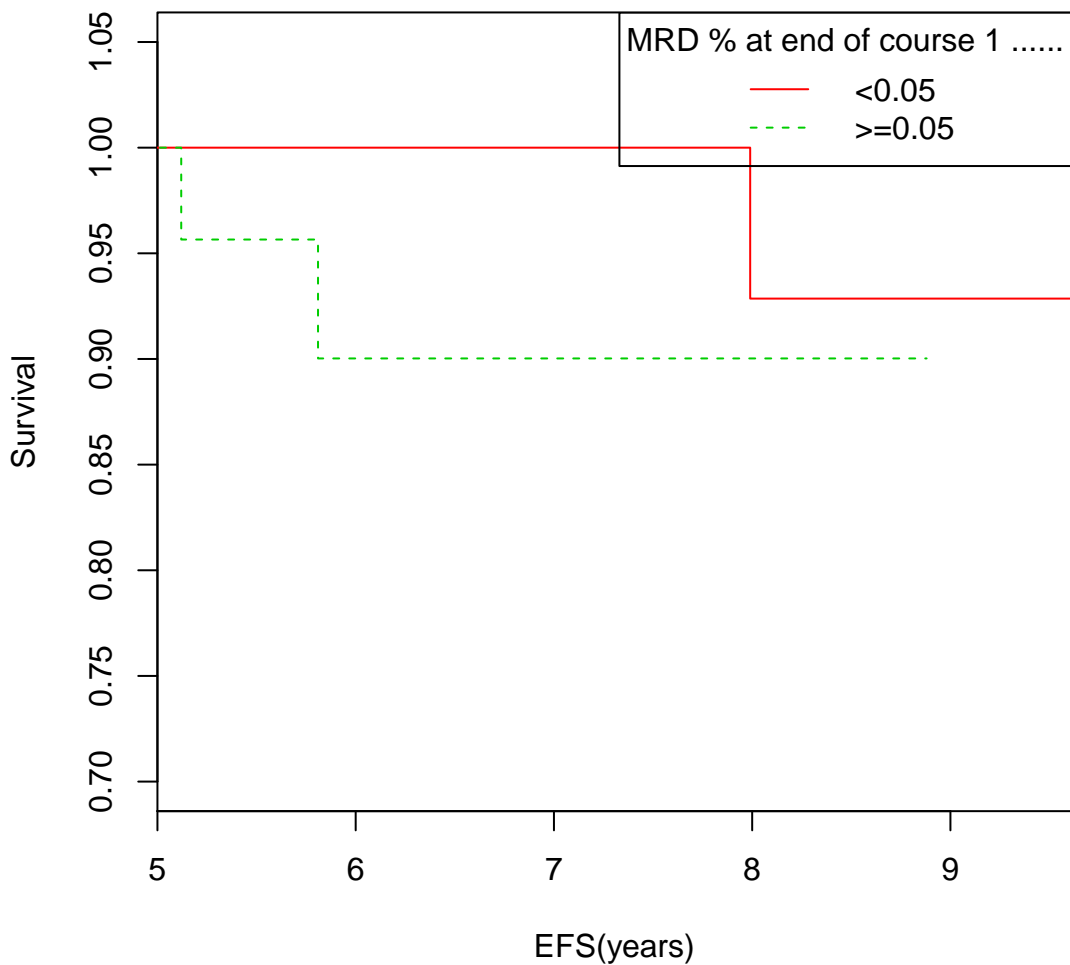

Supplement: S1 Code — (ZIP) [file pmed.1005088.s002.zip › S2 code/PROJ8_12_tbl1/PROJ8_12_tbl1_seg2_1.pdf]

## First Event

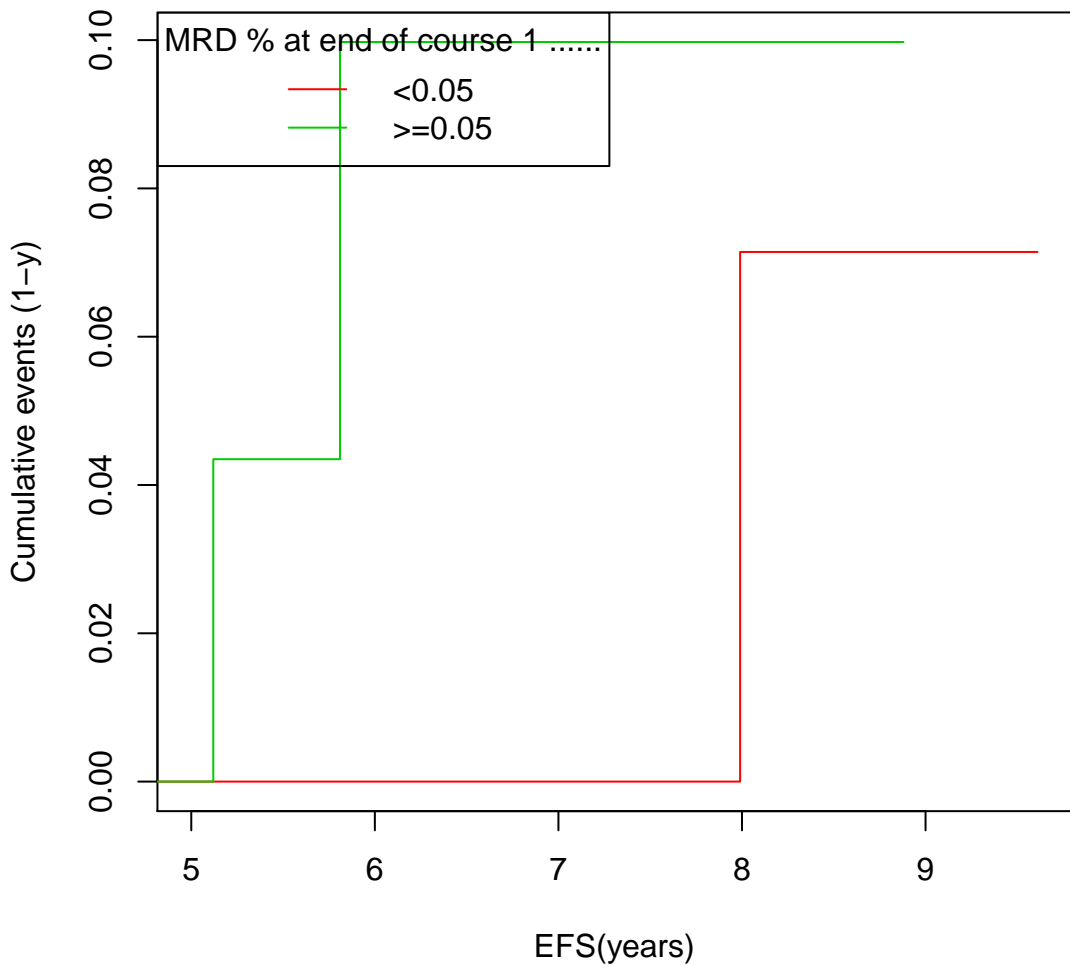

Supplement: S1 Code — (ZIP) [file pmed.1005088.s002.zip › S2 code/PROJ8_12_tbl1/PROJ8_12_tbl1_seg2_3.pdf]

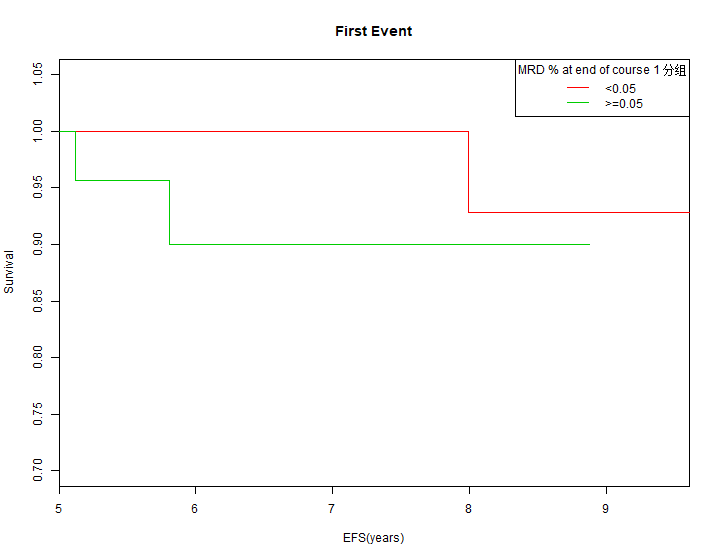

Supplement: S1 Code — (ZIP) [file pmed.1005088.s002.zip › S2 code/PROJ8_12_tbl1/PROJ8_12_tbl1_seg2_1.png]

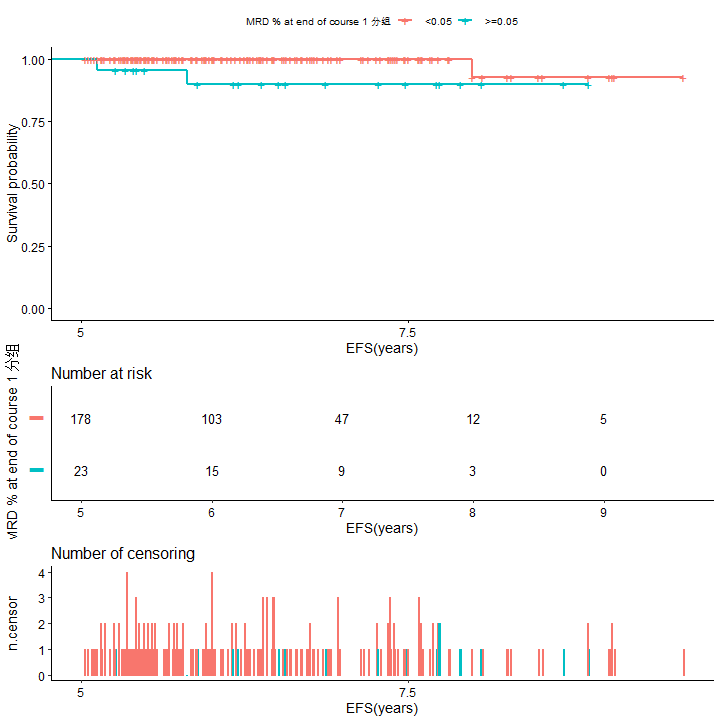

Supplement: S1 Code — (ZIP) [file pmed.1005088.s002.zip › S2 code/PROJ8_12_tbl1/PROJ8_12_tbl1_seg2_0.png]

## First Event

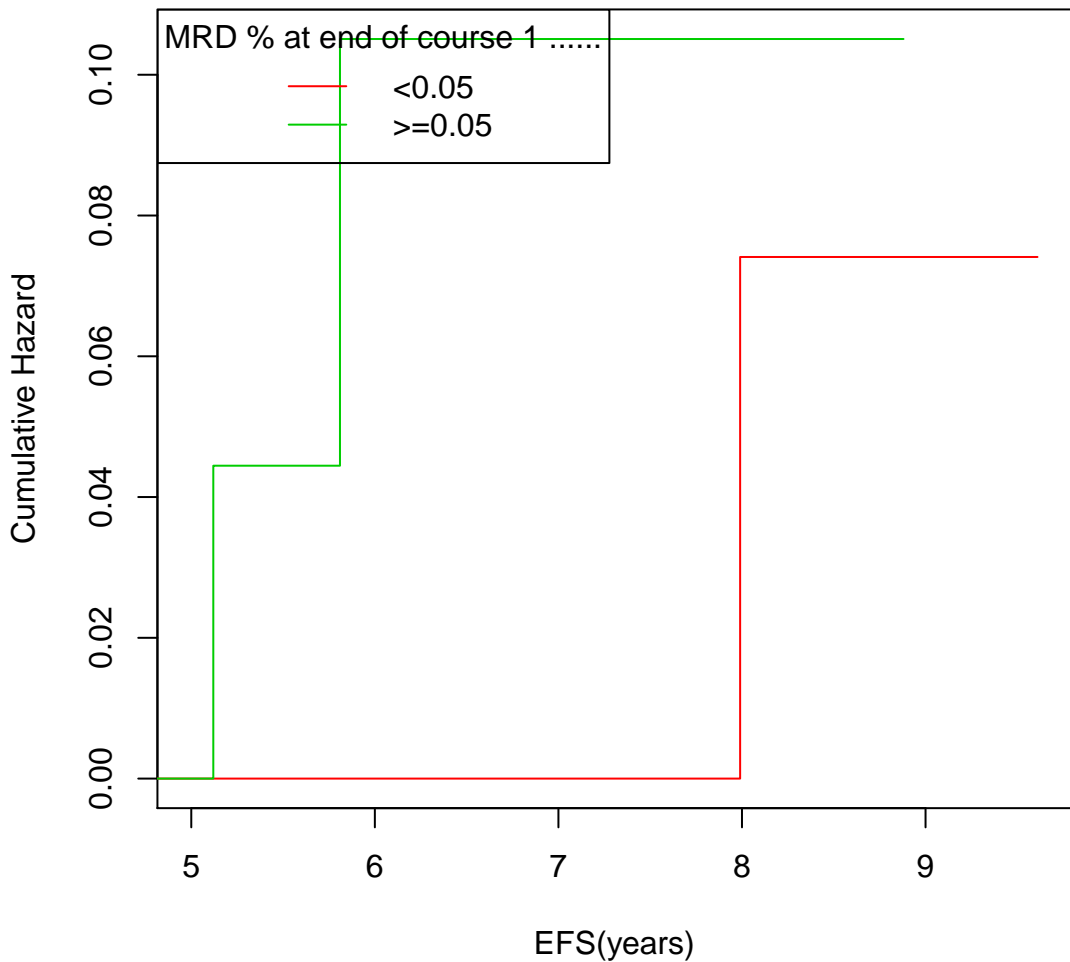

Supplement: S1 Code — (ZIP) [file pmed.1005088.s002.zip › S2 code/PROJ8_12_tbl1/PROJ8_12_tbl1_seg2_2.pdf]

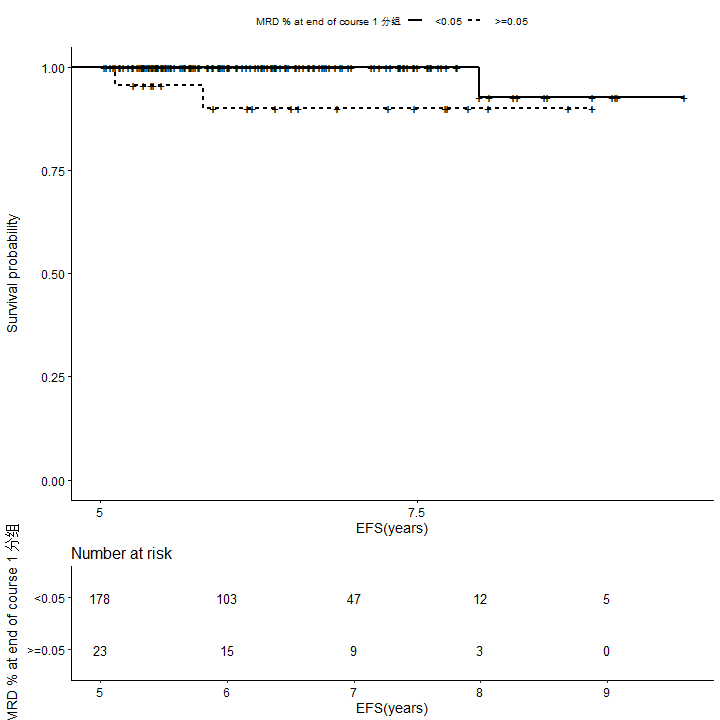

Supplement: S1 Code — (ZIP) [file pmed.1005088.s002.zip › S2 code/PROJ8_12_tbl1/PROJ8_12_tbl1_seg2_b.png]

MRD % at end of course 1 ..... — <0.05 - - - ≥0.05

Survival probability

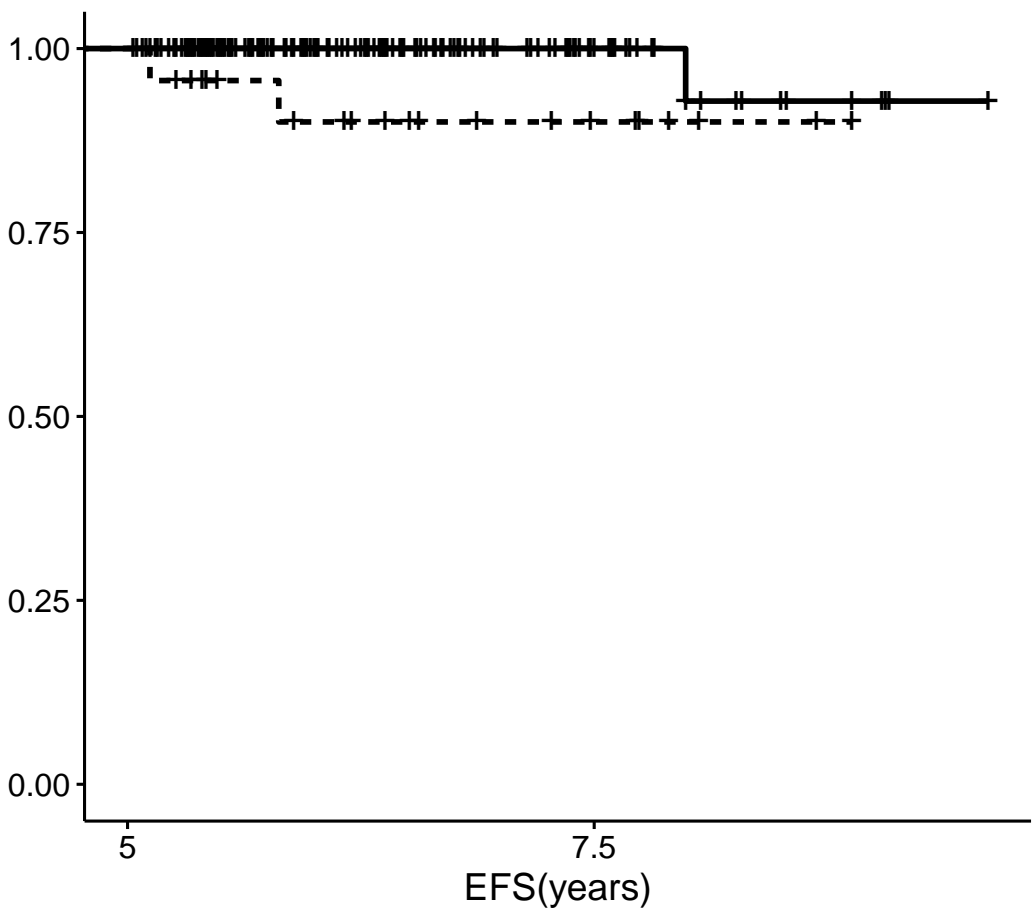

Number at risk

|       |            |     |    |    |   |
|-------|------------|-----|----|----|---|
| <0.05 | 178        | 103 | 47 | 12 | 5 |
| ≥0.05 | 23         | 15  | 9  | 3  | 0 |
|       | 5          | 6   | 7  | 8  | 9 |
|       | EFS(years) |     |    |    |   |

Supplement: S1 Code — (ZIP) [file pmed.1005088.s002.zip › S2 code/PROJ8_12_tbl1/PROJ8_12_tbl1_seg2_b.pdf]

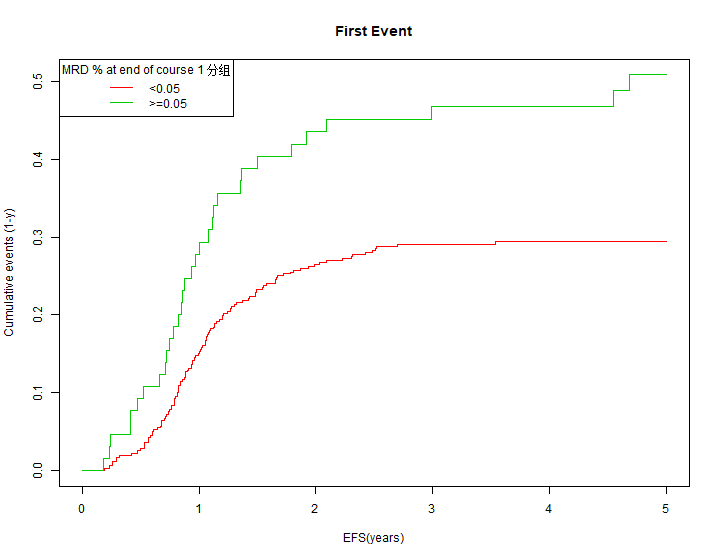

Supplement: S1 Code — (ZIP) [file pmed.1005088.s002.zip › S2 code/PROJ8_12_tbl1/PROJ8_12_tbl1_seg1_3.png]

## First Event

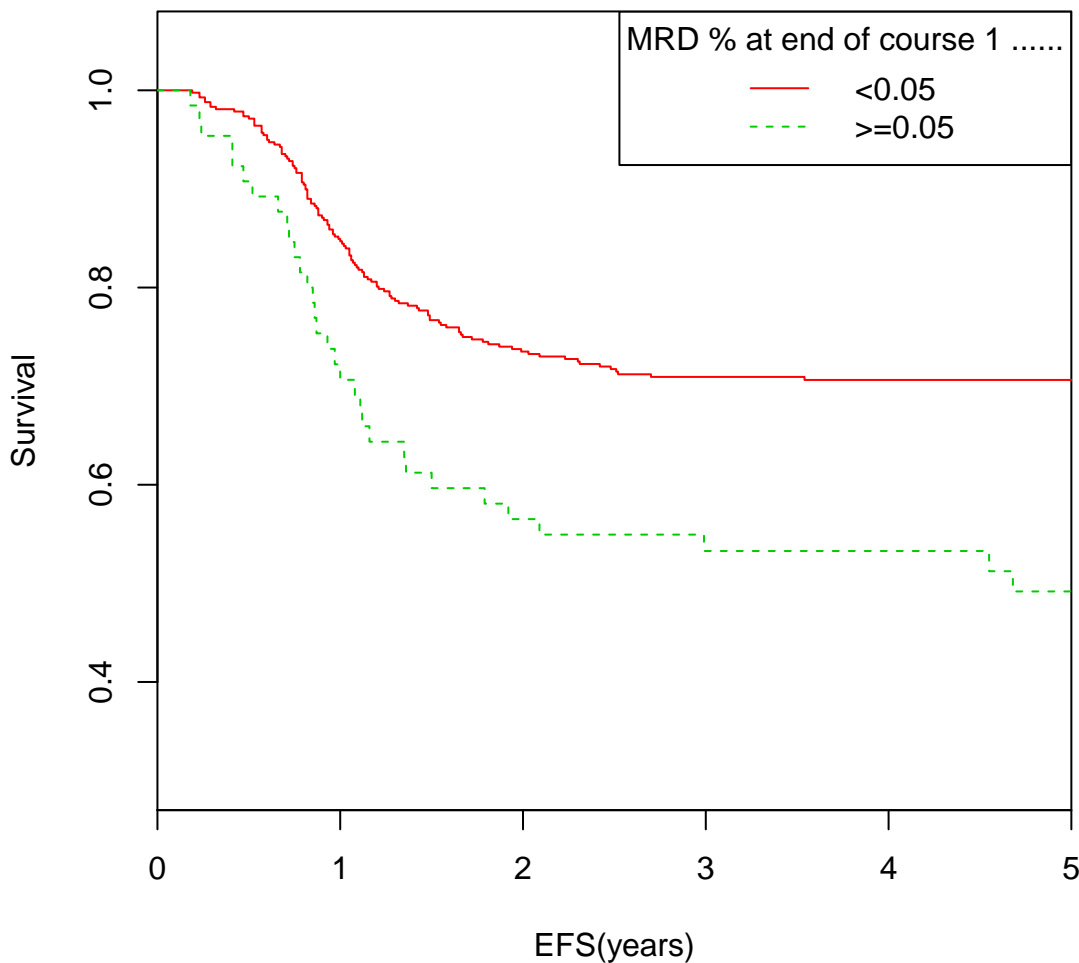

Supplement: S1 Code — (ZIP) [file pmed.1005088.s002.zip › S2 code/PROJ8_12_tbl1/PROJ8_12_tbl1_seg1_1.pdf]

MRD % at end of course 1 ..... + <0.05 + ≥0.05

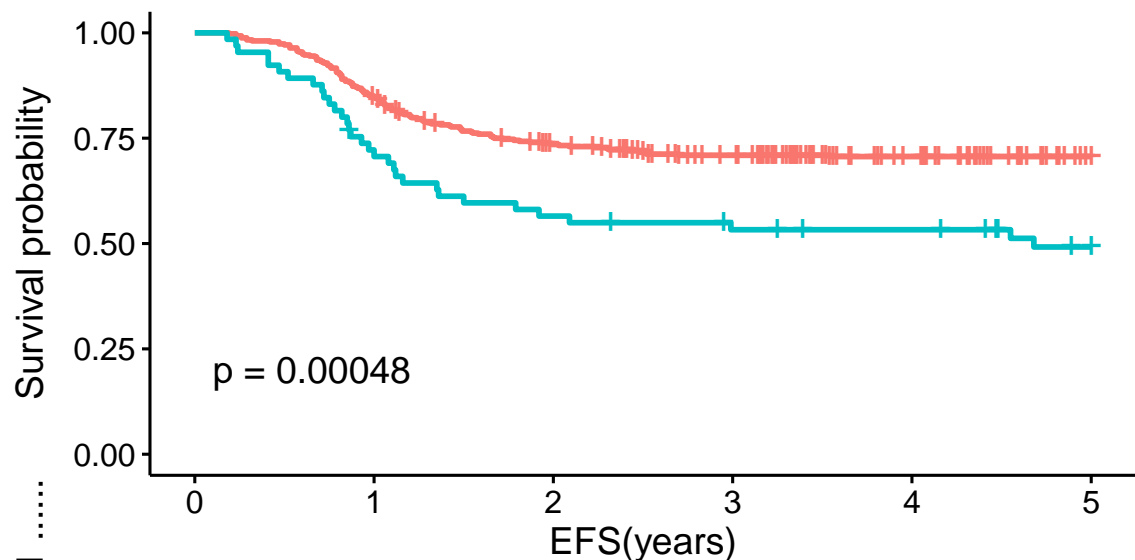

Number at risk

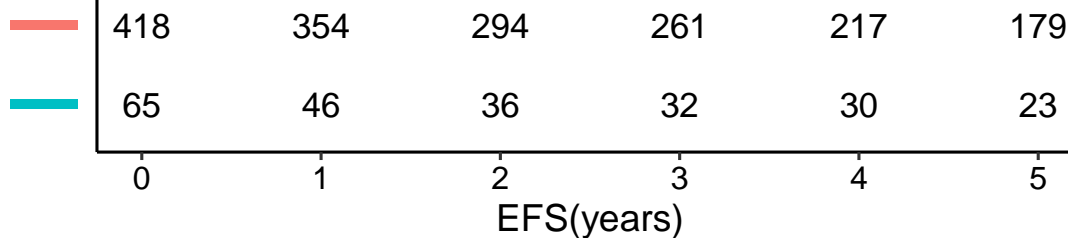

Number of censoring

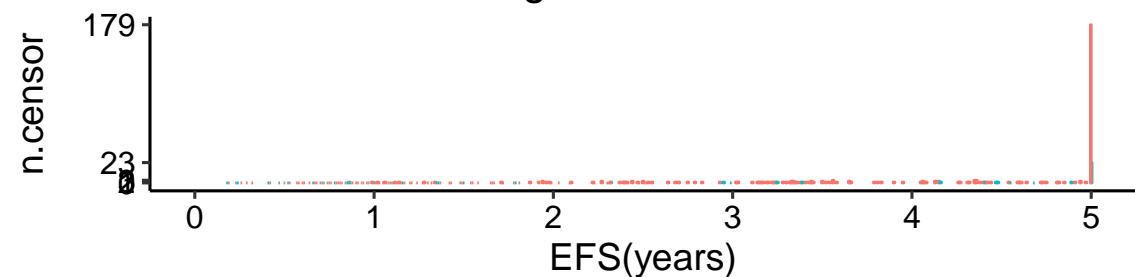

Supplement: S1 Code — (ZIP) [file pmed.1005088.s002.zip › S2 code/PROJ8_12_tbl1/PROJ8_12_tbl1_seg1_0.pdf]

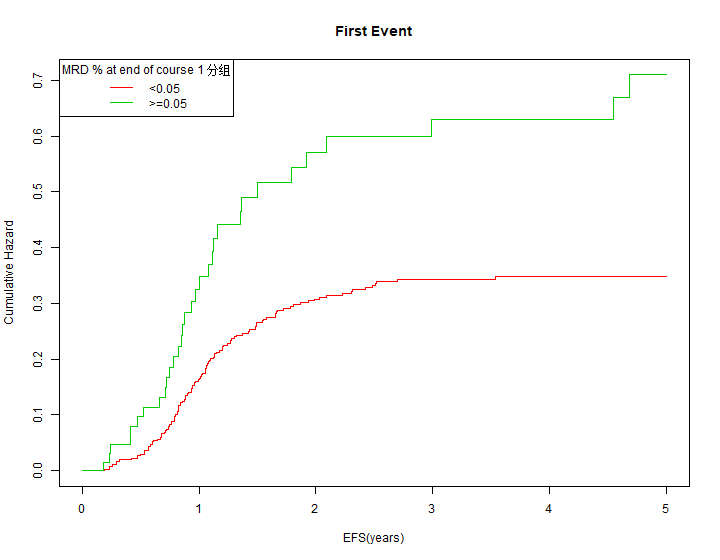

Supplement: S1 Code — (ZIP) [file pmed.1005088.s002.zip › S2 code/PROJ8_12_tbl1/PROJ8_12_tbl1_seg1_2.png]

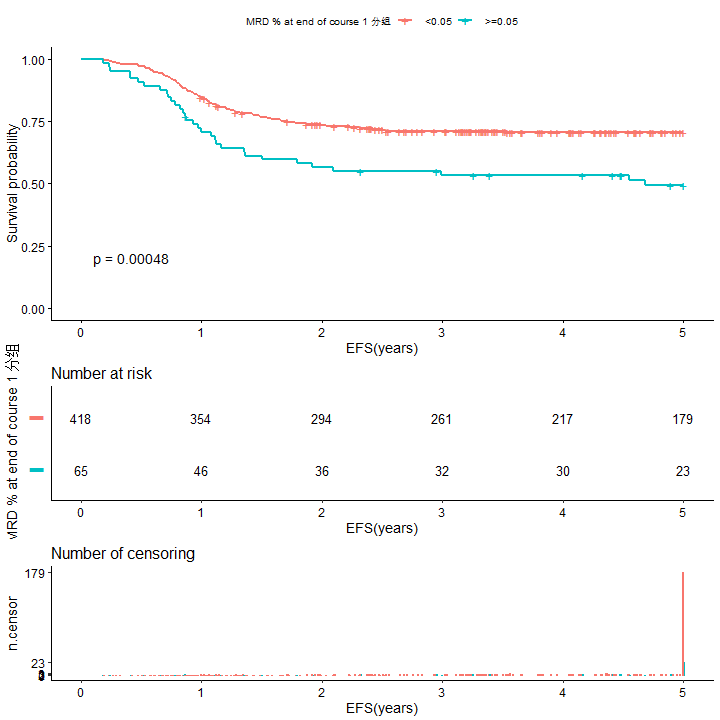

Supplement: S1 Code — (ZIP) [file pmed.1005088.s002.zip › S2 code/PROJ8_12_tbl1/PROJ8_12_tbl1_seg1_0.png]

## First Event

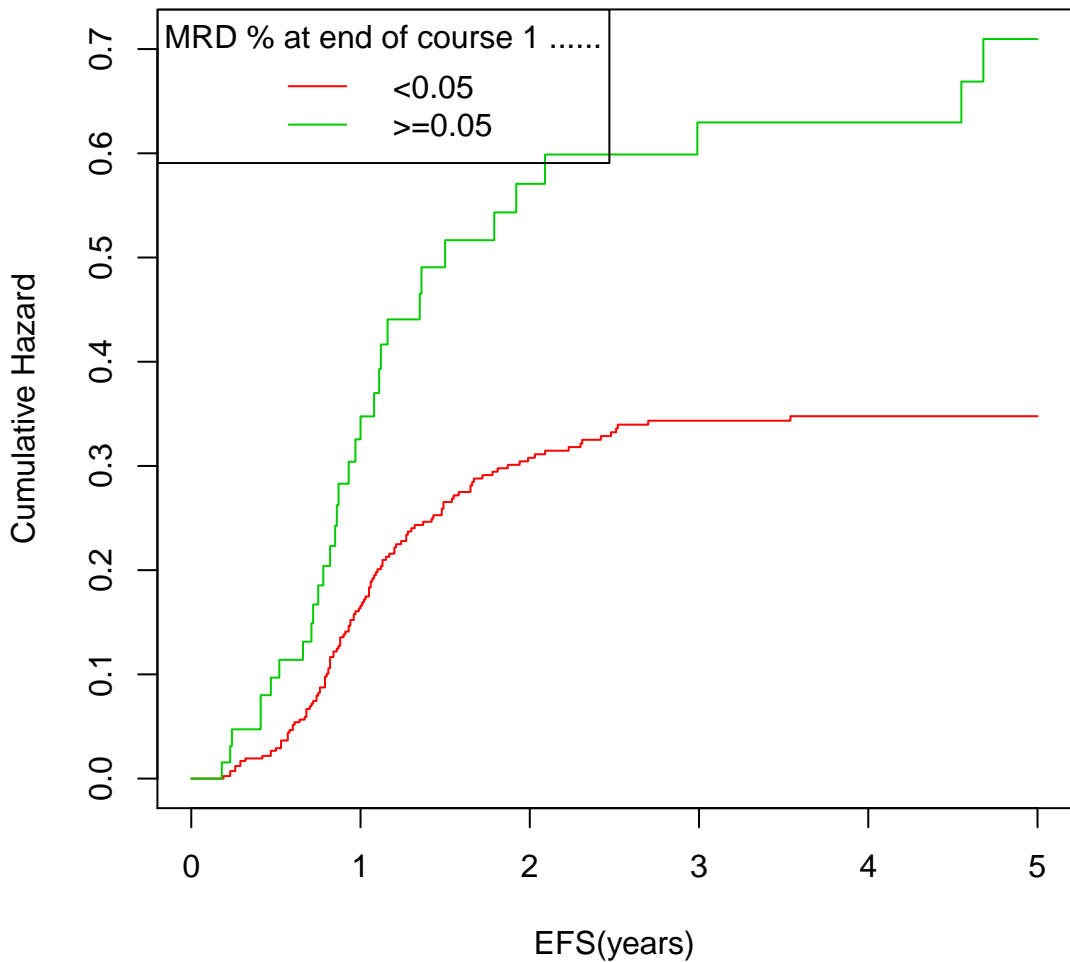

Supplement: S1 Code — (ZIP) [file pmed.1005088.s002.zip › S2 code/PROJ8_12_tbl1/PROJ8_12_tbl1_seg1_2.pdf]

## First Event

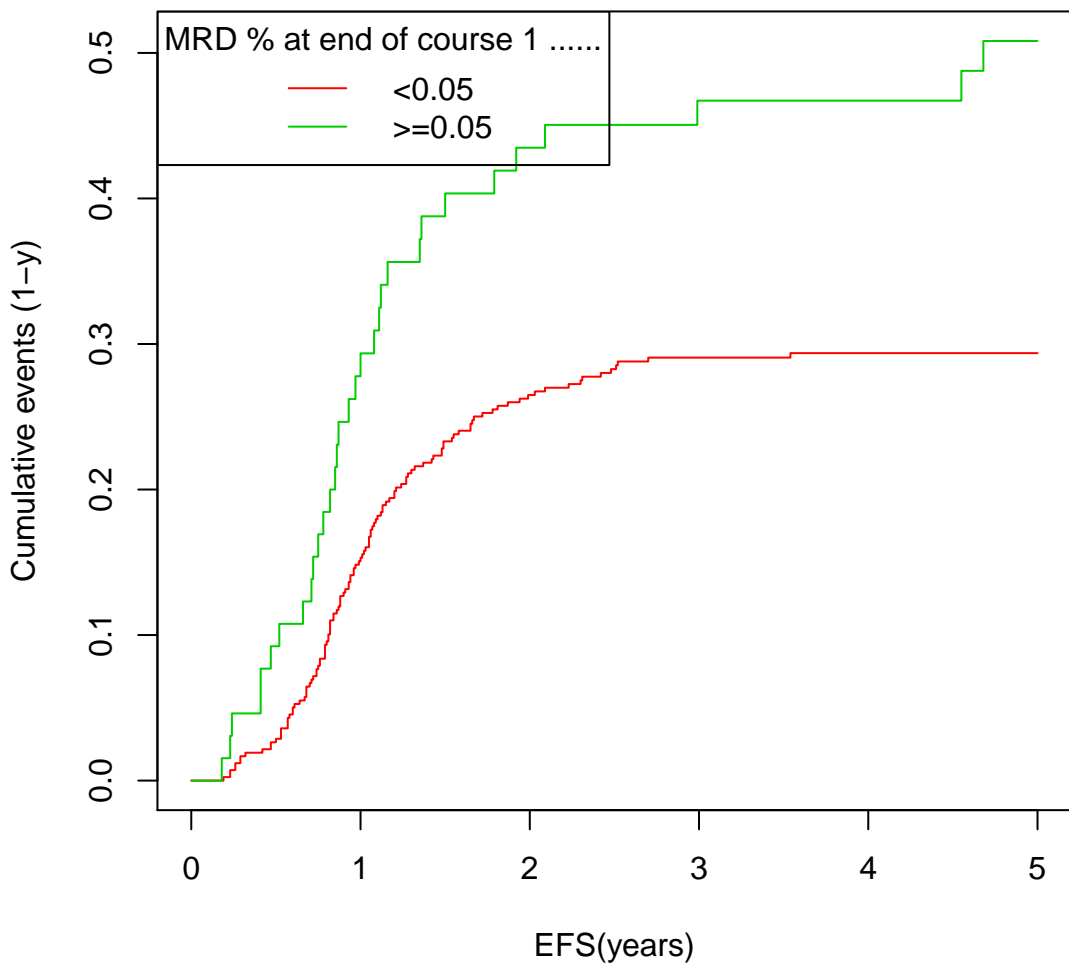

Supplement: S1 Code — (ZIP) [file pmed.1005088.s002.zip › S2 code/PROJ8_12_tbl1/PROJ8_12_tbl1_seg1_3.pdf]

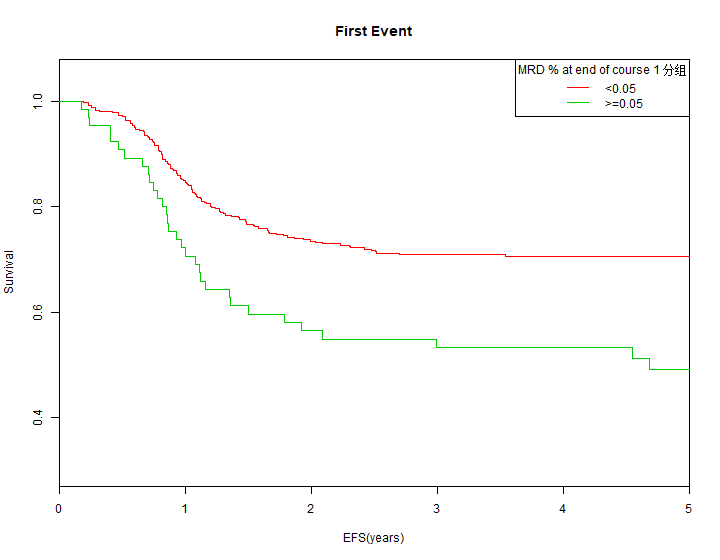

Supplement: S1 Code — (ZIP) [file pmed.1005088.s002.zip › S2 code/PROJ8_12_tbl1/PROJ8_12_tbl1_seg1_1.png]

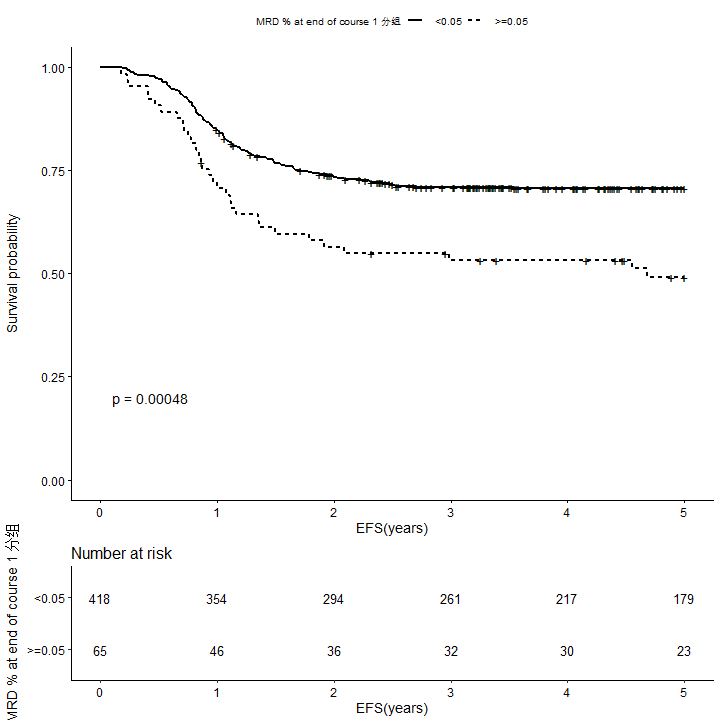

Supplement: S1 Code — (ZIP) [file pmed.1005088.s002.zip › S2 code/PROJ8_12_tbl1/PROJ8_12_tbl1_seg1_b.png]

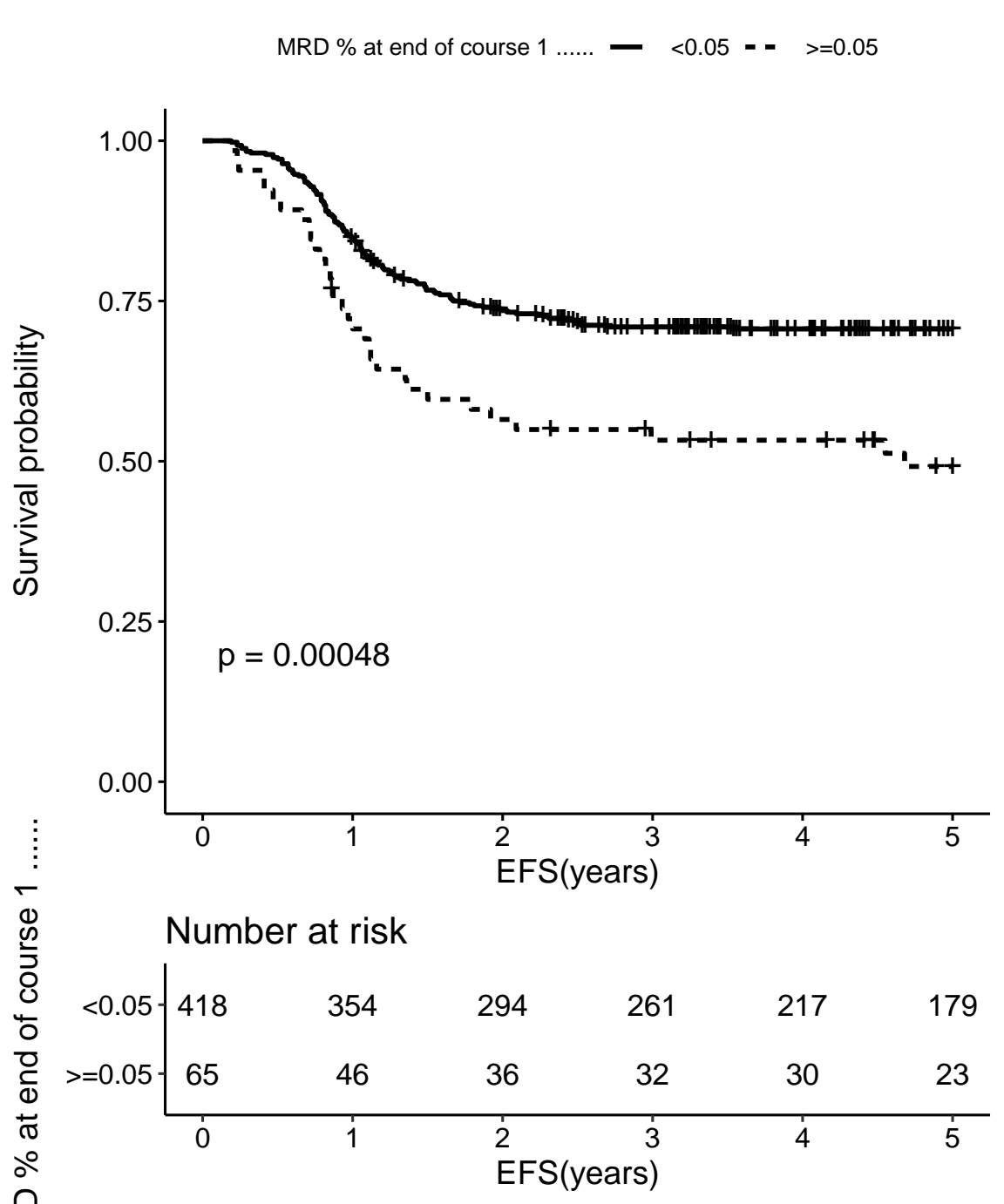

Supplement: S1 Code — (ZIP) [file pmed.1005088.s002.zip › S2 code/PROJ8_12_tbl1/PROJ8_12_tbl1_seg1_b.pdf]

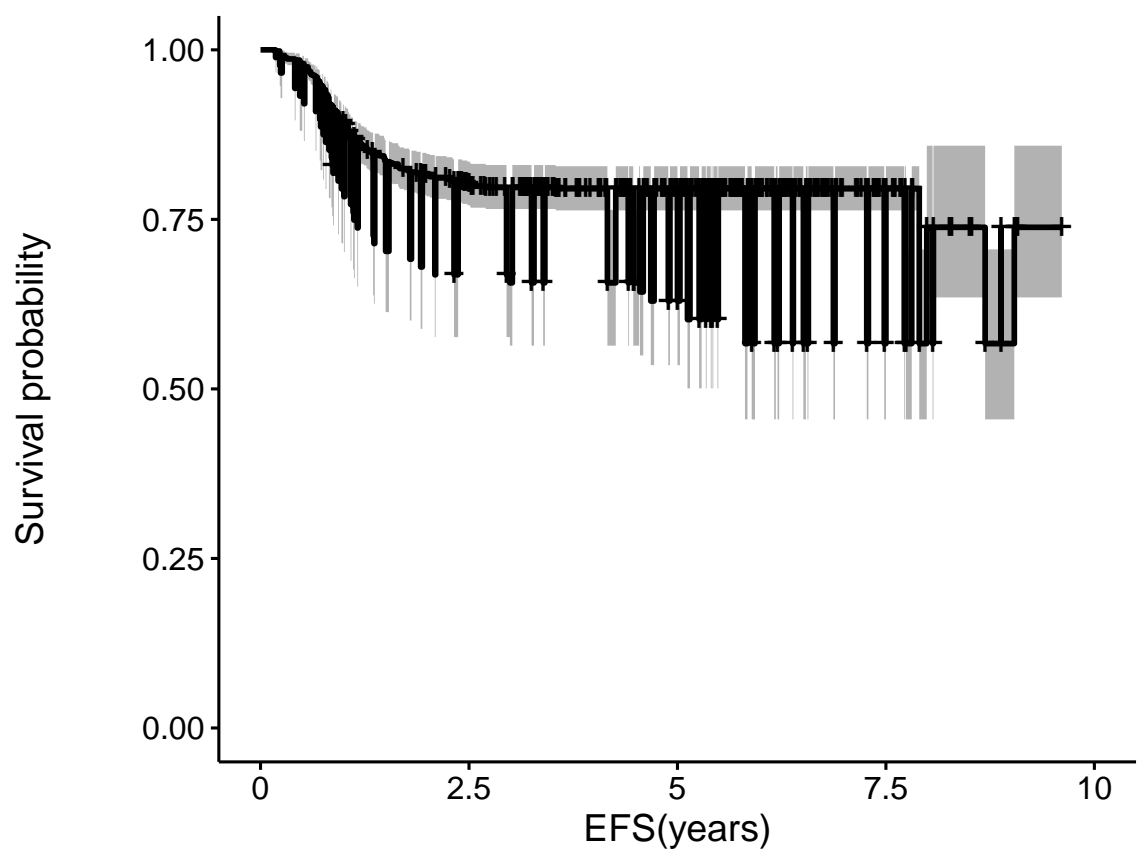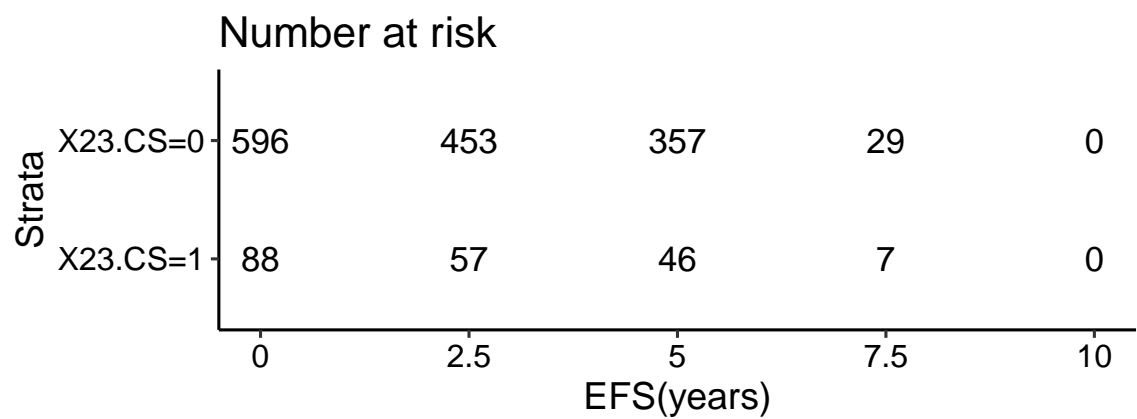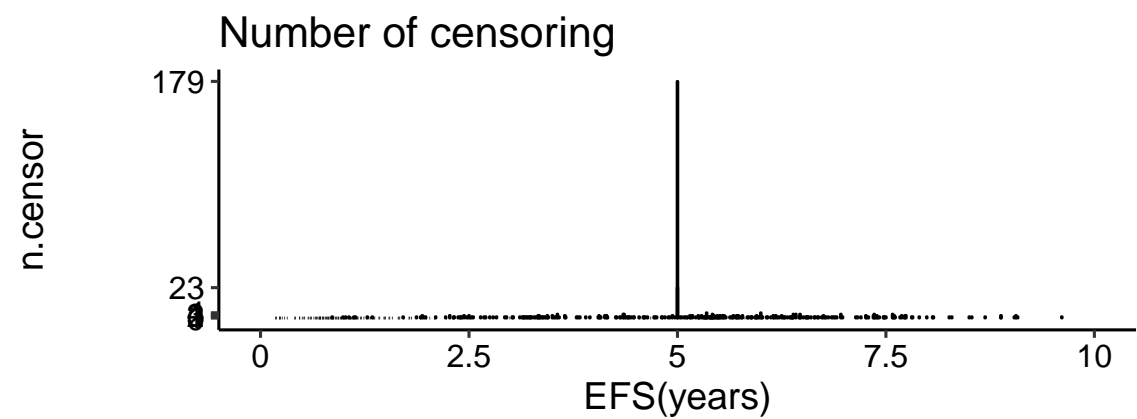

Supplement: S1 Code — (ZIP) [file pmed.1005088.s002.zip › S2 code/PROJ8_12_tbl1/PROJ8_12_tbl1_0.pdf]
