# Supplementary material for: Optimal minimal residual disease threshold in pediatric acute myeloid leukemia: A retrospective cohort study based on the TARGET database
Source: PLoS Med. 2026 May 8;23(5):e1005088. doi: 10.1371/journal.pmed.1005088 (PMC13155632; doi:10.1371/journal.pmed.1005088)
Supplement: S1 Protocol — (DOCX) [file pmed.1005088.s003.docx]

# Study Protocol

# Optimal Minimal Residual Disease Threshold in Pediatric Acute Myeloid Leukemia: A Prognostic Reevaluation Based on the TARGET Cohort

**Protocol Version**: 2.0

**Submission Date**: December 3, 2025

**Investigators**: Xiong-yu Liao, Hong Zheng, Jian-pei Fang, Dun-hua Zhou, Kun-yin Qiu

**Institution**: Department of Hematology/Oncology, Children's Medical Center, Sun Yat-sen Memorial Hospital, Sun Yat-sen University, Guangzhou, China

**Version for Ethical Review Board Submission**

## Table of Contents

1. Study Summary
2. Research Background and Scientific Rationale
3. Research Objectives and Hypotheses
4. Study Design
5. Study Population
6. Methods
7. Statistical Analysis Plan
8. Ethical Considerations
9. Data Management and Quality Control
10. Risk Management Plan
11. Timeline and Milestones
12. References

## 1. Study Summary

This comprehensive retrospective cohort study aims to redefine the optimal prognostic threshold for minimal residual disease (MRD) detection in pediatric acute myeloid leukemia (AML) using data from the TARGET-AML initiative. The conventional MRD positivity threshold of ≥0.1% by multiparametric flow cytometry (MFC) will be compared against a proposed lower threshold of ≥0.05% to evaluate its superiority in risk stratification and prognostic accuracy. The study population comprises 1,205 pediatric AML patients with complete MFC-MRD data following induction chemotherapy courses. Primary endpoints include event-free survival (EFS) analysis using advanced statistical methods including Kaplan-Meier survival curves, Cox proportional hazards models, receiver operating characteristic (ROC) curves, and net reclassification improvement (NRI) analysis. The study aims to provide evidence-based recommendations for refining MRD monitoring standards in pediatric AML clinical practice.

## 2. Research Background and Scientific Rationale

Pediatric acute myeloid leukemia represents a heterogeneous group of hematologic malignancies characterized by clonal proliferation of myeloid precursors with impaired differentiation. Despite significant advancements in risk-adapted therapy approaches, including allogeneic hematopoietic stem cell transplantation for high-risk patients, relapse remains the predominant cause of treatment failure, with 5-year event-free survival rates plateauing at approximately 50-60% in contemporary clinical trials. Minimal residual disease monitoring has emerged as a critical component of modern risk stratification in acute leukemias. Among various MRD detection methodologies, multiparametric flow cytometry-based MRD assessment offers the advantage of rapid turnaround time, widespread availability, and applicability to nearly all patients regardless of genetic markers. The conventionally accepted threshold of 0.1% for defining MRD positivity has demonstrated prognostic utility across multiple studies, yet evolving technological capabilities in flow cytometry warrant reevaluation of this standard. Recent evidence suggests that lower levels of residual disease, previously classified as MRD-negative by the 0.1% threshold, may still harbor significant prognostic implications. Technological advancements now enable reliable detection of leukemic cells at levels as low as 0.01%, raising important questions about the optimal threshold for clinical decision-making. This study addresses a critical evidence gap by systematically evaluating a lower MRD threshold in a large, well-annotated pediatric AML cohort.

## 3. Research Objectives and Hypotheses

### 3.1 Primary Objectives

1. To determine the optimal MRD threshold for predicting 5-year event-free survival following Induction Course 1 and Course 2 chemotherapy using ROC curve analysis.
2. To compare the prognostic discrimination capacity of the traditional 0.1% MRD threshold versus a proposed 0.05% threshold through Kaplan-Meier survival analysis and hazard ratio calculations.
3. To quantify the improvement in risk classification accuracy using net reclassification improvement (NRI) analysis when applying the lower MRD threshold.

### 3.2 Secondary Objectives

1. To validate the consistency of the proposed 0.05% MRD threshold across established genetic risk subgroups (low-risk, standard-risk, high-risk).
2. To establish the independent prognostic value of the novel MRD threshold through multivariate Cox regression analysis adjusting for known clinical and molecular variables.
3. To assess the clinical utility of the lower threshold in guiding treatment decisions, particularly regarding transplantation candidacy.

### 3.3 Research Hypotheses

- **Primary Hypothesis**: An MRD threshold of 0.05% provides superior prognostic discrimination compared to the conventional 0.1% threshold for predicting 5-year EFS in pediatric AML.
- **Secondary Hypothesis**: The 0.05% threshold demonstrates consistent prognostic performance across all genetic risk categories and offers significant net reclassification improvement.

## 4. Study Design

### 4.1 Study Type

Retrospective cohort study utilizing existing data from the TARGET-AML database.

### 4.2 Data Source

The Therapeutically Applicable Research to Generate Effective Treatments (TARGET) initiative database, version dated April 28, 2021, containing comprehensive clinical and laboratory data from pediatric AML patients treated between September 1996 and December 2016.

### 4.3 Study Period

Data collection period: September 1996 to December 2016 Analysis period: January 2025 to June 2025

### 4.4 Study Setting

Multicenter collaborative analysis with primary data management and statistical analysis conducted at Sun Yat-sen Memorial Hospital, Sun Yat-sen University.

### 4.5 Study Schema

Patient Identification (N=1,205)

↓

Inclusion/Exclusion Criteria Application

↓

Data Extraction and Validation

↓

Stratification by MRD Thresholds (0.1% vs 0.05%)

↓

Primary Analysis: EFS Comparison

↓

Secondary Analyses: Subgroup, NRI, Multivariate

↓

Results Interpretation and Validation

## 5. Study Population

### 5.1 Inclusion Criteria

- Pediatric patients (<18 years at diagnosis) with newly diagnosed de novo AML (non-M3 subtype)
- Availability of MFC-MRD data measured after completion of Induction Course 1 and/or Course 2
- Treatment according to one of the specified protocols: AAML03P1, AAML0531, or AAML1031
- Complete baseline clinical and molecular characterization data
- Minimum follow-up duration of 36 months for surviving patients

### 5.2 Exclusion Criteria

- Acute promyelocytic leukemia (M3 AML) patients
- Patients with incomplete MRD data or key prognostic variables
- Secondary AML or therapy-related AML cases
- Patients with insufficient follow-up data

### 5.3 Sample Size Justification

The initial TARGET-AML cohort included 1,205 pediatric patients with non-M3 AML. Based on power calculations for detecting a hazard ratio of 2.0 for EFS with 80% power at α=0.05, the available sample size provides adequate statistical power for the proposed analyses. The cohort characteristics demonstrate balanced representation across key prognostic subgroups.

## 6. Methods

### 6.1 Data Collection Procedures

Data will be extracted from the TARGET database using standardized data collection forms. The following variable categories will be included:

- **Demographic data**: Age at diagnosis, gender, ethnicity
- **Disease characteristics**: WBC count at diagnosis, bone marrow and peripheral blast percentages, FAB classification, central nervous system involvement status
- **Genetic markers**: Karyotype, FLT3-ITD, NPM1, CEBPA, and WT1 mutation status
- **Treatment data**: Chemotherapy protocol, response assessment after each induction course, stem cell transplantation details
- **MRD data**: Quantitative MRD levels after Induction Courses 1 and 2
- **Outcome data**: Event-free survival, overall survival, relapse details

### 6.2 MRD Assessment Methodology

MRD was quantified using standardized multiparametric flow cytometry protocols with a minimum sensitivity of 0.01%. The methodology included:

- **Sample processing**: Bone marrow aspirates collected at the end of Induction Courses 1 and 2
- **Antibody panels**: Standardized 8-color flow cytometry panels targeting leukemia-associated immunophenotypes
- **Analysis protocol**: A minimum of 500,000 events were acquired using BD FACS Canto II flow cytometers
- **Quality control**: Regular instrument calibration and compensation controls using compensation beads
- **Data interpretation**: Expert hematopathologist review of all MRD assessments

### 6.3 Variable Definitions

- **Event-free survival (EFS)**: Time from diagnosis to first event (induction failure, relapse, secondary malignancy, or death from any cause)
- **MRD positivity**: Defined using two thresholds: traditional (≥0.1%) and novel (≥0.05%)
- **Complete remission**: <5% blasts in morphologically normal bone marrow with recovery of peripheral counts
- **Genetic risk groups**:
  - Low risk: inv(16)/t(16;16) or t(8;21)
  - High risk: monosomy 7 or del(5q)
  - Standard risk: All other cases with adequate cytogenetic data

### 6.4 Data Quality Assurance

- Double data entry for key variables with discrepancy resolution
- Range checks for continuous variables
- Consistency checks across related variables
- Missing data assessment and appropriate handling strategies

## 7. Statistical Analysis Plan

### 7.1 Primary Analysis

**Survival Analysis**: Kaplan-Meier method will be used to estimate EFS distributions stratified by MRD status at both thresholds. The log-rank test will compare survival curves.

**ROC Analysis**: Receiver operating characteristic curves will determine the optimal MRD threshold for predicting 5-year EFS status using Youden's index.

### 7.2 Secondary Analyses

**Cox Regression**: Univariate and multivariate Cox proportional hazards models will assess the association between MRD thresholds and EFS, adjusting for relevant covariates. **Net Reclassification Improvement**: Categorical NRI will quantify improvement in risk prediction using the novel threshold. **Subgroup Analysis**: Consistency of the MRD threshold will be evaluated within predefined genetic risk categories.

### 7.3 Sensitivity Analyses

- Multiple imputation for missing data
- Landmark analysis at 3 months post-induction
- Competing risks analysis considering non-relapse mortality

### 7.4 Statistical Software

- Primary analysis: R software (version 4.1.0)
- Secondary analysis: SPSS (version 26.0)
- Sample size justification: Power analysis using PASS software

## 8. Ethical Considerations

### 8.1 Ethical Approval

The original TARGET study obtained approval from institutional review boards of all participating institutions. This retrospective analysis protocol will be submitted to the Ethics Committee of Sun Yat-sen Memorial Hospital for expedited review.

### 8.2 Informed Consent

Waiver of informed consent will be requested as the study involves analysis of existing de-identified data. The original TARGET study obtained appropriate consent from participants or guardians.

### 8.3 Privacy and Confidentiality

- All patient identifiers removed from analytic dataset
- Data stored on encrypted servers with password protection
- Limited access to authorized research personnel only
- Data transfer using secure encrypted methods

### 8.4 Risk-Benefit Assessment

**Risks**: Minimal risk study; potential breach of confidentiality mitigated by de-identification and secure data handling. **Benefits**: No direct benefits to participants; potential improvement in risk stratification for future pediatric AML patients.

## 9. Data Management and Quality Control

### 9.1 Data Handling Procedures

- Secure electronic data capture system
- Regular data backup procedures
- Version control for analytic datasets
- Audit trail maintenance

### 9.2 Quality Assurance Measures

- Protocol adherence monitoring
- Data quality checks at multiple timepoints
- Independent statistical validation
- Regular team meetings for quality review

### 9.3 Data Monitoring

Independent data monitoring committee review of interim analyses and final results.

## 10. Risk Management Plan

### 10.1 Potential Risks

- Data security breaches
- Statistical model misspecification
- Incomplete case ascertainment

### 10.2 Mitigation Strategies

- Regular security audits
- Pre-specified statistical analysis plan
- Sensitivity analyses for missing data
- Peer review of analytical methods

## 11. Timeline and Milestones

**Months 1-2**: Data extraction and cleaning

**Months 3-4**: Primary statistical analysis

**Month 5**: Results validation and sensitivity analyses

**Month 6**: Manuscript preparation and submission

**Month 7**: Ethics committee reporting

## 12. References

1. Tarlock K, Lamble AJ, Wang YC, et al. CEBPA-bZip mutations are associated with favorable prognosis in de novo AML: a report from the Children's Oncology Group. *Blood*. 2021;138(13):1137-1147. doi:10.1182/blood.2020009652
2. Pollard JA, Guest E, Alonzo TA, et al. Gemtuzumab Ozogamicin Improves Event-Free Survival and Reduces Relapse in Pediatric *KMT2A*-Rearranged AML: Results From the Phase III Children's Oncology Group Trial AAML0531. *J Clin Oncol*. 2021;39(28):3149-3160. doi:10.1200/JCO.20.03048
3. Huang BJ, Meyer LK, Alonzo TA, et al. Hematopoietic Stem Cell Transplantation Outcomes for High-Risk AML: A Report From the Children's Oncology Group. *J Clin Oncol*. 2025;43(17):1961-1971. doi:10.1200/JCO-24-01841
4. Zarnegar-Lumley S, Caldwell KJ, Rubnitz JE. Relapsed acute myeloid leukemia in children and adolescents: current treatment options and future strategies. *Leukemia*. 2022;36(8):1951-1960. doi:10.1038/s41375-022-01619-9
5. Heuser M, Freeman SD, Ossenkoppele GJ, et al. 2021 Update on MRD in acute myeloid leukemia: a consensus document from the European LeukemiaNet MRD Working Party. *Blood*. 2021;138(26):2753-2767. doi:10.1182/blood.2021013626
6. Tettero JM, Freeman S, Buecklein V, Venditti A, Maurillo L, Kern W, et al. Technical aspects of flow cytometry-based measurable residual disease quantification in acute myeloid leukemia: experience of the European LeukemiaNet MRD Working Party. HemaSphere. 2022;6(1):e676.
7. Maurer-Granofszky M, Kohrer S, Fischer S, et al. Genomic breakpoint-specific monitoring of measurable residual disease in pediatric non-standard-risk acute myeloid leukemia. Haematologica. 2024;109(3):740-750. doi:10.3324/haematol.2022.282424
8. Qiu KY, Liao XY, Liu Y, et al. Poor outcome of pediatric patients with acute myeloid leukemia harboring high FLT3/ITD allelic ratios. Nat Commun. 2022;13(1):3679. Published 2022 Jun 27. doi:10.1038/s41467-022-31489-9
9. Brodersen LE, Gerbing RB, Pardo ML, et al. Morphologic remission status is limited compared to ΔN flow cytometry: a Children's Oncology Group AAML0531 report. Blood Adv. 2020;4(20):5050-5061. doi:10.1182/bloodadvances.2020002070
10. McCarthy N, Gui G, Dumezy F, et al. Pre-emptive detection and evolution of relapse in acute myeloid leukemia by flow cytometric measurable residual disease surveillance. Leukemia. 2024;38(8):1667-1673. doi:10.1038/s41375-024-02300-z
11. Jen WY, Sasaki K, Ravandi F, et al. Impact of measurable residual disease clearance kinetics in patients with AML undergoing intensive chemotherapy. Blood Adv. 2025;9(4):783-792. doi:10.1182/bloodadvances.2024013826
12. Pratz KW, Jonas BA, Pullarkat V, et  al. Measurable residual disease response and prognosis in treatment-naïve acute myeloid leukemia with venetoclax and azacitidine. J Clin Oncol. 2022;40(8):855–65.
13. Dillon R, Hills R, Freeman S, Potter N, Jovanovic J, Ivey A, et al. Molecular MRD status and outcome after transplantation in NPM1-mutated AML. Blood. 2020;135(9):680–8.
14. Kayser S, Benner A, Thiede C, Martens U, Huber J, Stadtherr P, et al. Pretransplant NPM1 MRD levels predict outcome after allogeneic hematopoietic stem cell transplantation in patients with acute myeloid leukemia. Blood Cancer J. 2016;6(7):e449.
15. Bazinet A, Kadia T, Short NJ, et al. Undetectable measurable residual disease is associated with improved outcomes in AML irrespective of treatment intensity. Blood Adv. 2023;7(13):3284–96.
16. Grob T, Sanders MA, Vonk CM, et al. Prognostic value of FLT3-internal tandem duplication residual disease in acute myeloid leukemia. J Clin Oncol. 2023;41(4):756–65.
17. Yin JAL, O'Brien MA, Hills RK, Daly SB, Wheatley K, Burnett AK. Minimal residual disease monitoring by quantitative RT-PCR in core binding factor AML allows risk stratification and predicts relapse: results of the United Kingdom MRC AML-15 trial. Blood. 2012;120(14):2826–35.
18. Rücker FG, Agrawal M, Corbacioglu A, et al. Measurable residual disease monitoring in acute myeloid leukemia with t(8;21)(q22;q22.1): results from the AML Study Group. Blood. 2019;134(19):1608–18.
19. Jourdan E, Boissel N, Chevret S, et al. Prospective evaluation of gene mutations and minimal residual disease in patients with core binding factor acute myeloid leukemia. Blood. 2013;121(12):2213–23.
20. Bataller A, Oñate G, Diaz-Beyá M, et al. Acute myeloid leukemia with NPM1 mutation and favorable European LeukemiaNet category: outcome after preemptive intervention based on measurable residual disease. Br J Haematol. 2020;191(1):52–61.
21. Nachmias B, Haran A, Yisraeli Salman M, Stein EM. Measurable residual disease-guided therapy in acute myeloid leukaemia: Practical insights. Br J Haematol. Published online August 31, 2025. doi:10.1111/bjh.70038
