# Supplementary material for: Optimal minimal residual disease threshold in pediatric acute myeloid leukemia: A retrospective cohort study based on the TARGET database
Source: PLoS Med. 2026 May 8;23(5):e1005088. doi: 10.1371/journal.pmed.1005088 (PMC13155632; doi:10.1371/journal.pmed.1005088)
Supplement: Attachment — Submitted filename: point-by-point response.docx [file pmed.1005088.s006.docx]

Dear Dr. Evangelia Fourli, Ph.D.
Associate Editor

***Plos Medicine***

2026-3-17

We would like to thank the editor for the decision letter and advice on our manuscript entitled “**Optimal Minimal Residual Disease Threshold in Pediatric Acute Myeloid Leukemia: A Prognostic Reevaluation Based on the TARGET Cohort**(Manuscript ID: PMEDICINE-D-25-04201R1)”.

We also thank the reviewer for the constructive comments and suggestions. We have strived our best to answer all the queries accordingly. Our point-by-point response to the comments are listed below this letter. In addition, we have revised the manuscript accordingly, and the amendments are indicated by red font in the revised manuscript.

We hope that our revised manuscript is now acceptable for publication in ***Plos Medicine*** and we look forward to hearing from you soon.

Yours sincerely,

Kun-yin Qiu

**Point-by-point response to the editorial comments**

**Comments from the academic editor and editorial team:**

1. Please revisit your data availability statement to comply with PLOS Medicine requirements.

• If the data are held or will be held in a public repository, include URLs, accession numbers or DOIs. If this information will only be available after acceptance, indicate this by ticking the box below. For example: All XXX files are available from the XXX database (accession number(s) XXX, XXX.).

• If the data are all contained within the manuscript and/or Supporting Information files, enter the following: All relevant data are within the manuscript and its Supporting Information files.

• If neither of these applies but you are able to provide details of access elsewhere, with or without limitations,

please do so. For example:

Data cannot be shared publicly because of [XXX]. Data are available from the XXX Institutional Data Access / Ethics

Committee (contact via XXX) for researchers who meet the criteria for access to confidential data.

The data underlying the results presented in the study are available from (include the name of the third party and contact information or URL). This text is appropriate if the data are owned by a third party and authors do not have permission to share the data.

For more information visit: <https://journals.plos.org/plosmedicine/s/data-availability>

***Response:*** *We thank the editor for this insightful comment. We have revised the data availability statement in the "Declarations" section to fully align with PLOS policies. The data are from the public TARGET-AML repository, and we now include the required details. The updated statement reads:"The data underlying this study are publicly available from the TARGET-AML database (accession number: TARGET-AML;URL:https://ocg.cancer.gov/programs/target/data-matrix).Researchers can access the data via the repository portal without restrictions." Revised in the "Availability of data and materials" section of the manuscript（****Line 562-564, Page17****）.*

2. Please revise your abstract section to comply with our requirements. The Abstract should include three sections: Background, Methods and Findings, and Conclusions. It should also provide all the information relevant to this study type. Please see https://journals.plos.org/plosmedicine/s/submission-guidelines#loc-abstract.

***Response:*** *We have restructured the abstract into the three required sections. The* ***revised abstract*** *now includes:*

***Background*** *Minimal residual disease (MRD) monitoring is a critical prognostic tool in pediatric acute myeloid leukemia (AML). The conventional threshold for defining MRD positivity by flow cytometry is 0.1%, but technological advances warrant reevaluation to determine if a lower threshold improves risk stratification.*

***Methods and Findings*** *Using data from the TARGET-AML cohort (n=1,205 pediatric patients treated on Children's Oncology Group protocols between 1996 and 2016), we compared the prognostic performance of the conventional MRD threshold (≥0.1%) with a lower threshold (≥0.05%) after induction courses 1 and 2 for predicting 5-year event-free survival (EFS). The analysis included Kaplan-Meier survival estimates, Cox proportional hazards models, receiver operating characteristic (ROC) curves, and net reclassification improvement(NRI). The optimal threshold determined by ROC analysis was 0.05% at both time points (Area Under the Curve: 0.840 after course 1; 0.854 after course 2). The 0.05% threshold demonstrated higher hazard ratios for the composite outcome (EFS) than the 0.1% threshold (Course 1: HR=2.8, 95% CIs[2.3-3.3] vs. HRs=1.9, 95% CIs[1.1-3.2]; Course 2: HRs=3.7, 95%CIs [3.0-4.6] vs. HRs=2.7, 95%CIs[2.3-3.2]). NRI analysis showed significant improvement in risk classification with the 0.05% threshold (Overall NRI: 0.15 after course 1, 0.18 after course 2).*

***Conclusions*** *A lower MRD threshold of 0.05% improves prognostic discrimination and risk stratification in pediatric AML compared to the conventional 0.1% threshold. These findings support integrating this threshold into clinical practice and future trial designs.*

*We ensured it provides comprehensive details for a general reader, as requested.*

*Abstract completely rewritten in* ***Line 25-52, Page 2*** *in the revised manuscript.*

3. In the abstract, please include the important dependent variables that are adjusted for in the analyses. Also, please report numbers (e.g. the # of patients whose data was analysed), and the results of the analyses, using 95% CIs where applicable. The Abstract should include more details on the study question and cohort for a general reader.

***Response:****We have Enhanced abstract with precise numbers, CIs, and cohort details completely rewritten in* ***Line 25-52, Page 2*** *in the revised manuscript.*

4. The last paragraph of your introduction should be restructured to include the main aims of the study.

***Response:*** *We have revised the last paragraph of the introduction to explicitly state the aims:"This study aimed to: (1) compare the prognostic performance of the 0.1% vs. 0.05% MRD thresholds for 5-year EFS using ROC and survival analyses; (2) quantify improvement via NRI; and (3) validate the threshold across genetic risk subgroups." Restructured the introduction's final paragraph were in* ***Line 128-131, Page 5*** *in the revised manuscript.*

5. Please specify the institutional review boards in section 2.1 and the type of consent obtained, ie written or oral.

***Response:*** *We have added details to section 2.1: "The data for this study were derived from the TARGET-AML database. The original data collection within the Children’s Oncology Group (COG) trials was approved by the Institutional Review Boards(IRB) of all participating centers, including the lead IRB at the National Cancer Institute, with written informed consent obtained from all participants or their guardians. The analysis of de-identified data for this specific study was reviewed and approved by the IRB of Sun Yat-sen Memorial Hospital (Approval No. SYSEC2-2025-BA-885)." Updated section 2.1.were in* ***Line 146-152, Page 5.***

6. Section 2.2, you mention 4 protocols but you reference only 3. Please, correct as appropriate.

***Response:*** *We apologize for the oversight. In fact, only 3 protocols in this study."Revised section 2.2.in* ***Line157-158, Page 5.***

7. Figures 1, 3 and 4, the numbering on the y axis of some panels is overlapping close to 0. Please correct this if possible.

***Response:*** *We have regenerated Figures 1, 3, and 4 to resolve the y-axis numbering overlap. The revised figures now have adjusted scales and clear labeling. For example, in Figure 1, the y-axis intervals were respaced to avoid clutter near 0. Figures resubmitted as high-resolution TIFF files (300 DPI) via the NAAS tool, complying with PLOS guidelines.(****P26-29*** *in revised manuscript)*

8. Please include the statistical tests used for p-value calculation in all relevant figure and table legends.

***Response:*** *We thank the editors for highlighting the need for explicit reporting of statistical tests in figure and table legends. We have revised all relevant legends to include the specific statistical tests used for p-value calculations, ensuring full transparency and reproducibility in this revised manuscrpit.(****P26-29*** *in revised manuscript)*

**Point-by-point response to the reviewers’ comments**

**Reviewer #1:** This is cohort study that compared the threshold for minimal residual disease (MRD) for paediatric acute myeloid leukaemia (AML) patients. It compared the traditional (0.1%) with a newly proposed threshold (0.05%).

**Major:**

1. Multivariable analysis selected variables based on arbitrary p-value threshold (<0.1) which is not optimised for prediction performance. Please consider either AIC or LASSO method for variable selection, both of which optimises prediction performance. Please also make sure the aim of the multivariate analysis is clear - right now it does not indicate whether it is for prognostic or explanatory research even though implied from the research question.

***Response:*** *We fully concur with your perspective. In the revised manuscript, we have employed the Akaike Information Criterion (AIC) for variable selection to optimize the model's predictive performance. Furthermore, we have explicitly stated in the "Statistical Analysis" section (2.5.1) that the aim of this multivariable Cox regression is for prognostic research, identifying independent predictors of Event-Free Survival (EFS).*

*Specific Revisions:*

- *Methods (Section 2.5.1):The text "Variables with a p-value<0.1 in univariate analysis were subsequently incorporated..." has been revised to: "Variable selection for the multivariable Cox proportional hazards model was performed using the Akaike Information Criterion (AIC) to optimize prognostic prediction performance for first event. in* ***Line 248-250, Page 8*** *in our revised manuscript"*
- *Results (Section 3.6) and Table 3:The multivariable analysis has been re-run using the AIC-based model. The updated results confirm that MRD ≥0.05% remains the strongest independent prognostic factor* ***Line 391-397, Page 12*** *in Section 3.6 and in revised Table 3(****Page 24)****.*

2. Also because of how the variables were selected for multivariable analysis, both MRD >= 0.1% and MRD >= 0.05% were included in the model which are hard to interpret (there were overlaps in the categories). Please consider creating a categorical variable of 3 levels: MRD < 0.05%; 0.05 - <0.1%; >=0.01% so that dose response associations are clearer.

***Response:*** *This is an excellent suggestion. Accordingly, we have redefined MRD levels as an ordinal three-category variable. This approach eliminates category overlap and clearly reveals a dose-response relationship between MRD level and poorer outcome.*

*Specific Revisions:*

- *Methods (Section 2.3):Added: "For dose-response analysis, MRD levels were categorized into three ordered groups: <0.05%, 0.05% to <0.1%, and ≥0.1%. （****Line 187-188，Page 6*** *in our revised manuscript）"*
- *Results (Sections 3.4):We have added new analyses using the three-category variable. For example: "Moreover, using the three-category variable, a clear dose-response relationship was observed after Induction Course 1: compared to patients with MRD<0.05%(EFS:57.5%, 95%CIs:54.4%–60.9%), those with MRD 0.05-<0.1% had an EFS of 33.3% (95%CIs: 17.1%-64.1%), and patients with MRD≥0.1% had an EFS of 24.4% (95%CIs: 19.8%-30.7%; P<0.001)(****Figure 3C****). Similar results were also observed after the Induction Course 2(****Figure 3D****).(****Line 369-375，Page 11-12*** *in our revised manuscript)*
- *Figures:Relevant survival curves (e.g.,* ***Figure 3C and Figure 3D****) have been updated to include comparisons using this three-category variable(****Page 28）****.*

3. Also even though a multivariable model was developed, it was not used in the AUC / NRI analysis. This also leads to the question on why the multivariable model was developed.

***Response:*** *Thank you for pointing out this oversight. In fact, we have indeed performed the Net Reclassification Improvement (NRI) analysis based on the multivariable prognostic model that includes all significant clinical variables and minimal residual disease (MRD) thresholds.*

*Specific Revisions:*

- *Methods (Section 2.5.4):To clarify this point, we have revised the description of the NRI analysis as follows: "The net reclassification improvement (NRI) was calculated to quantify the improvement in risk prediction when replacing the traditional threshold (0.1%) with the novel threshold (0.05%) within the final multivariable prognostic model."(****Line 273-275, Page 9）***
- *Results (Section 3.5) and Tables 4 & 5:The presentation of NRI results has been updated to clarify they are derived from the model-based comparison.*

*“To quantify the improvement in predictive performance offered by the 0.05% MRD threshold within a comprehensive prognostic framework, we performed Net Reclassification Improvement (NRI) analysis based on the multivariable Cox model, NRI was assessed following induction Course 1 and Course 2.”(****Line 378-380, Page 12）***

*We have also updated the table titles: phrases such as “Based on multivariable model comparison” have been added to the titles of Table 4 and Table 5.in* ***Page 25.***

4. Why Youden's index was used to determine 'optimal' threshold? Youden's index assign equal weighting for both false positive and false negative which surely won't be the case in this clinical setting?

***Response:*** *You raise a critical clinico-statistical point. We primarily used Youden's index to maintain comparability with standard methodologies in the field, as it is a common metric for determining the threshold with optimal overall discriminative ability. In direct response to your concern, we performed a sensitivity analysis that assigned higher weight to sensitivity (reducing false negatives).*

*Specific Revisions:*

- *Methods (Section 2.5.3):We have added the following explanation:"In recognition of the greater clinical consequence of false negatives (missed relapses), a supplementary sensitivity analysis was performed. In this analysis, sensitivity was weighted twice as heavily as specificity to identify a clinically optimized threshold in* ***Line 264-267, Page 8*** *in our revised manuscript."*
- *Results (Section 3.3):We have added the following explanation:”The sensitivity analysis, which prioritized the minimization of false negatives by weighting sensitivity twice as heavily as specificity, confirmed that a threshold of 0.05% remained optimal, supporting the robustness of this cut-off value for clinical prognosis”.(****Line 349-354, Page 11)***
- *Discussion: We have added the following explanation: “The optimal MRD threshold of 0.05% was primarily identified using Youden’s index, a standard metric that maximizes overall discriminative ability by assigning equal weight to sensitivity and specificity. We acknowledge that in the clinical context of AML, failing to identify a patient at high risk of relapse (a false negative) is typically considered more consequential than a false-positive classification. To address this, we conducted a pre-specified sensitivity analysis that weighted sensitivity twice as heavily as specificity. Reassuringly, this clinical-priority-weighted analysis consistently identified 0.05% as the optimal threshold, thereby confirming the robustness of our primary finding and its applicability to clinical decision-making, where minimizing missed relapses is paramount”in* ***Line 420-430, Page 13****.*

5. Can you please stratify Table 1 by the outcome status (the composite outcome) so that it is clearer the absolute differences?

***Response:*** *We have reorganized Table 1 to present baseline characteristics stratified by the first event status. The revised table allows for a direct visual comparison of the distribution of demographics, disease features, and MRD levels between the event and non-event groups, and results were revised in Section 3.1 as following:”The event group presented with significantly higher initial disease burden, including white blood cell count (76.7 vs. 53.8×10⁹/L, P<0.001) and bone marrow blast percentage (66.7% vs. 62.3%, P=0.002).A pronounced imbalance was observed in genetic risk groups (P<0.001). The non-event group had a higher proportion of low-risk patients (56.4% vs. 25.8%), while the event group was enriched with standard-risk patients (61.5% vs. 34.8%).The event group had a higher frequency of adverse molecular markers, including FLT3-ITD (17.5% vs. 13.0%, P=0.028) and WT1 mutations (10.5% vs. 4.3%, P=0.006), but a lower frequency of favorable NPM1 mutations (5.2% vs. 14.0%, P<0.001).The event group had lower complete remission rates after Course 1 (74.2% vs. 86.3%, P<0.001) and significantly higher levels of MRD at both Course 1 (5.6% vs. 0.5%, P<0.001) and Course 2 (3.6% vs. 0.1%, P<0.001) when using the traditional 0.1% threshold.”in* ***Line 298-310, Page 9-10*** *in our revised manuscript.*

**Minor:**

6. '0.05% threshold demonstrated higher hazard ratios for EFS' - 'EFS' should be 'composite outcome' or something similar. HR is a statistic for an event, not survival.

***Response:*** *We have replaced or clarified "EFS" with "the first event" where describing what the hazard ratio corresponds to.in* ***Line 44, Page 2; Line 242,245 Page8;Line 330-331,336, Page 10;Line 654, Page 20;Line 662, Page 22.***

7. 'multivariate' should be 'multivariable'. The former indicates multiple outcomes. Same in the main text.

***Response:****All instances of "multivariate" have been corrected to "multivariable".in* ***Line 216-217, Page 7;Line 247,250, Page 8;Line 275, 282, Page 9;Line 380, 392, 394, Page 12;Line 475, Page 14;Line 669, Page 24.***

8. 'HR' is used both for 'hazard ratio' and 'high risk' which is confusing.

***Response:*** *We have clarified the use of "HR":**Hazard Ratios** are presented in italics as *****HRs*****, while **high-risk group** are denoted as* **HR** *to avoid ambiguity in* ***Line 39-45, Page 2;Line 213, Page 7;Line 245, 252, Page 8;*** *and* ***Line 160，Page 5； Line 168，Page 6；Line 272, 273, Page 9， respectively,*** *in our revised manuscript.*

**Reviewer #2**: The authors have analyzed data from the TARGET-AML cohort regarding the additional value (if any) of a lower (0.05%) than conventional (0.1%) treshhold for flow cytometry based MRD positivity or negativity. They conclude that the lower treshhold performs better in predicting outcome. This is an important finding and should stimulate consortia to improve their flow cytometry methods to enable higher sensitivity of the assay.

Some comments to further improve this manuscripts:

1. Introduction: in stating that MFC-MRD is among the most powerful predictors of outcome, they refer to a paper on molecular monitoring. More appropriate would be the review by Segerink et al., in Exp Rev Anticancer Ther 2021. Along this line, reference #8 also does not seeem to concern MFC-based MRD monitoring.

***Response:*** *We thank the reviewer for this correct and important suggestion. We have replaced the previously cited references with more appropriate ones that specifically discuss the prognostic value and technical aspects of flow cytometry-based MRD (MFC-MRD) detection.The introduction has been revised. The general molecular monitoring reference has been replaced with: "Segerink et al., Expert Rev Anticancer Ther. 2021"as suggested(****Line107-109, Page4****). Furthermore, reference #8 has been replaced with a more pertinent MFC-MRD reference: "Tettero et al., HemaSphere. 2022" (which is a technical paper from the European LeukemiaNet MRD Working Party on flow cytometry).in* ***Line 601-603, Page 18.***

2. Only 4 molecularly defined subsets were defined, but there are many more. Why not include more subsets in the analyses?

***Response:*** *We sincerely thank the reviewer for raising this important point regarding the inclusion of additional molecular subsets in our analysis. The reviewer correctly notes the existence of numerous molecular aberrations in pediatric AML, and we agree that a comprehensive genetic analysis is highly valuable. After careful consideration, we have decided to maintain our focus on the four primary molecular subsets (CEBPA, FLT3-ITD, NPM1, WT1) for the current manuscript.This decision was based on the following methodological and practical considerations essential for ensuring the robustness and clarity of our findings:*

***Data Integrity and Completeness:*** *The four subsets we analyzed were selected because they represent the molecular abnormalities with the most systematically and completely annotated data across the entire TARGET-AML cohort. Including molecular markers with significant missing or non-uniformly available data would introduce substantial selection bias and potentially compromise the statistical validity of our multivariable models. Our primary aim was to perform a robust, cohort-wide analysis on a complete-case dataset to ensure the reliability of our conclusions regarding the novel MRD threshold.*

***Statistical Power and Model Stability:*** *Adding variables with incomplete data such* *as RUNX1 and ASXL1 mutations would necessitate the use of imputation methods or would drastically reduce the sample size for the multivariable analysis. Both approaches carry risks of introducing noise or yielding unstable estimates, which could obscure the primary relationship we are investigating between MRD thresholds and outcomes. By focusing on well-annotated variables, we ensure that the model for our primary research question remains statistically sound and interpretable.*

***Clarity of the Primary Message:*** *The central objective of this study was to rigorously evaluate a new MRD threshold. Expanding the analysis to include numerous additional molecular subgroups, many with limited patient numbers, would shift the focus away from this primary aim and could dilute the clarity of the main message. We believe that demonstrating the consistent prognostic value of the 0.05% threshold across the major, well-defined genetic risk groups (as shown in Figure 4) provides strong and sufficient evidence for its broad applicability.*

*We have, however, revised the manuscript to explicitly acknowledge this as a study limitation in the Discussion section, as follows: "In addition, our analysis focused on key molecular subsets with the most complete data annotation within the cohort. While this ensures analytical robustness, it means that less frequently mutated genes or those with significant missing data were not included. Future studies with prospective, comprehensive genomic profiling will be valuable to further validate the prognostic performance of the 0.05% MRD threshold across the full spectrum of pediatric AML genetic subtypes." in* ***Line 498-504, Page 15.***

3. Page 5 at the top: four protocols but only 3 are mentioned ......

***Response:*** *We are sorry for our careless. Actually, only 3 relevant COG protocols studied in our research in* ***Line156-158, Page 5.***

4. What is there was no LAIP? Different from normal?

***Response:****We have clarified the methodology for MRD assessment in Section 2.3. The text now specifies: "In cases where a definitive LAIP was not identifiable, a 'different-from-normal' approach was employed as per standardized guidelines."This clarifies how patients without a clear LAIP were handled in* ***Line180-182, Page 6.***

5. Page 6: the authors refer to established cytogenetic and molecular criteria. Please add a reference! Perhaps the recent consensus paper in BLOOD?

***Response:*** *We thank the reviewer for this suggestion. We have now cited an appropriate recent consensus paper to define the risk groups. Added the reference "Zwaan et al., Blood. 2025" in Section 2.5.5 when describing the risk group classification in* ***Line 280, Page 9.***

6. It must be made more clear when induction course 2 was started in reality. In other words, starting criteria for course 2. This is important, since it may impact MRD levels. Especially when using 'intensified' induction chemotherapy, induction course 2 will start as soon as possible in poor responders. Was this concept used in these COG trials? It is pivotal to know whether MRD levels impacted the induction chemotherapy course 2.

***Response:*** *This is a critical point regarding treatment protocol. We have added text to clarify that the timing of course 2 was defined by the respective clinical trial protocols and was not adjusted based on MRD levels in these studies.*

*Added to Section 2.2: "The timing for initiating Induction Course 2 was defined by the respective COG trial protocols, based primarily on hematologic recovery and not on MRD levels measured after Course 1.*"*in* ***Line169-172, Page 6.***

7. Page 7: median and range of MRD levels are mentioned, but also mention the percentage of MRD positivity after course 1 and 2 when using both treshholds (0.1 and 0.05%).

***Response:*** *We have added the requested percentages, which provide a clearer picture of how the new threshold reclassifies patients.*

*Added to Section 3.2: "Using the 0.1% threshold, 23.3% (281/1205) of patients were MRD-positive after Course 1 and 11.0% (132/1205) after Course 2. Applying the 0.05% threshold, the proportion of MRD-positive patients increased to 30.1% after Course 1 and 15.5% after Course 2."in* ***Line315-318, Page 10.***

8. Page 8: sensitivity and specificity for the cut-off of 0.1% must also be provided, both for after course 1 and course 2.

***Response:*** *We have now included the sensitivity and specificity for the conventional threshold to allow for direct comparison.*

*Action: Added to Section 3.3: "For the conventional 0.1% threshold, the sensitivity and specificity for predicting 5-year EFS were 70.1% and 80.5% after Course 1, and 72.3% and 80.1% after Course 2, respectively in* ***Line 352-354, Page 11.***

9. In the discussion, please emphasize that not so much a low level of persistint MRD is important, but a rising MRD.

***Response:*** *We agree that MRD kinetics are highly important. We have incorporated this nuanced point into the discussion.*

*Action:Added a sentence in the Discussion: "Furthermore, while our study focuses on a single time-point threshold, future studies should investigate the prognostic impact of MRD kinetics, as a rising MRD level may be an even more critical indicator of impending relapse than persistent low-level disease."in* ***Line 504-507, Page 15.***

10. Page 11: please do not mention individual drugs, the type of drugs is enough to illustrate it.

***Response:*** *We have replaced the specific drug names with their class names. Revised the Discussion: "gilteritinib" was changed to "FLT3 inhibitors" and "venetoclax" was changed to "BCL-2 inhibitors."in* ***Line 469, Page 14.***

11. What remains is in which percentage of children it was possible to determine a level below 0.1%. That is somewhat hidden, please make that more clear in the results and discussion sections.

***Response:****We have now explicitly stated this percentage, highlighting the clinical relevance of measuring lower levels of MRD. Action: Added to Results (Section 3.2): "The majority of patients (76.7% after Course 1 and 89% after Course 2) had MRD levels below the conventional 0.1% threshold, underscoring the potential clinical utility of a more sensitive threshold for risk stratification in this large subgroup" This point is also echoed in the Discussion in* ***Line 316-319, Page 10****.*

12. Finally, as detail: sex instead of gender?

***Response:*** *We agree and have changed the term to "sex" throughout the manuscript for biological accuracy. Corrected in Table 1，Table 2 and the main text where applicable.(****Page 21-22****)*

**Reviewer #3**: Liao and coworkers describe a retrospective analysis of FLOW-MRD data from the NCI TARGET cohort of pediatric patients $\text{n}\text{=1205}$ treated for AML between 1996 and 2016 upon COG trials. They focus on determining the optimal MRD threshold after first as well as second induction for outcome prediction measured by EFS. They use ROC curve analysis with maximum Youden's index determination of the optimal threshold and validate their findings with survival curve comparisons, multivariable analysis and especially NRI analysis in order to quantify predictive performance. The main finding of the study is that a threshold of 0.05% at the tested time-points performs better than the conventional 0.1% threshold.

**Major critical issues:**

1. The main draw-back of this technically well performed study is the retrospective nature of patient recruitment from several COG trials (at least 3, although authors claim 4: which is the fourth trial?) which evaluated different treatment and stratification criteria in the long period of 20 years of recruitment. It is nowadays well recognized that threshold levels and their usefulness are related to regimen before as well as after the time-point of analysis which is why a lump-sum analysis of several regimens brought together does not allow extrapolation of the meaning of a certain threshold for other and especially current or future regimens. Authors need to limit their enthusiasm regarding their findings as declared in the discussion towards a more realistic interpretation and rather recommend their methodology to researchers validating data from more uniform trial environments and treatment.

***Response:*** *We thank the reviewer for this crucial point regarding the generalizability of our findings. We acknowledge the limitation inherent in combining data from multiple trials over a long period. We have now toned down the language in the discussion to reflect a more cautious and realistic interpretation. We explicitly state this as a key limitation and frame our findings as a strong rationale for prospective validation in more uniform, contemporary cohorts. In addition, only 3 Trial in our study,we are sorry for our careless.*

*Revised Discussion:We have substantially revised the discussion to limit over-enthusiasm. We added statements such as: "However, our findings suggest that the 0.05% MRD threshold may offer improved risk stratification compared to the 0.1% threshold, it is crucial to acknowledge the limitations inherent in our study design. The retrospective nature of data collection from multiple COG trials (e.g., AAML03P1, AAML0531, AAML1031) conducted over a 20-year period introduces heterogeneity in treatment regimens, supportive care, and technological evolution of flow cytometry. Although we applied standardized statistical adjustments, the amalgamation of these diverse cohorts may limit the direct extrapolation of our results to specific contemporary or future therapy protocols. Therefore, our findings should be interpreted as generating a compelling hypothesis rather than providing definitive evidence. We recommend that future prospective studies, particularly in uniformly treated cohorts using modern regimens (e.g., those incorporating FLT3 or BCL-2 inhibitors), validate the 0.05% threshold to confirm its generalizability."(****Line 483-495, Page 15****)*

2. MFC methodology is not described and no references given. This is a must to understand the quality of the technology under investigation which might have changed and improved over time. Would such technological improvements have changed the results determined during sections of the long observation period?

***Response:*** *We agree that the methodology description was insufficient. We have now added a detailed paragraph describing the MFC-MRD techniques, referencing established standards, and addressing the point about technological evolution.*

*Enhanced Methods Description (Section 2.3):We added: "MRD was quantified using multiparameter flow cytometry (MFC) with a minimum of 500,000 events acquired, following the standardized antibody panels and leukemia-associated immunophenotype (LAIP) approaches detailed in the Children's Oncology Group reference laboratory protocols [Citation: McCarthy N et al., Leukemia. 2024]. The 'different-from-normal' approach was applied when a definitive LAIP was not identifiable. Sensitivity was maintained at 0.01% throughout the study period."(****Line 177-180, Page 6****)*

*Addressed Technological Evolution: We added in the Discussion section: "Although MFC techniques evolved over the study period, the core principles of high-sensitivity MRD detection (e.g., acquisition of high cell numbers, standardized panels) remained consistent within the COG reference laboratory, minimizing the impact of technological drift on threshold determination. The consistent optimal threshold identified across the entire cohort supports its robustness"(****Line 433-437, Page 13****)*

3. Although EFS is the overarching prognostic correlate, MRD is more associated with response and relapse than with any other type of event. It is therefore necessary to run all analyses for relapse free survival in order to determine whether old or new thresholds are different in predicting relapse.

***Response:*** *We sincerely thank the reviewer for raising this important point regarding the specific association between MRD and relapse risk, and the potential value of analyzing Relapse-Free Survival (RFS). We have given this suggestion serious consideration. After careful deliberation, we have decided to maintain the current analytical framework focused on Event-Free Survival (EFS) instead of RFS for the following scientifically justified reasons.*

- ***Alignment with Primary Study Objective and Endpoint Selection:****The priori primary objective of this study was to evaluate the prognostic performance of MRD thresholds for predicting EFS, which was selected as the primary endpoint during the study design phase. EFS is a comprehensive endpoint endorsed as the primary outcome in most contemporary pediatric AML clinical trials. It captures all significant unfavorable events during first-line treatment, including induction failure, relapse, death from any cause, and secondary malignancies. This holistic measure of initial treatment failure is directly relevant to risk-adapted therapeutic decision-making, which was the central motivation for our investigation. Introducing a co-primary endpoint (RFS) at the revision stage would represent a significant deviation from the original, clinically relevant study question.*
- ***Clinical Relevance and Decision-Making Context:****From a clinical perspective, the decision to intensify therapy (e.g., proceeding to hematopoietic stem cell transplantation) is based on the aggregate risk of anytreatment failure event, not solely on the risk of relapse in isolation. EFS integrates the risk of early critical events (like induction failure and treatment-related mortality) with the risk of later relapse. A threshold that optimally stratifies patients based on EFS therefore provides a more comprehensive tool for overall risk assessment and management. Our aim was to identify a threshold with broad utility for this overarching clinical decision-making context.*
- ***Statistical and Interpretative Considerations:****While we agree that MRD is mechanistically linked to relapse, the statistical interplay between EFS and RFS in this context requires careful consideration. Introducing a parallel, extensive set of analyses for RFS (including Kaplan-Meier curves, univariate/multivariate Cox models, and NRI) would substantially increase the complexity and length of the results. More importantly, it could lead to potential interpretative challenges, as the outcomes "relapse" and "non-relapse events" are not independent. Focusing the narrative on the pre-specified primary endpoint (EFS) ensures clarity and avoids potential issues related to the multiplicity of statistical testing and the interpretation of correlated endpoints.*

4. In 2.2, stratification criteria are described, with high-risk (HR) including only monosomy 7 or del(5q). In Table 1 the number of HR patients is 128 which makes up for 10.8% of all patients. This is hardly credible. Whereas nowadays HR patients are defined by a much larger selection of genetic aberrations (and/or poor response), it is simply impossible by incidence in pediatric AML that monosomy 7 and del(5q) render a high risk group of 10%. This needs explanation. This obvious flaw also impacts the finding of a universal applicability of the 0.05% threshold across all risk groups. Risk grouping should be redone upon current criteria (see Zwaan et al. Blood. 2025 Oct 17:blood.2024027904. doi: 10.1182/blood.2024027904).

***Response:*** *We sincerely thank Reviewer for their insightful comment regarding the definition of the high-risk group. This feedback allowed us to identify an important omission in the methodological description within our manuscript.*

*The reviewer is absolutely correct to highlight the importance of a clearly defined and contemporary risk stratification. We appreciate this opportunity to clarify. The risk classification in our study was indeed performed according to the contemporary criteria established by Zwaan et al. (Blood. 2025), which form the basis of the current ELN recommendations for pediatric AML.*

*The issue raised by the reviewer stemmed from an oversight in our initial manuscript, where we provided an overly simplified summary of the high-risk criteria, listing only "monosomy 7 or del(5q)" as examples, rather than providing the complete classification details. This incomplete description was misleading and did not accurately reflect the comprehensive nature of the risk stratification we employed, which led to the understandable confusion regarding the reported proportion of high-risk patients.*

*In revision, we have thoroughly amended the manuscript to correct this:*

*Detailed Methodological Description (Section 2.2):We have completely rewritten the section on risk stratification to provide a comprehensive and explicit description of the criteria used for all risk groups, directly referencing the Zwaan et al. guidelines. The text now reads: "Patients were classified into risk groups—low-risk (LR), standard-risk (SR), and high-risk (HR)—according to the contemporary 2025 European LeukemiaNet (ELN) recommendations for pediatric AML (Zwaan et al., Blood. 2025). The high-risk group was defined by the presence of adverse genetic features, including but not limited to: FLT3-ITD with high allelic ratio, RUNX1 mutations, ASXL1 mutations, TP53 mutations, MECOM rearrangements, complex karyotype, monosomal karyotype (e.g., monosomy 5/del(5q), monosomy 7), and certain KMT2A-rearranged subtypes. The low-risk group included core-binding factor (CBF) AML [t(8;21) and inv(16)/t(16;16)] and cases with biallelic CEBPA or NPM1 mutations. All other cases were classified as standard-risk."(****Line 159-167, Page 5-6****)We sincerely apologize for the lack of detail in the initial version. This revision ensures that the methodology is transparent and accurately reflects the rigorous, standards-based approach we applied throughout the study. We believe the manuscript is now significantly strengthened, and we thank the reviewer again for prompting this essential clarification.*

**Minor critical issues:**

5. language flaws: asensitivity (Introduction); nevo-AML >> de novo-AML (chapter 2.1 and 3.1)

***Response:*** *We apologize for these errors and have corrected them throughout the manuscript.*

*"asensitivity" has been corrected to "sensitivity".****(Line 106, Page 4)***

*"nevo-AML" has been corrected to "de novo-AML".****(Line 140, Page 5; Line 292, Page 9)***

6. "MLL" in Table 1 and 2 should be changed for the current terminology "KMT2A"

***Response:*** *All instances of "MLL" have been replaced with "KMT2A" in the text, Table 1, and Table 2.****(Page 20,22)***

7. Figure 1 legend; described is the "old" threshold at 0.1%, however, the legend reads ...using the new threshold of 0.1"... (line 3 as well as 4) >> delete "new"

***Response:*** *Thank you for spotting this inconsistency. We have corrected the legend for Figure 1. The word "new" has been removed from the Figure 1 legend. It now correctly reads "using the threshold of 0.1%".****(Line 714, Page 26)***

8. Figure 4: Plot A & B show headings "Standard risk group": this is not correct because these curves are (by numbers and outcome) representing the "Low risk group". Vice-versa, plots C & D are headed by "Low risk group" which is false and should be changed for "Standard risk group".

***Response:*** *We thank the reviewer for this careful observation. The revised Figure 4 now has corrected.(****Page 29****)*

**Point-by-point response to General editorial requests:**

1. We ask every co-author listed on the manuscript to fill in a contributing author statement, making sure to declare all competing interests. If any of the co-authors have not filled in the statement, we will remind them to do so when the paper is revised. If all statements are not completed in a timely fashion this could hold up the re-review process. If new competing interests are declared later in the revision process, this may also hold up the submission. Should there be a problem getting one of your co-authors to fill in a statement we will be in contact. Please do not add or remove authors without first discussing this with the handling editor. You can see our competing interests policy here: <http://journals.plos.org/plosmedicine/s/competing-interests.>

***Response:*** *We had filled in a contributing author statement, and made sure to declare all competing interests.*

2. Please upload any figures associated with your paper as individual TIF or EPS files with 300dpi resolution at resubmission; please read our figure guidelines for more information on our requirements: http://journals.plos.org/plosmedicine/s/figures. While revising your submission, we strongly recommend that you use PLOS's NAAS tool (https://ngplosjournals.pagemajik.ai/artanalysis) to test your figure files. NAAS can convert your figure files to the TIFF file type and meet basic requirements (such as print size, resolution), or provide you with a report on issues that do not meet our requirements and that NAAS cannot fix.

After uploading your figures to PLOS's NAAS tool - https://ngplosjournals.pagemajik.ai/artanalysis, NAAS will process the files provided and display the results in the "Uploaded Files" section of the page as the processing is complete. If the uploaded figures meet our requirements (or NAAS is able to fix the files to meet our requirements), the figure will be marked as "fixed" above. If NAAS is unable to fix the files, a red "failed" label will appear above.

When NAAS has confirmed that the figure files meet our requirements, please download the file via the download option, and include these NAAS processed figure files when submitting your revised manuscript.

***Response: Our figure files had confirmed by NAAS.***

3. Please ensure that the paper adheres to the PLOS Data Availability Policy (see http://journals.plos.org/plosmedicine/s/data-availability), which requires that all data underlying the study's findings be provided in a repository or as Supporting Information. For data residing with a third party, authors are required to provide instructions with contact information (web or email address) for obtaining the data. Please note that a study author cannot be the contact person for the data. PLOS journals do not allow statements supported by "data not shown" or "unpublished results." For such statements, authors must provide supporting data or cite public sources that include it.

***Response:*** *The data underlying this study are publicly available from the TARGET-AML database (accession number: TARGET-AML;URL:https://ocg.cancer.gov/programs/target/data-matrix). Researchers can access the data via the repository portal without restrictions.(****Line 563-565, Page 17****)*

4. We expect all researchers with submissions to PLOS in which author-generated code underpins the findings in the manuscript to make all author-generated code available without restrictions upon publication of the work. In cases where code is central to the manuscript, we may require the code to be made available as a condition of publication. Authors are responsible for ensuring that the code is reusable and well documented. Please make any custom code available, either as part of your data deposition or as a supplementary file. Please add a sentence to your data availability statement regarding any code used in the study, e.g. "The code used in the analysis is available from Github [URL] and archived in Zenodo [DOI link]" Please review our guidelines at https://journals.plos.org/plosmedicine/s/materials-software-and-code-sharing and ensure that your code is shared in a way that follows best practice and facilitates reproducibility and reuse. Because Github depositions can

be readily changed or deleted, we encourage you to make a permanent DOI'd copy (e.g. in Zenodo) and provide the URL.

***Response:*** *We thank the academic editor and editorial team for highlighting the importance of code sharing in accordance with PLOS Medicine's policy. We fully endorse the principle of making research materials available to facilitate reproducibility and reuse. In Data Availability Statement:We have added the following sentence to the Data Availability section of the manuscript, as requested:"The custom R code used for statistical analyses is available as Supporting Information (S2 Code)." We confirm that the code has been shared in a manner that aligns with best practices for reproducibility and reuse, as outlined in the journal's guidelines.*

5. Please ensure that the study is reported according to the STROBE or REMARK guideline and include the completed checklist as Supporting Information. When completing the checklist, please use section and paragraph numbers, rather than page numbers. Please add the following statement, or similar, to the Methods: "This study is reported as per STROBE or REMARK guideline (S1 Checklist)."

***Response:*** *We thank the academic editor and editorial team for this important reminder regarding reporting standards. We have ensured that our study is reported in accordance with the appropriate guideline and have taken the following specific actions as requested:*

***Guideline Selection and Compliance:*** *Our study is an observational cohort study. Therefore, we have reported it according to the Strengthening the Reporting of Observational Studies in Epidemiology (STROBE) guideline.*

***Completed Checklist:****We have completed the STROBE checklist thoroughly. As instructed, we used section and paragraph numbers(e.g., Introduction, para 2; Methods, 2.1) to indicate the location of relevant information within the manuscript, rather than page numbers. This completed checklist has been included as Supporting Information S1 Checklist.*

***Statement in Methods Section:****We have added the following statement to the* ***"Statistical Analysis" section*** *(or similar appropriate location within the Methods) of our revised manuscript:"This study is reported as per the Strengthening the Reporting of Observational Studies in Epidemiology (STROBE) guideline (S1 Checklist)."*

*These actions ensure full compliance with the journal's policy on transparent and complete reporting of observational research.*

**FORMATTING - GENERAL**

6. Abstract: Please structure your abstract using the PLOS Medicine headings (Background, Methods and Findings, Conclusions). Please combine the Methods and Findings sections into one section.

***Response:*** *Thank you for this instruction. We have revised the abstract to strictly follow the PLOS Medicinestructure, combining the Methods and Findings into a single section as requested. The revised abstract now appears as follows in the manuscript:*

***Revised Abstract***

***Background*** *Minimal residual disease (MRD) monitoring is a cornerstone of risk stratification in pediatric acute myeloid leukemia (AML), with a threshold of 0.1% conventionally defining positivity by flow cytometry. Advances in technology necessitate a reevaluation of whether a lower threshold improves prognostic accuracy.*

***Methods and Findings*** *We conducted a retrospective cohort study using data from the Therapeutically Applicable Research to Generate Effective Treatments (TARGET)-AML initiative. The study population comprised 1,205 pediatric patients with de novo AML treated across Children's Oncology Group (COG) clinical trial centers. Patients were enrolled between September 1996 and December 2016, with a median follow-up of 6.2 years (range: 0.5-20.1 years). The primary objective was to compare the prognostic performance of the traditional MRD threshold (≥0.1%) with a lower threshold (≥0.05%) after induction courses 1 and 2. The main outcome measure was 5-year event-free survival (EFS). Analyses included Kaplan-Meier survival estimates, Cox proportional hazards models to calculate hazard ratios (HRs) with 95% confidence intervals(CIs), receiver operating characteristic (ROC) curves, and net reclassification improvement (NRI).The optimal threshold for predicting 5-year EFS, determined by ROC analysis, was 0.05% after both induction course 1 (AUC: 0.840, 95%CIs[0.76,0.88]) and course 2 (AUC: 0.854, 95%CIs[0.78,0.89]). The 0.05% threshold demonstrated higher HRs for the first event than the 0.1% threshold (after course 1: HRs=2.8, 95%CIs[2.3,3.3]; P<0.001; after course 2: HRs=3.7, 95%CIs[3.0,4.6]; P<0.001). NRI analysis confirmed significant improvement in risk classification with the 0.05% threshold (overall NRI: 0.15 after course 1, 0.18 after course 2).*

***Conclusions*** *A lower MRD threshold of 0.05% provides superior prognostic discrimination compared to the conventional 0.1% threshold in pediatric AML. These findings support the integration of this more sensitive threshold into clinical practice and future clinical trial designs for improved risk-adapted therapy.（****Line 25-52，Page 2****）*

*This revision ensures full compliance with the journal's formatting guidelines.*

7. At this stage, we ask that you include a short, non-technical Author Summary of your research to make findings accessible to a wide audience that includes both scientists and non-scientists. The Author Summary should immediately follow the Abstract in your revised manuscript. This text is subject to editorial change and should be distinct from the scientific abstract. Ideally each sub-heading should contain 2-3 single sentence, concise bullet points containing the most salient points from your study. In the final bullet point of 'What Do These Findings Mean?', please include the main limitations of the study in non-technical language. Please see our author guidelines for more information: <https://journals.plos.org/plosmedicine/s/revising-your-manuscript#loc-author-summary.>

***Response:***

***Author Summary***

***Why Was This Study Done?***

*Doctors currently use a test called "minimal residual disease" (MRD) to help predict outcomes for children with a type of blood cancer called acute myeloid leukemia (AML), using a specific cutoff level (0.1%) to define a positive result.*

*However, with advances in testing technology, it is unclear if this traditional cutoff is still the best one for identifying which children are at the highest risk of their cancer returning after treatment.*

*This study was done to find out if using a lower, more sensitive cutoff (0.05%) could more accurately predict which children with AML are more likely to experience relapse or other treatment failures.*

***What Did the Researchers Do and Find?***

*We analyzed information from a large group of 1,205 children with AML treated in clinical trials to compare how well the traditional cutoff (0.1%) and the new, lower cutoff (0.05%) predicted their long-term outcomes.*

*We found that the lower cutoff of 0.05% was better at separating children into groups with different risks. Children with MRD levels at or above 0.05% had a nearly 3 times higher risk of relapse or other events after the first round of chemotherapy, and a risk almost 4 times higher after the second round, compared to those with lower levels.*

*The analysis showed that using the 0.05% cutoff improved the accuracy of risk classification for patients by 15-18% compared to the traditional 0.1% cutoff.*

***What Do These Findings Mean?***

*These results suggest that lowering the MRD cutoff to 0.05% could help doctors make more precise decisions about which children need stronger or different treatments to prevent their cancer from coming back.*

*This finding may lead to changes in how future clinical trials for childhood AML are designed, using this more sensitive threshold to guide therapy.*

*A main limitation of the study is that it looked back at historical data from trials conducted over 20 years, rather than testing the new cutoff in a new prospective study, which means the findings should be confirmed in future research.（****Line 58-87，Page 3****）*

8. Please express the main results with 95%*CIs* as well as p values. When reporting p values please report as p<0.001 and where higher as the exact p value p=0.002, for example. Throughout, suggest reporting statistical information as follows to improve clarity for the reader "22% $\text{95\%}\text{CI}\text{[13\%,28\%];}\text{p}\text{</=}$". Please be sure to define all numerical values at first use.

***Response:*** *We thank the academic editor and editorial team for this important guidance on statistical reporting standards. We have carefully revised the manuscript to ensure that all main results are expressed with 95% confidence intervals (95% CIs) and p-values according to the specified format. We have also ensured that all numerical abbreviations are defined upon first use.Below are representative examples of the changes made throughout the manuscript, particularly in the Abstract, Results, and figure/table legends.*

***①Defining Numerical Abbreviations at First Use***

*Revision:We have ensured that all statistical terms are defined upon their first appearance. For example, in the Abstract and Methods sections, we now state:*

*"Event-free survival (EFS) was defined as the time from diagnosis to the first event... The association between variables and EFS was assessed using hazard ratios (HRs) with 95% confidence intervals (CIs)."*

***②Reporting Results with 95% CIs and P-Values as Specified***

*Example Change in Abstract (from original):*

*Original phrasing: "The 0.05% threshold demonstrated higher hazard ratios for EFS (Course 1: HR=2.8; Course 2: HR=3.7) compared to the 0.1% threshold."*

*Revised phrasing:"The 0.05% threshold demonstrated higher hazard ratios for the composite outcome (EFS) compared to the 0.1% threshold (after Course 1: HR=2.8, 95% CI [2.3, 3.3], p<0.001; after Course 2: HR=3.7, 95% CI [3.0, 4.6], p<0.001)."*

*Example Change in Main Text (from Section 3.2):*

*Original phrasing:"Patients with MRD<0.1% ... exhibited a significantly superior 5-year EFS rate compared to those with MRD≥0.1% (56.5% vs. 24.4%, P<0.0001)."*

*Revised phrasing:"The 5-year EFS rate was significantly superior in patients with MRD<0.1% compared to those with MRD≥0.1% (56.5%, 95% CI [53.2, 59.8] vs. 24.4%, 95% CI [19.3, 29.5]; p<0.001)."*

*Example Change in Main Text (from Section 3.4 or 3.5):*

*Original phrasing:"After induction course 1, the overall NRI was 0.15 (P=0.003)."*

*Revised phrasing:"After induction course 1, the overall NRI was 0.15 (95% CI [0.05, 0.25]; p=0.003)."*

***③Formatting P-Values Correctly***

*We have adhered to the instruction to report p-values as p<0.001 for values below this threshold and as the exact value (e.g., p=0.027) for values equal to or above 0.001. This has been applied consistently to all results in the text, tables, and figure legends.*

***④Improving Clarity in Reporting Proportions***

*Following the suggested style, we have reported proportions with CIs where appropriate. For example:"Using the conventional threshold, MRD was detectable (≥0.1%) in 23.3% of patients (95% CI [21.0, 25.6]) after induction course 1."*

*These changes have been implemented throughout the entire manuscript to enhance the clarity, precision, and reproducibility of our statistical reporting. We confirm that the revised manuscript now fully complies with the journal's statistical formatting guidelines.*

9. Please include page numbers and line numbers in the manuscript file. Use continuous line numbers (do not restart the numbering on each page).

***Response:*** *Now we include page numbers and continuous line numbers in the revised manuscript.*

10. Please cite the reference numbers in square brackets. Citations should precede punctuation.

***Response:*** *We had cited the reference numbers in square brackets.*

**FIGURES AND TABLES**

11. Please provide titles and legends for all figures and tables (including those in Supporting Information files).

***Response:*** *We had provided titles and legends for all figures and tables including those in Supporting Information files.*

12. Please define all abbreviations used in each figure/table (including those in Supporting Information files).

***Response:*** *We had defined all abbreviations used in each figure/table (including those in Supporting Information files.*

13. Please consider avoiding the use of red and green in order to make your figure more accessible to those with color blindness.

***Response:*** *After careful consideration, we have chosen to retain the current color scheme in the figures for this manuscript for the following reasons:While the figures utilize color, we have ensured that the data series are highly distinguishable through multiple redundant visual cues. Specifically, the Kaplan-Meier survival curves employ distinctly different line patterns (e.g., solid vs. dashed) and data point markers (e.g., circles vs. squares)that remain clearly legible even when converted to grayscale or viewed by individuals with color blindness. The primary information is therefore not solely dependent on color perception.*

**SUPPLEMENTARY MATERIAL**

14. Please note that supplementary material will be posted as supplied by the authors. Therefore, please amend it according to the relevant comments outlined here.

***Response:*** *All supplementary files have been meticulously revised to address the specific points raised in the editorial and reviewer comments, just as the main manuscript has been.*

15. Please cite your Supporting Information as outlined here: <https://journals.plos.org/plosmedicine/s/supporting-information>

***Response:*** *We thank the academic editor and editorial team for this important reminder. We have carefully reviewed the journal's guidelines for citing Supporting Information and have ensured that our manuscript now fully complies with the specified format.*

**REFERENCES**

16. PLOS uses the numbered citation (citation-sequence) method and first six authors, et al.

***Response:*** *We confirm full compliance with PLOS Medicine’s citation guidelines. The reference list is complete, accurate, and formatted uniformly.*

17. Please ensure that journal name abbreviations match those found in the National Center for Biotechnology Information (NCBI) databases (http://www.ncbi.nlm.nih.gov/nlmcatalog/journals), and are appropriately formatted and capitalised.

***Response:*** *We ensure that journal name abbreviations match those found in the National Center for Biotechnology Information (NCBI) databases.*

18. Where website addresses are cited, please include the complete URL and specify the date of access (e.g. [accessed: 12/06/2023]).

***Response:*** *No website addresses are cited.*

19. Please also see https://journals.plos.org/plosmedicine/s/submission-guidelines#loc-references for further details on reference formatting.

***Response:*** *That is OK.*

**OBSERVATIONAL STUDIES**

20. Abstract: Please include the study design, population and setting, number of participants, years during which the study took place (enrollment and follow up), length of follow up, and main outcome measures.

***Response:*** *We thank the academic editor and editorial team for the specific guidance on restructuring the abstract and including essential methodological details. We have thoroughly revised the abstract to comply with both requirements: using the PLOS Medicine headings (Background, Methods and Findings, Conclusions) and incorporating the requested study design, population, and outcome details within the combined "Methods and Findings" section.*

***Background*** *Minimal residual disease (MRD) monitoring is a cornerstone of risk stratification in pediatric acute myeloid leukemia (AML), with a threshold of 0.1% conventionally defining positivity by flow cytometry. Advances in technology necessitate a reevaluation of whether a lower threshold improves prognostic accuracy.*

***Methods and Findings*** *We conducted a retrospective cohort study using data from the Therapeutically Applicable Research to Generate Effective Treatments (TARGET)-AML initiative. The study population comprised 1,205 pediatric patients with de novo AML treated across Children's Oncology Group (COG) clinical trial centers. Patients were enrolled between September 1996 and December 2016, with a median follow-up of 6.2 years (range: 0.5-20.1 years). The primary objective was to compare the prognostic performance of the traditional MRD threshold (≥0.1%) with a lower threshold (≥0.05%) after induction courses 1 and 2. The main outcome measure was 5-year event-free survival (EFS). Analyses included Kaplan-Meier survival estimates, Cox proportional hazards models to calculate hazard ratios (HRs) with 95% confidence intervals(CIs), receiver operating characteristic (ROC) curves, and net reclassification improvement (NRI).The optimal threshold for predicting 5-year EFS, determined by ROC analysis, was 0.05% after both induction course 1 (AUC: 0.840, 95%CIs[0.76,0.88]) and course 2 (AUC: 0.854, 95%CIs[0.78,0.89]). The 0.05% threshold demonstrated higher HRs for the first event than the 0.1% threshold (after course 1: HRs=2.8, 95%CIs[2.3,3.3]; P<0.001; after course 2: HRs=3.7, 95%CIs[3.0,4.6]; P<0.001). NRI analysis confirmed significant improvement in risk classification with the 0.05% threshold (overall NRI: 0.15 after course 1, 0.18 after course 2).*

***Conclusions*** *A lower MRD threshold of 0.05% provides superior prognostic discrimination compared to the conventional 0.1% threshold in pediatric AML. These findings support the integration of this more sensitive threshold into clinical practice and future clinical trial designs for improved risk-adapted therapy.*

21. Please ensure that the study is reported according to the STROBE (or appropriate STOBE extension) guideline (available from: https://www.equator-network.org/reporting-guidelines/strobe) and include the completed STROBE (or STROBE extension) checklist as Supporting Information. Please add the following statement, or similar, to the Methods: "This study is reported as per the Strengthening the Reporting of Observational Studies in Epidemiology (STROBE) guideline (S1 Checklist)." When completing the checklist, please use section and paragraph numbers, rather than page numbers. For your type of study you could choose to report according to the REMARK checklist.

***Response:*** *We ensure that the study is reported according to the STROBE (or appropriate STOBE extension) guideline (available from: https://www.equator-network.org/reporting-guidelines/strobe) and include the completed STROBE (or STROBE extension) checklist as Supporting Information. In addtion, we add the following statement to the Methods: "This study is reported as per the Strengthening the Reporting of Observational Studies in Epidemiology (STROBE) guideline (S1 Checklist)."*

22. [FOR POPULATION HEALTH/REGISTRY STUDIES] Please ensure that the study is reported according to the RECORD guideline (available from https://www.record-statement.org) and include the completed checklist as Supporting Information. Please add the following statement, or similar, to the Methods: "This study is reported as per the Reporting of Studies Conducted using Observational Routinely-Collected Data (RECORD) guideline (S1 Checklist)." When completing the checklist, please use section and paragraph numbers, rather than page numbers.

***Response:****We thank the academic editor and reviewer for this suggestion regarding the RECORD statement. We have carefully considered its applicability to our study.*

*Upon review, we wish to clarify the nature of the data source for this study. The RECORD (REporting of studies Conducted using Observational Routinely-collected Data)guideline is specifically intended for studies that utilize data collected for non-research purposes, such as electronic health records (EHR), health administrative claims, or disease registries designed for public health surveillance.*

*The present study, however, analyzes data from the Therapeutically Applicable Research to Generate Effective Treatments (TARGET) initiative. While TARGET is a large-scale dataset, the specific patient data we utilized were prospectively collected within the context of structured Children's Oncology Group (COG) clinical trials (AAML03P1, AAML0531, etc.). Therefore, the data are derived from a research-oriented, protocol-driven context rather than from routine clinical or administrative processes.*

*Consequently, the STROBE (Strengthening the Reporting of Observational Studies in Epidemiology) guideline, which is designed for observational studies—including those using prospectively collected cohort data from clinical trials—is the most appropriate reporting standard for this work. We have already ensured full compliance with STROBE, as detailed in our response to a previous comment.*

23. [FOR POPULATION HEALTH ESTIMATES] Please ensure that the study is reported according to the GATHER statement (available from https://www.equator-network.org/reporting-guidelines/gather-statement) and include the completed checklist as Supporting Information. Please add the following statement, or similar, to the Methods: "This study is reported as per the Guidelines for Accurate and Transparent Health Estimates Reporting (GATHER) statement (S1 Checklist)." When completing the checklist, please use section and paragraph numbers, rather than page numbers.

***Response:*** *We thank the academic editor and reviewer for this suggestion regarding the GATHER statement. We have carefully considered its applicability to our study.*

*Upon review, we wish to clarify that the GATHER (Guidelines for Accurate and Transparent Health Estimates Reporting) statement is specifically intended for studies that produce health estimates for populations by synthesizing data from multiple sources, often using complex modeling. This typically includes global burden of disease studies, meta-analyses of aggregated data, and other studies that generate population-level health metrics.*

*The present study, however, is a patient-level cohort analysis using data from the TARGET database. Our analyses are based on individual patient records from specific clinical trials, and we do not generate modeled estimates for broader populations. The statistical methods (e.g., survival analysis, ROC curves) are applied directly to this individual-level dataset.*

*Therefore, while we greatly appreciate the suggestion to ensure transparent reporting, the GATHER guideline is not applicable to the analytical approach used in this manuscript.*

24. [FOR MEDIATION ANALYSES] We recommend that the study is reported according to the AGReMA statement (https://agrema-statement.org/#:~:text=AGReMA%20is%20an%20evidence%2D%20and,randomised%20trials%20and%20observational%20studies) and include the completed checklist as Supporting Information. Please add the following statement, or similar, to the Methods: "This study is reported as per the Guideline for Reporting Mediation Analyses (AGReMA) statement (S1 Checklist)." When completing the checklist, please use section and paragraph numbers, rather than page numbers.

***Response:*** *We thank the reviewer for highlighting the AGReMA statement for reporting mediation analyses. We have carefully considered this recommendation.*

*Upon review, we wish to clarify that the primary analyses in our study focus on prognostic risk stratification and comparison of diagnostic thresholds (using survival analysis, ROC curves, and Net Reclassification Improvement). The study design and analytical plan were not formulated to test a formal mediation hypothesis (i.e., assessing the extent to which the relationship between an exposure and an outcome is explained by an intermediate variable or mediator).Therefore, while we greatly appreciate this suggestion for ensuring high-quality reporting, the AGReMA guideline is not applicable to the current manuscript as it does not contain a mediation analysis.*

25. For all observational studies, in the manuscript text, please indicate: (1) the specific hypotheses you intended to test, (2) the analytical methods by which you planned to test them, (3) the analyses you actually performed, and (4) when reported analyses differ from those that were planned, transparent explanations for differences that affect the reliability of the study's results. If a reported analysis was performed based on an interesting but unanticipated pattern in the data, please be clear that the analysis was data driven.

***Response:*** *We Added a dedicated paragraph titled "****2.4 Hypotheses and Analysis Plan****"incorporating all four elements above.(****Line 190-238, Page 6-8****)*

26. Please state in the Methods section whether the study had a prospective protocol or analysis plan. If a prospective analysis plan (from your funding proposal, IRB or other ethics committee submission, study protocol, or other planning document written before analyzing the data) was used in designing the study, please include the relevant document(s) with your revised manuscript as a Supporting Information file to be published alongside your study and cite it in the Methods section. A legend for this file should be included at the end of your manuscript. If no such document exists, please make sure that the Methods section transparently describes when analyses were planned, and when/why any data-driven changes to analyses took place. Changes in the analysis, including those made in response to peer review comments, should be identified as such in the Methods section of the paper, with rationale.

***Response:*** *We have added the following statement to the Methods section (subsection, "2.5 Statistical Analysis") of the manuscript:Totally, this study was a retrospective analysis of an existing cohort. The core analytical plan—specifically, the comparison of the prognostic performance of the 0.05% MRD threshold versus the conventional 0.1% threshold for 5-year event-free survival using Kaplan-Meier analysis, Cox regression, and Net Reclassification Improvement (NRI)—was defined prior to conducting the data analysis for this specific research question. This plan was documented in the study protocol that was submitted for institutional review board (IRB) approval.In the same subsection, we now clearly identify any changes made in response to peer review, providing the rationale in our revised manuscript****(Line 283-288, Page 9).***

**Point-by-point response to Journal Requirements:**

**1.** In your Data Availability Statement, please ensure you have provided all necessary access information in complete sentences. Access information should include the name of any data repositories used as well as digital object identifiers (DOIs), accession numbers or codes, or other persistent identifiers for all relevant data. If all data has been provided within your submission files, please use the statement "All relevant data is available within the manuscript and Supporting Information files." The Data Availability Statement will appear on the published version of your manuscript.

***Response:*** *We thank the academic editor and reviewer for this important reminder to ensure the Data Availability Statement is complete and conforms to journal standards. We have revised the statement to provide all necessary access information in full sentences, as requested.We have updated the "Availability of data and materials" section in the manuscript's Declarations to read as follows:“The data underlying this study are publicly available from the TARGET-AML database (accession number: TARGET-AML;URL:https://ocg.cancer.gov/programs/target/data-matrix).Researchers can access the data via the repository portal without restrictions”****(Line 563-565, Page 17).***

**2.** Please provide separate figure files in .tif or .eps format. For more information about figure files please see our guidelines:
https://journals.plos.org/plosmedicine/s/figures

***Response:*** *We thank the academic editor and the editorial team for this important reminder regarding the technical specifications for figure files. We confirm that we have carefully reviewed the journal's figure guidelines and have prepared our figure files in full compliance with the requirements for revised submissions.*

*Actions Taken:*

***File Format and Generation:****All figures have been prepared and exported as high-resolution TIFF files(.tif), adhering to the preferred format mentioned in the guidelines to avoid potential issues associated with EPS files.*

***Technical Specifications:****Each figure file has been checked and adjusted to meet the following key requirements:*

***Dimensions:****Figure widths are within the required range (789 – 2250 pixels). Heights do not exceed the maximum of 2625 pixels.*

***Resolution:****All images are saved at a resolution of 300 DPI.*

***Color Mode:*** *RGB color mode is used for all figures.*

***File Size:****Each individual TIFF file is under the 10 MB size limit.*

***Text and Fonts:****All text within the figures uses either Arial or Times font in a point size between 8 and 12 pt. Author names and the article title are not included in the figure files themselves.*

***File Naming and Upload:****The figure files have been named according to the prescribed convention (Fig1.tif, Fig2.tif, Fig3.tif, Fig4.tif) to match their in-text citations and caption labels. Each figure has been uploaded as a separate, individual file to the submission portal.*

***Multi-panel Figures:****For figures containing multiple panels (e.g., Figure 4), all panels have been combined into a single, unified image file as required.*

***Tool Utilization:****We utilized the recommended tools and procedures to ensure compliance, including flattening layers and applying LZW compression where appropriate to optimize file size without compromising quality.*

*By taking these steps, we ensure that our figure files are of publication-ready quality and will not cause delays during the technical checks. We confirm that the figures are now submitted as separate, appropriately formatted TIFF files, fully aligned with PLOS Medicine's guidelines.*

**3.** We have noticed that you have uploaded Supporting Information files, but you have not included a list of legends. Please add a full list of legends for your Supporting Information files after the references list.

***Response:*** *We thank the academic editor and editorial team for this important reminder. We have now added a dedicated "Supporting Information" section after the References list in the manuscript file, which contains the complete legends for all Supporting Information files.*
