# Supplementary material for: Optimal minimal residual disease threshold in pediatric acute myeloid leukemia: A retrospective cohort study based on the TARGET database
Source: PLoS Med. 2026 May 8;23(5):e1005088. doi: 10.1371/journal.pmed.1005088 (PMC13155632; doi:10.1371/journal.pmed.1005088)
Supplement: Attachment — Submitted filename: point to point response 2.docx [file pmed.1005088.s007.docx]

Dear Dr. Evangelia Fourli, Ph.D.
Associate Editor

***Plos Medicine***

2026-4-16

We would like to thank the editor for the decision letter and advice on our manuscript entitled “**Optimal Minimal Residual Disease Threshold in Pediatric Acute Myeloid Leukemia: A Prognostic Reevaluation Based on the TARGET Cohort**(Manuscript ID: PMEDICINE-D-25-04201R2)”.

We also thank the reviewer for the constructive comments and suggestions. We have strived our best to answer all the queries accordingly. Our point-by-point response to the comments are listed below this letter. In addition, we have revised the manuscript accordingly, and the amendments are indicated by red font in the revised manuscript.

We hope that our revised manuscript is now acceptable for publication in ***Plos Medicine*** and we look forward to hearing from you soon.

Yours sincerely,

Kun-yin Qiu

**Point-by-point response to the editorial requests**

**GENERAL EDITORIAL REQUESTS**

1. **Author Summary**:

**Comment**: "At this stage, we ask that you include a short, non-technical Author Summary... The Author Summary should immediately follow the Abstract."

**Response**: We have added an Author Summary section after the Abstract, as requested. The summary is structured with bullet points under the subheadings "Why Was This Study Done?", "What Did the Researchers Do and Find?", and "What Do These Findings Mean?" including the main limitations in non-technical language. The content is distinct from the scientific abstract.(Page 3, Line 58-87).

2. **Title Compliance:**

**Comment:** "Please confirm that your title complies with PLOS Medicine's style."

**Response:** We confirm that the title is non-declarative and not a question. It begins with the main concept, and the study design ("A retrospective cohort study") is included in the subtitle after a colon. The revised title is: "Optimal Minimal Residual Disease Threshold in Pediatric Acute Myeloid Leukemia: A Retrospective Cohort Study Based on the TARGET Database."(Page 1, Line 1-2).

3. **Abstract Compliance:**

**Comment:** "Please confirm that your abstract complies with our requirements, including format (three sections: Background, Methods and Findings, and Conclusions)."

**Response:** We have ensured the abstract follows the required structure: Background, Methods and Findings, and Conclusions. (Page 2, Line 25-52).

4. **Introduction Clarity:**

**Comment:** "Please ensure that the Introduction ends with a clear description of the study question or hypothesis."

**Response:** The Introduction now ends with a clear statement of the study question: "This study aimed to: (1) compare the prognostic performance of the 0.1% vs. 0.05% MRD thresholds... (2) quantify improvement via NRI; and (3) validate the threshold across genetic risk subgroups." (Page 5, Line 128-134).

5. **Abbreviations**:

**Comment:** "Please ensure that all abbreviations are defined at first use throughout the text."

**Response:** We have reviewed the manuscript and defined all abbreviations at first use (e.g., MRD, AML, EFS, NRI).

6. **Confirm all numbers:**

**Comment:** Please confirm that all numbers presented in the abstract are present and identical to numbers presented in the main manuscript text.

**Response:** I can confirm that all numbers presented in the abstract are present and identical to the numbers presented in the main manuscript text.

#### ****GENERAL REQUESTS****

1. **Novelty Claims:**

**Comment:** "Please review your text for claims of novelty or primacy and remove this language."

**Response:** We have removed any language implying novelty and ensured statistical terms (e.g., "significant") are supported by data.

2. **Discussion Subheadings:**

**Comment:** "Please remove the 'conclusions' subheading from the discussion. Please also remove any other subheadings from the discussion."

**Response:** We have removed all subheadings from the Discussion section, including "Conclusions." The text now flows as a continuous narrative.

3. **Statistical Reporting:**

**Comment:** "Please revise statistical reporting throughout: use commas for CIs, repeat statistical definitions for each set of parentheses."

**Response:** We have revised statistical reporting as requested. For example, "95% CI [13, 28]" instead of hyphens, and HR/CI are defined for each instance. This has been applied to the text, tables, and figures.

4. **Abstract Limitations:**

**Comment:** "In the last sentence of the Abstract Methods and Findings section, please describe the main limitation(s)."

**Response:** We have added: "The main limitation of this study is its retrospective design using historical data from trials conducted over 20 years, which may limit generalizability to contemporary treatments."

5. **Author Summary Formatting:**

**Comment:** "In the author summary, please revise formatting and ensure you use bullet points."

**Response:** The Author Summary now uses bullet points for each subheading, as shown in the cleaned manuscript.

6. **Analyses Performed Section:**

**Comment:** "Paragraph 2.4.3 Analyses Performed: please add bullet points or a type of numbering."

**Response:** We have added numbering to the list of analyses in section 2.4.3 for clarity.

7. **Line 228 Correction:**

**Comment:** "Line 228, remove 'A' and substitute 'was' with 'were'."

**Response:** Corrected to: "Modifications were made to the initial analysis plan..."

8. **Spacing Issues (Lines 273 and 319):**

**Comment:** "Line 273, insert a space between ' not).' and 'The'. Same for line 319 'subgroup.Applying'."

**Response:** We have added spaces: " not). The" and "subgroup. Applying".

9. **S1 Checklist Punctuation:**

**Comment:** "Please revise S1 Checklist for punctuation errors."

**Response:** We have reviewed and corrected punctuation in the S1 Checklist file.

10. **Causality Language**:

**Comment**: "Your study is observational... remove language that implies causality."

**Response**: We have replaced causal language throughout the manuscript.

#### ****FUNDING STATEMENT****

**Funding Details**:

**Comment**: "The funding statement should include specific grant numbers, URLs, and role of funders."

**Response**: We have updated the Funding Statement in the manuscript with grant numbers, author initials, and URLs. We added: "The funders had no role in study design, data collection and analysis, decision to publish, or preparation of the manuscript."

#### ****COMPETING INTERESTS STATEMENT****

**Competing Interests**:

**Comment**: "All authors must declare competing interests."

**Response**: We confirm that all authors have declared no competing interests in the manuscript: "None."

#### ****DATA AVAILABILITY****

**Data Availability Statement (DAS)**:

**Comment**: "The DAS requires revision... provide details on data access."

**Response**: We have revised the DAS to state: "The data underlying this study are publicly available from the TARGET-AML database (accession number: TARGET-AML; URL: <https://ocg.cancer.gov/programs/target/data-matrix>). Researchers can access the data via the repository portal without restrictions."(P17, Line 559-563).

#### ****ETHICS AND CONSENT****

**Informed Consent Clarification**:

**Comment**: "Please clarify whether informed consent was waived due to de-identified data."

**Response**: We have added in the Ethics section and Methods section: "The original data collection within the Children’s Oncology Group (COG) trials was approved by the Institutional Review Boards (IRB) of all participating centers, including the lead IRB at the National Cancer Institute, with written informed consent obtained from all participants or their guardians. The analysis of de-identified data for this specific study was reviewed and approved by the IRB of Sun Yat-sen Memorial Hospital (Approval No. SYSEC2-2025-BA-885). "

**IRB Citation:**

**Comment:** "Please cite the original paper reporting those IRBs."

**Response:** We have cited the original COG trial publications in the Ethics statement for IRB approval.

#### ****FIGURES****

**Figure Revisions**:

**Comment**: "Show graph axes beginning at zero; provide number at risk in Kaplan-Meier curves; specify statistical tests in legends."

**Response**: Kaplan-Meier curves had already included numbers at risk in the present figure, and legends had already specify the log-rank test for p-values. However, the survival analysis generated by our statistical software cannot set the coordinate axis starting from zero, and we do not know how to add an axis break symbol. Could you please help us insert the axis break symbol on the axis?

#### **ACKNOWLEDGMENTS**

**Acknowledgments Section:**

**Comment:** "Please include an Acknowledgments section without funders or authors."

**Response:** We have added an Acknowledgments section thanking contributors not involved in the study, excluding funders and authors.

### ****Response to Reviewers' Comments****

#### ****Reviewer #1**:**

**Comment**: "Thank you for addressing my comments. I have no further comments. Congratulations on completing this study."

**Response**: We thank the reviewer for their positive feedback.

#### ****Reviewer #2****:

**Comment**: "The revision is fine with me, all my comments have been addressed appropriately."

**Response**: We appreciate the reviewer's approval.

#### ****Reviewer #3****:

**Comment**: The reviewer suggested wording changes to better reflect limitations. We have implemented all four changes as requested:

**Abstract Conclusions (Lines 49-52)**:

**Original**: "Conclusions A lower MRD threshold of 0.05% provides superior prognostic discrimination compared to the conventional 0.1% threshold in pediatric AML. These findings support the integration of this more sensitive threshold into clinical practice..."

**Revised**: "Conclusions A lower MRD threshold of 0.05% provides superior prognostic discrimination compared to the conventional 0.1% threshold in pediatric AML treated in previous COG trials. These findings support testing this more sensitive threshold in future clinical trial designs for improved risk-adapted therapy."

**Discussion (Page 14, Lines 466-467)**

**Original**: "Perhaps the most compelling evidence for the universal applicability of the 0.05% threshold is its consistent prognostic validity across all genetic risk subgroups..."

**Revised**: "Perhaps the most compelling evidence for the universal applicability of the 0.05% threshold is its consistent prognostic validity across all tested genetic risk subgroups."

**Discussion (Page 15, Lines 499-501)**:

**Original**: "While this ensures analytical robustness, it means that less frequently mutated genes or those with significant missing data were not included."

**Revised**: "While this ensures analytical robustness, it means that less frequent genetic aberrations or those with significant missing data were not included."

**Conclusion (Page 16, Lines 514-515)**:

**Original**: "Adopting this more sensitive threshold will enable more precise risk-adapted therapy, ultimately improving outcomes for children with AML."

**Revised**: "Adopting this more sensitive threshold may enable more precise risk-adapted therapy, ultimately improving outcomes for children with AML."

**Response to technical requests**

**Comment:** Thank you for stating "The data in the current study are available from the corresponding author on reasonable request". Please note that the contact for accessing your data cannot be an author on the manuscript. Please therefore replace the current contact information with a third party contact, such as an institutional data access committee.

**Response:** The data underlying this study are publicly available from the TARGET-AML database (accession number: TARGET-AML;URL:https://ocg.cancer.gov/programs/target/data-matrix). Researchers can access the data via the repository portal without restrictions.The custom R code used for statistical analyses is available as Supporting Information (S2 Code).The study protocol was included as Supporting Information (S3 Study Protocol).(revise in P17, Line 562-567)
